# Silyl Radicals as Single-Electron Reductants: α-Aminoalkyl Radical Formation via a Photocatalytic Oxidatively Initiated Radical Chain Process

**DOI:** 10.1021/jacs.4c08230

**Published:** 2024-09-16

**Authors:** Harry C. Waller, Matthew J. Gaunt

**Affiliations:** Yusuf Hamied Department of Chemistry, University of Cambridge, Cambridge CB2 1EW, United Kingdom

## Abstract

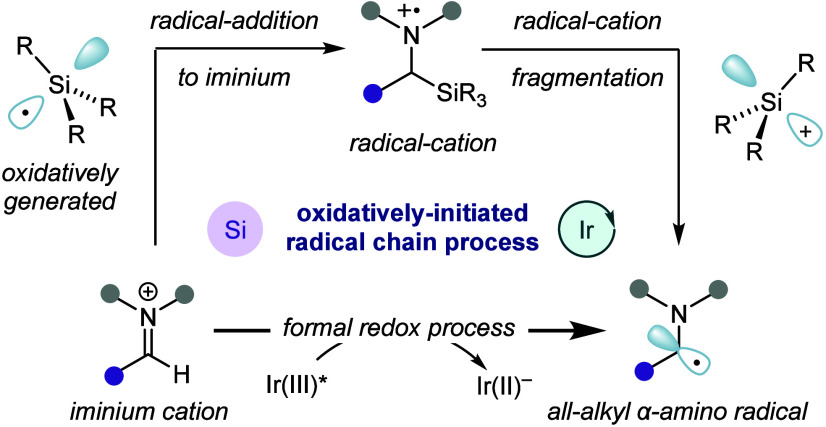

The α-amino-radical constitutes a versatile reactive intermediate that has been used to great effect in the synthesis of complex amine-containing products. Here, we report the development of a multicomponent photocatalytic platform enabling access to all-alkyl α-amino-radicals, exploiting the oxidative formation of silyl-radicals from commercially available tris(trimethylsilyl)silane. A key design element of the new process involves the role of silyl-radicals in generating α-amino-radicals from iminium ions as part of an oxidatively initiated photocatalytic radical chain process. This distinct activation mode is showcased by engaging the ensuing radicals in cross-radical coupling with persistent arene radical anions, enabling the arylation of in situ-generated all-alkyl iminium ions to furnish alkyl-substituted benzylamines.

Photoredox catalysis represents a powerful platform for the generation of radical intermediates under mild reaction conditions.^1,2^ Among these, the α-amino-radical has found broad application in the synthesis of structurally diverse amine-containing products^3^—functional features that are ubiquitous in bioactive molecules.^4^ Many prominent methods for α-amino-radical formation effectively exploit native amine functionality, such as through amine oxidation followed by α-deprotonation or direct hydrogen atom transfer, although these activation modes can lack regioselectivity when similar C–H bonds are present.^5−8^ Regiospecific α-amino-radical generation methods have, therefore, been developed for a broad range of C–C bond-forming transformations. In these cases, the amine starting material must be equipped with a preinstalled activating group through which regiospecific, photochemically induced oxidative fragmentation can be triggered.^9−15^ Single-electron reduction of in situ-generated imines or iminium ions provides an alternative, more modular approach for regiospecific α-amino-radical generation. While reactivity-augmented imines derived from anilines and benzaldehydes have been explored in a range of transformations through this activation mode,^3,16−20^ unbiased all-alkyl α-amino-radicals formed through these processes have only been exploited in addition to alkenes (Figure 1A).^21−25^ Reflecting on the current state of the art for photocatalytic α-amino-radical formation, we reasoned that a regiospecific catalytic process that merges the broader reactivity displayed by oxidative fragmentation strategies with the modularity of the single-electron reduction approach would potentially advance the capabilities of photochemically generated α-amino-radicals. Guided by our group’s recent work on the addition of alkyl-radicals into all-alkyl iminium ions,^26^ we questioned whether silyl-radicals might undergo an analogous addition process to pave the way for a hybrid method for α-amino-radical generation. In our design plan, a photocatalytically generated silyl-radical would add to an in situ-generated iminium ion to afford an α-silyl aminium radical-cation. Fragmentation of this species would provide the desired α-amino-radical in a regiospecific manner. In this way, the silicon-centered radical would formally act as a single-electron reductant for the iminium ion to furnish the α-amino-radical through a visible light-mediated radical chain pathway—a process which, to the best of our knowledge, has not been previously explored (Figure 1B). Studer and co-workers have reported a photocatalytic synthesis of α-amino-silanes from secondary amines and aldehydes,^27^ however a key mechanistic question facing our strategy was the challenge of generating a silyl-radical through a single-electron oxidation event while orchestrating a pathway for carbon–carbon bond formation. Importantly, such an activation mode would enable α-amino-radical functionalization and photocatalytic turnover to be unified by a solitary single-electron reduction event. Throughout the design process, we had recognized that current methods for the photocatalytic arylation of imines via their α-amino-radicals exploit cross-radical coupling with persistent arene radical anions, requiring both reactive intermediates to be generated by single-electron reduction. Such a mechanism has enabled the photocatalytic arylation of *N*-aryl imines derived from benzaldehydes and acetophenones.^28,29^ A benchmark approach, developed by Rovis and co-workers, starts from iminium chloride salts or *O*-benzoyl ketoximes and exploits a broad range of heteroaryl cyanoarene arylating reagents, providing direct access to a variety of primary benzhydryl amine products.^30−32^ Benzaldehyde and acetophenone carbonyl reactants are pervasive throughout these approaches—a feature that might be attributed to the photocatalytic reduction of both the cyanoarene and the iminium ion necessitating the use of longer-lived benzylic α-amino-radicals. Accordingly, we questioned whether our hypothesized radical chain process, in which the photocatalytic reduction is confined to the arene, might enable expansion of this reactivity space.

**Figure 1:**
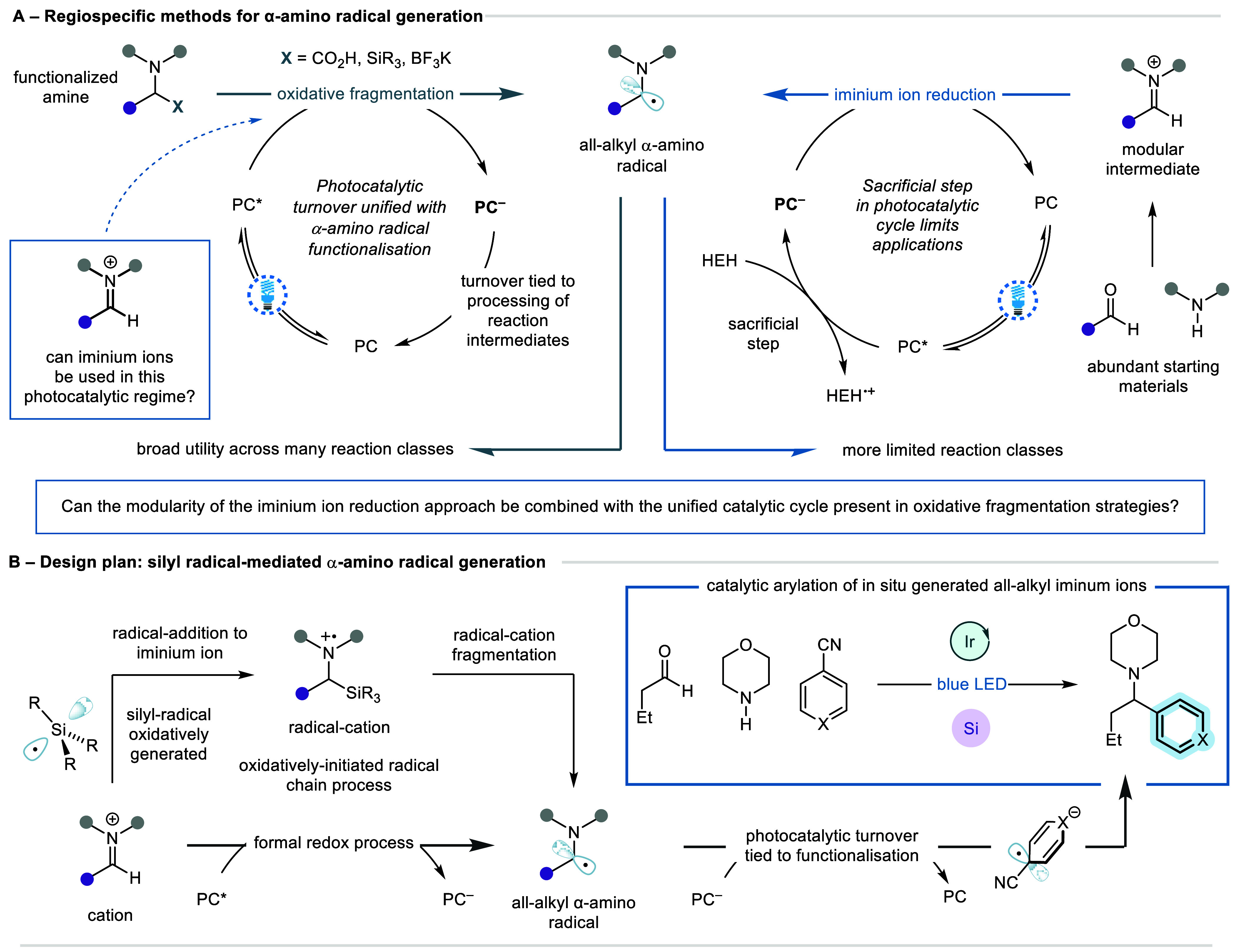
A) Methods for α-amino-radical generation. B) This work: silyl-radical-mediated α-aminoalkyl-radical generation.

Herein, we report the successful realization of this distinct activation mode. Through the cross-radical coupling of the resultant α-aminoalkyl-radicals with reductively generated persistent cyanoarene-based radical anions, previously intractable alkyl-aldehyde and secondary amine feedstocks are utilized to generate a range of α-alkyl-benzylamines—important motifs in many classes of bioactive molecule.

The feasibility of the proposed radical chain process was explored using 1,4-dicyanobenzene (1,4-DCB) as an arylating reagent, alongside commercially available tris(trimethylsilyl)silane ((Me_3_Si)_3_Si–H) as the silane source.^26^ We reasoned that the silyl-radical could be generated through a hydrogen atom transfer (HAT) process with an electrophilic thiyl-radical, itself derived from 2-naphthalenethiol (2-NapSH)—a non-malodorous reagent. The proposed mechanism would proceed via deprotonation of 2-NapSH to afford thiolate **1**, which reductively quenches the triplet excited state of the photocatalyst to form thiyl-radical **2**. Subsequent HAT with (Me_3_Si)_3_Si–H would afford silyl-radical **3**. This species would then undergo a radical exchange process with the in situ-generated iminium ion **4** to generate α-amino-radical **6**. Overall, the (Me_3_Si)_3_Si• formally acts as a single-electron reductant. We envisaged two pathways through which this would proceed: first, by means of an addition–elimination mechanism proceeding via α-silyl aminium radical-cation **5**; or second, through a single-electron transfer from silyl-radical **3** to iminium ion **4**. The photocatalytic cycle is completed through oxidative turnover by 1,4-DCB to generate radical anion **7**, which undergoes cross-radical coupling with α-amino-radical **6** to afford product **8** (Figure 2A). Based on this hypothesis, we were pleased to find our initial experiments, involving irradiating a DMA solution of butyraldehyde **9a**, morpholine **10a**, 1,4-DCB **11a**, (Me_3_Si)_3_Si–H, 1,1,3,3-tetramethylguanidine (TMG), 2-NapSH, and Ir(ppy)_3_, afforded benzylamine **12a** in 35% assay yield (Figure 2B, Entry 4). With unreacted 1,4-DCB accounting for the remaining mass balance, it was thought that inclusion of an additive which could either activate 1,4-DCB toward single-electron reduction or increase the lifetime of anionic intermediate **7** might facilitate reaction progression. Gratifyingly, inclusion of the hydrogen-bond donor 1,3-diphenylurea in these reaction conditions greatly improved reaction efficiency, affording **12a** in 85% assay yield (vide infra).

**Figure 2:**
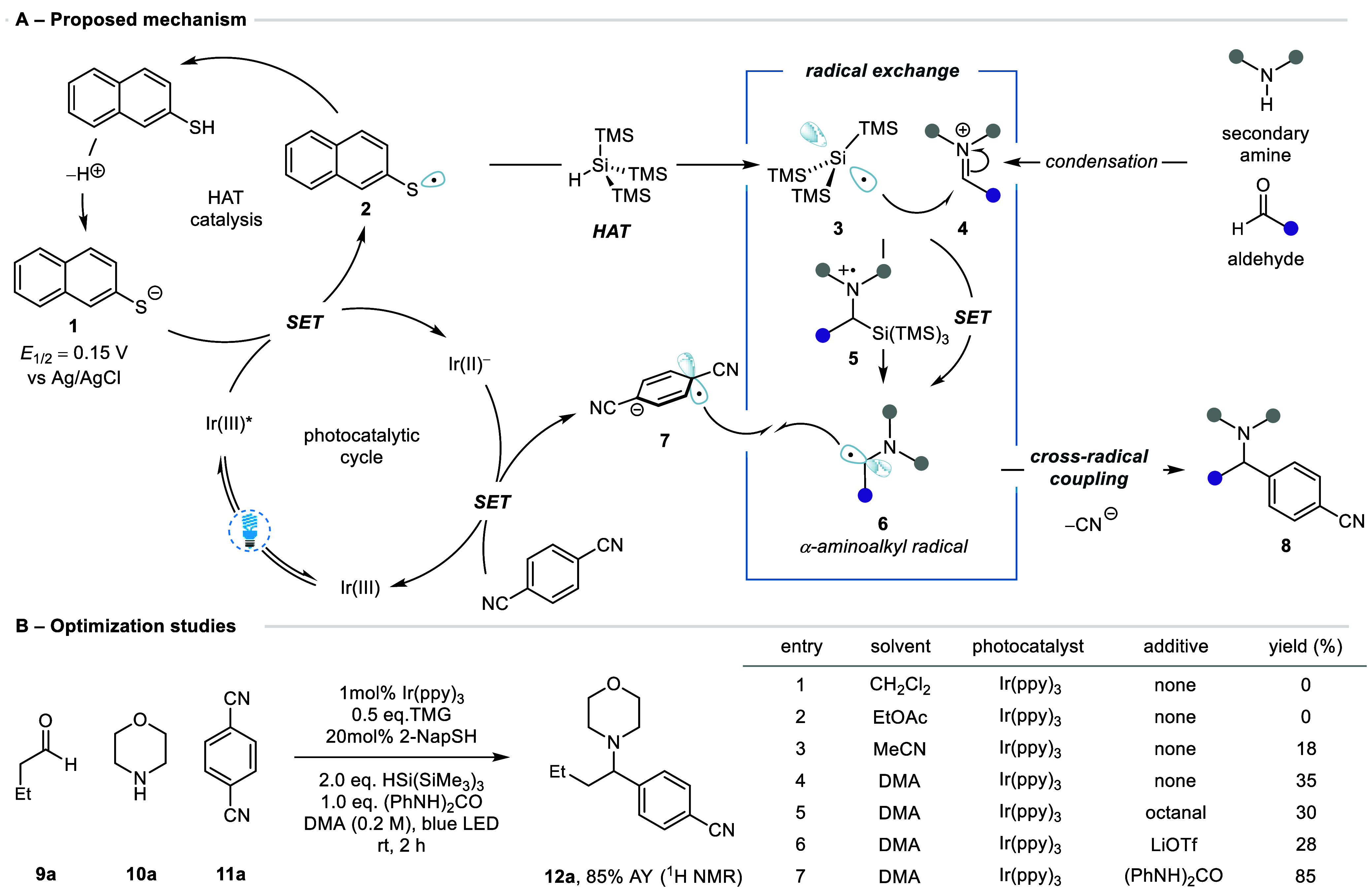
A) Proposed mechanism. B) Optimization studies.

To support the proposed mechanism for this new reaction pathway, Stern–Volmer quenching experiments indicated a strong quench of the photocatalyst by a mixture containing 2-NapSH and TMG (Figure 3A)—a feature attributed to facile oxidation of the thiolate anion (*E*_1/2_ = 0.10 V vs Ag/AgCl—Figure 3B). This is indicative of a reductive quenching cycle with oxidative catalyst turnover mediated by 1,4-DCB. Should the only role of the silane be to regenerate thiolate **1** to facilitate the photocatalytic reductive quenching process, then the use of stoichiometric thiolate would still enable product formation in the absence of the silane. However, removal of (Me_3_Si)_3_Si–H from the reaction mixture, while using 2 equiv of 2-NapSH and TMG, afforded none of **12f** (Figure 3C). If the silane was critical in generating the α-amino-radical, then the addition of a reagent which efficiently scavenges the silyl-radical should preclude formation of **12f**. Accordingly, inclusion of α-methylstyrene **13** in an otherwise standard reaction afforded none of **12f**. Instead, product **14**, derived from (Me_3_Si)_3_Si• addition to olefin **13**, was formed in quantitative yield (Figure 3D). In addition to providing evidence for the intermediacy of both silyl-radical **3** and radical-anion **7**, the inability to detect products derived from the α-amino-radical suggests an absence of this intermediate under these conditions. These results imply the involvement of (Me_3_Si)_3_Si• in α-amino-radical formation. If the cycle was disrupted at the thiyl-radical, then this would inhibit formation of (Me_3_Si)_3_Si• and should, in turn, inhibit α-amino-radical generation. Inclusion of TEMPO in the standard reaction conditions resulted in only the TEMPO-thiyl-radical adduct **15** being detected by quadrupole time-of-flight (QTof) high resolution mass spectrometric analysis of the crude reaction mixture (Figure 3D). The absence of (Me_3_Si)_3_Si• and α-amino-radical adducts indicates that these species are likely formed as part of a chain process which begins with oxidative formation of the thiyl-radical. Together, these experiments support the role of the photochemically generated silyl-radicals in the formation of the required α-amino-radical.

**Figure 3:**
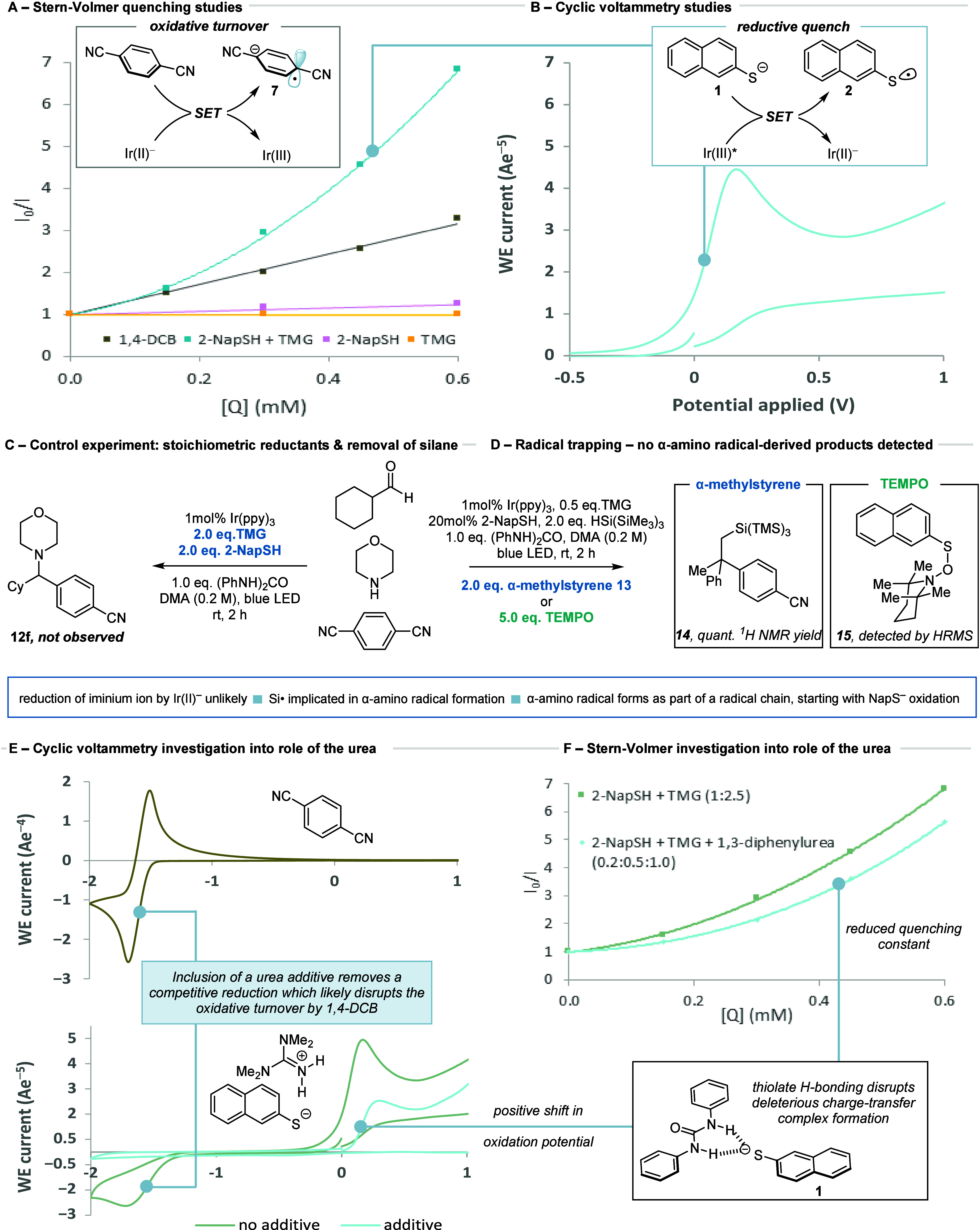
A) Stern–Volmer quenching studies. B) Cyclic voltammogram of 2-NapSH+TMG. C) Use of stoichiometric reductant. D) Radical trapping. Role of 1,3-diphenylurea: E) Cyclic voltammetry; F) Stern–Volmer quenching.

Finally, the role of the crucial 1,3-diphenylurea additive was explored. The cyclic voltammogram of a mixture containing 2-NapSH and TMG indicated the presence of a reduction event (*E*_1/2_ = −1.55 V vs Ag/AgCl) which was coincident with the reduction potential of 1,4-DCB—a feature attributed to the formation of a charge-transfer complex within the guanidinium-thiolate salt. We reasoned that the high 1,4-DCB recovery observed in the absence of the urea was a consequence of this competitive reductive process disrupting the formation of 1,4-DCB radical anion **7**. Addition of the urea into this mixture revealed complete suppression of this reduction process alongside a small increase in oxidation potential, consistent with hydrogen-bonding to thiolate anion **1** (Figure 3E). Indeed, the 2-NapSH–TMG mixture displayed an attenuated reductive quench in the presence of the urea (Figure 3F). Consequently, we believe 1,3-diphenylurea suppresses a competitive reduction event by disrupting a deleterious charge transfer complex through hydrogen-bonding to thiolate anion **1**, facilitating formation of 1,4-DCB radical anion **7** (see the Supporting Information (SI) for further discussion).

With a clearer understanding of the new reaction pathway, the scope of the reaction was explored. For the carbonyl component, a range of functional cyclic and acyclic alkyl-aldehydes (**12a**–**12j**) were amenable to the multicomponent arylation reaction (Figure 4A). Similarly, functional alkyl-substituted cyclic and acyclic secondary amines (**12k**–**12t**) were shown to be competent reaction partners (Figure 4B). As a representative aniline, *p*-anisidine proved to be an effective substrate, affording PMP-protected amine **12t** in good yield. In expanding the capability of the carbonyl component, we found that some cyclic ketones were competent reaction partners (**12u**–**12v**), although they displayed lower reactivity compared to their aldehyde counterparts (Figure 4C).

**Figure 4:**
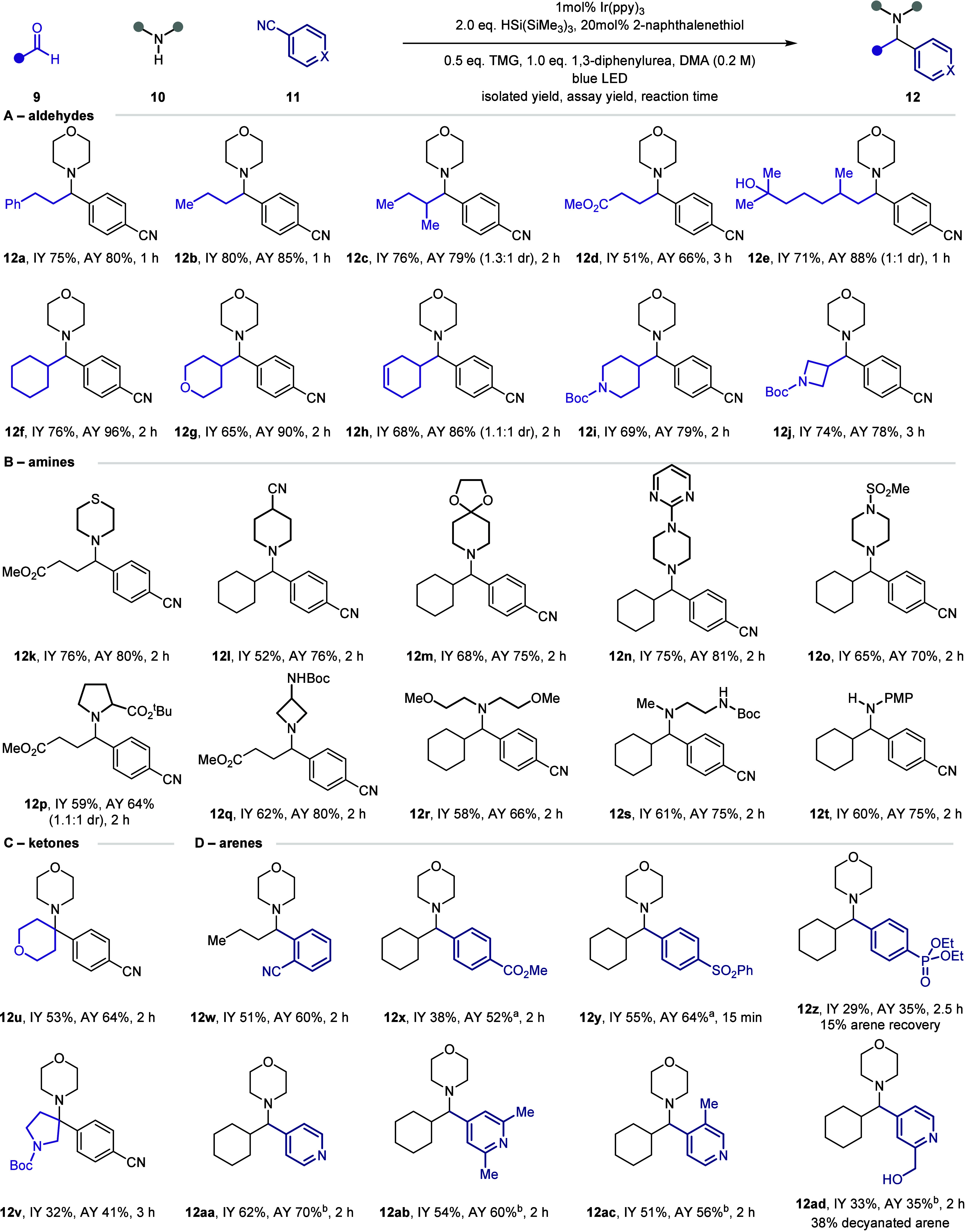
Reaction scope. Reactions performed on 0.2 mmol scale. Assay yields determined through quantitative ^1^H NMR assay against 1,1,2,2-tetrachloroethane as an internal standard. Aldehyde:Amine:Arene (3:3:1). ^a^Hantzsch ester used as the reductant (see the SI). ^b^No 1,3-diphenylurea.

Finally, the scope of the arene component was explored (Figure 4D). In addition to the 1,4-isomer (**12b**), 1,2-dicyanobenzene was a competent substrate under the standard reaction conditions (**12w**). A range of electron-withdrawing groups, including esters (**12x**), sulfones (**12y**), and phosphates (**12z**), were also shown to display reactivity. Pleasingly, we found that 4-pyridinecarbonitrile afforded product **12aa** in good yield, and that substitution could be tolerated in all positions (**12ab**–**ac**). Even 2-(hydroxymethyl)isonicotinonitrile showed reactivity (**12ad**), offering the potential to a wider scope of cyanopyridine arylating reagents.^31,32^

Throughout the course of these studies, benzhydryl amine products derived from the use of benzaldehyde could not be isolated; instead, deaminated cross-coupling product **19a** was always formed. Product **19a** was also formed in 60% ^1^H NMR yield by exposing benzhydryl amine **16** to the standard reaction conditions, implicating this species as an intermediate along the reaction pathway in this case. We were intrigued as to whether this reactivity could be expanded to alkyl-aldehydes. Exposing the standard reaction mixture to prolonged irradiation showed that a slow deamination reaction ensued to afford alkylbenzene products **19a**–**h** (Figure 5). This process is believed to proceed through single-electron reduction of the cyanoarene to radical anion **17**, followed by spin-center shift to the benzylic radical **18**, which is quenched by HAT with (Me_3_Si)_3_Si–H—a particularly rapid process with benzaldehyde owed to the stability of benzhydryl radical **18**. The overall process—from aldehyde to alkylbenzene—constitutes a photocatalytic deoxygenative arylation reaction, in which the aldehyde formally acts as an alkyl-radical precursor. Such a process renders carbonyl compounds a complementary and vast feedstock alongside alkyl halides and boronic acids for photocatalytic cross-coupling reactions.^33,34^ While electrochemical deoxygenative cross-couplings (proceeding via the benzylic alcohol) are known,^35^ such a photocatalytic cross-coupling process has not been previously reported, to the best of our knowledge. A preliminary evaluation of the reaction showed that a range of functional aldehydes were amenable to the process (**19a**–**h**). The use of heteroaryl-aldehydes enabled the use of 4-pyridinecarbonitrile in the reaction, affording a deoxygenative heteroarylation process (**19f**–**g**). We recognized that the diarylbutylpiperidine motif constitutes a core structure in a range of therapeutic agents targeted toward CNS modulation. Penfluridol, for example, is a highly potent oral antipsychotic agent used in the treatment of schizophrenia, which has gained increased interest owed to its recently discovered antiproliferative properties.^36,37^ Haloperidol, an antipsychotic agent displaying an aryl-substituted ketone, could be directly converted into a close analogue of Penfluridol (**19h**) in excellent yield, providing a streamlined means to access novel pharmaceutical candidates in re-emerging areas of medicinal chemistry.^38^

**Figure 5:**
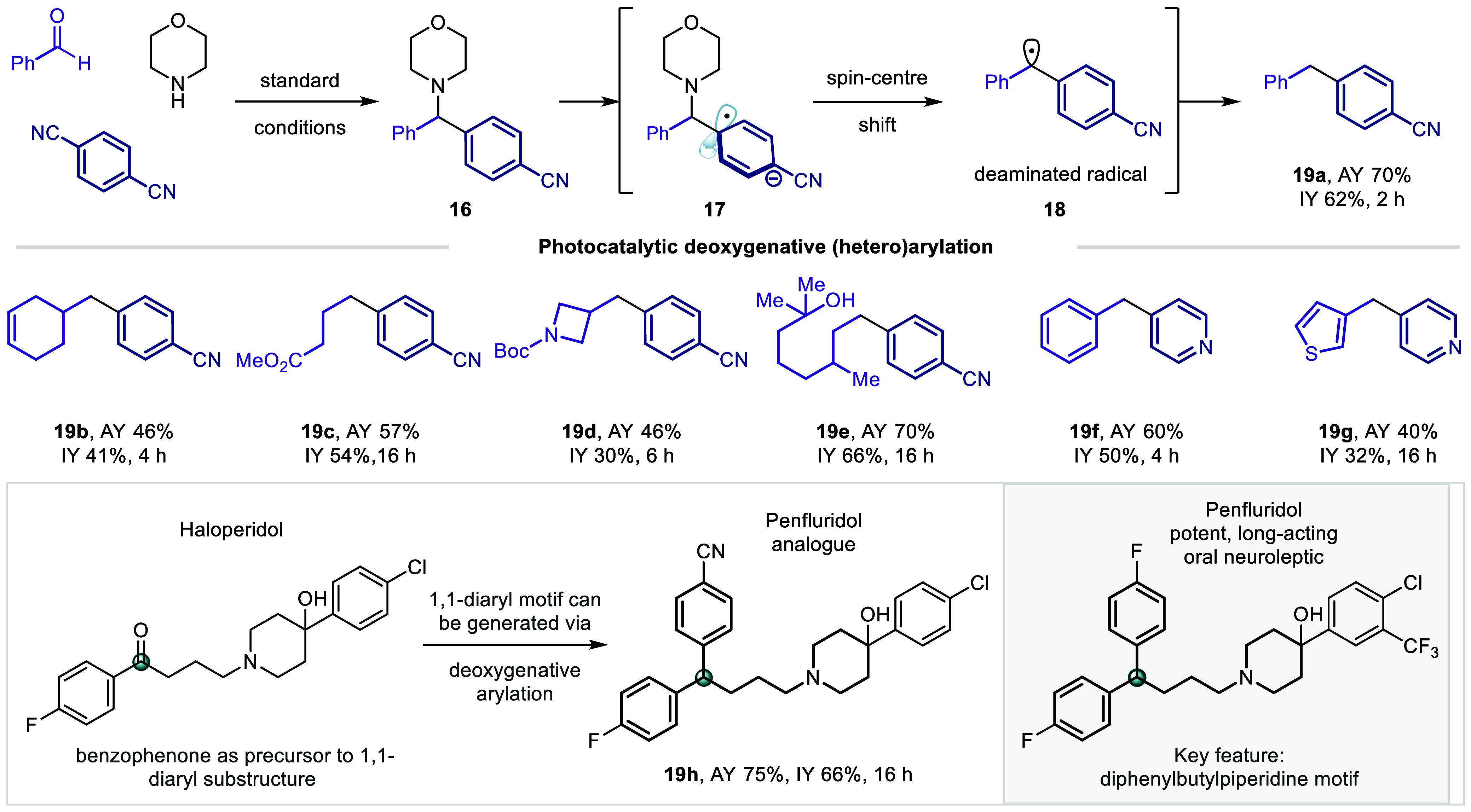
Scope of the deoxygenative (hetero)arylation reaction.

To conclude, we have developed a modular approach for α-amino-radical formation via a photocatalytic radical chain process, which takes place between an in situ-generated iminium ion and an oxidatively generated silyl-radical. This platform is showcased through the arylation of all-alkyl α-amino-radicals with persistent cyanoarene-derived radical anions, furnishing a variety of alkyl-substituted benzylamines. In addition, a photocatalytic deoxygenative arylation reaction is disclosed. This process can generate a range of alkylbenzenes, including those possessing the diarylbutylpiperidine motif, with broad functional group tolerance. Further exploration of these reaction modes will be reported in due course.

## Supporting Information Available

Experimental procedures and compound characterization (PDF)

## Abbreviations

AAR: α-amino-radical
1,4-DCB: 1,4-dicyanobenzene
2-NapSH: 2-naphthalenethiol
DMA: *N*,*N*-dimethylacetamide
TMG: 1,1,3,3-tetramethylguanidine
PMP: *para*-methoxyphenyl
