# Supplementary material for: Silyl Radicals as Single-Electron Reductants: α-Aminoalkyl Radical Formation via a Photocatalytic Oxidatively Initiated Radical Chain Process
Source: J Am Chem Soc. 2024 Sep 16;146(38):25894–901. doi: 10.1021/jacs.4c08230 (PMC11440502; doi:10.1021/jacs.4c08230)

Silyl Radicals as Single-Electron Reductants: α-Aminoalkyl Radical Formation via a Photocatalytic Oxidatively-Initiated Radical Chain Process

Harry C. Waller and Matthew J. Gaunt

Yusuf Hamied Department of Chemistry, University of Cambridge, Cambridge, United Kingdom, CB21EW

Table of Contents

1. General Experimental Considerations 2
2. Reagent synthesis 3
3. Hantzsch Ester Conditions 4
4. Control Experiments and Reaction Monitoring 5
5. Mechanistic Considerations 8
   A. Double single-electron reduction mechanism 8
   B. Role of the silane – addition of a silyl radical scavenger 9
   C. Role of the silane – TEMPO addition to shut down silyl radical formation 11
   D. Potential charge-transfer complex formation 13
   E. Role of the urea 15
6. Stern-Volmer Quenching Experiments 17
7. Cyclic Voltammetry 20
8. General Procedures 23
9. Compound Characterisation 24
10. NMR Spectra 45

1. General Experimental Considerations

All reactions were conducted under inert atmosphere (N_2) and stirred using Teflon-coated magnetic stir bars unless otherwise stated. Anhydrous solvents were obtained from Acros Organics (AcroSeal® extra dry) or from solvent stills (THF was distilled from Na/benzophenone; dichloromethane, acetonitrile, and toluene from calcium hydride). Unless otherwise specified, reagents were purchased from commercial suppliers and used without further purification. Photoredox reactions were performed in 4 mL glass vials and sealed with screw caps containing holes which were equipped with PFTE-lined septa. The reaction vessels were irradiated using 40 W Kessil A160WE LED lamps with intensity dials set to maximum and colour dials set to maximum blue (λ = 371 – 535 nm). Cooling was provided by a fan placed over the top of the reaction vessels. Assay yields by ^{1}H NMR analysis of the crude reaction mixture were determined against 1,1,2,2-tetrachloroethane as an internal standard.

Stern-Volmer quenching experiments were performed using an Agilent Cary Eclipse Fluorescence Spectrophotometer using a quartz cell equipped with septa-lined screw cap under N2. Cyclic voltammograms (CVs) were recorded using a Metrohm Autolab PGSTAT204 Potentiostat Galvanostat. Analytical thin layer chromatography (TLC) was performed on Merck Kieselgel 60 F254 0.20 mm glass-backed plates pre-coated with silica (0.2 mm) with vizualisation by UV absorbance (λ_{max} = 254 nm) or staining with aqueous $KMnO_4$ stain. Silica-gel flash column chromatography was performed with Teledyne CombiFlash NextGen 300 using a RediSep Rf GoldTM column with the specified solvent systems.

Nuclear Magnetic Resonance (NMR) spectra were recorded in deuterated chloroform using Brücker Avance III NMR spectrometers at the indicated field strength and at 298 K. Chemical shifts (δ) are reported in parts per million (ppm) relative to the residual solvent peak ($CHCl_3$ at 7.26 and 77.16 ppm for ^{1}H and ^{13}C NMR, respectively). ^{1}H NMR data are presented as follows: chemical shift (multiplicity [s = singlet; d = doublet; t = triplet; q = quartet; m = multiplet], coupling constant (*J*), integration, assignment). Coupling constants (*J*) are reported in Hertz (Hz). ^{13}C NMR spectra were recorded with complete proton decoupling. ^{13}C-DEPT 135 and additional 2D NMR experiments (COSY, HSQC and HMBC) were used to support assignments. All spectra were analysed and processed using MestReNova v.14.2.1-27684 software. Infrared spectra (IR) were acquired using a Thermo Scientific Nicolet Summit Pro FTIR spectrometer equipped with an Everest ATR attachment. Absorptions (υ_{max}) are reported in wavenumbers (cm^{-1}). Samples were applied as a solution in CH_2Cl_2 and the solvent evaporated to leave a thin film. High-resolution mass spectrometry (HRMS) was performed on a Shimadzu LCMS-9030 quadrupole time-of-flight mass spectrometer. Compound names are those generated by PerkinElmer ChemDraw Professional v. 20.1.1.125, according to IUPAC nomenclature.

2. Reagent synthesis

Diethyl (4-cyanophenyl)phosphonate (11z)

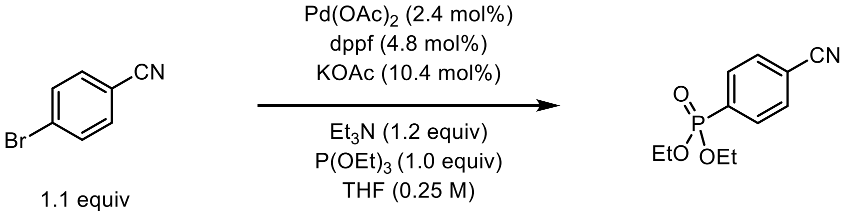

Pd(OAc)2 (14 mg, 0.06 mmol, 2.4 mol%), dppf (66 mg, 0.12 mmol, 4.8 mol%) and KOAc (26 mg, 0.26 mmol, 10.4 mol%) were added to an oven-dried 25 mL round-bottomed flask. The vessel was sealed and evacuate-backfilled with N_2 (3×). THF was introduced *via* a septum (10 mL, 0.25 M), followed by triethylamine (3.0 mmol, 416 uL, 1.2 equiv), and the mixture was stirred and heated at 68 °C. After 15 min, triethyl phosphite (0.32 mL, 2.50 mmol, 1.0 equiv) and 4-bromobenzenecarbonitrile (510 mg, 2.80 mmol, 1.1 equiv) were dissolved in a small amount of THF and were added to the reaction mixture in one portion. After heating at 68 °C for 16 h, the solvent was removed *in vacuo*. The crude residue was purified by flash column chromatography on silica gel (0–50% PET:EtOAc) to afford the title compound as a colourless oil (359 mg, 1.5 mmol, 60%). **^{1}H NMR** (400 MHz, $CDCl_3$): δ 7.96 – 7.86 (m, 2H), 7.79 – 7.71 (m, 2H), 4.24 – 4.03 (m, 4H), 1.32 (td, *J* = 7.0, 0.6 Hz, 6H). **^{13}C NMR** (101 MHz, $CDCl_3$) δ 134.1 (d, *J* = 187.7 Hz), 132.4 (d, *J* = 9.9 Hz), 132.1 (d, *J* = 15.1 Hz), 118.0 (d, *J* = 1.7 Hz), 116.1 (d, *J* = 3.5 Hz), 62.8 (d, *J* = 5.6 Hz), 16.5 (d, *J* = 6.4 Hz). Data consistent with literature:

Roy, V. J.; Roy, S. R. Light-Induced Activation of C−X Bond via Carbonate-Assisted Anion−π Interactions: Applications to C−P and C−B Bond Formation. *Org. Lett.* **2023**, *25*, 923−927.

3. Hantzsch Ester Conditions

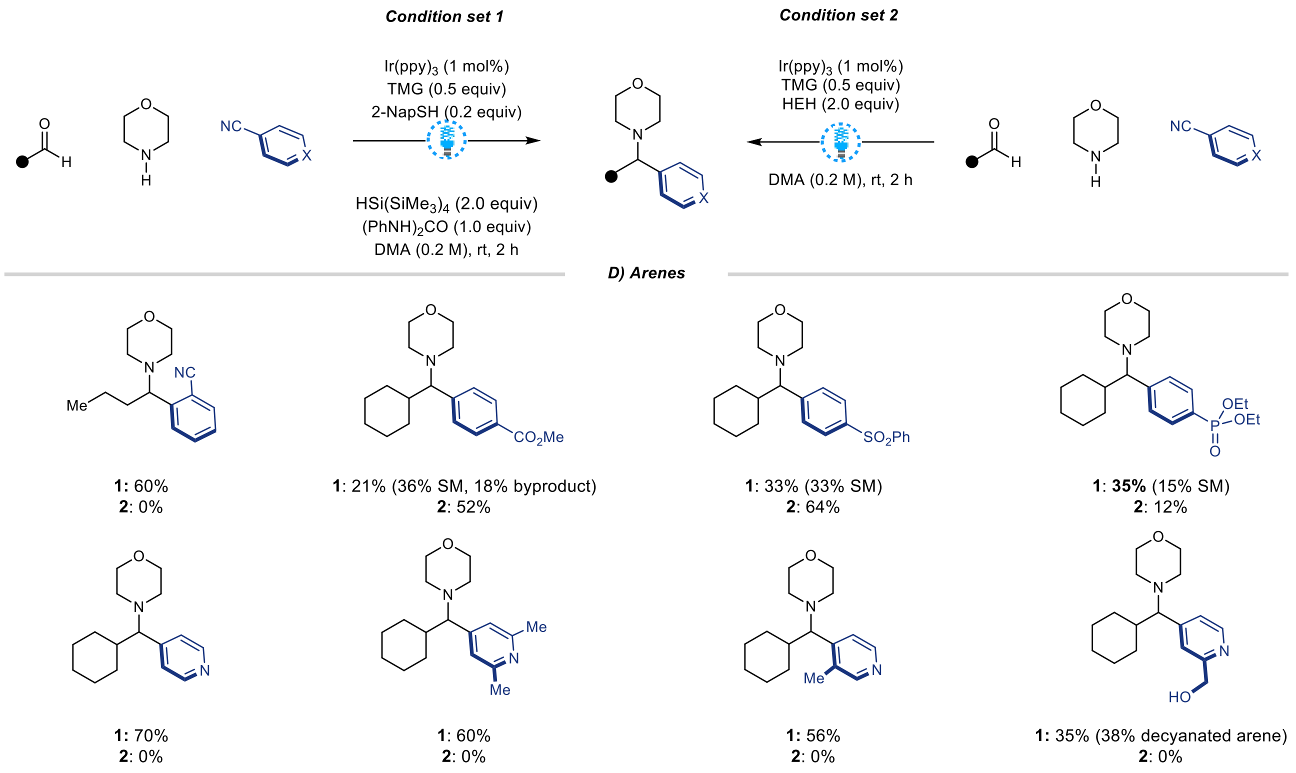

Figure S1. Comparison to reaction conditions using Hantzsch Ester.

In the early stages of reaction development, evaluation of reaction conditions using Hantzsch Ester was shown to be capable of enabling this process *via* a precedented double single-electron reduction mechanism of both the cyanoarene and iminium ion. It was noticed, however, that this approach was limited to the use of two arenes (the ester and sulfone), with all other examples displaying no reactivity under these alternative conditions. This divergence in the number of amenable coupling partners suggests a switch in mechanism between the two sets of conditions, consistent with our other mechanistic investigations into the proposed radical chain process.

4. Control Experiments and Reaction Monitoring

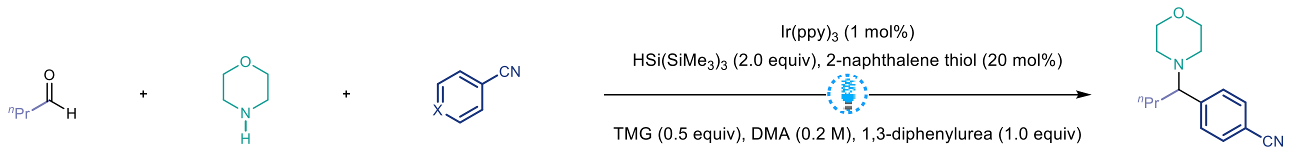

Table S1. Control experiments

Entry	Deviation from standard conditions	Yield
1	no photocatalyst	18%
2	no HSi(SiMe$_3$)$_3$	0%
3	No 2-NapSH	0%
4	No TMG	0%
5	No 1,3-diphenylurea	35%

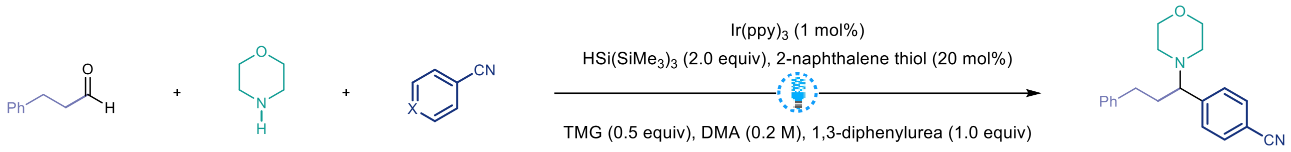

Table S2. Effect of light intensity

Entry	Light intensity	Yield	1,4-DCB recovery
1	25%	39%	45%
2	50%	56%	20%
3	75%	72%	0%
4	100%	78%	0%

30 min - large DCB recovery

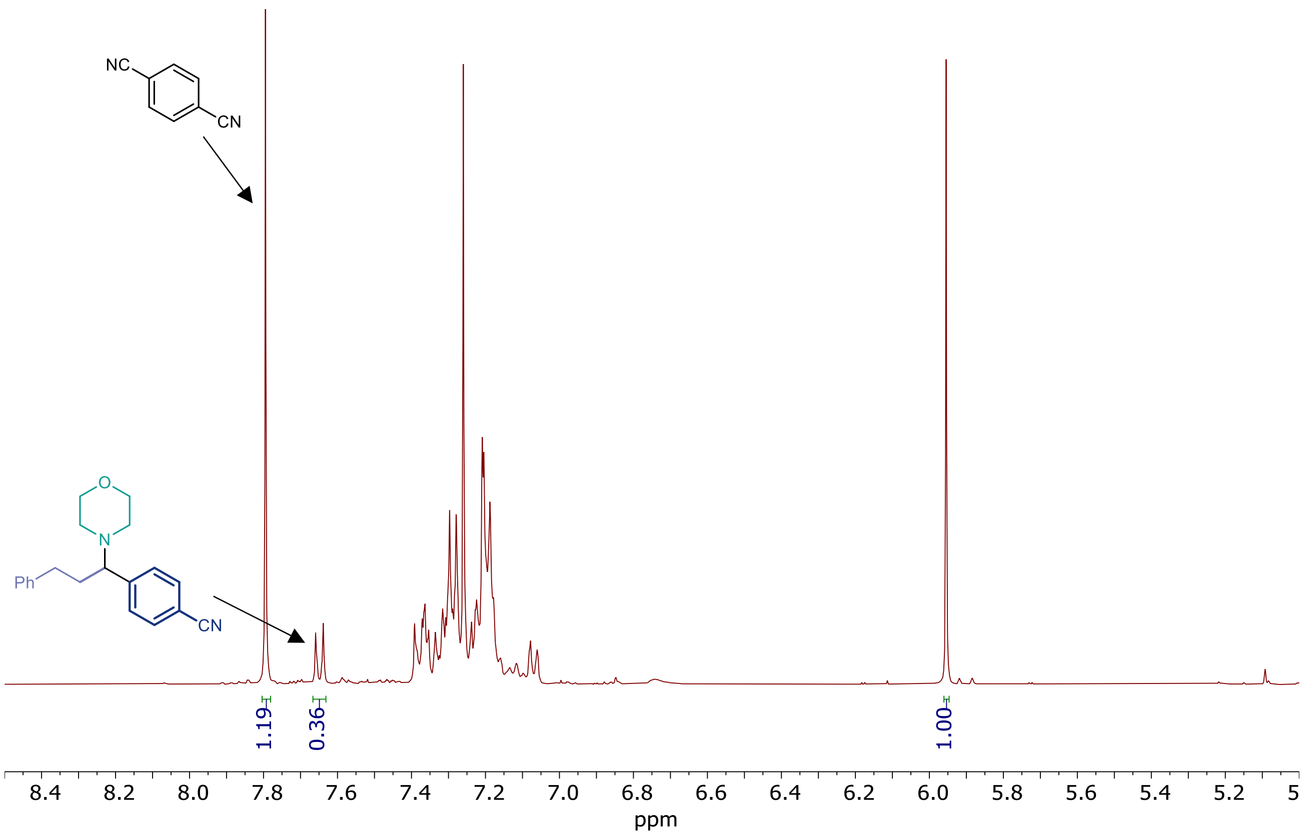

1 h - reaction complete

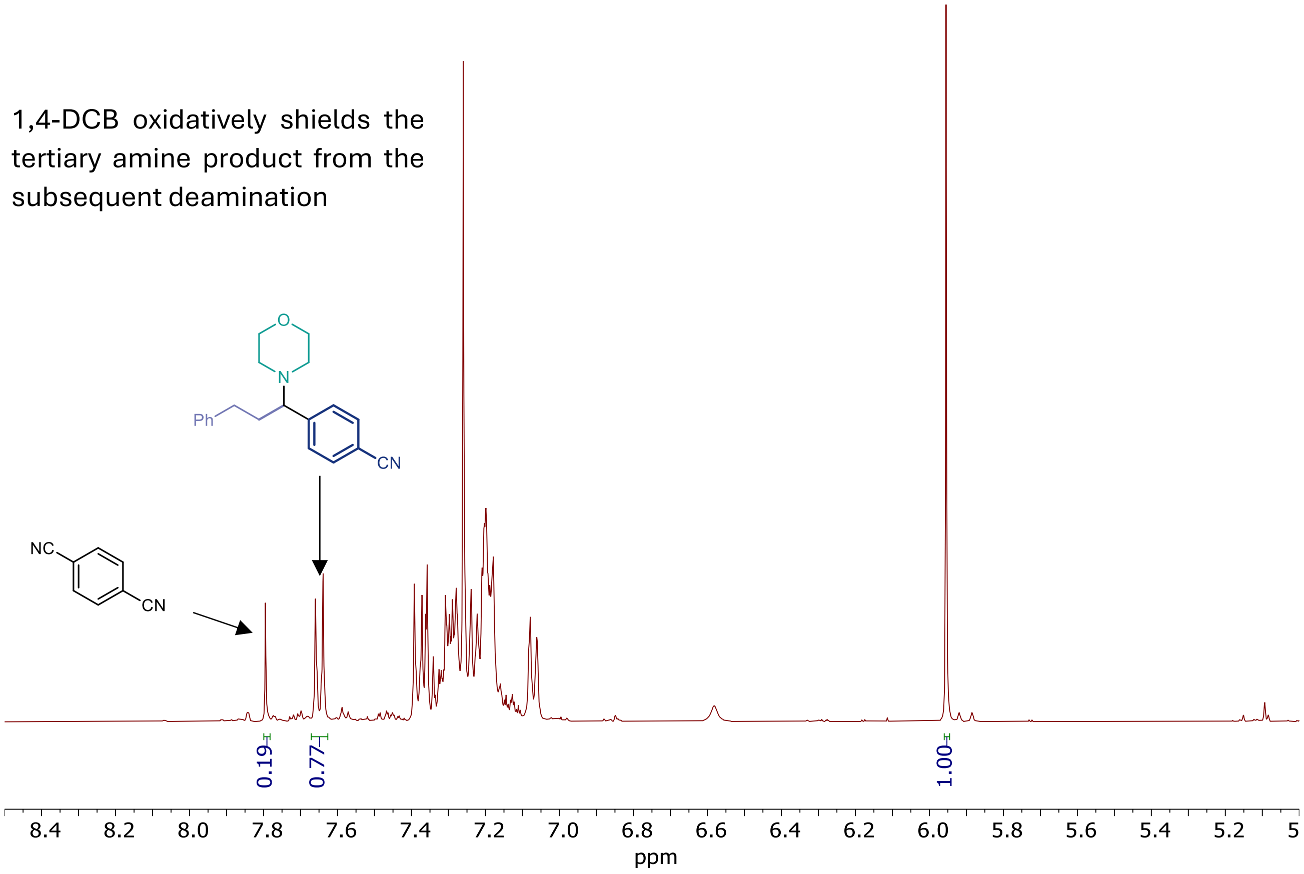

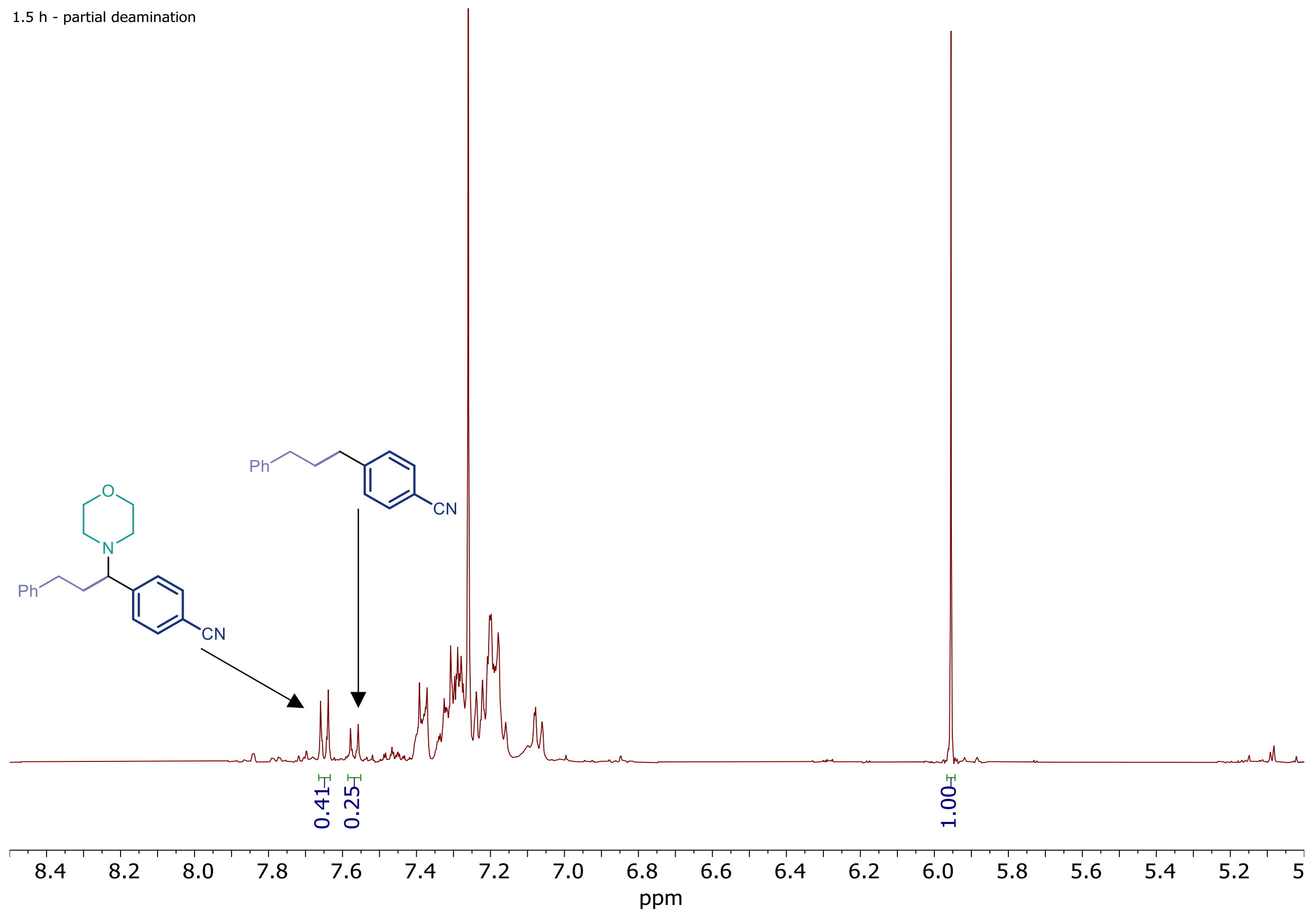

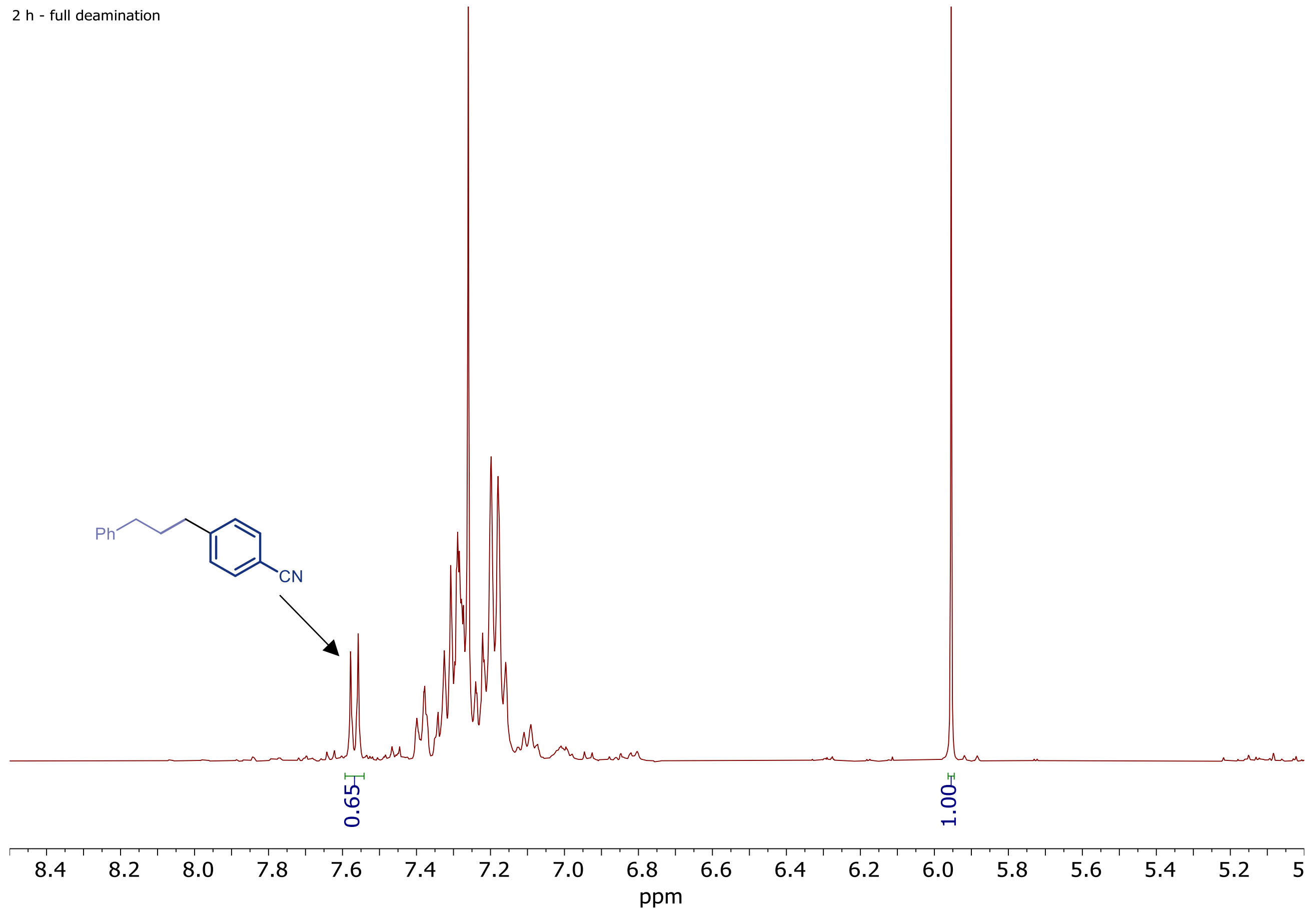

Figure S2. Reaction monitoring

5. Mechanistic Considerations

A. Double single-electron reduction mechanism

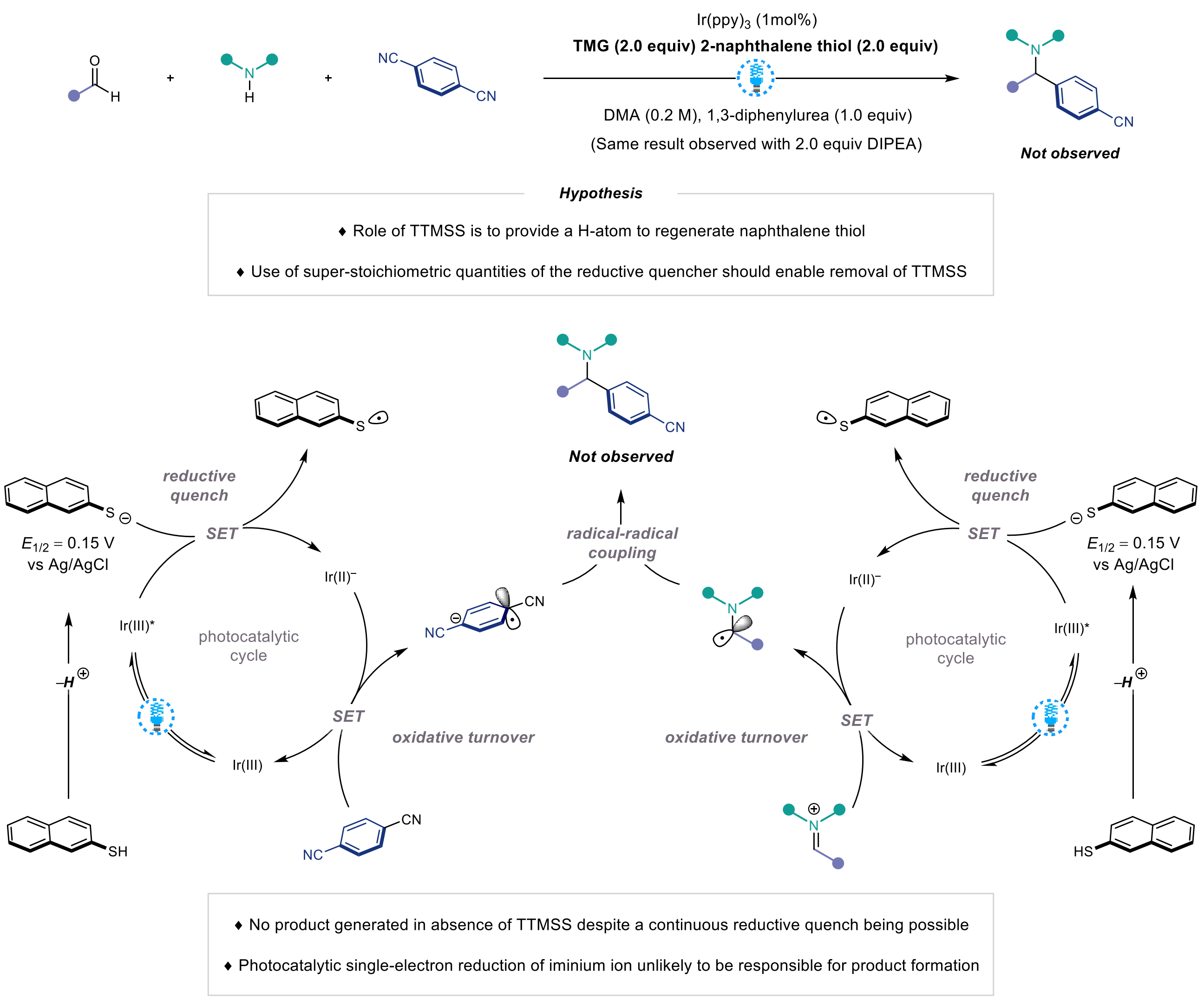

Figure S3. Double single-electron reduction mechanism mediated by stoichiometric reductant.

It was reasoned that should Ir(II)$^{-}$ be responsible for the formation of the required α-amino radical *via* a precedented iminium ion single-electron reduction mechansim, then the role of the silane would be only to enable the regeneration of the thiolate anion which is required for the formation of this reduced state of the photocatalyst. As a result, the use of stoichiometric thiolate (such that the thiolate anion would no longer need to be catalytic to lead to full conversion) should enable product formation with the silane omitted from the reaction conditions (**Figure S3**). However, it was found that omission of H−Si(SiMe$_3$)$_3$ from the reaction conditions whilst using 2.0 equiv of 2-NapSH and TMG (or 2.0 equiv of diisopropylethylamine) afforded no product formation. Consequently, it is reasoned that a mechanism such as the one shown in Figure **S3** is not operative in this system under these conditions.

B. Role of the silane – addition of a silyl radical scavenger

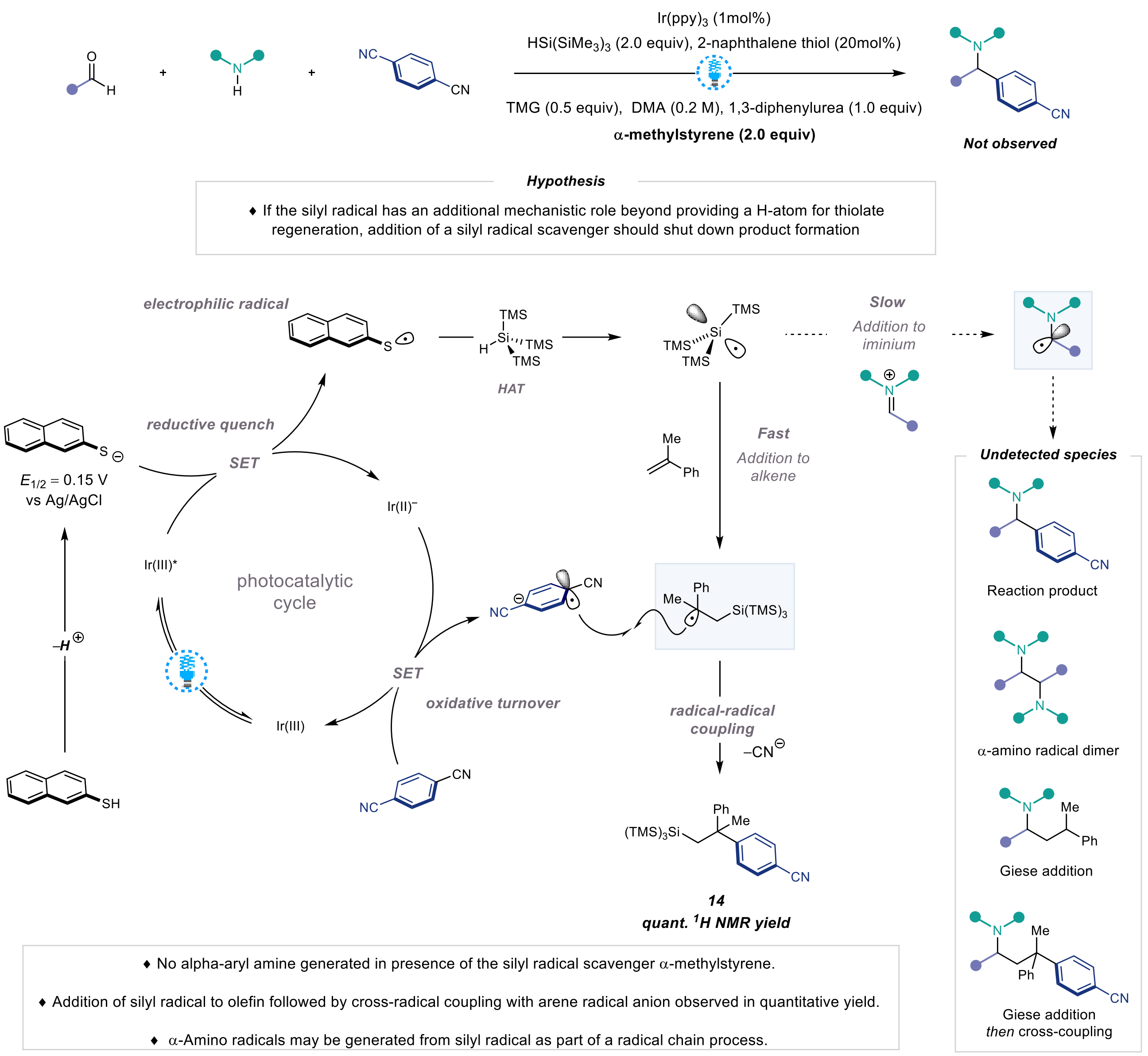

Figure S4. α-Methylstyrene as a silyl radical scavenger.

Should the silyl radical have a mechanistic role extending beyond regeneration of the thiolate anion, then addition of a species which efficiently removes this intermediate should prevent formation of the desired benzylamine product. Addition of α-methylstyrene into an otherwise standard reaction was observed to completely shut down product formation. Instead, silyl radical addition to the olefin, followed by cross-radical coupling with the arene radical anion, was observed in quantitative yield. It is reasoned that the addition of this reagent initiates a competition between silyl radical addition into the iminium ion and addition into the styrene, using cross-radical coupling to 1,4-DCB radical anion as a read-out for the process. Since addition into the styrene is much faster than addition into the iminium ion, the α-amino radical is not formed under these conditions and the tertiary benzylic radical generated undergoes efficient cross-radical coupling with the arene radical anion.

4-(1-(1,1,1,3,3,3-Hexamethyl-2-(trimethylsilyl)trisilan-2-yl)-2-phenylpropan-2-yl)benzonitrile (14)

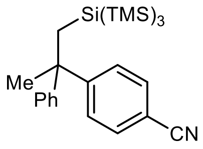

The title compound was synthesised according to General Procedure A, using cyclohexanecarboxaldehyde (distilled prior to use - 73 uL, 0.6 mmol, 3.0 equiv), morpholine (52 µL, 0.6 mmol, 3.0 equiv) and 1,4-DCB (26 mg, 0.2 mmol, 1.0 equiv). α-Methylstyrene (52 µL, 0.4 mmol, 2.0 equiv) was added after TTMSS. Irradiation time 2 h. Purification by flash column chromatography on silica gel (0–20% PET:EtOAc) afforded the title compound as a colourless oil (87 mg, 0.186 mmol, 93% – quantitative yield by ^{1}H NMR assay). **^{1}H NMR** (500 MHz, $CDCl_3$) δ 7.55 – 7.52 (m, 2H), 7.31 – 7.27 (m, 4H), 7.22 – 7.18 (m, 3H), 1.93 (d, *J* = 14.6 Hz, 1H), 1.78 (m, 4H), 0.09 (s, 27H). **^{13}C NMR** (126 MHz, $CDCl_3$) δ 157.4, 149.1, 132.2, 128.7, 127.7, 127.0, 126.5, 119.2, 109.6, 45.9, 29.2, 25.0, 1.6. **IR** (film) ν_{max}/cm^{-1} 2947, 2893, 2228 (C≡N), 1604, 1499, 1444, 1397, 1243, 1057, 825, 745, 699, 684, 622. **HRMS** (ESI+) calculated for $C_{25}H_{42}NSi_4$ $[M+H]^+$ 468.23888, found 468.23879.

C. Role of the silane – TEMPO addition to shut down silyl radical formation

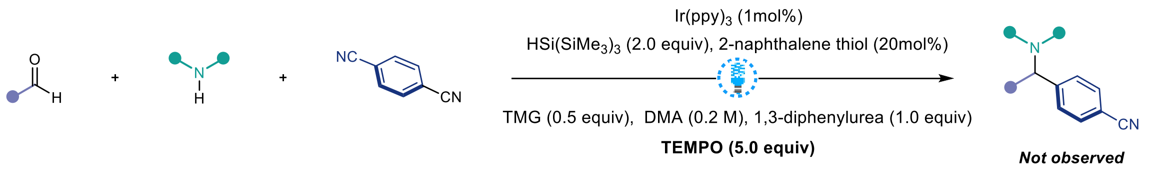

Hypothesis

- ♦ Inclusion of TEMPO should trap transient radical species, shutting down reactivity.
- ♦ Trapping of the first radical in the sequence prevents formation of those which occur downstream.

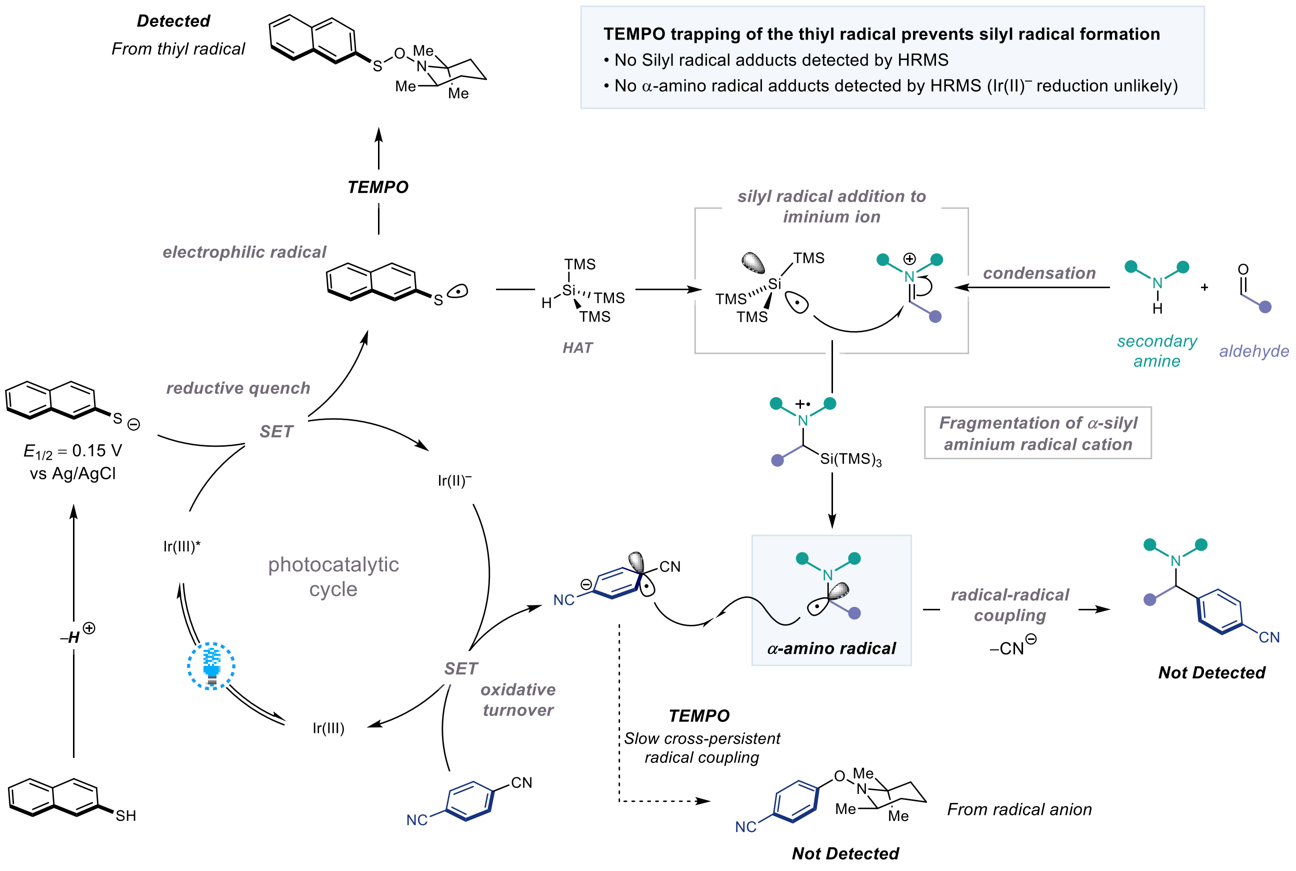

Figure S5. TEMPO trapping experiment to shut down radical chain process.

If the α-amino radical is generated from the silyl radical as part of a radical chain process (oxidative thiyl radical formation, then HAT to generate silyl radical, followed by radical exchange to generate the α-amino radical), then removal of the thiyl radical through TEMPO trapping should inhibit the formation of the two subsequent radicals in the sequence. Inclusion of 5.0 equiv of TEMPO resulted in only the thiyl radical, the first radical to be produced in the mechanism, being detected by HRMS; neither the silyl radical nor α-amino radical adducts could be detected. This supports that the silyl radical and α-amino radical are both generated as part of a radical chain process which begins with the formation of the thiyl radical. What's more, a lack of observation of the α-amino radical adduct indicates an absence of this intermediate under these conditions, supporting that Ir(II)⁻ reduction of the iminium ion is unlikely to be taking place. The 1,4-DCB-TEMPO adduct is not detected owed to the slow cross-radical coupling of two persistent radicals.

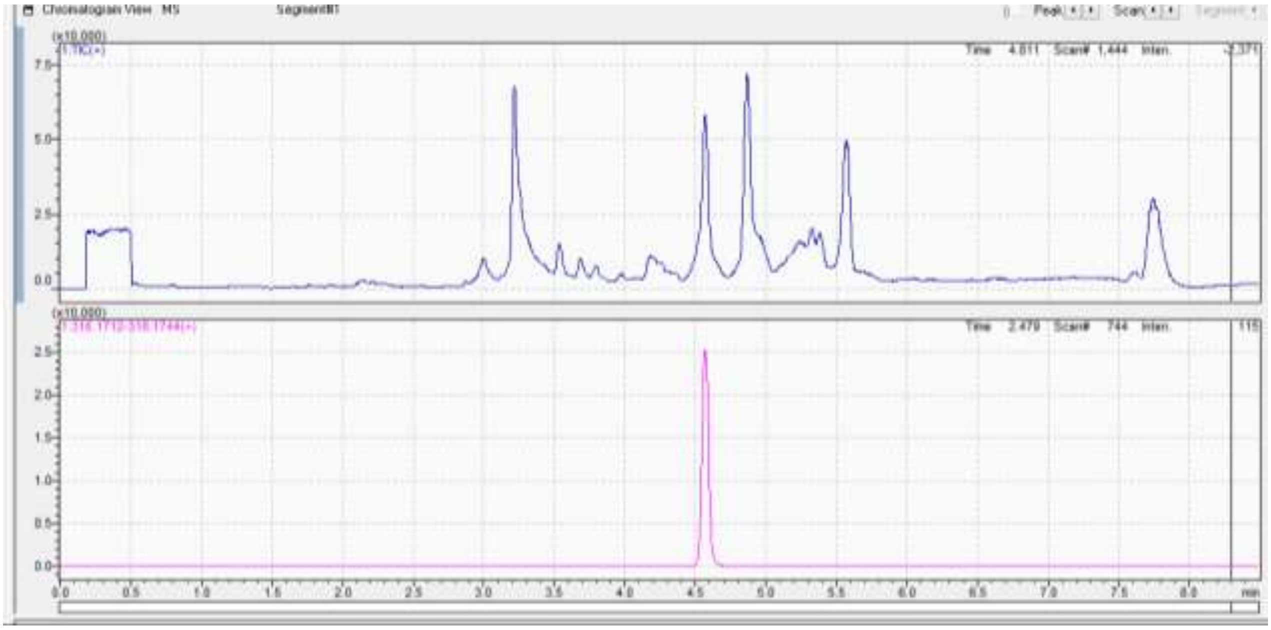

Figure S6. Thiyl radical-TEMPO adduct HRMS chromatogram.

#	Score	Pred. (M)	Pred. m/z	Meas. m/z	Diff. (mDa)	Formulae (M)	Ion	Diff. (ppm)	Iso Score	DBE
2	97.56	315.16569	316.17296	316.17284	-0.12	C19 H25 N O S	[M+H]+	-0.380	97.45	8.0

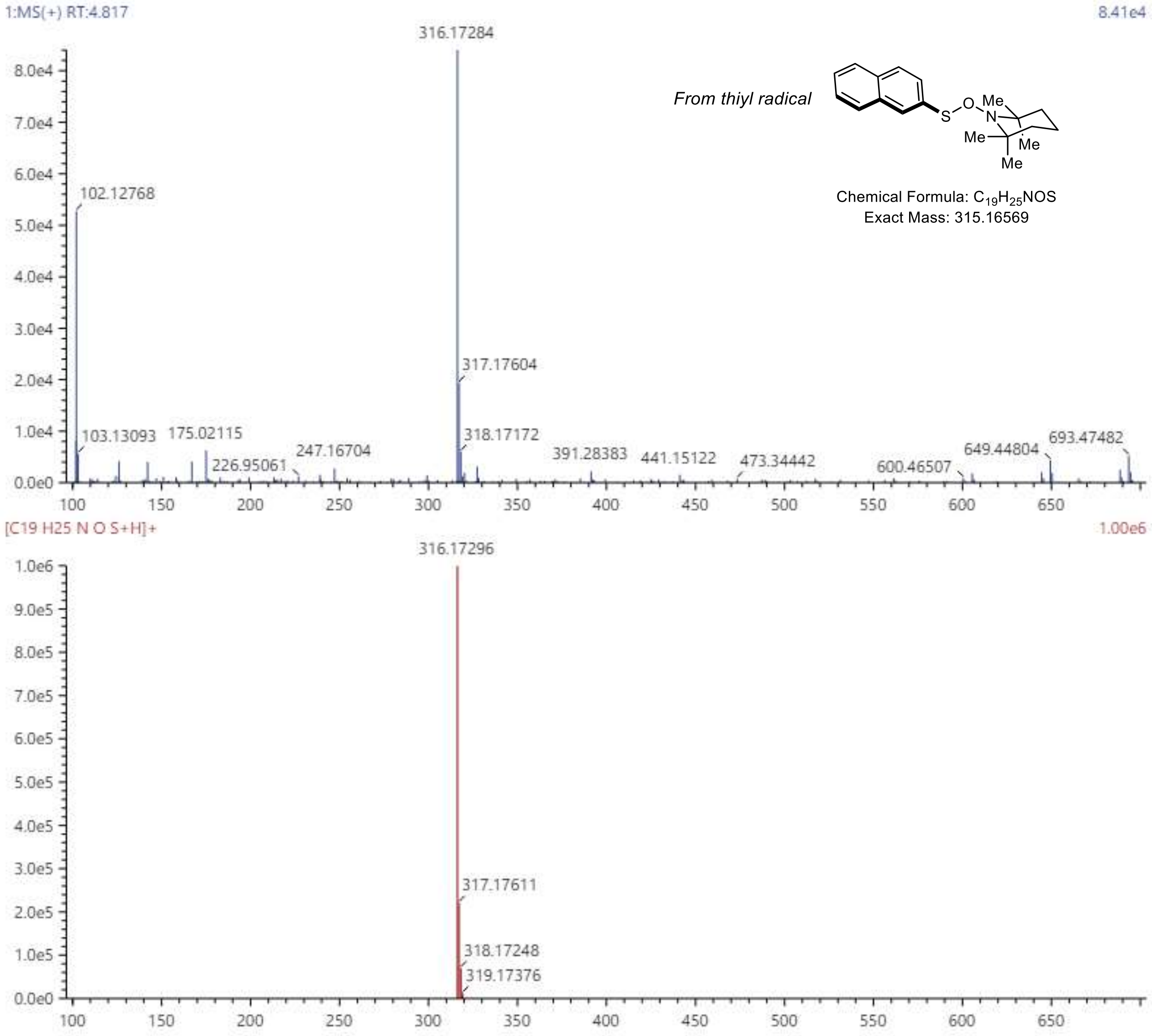

Figure S7. Thiyl radical-TEMPO adduct HRMS report.

D. Potential charge-transfer complex formation

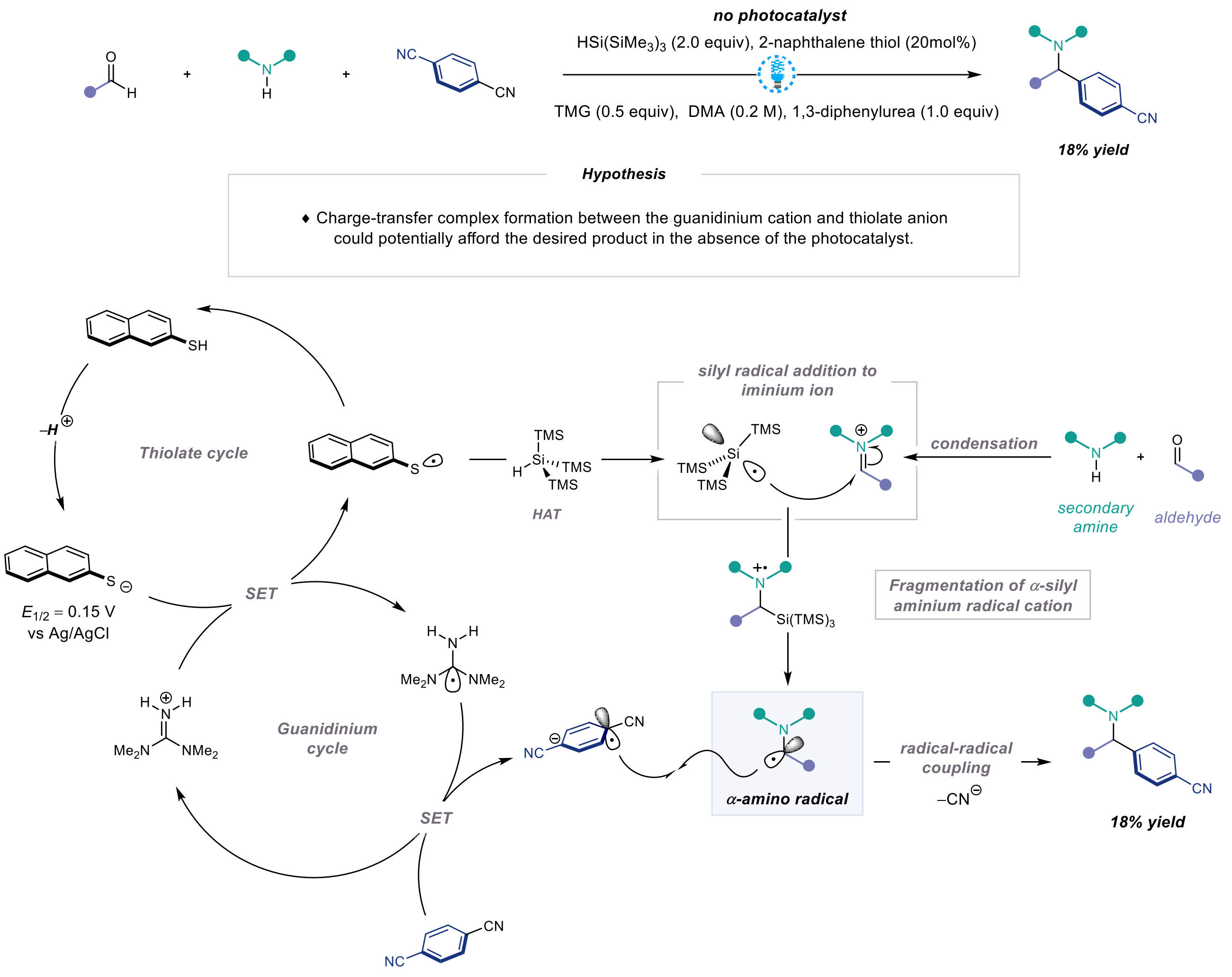

Figure S8. Charge-transfer-mediated multicomponent arylation reaction

Control experiments revealed that product could still be afforded in the absence of the photocatalyst. It is believed that a charge-transfer complex can form between the guanidinium cation and thiolate anion. Visible-light-mediated single-electron transfer between these two components would afford a thiyl radical and an electron-rich radical derived from the guanidinium cation. The former of these species can undergo HAT with $(SiMe_3)_3SiH$ to afford the required silyl radical (regenerating 2-NapSH), which in turn can generate the requisite α-amino radical. The latter can act as a single-electron reductant for 1,4-DCB (regenerating the guanidinium cation) to generate the requisite persistent radical anion; cross radical coupling between this species and the α-amino radical would then generate the desired product.

In order to investigate the feasibility of such a charge-transfer complex, UV-Vis absorption experiments were performed on the 2-NapSH+TMG salt. An absorption band at λ_{max} = 414 nm could be observed, indicating the ability of the salt to absorb light under blue-light irradiation:

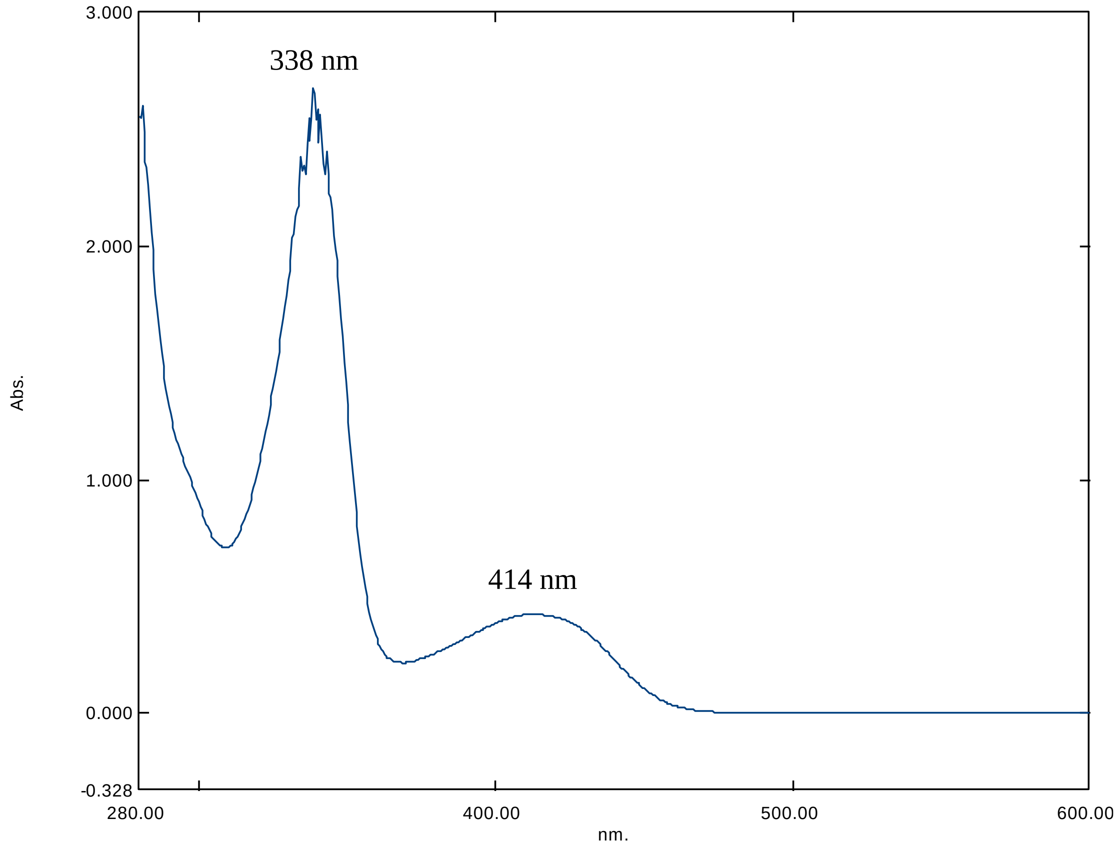

Figure S9. UV-Vis absorption spectrum of 2-NapSH + TMG (1:2.5)

Furthermore, cyclic voltammetry of this mixture revealed both its donor and acceptor properties, with an oxidation peak at ($E_{1/2}$ = −1.55 V vs Ag/AgCl) – which is believed to correspond to oxidation of the thiolate anion – and an irreversible reduction peak at ($E_{1/2}$ = −1.55 V vs Ag/AgCl), which is believed to correspond to reduction of the guanidinium cation – a process potentially rendered irreversible by the evolution of H_2 at the cathode (Figure **3**).

E. Role of the urea

Examination of the cyclic voltammogram of the 2-NapSH + TMG mixture revealed a reduction process which occurs at $E_{1/2} = -1.55$ V vs Ag/AgCl – the same potential as the reduction of 1,4-DCB. This process is believed to correspond to reduction of the guanidinium cation (see Bhattacharya, M.; Sebghati, S.; Vercella, Y. M.; Saouma, C. T. Electrochemical Reduction of Carbamates and Carbamic Acids: Implications for Combined Carbon Capture and Electrochemical CO_2 Recycling, *Journal of The Electrochemical Society*, **2020**, *167*, 086507); whilst the resulting radical itself may be capable of reducing 1,4-DCB (see **D. potential charge-transfer complex formation**), the stability of the tri-heteroatom-substituted radical likely renders this process slower than direct photocatalytic reduction. Furthermore, the irreversibility of the reduction event observed in the CV indicates a subsequent chemical reaction ensues following the reduction (see **7. Cyclic Voltammetry**), a process which will likely compete with the transfer of the electron to 1,4-DCB. Consequently, the presence of this competing reduction process likely results in low product yield (35% in the absence of the 1,3-diphenylurea additive). Addition of 1,3-diphenylurea into the 2-NapSH + TMG mixture revealed suppression of this reduction process, resulting in improved product yield. The addition of the urea additive into this mixture is also concomitant with an increase in oxidation potential of the thiolate anion, consistent with hydrogen bonding to the thiolate (Figure **3**). To further confirm the presence of the hydrogen bond to the thiolate anion, a ^{1}H NMR titration study in DMF-d_7 was conducted:

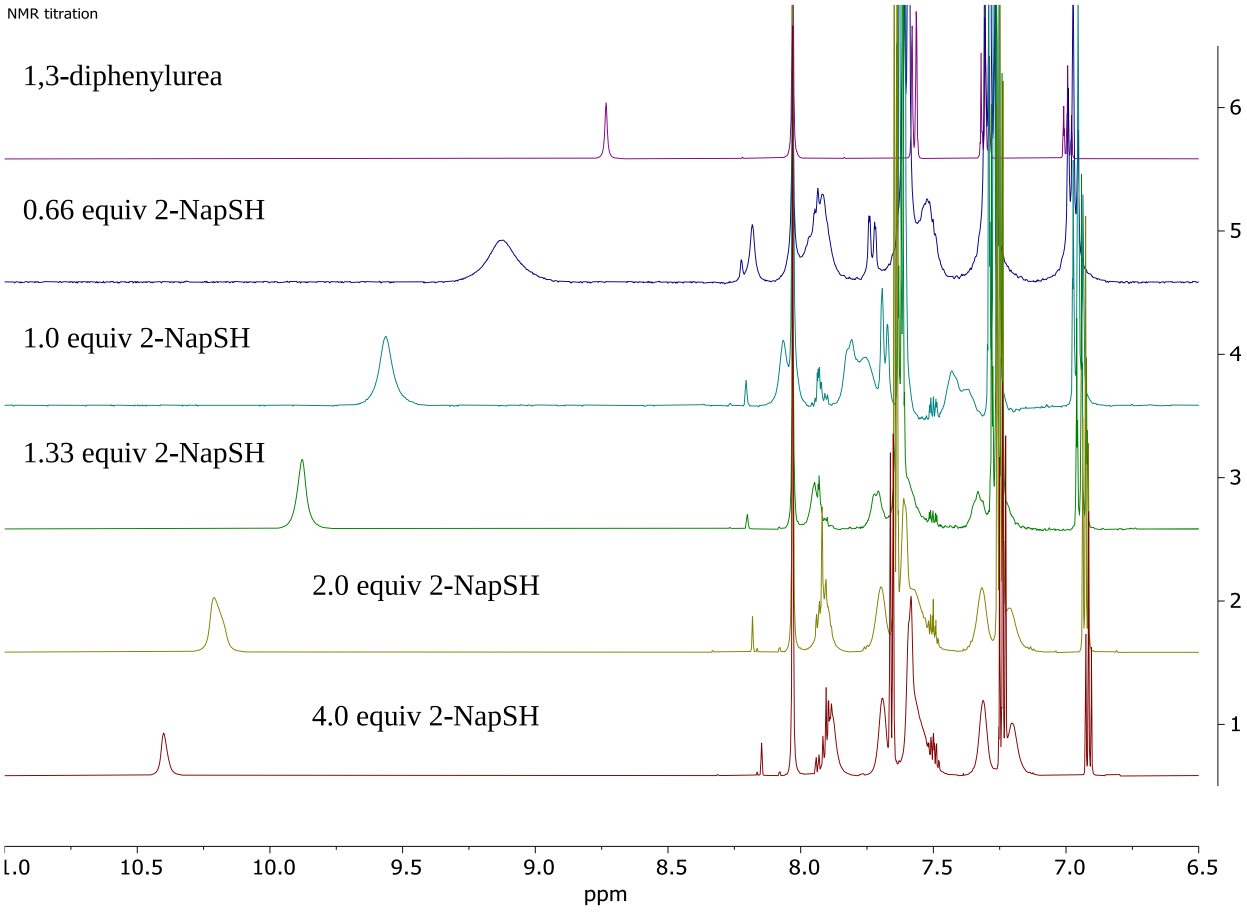

Figure S10. ^{1}H NMR titration study

The deshielding of the N-H protons of the urea with increasing 2-NapSH anion concentration is supportive of the proposed hydrogen bonding interaction. To evaluate the impact of this on the charge-transfer complex shown in **Figure S9**, a UV-Vis absorption spectrum was recorded of the 2-NapSH + TMG mixture in the presence of 1,3-diphenylurea:

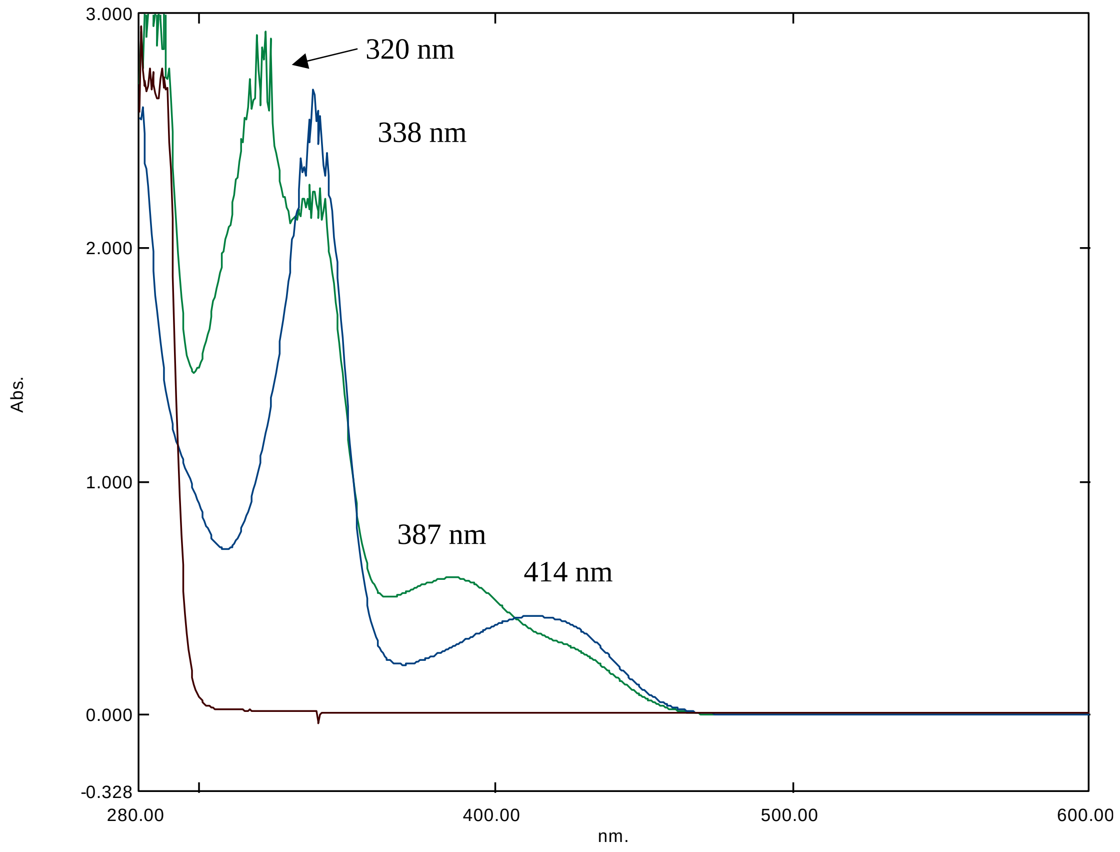

Figure S11. Overlayed UV-Vis absorption spectra of 2-NapSH + TMG (blue), 2-NapSH + TMG + 1,3-diphenylurea (green), and 1,3-diphenylurea (black).

The resulting hypsochromic shift might reflect a higher energy transition within the guanidinium thiolate charge transfer complex, which would be consistent with hydrogen bonding to the thiolate anion with the resulting stabilisation of the donor component attenuating its ability to act as a reductant.

6. Stern-Volmer Quenching Experiments

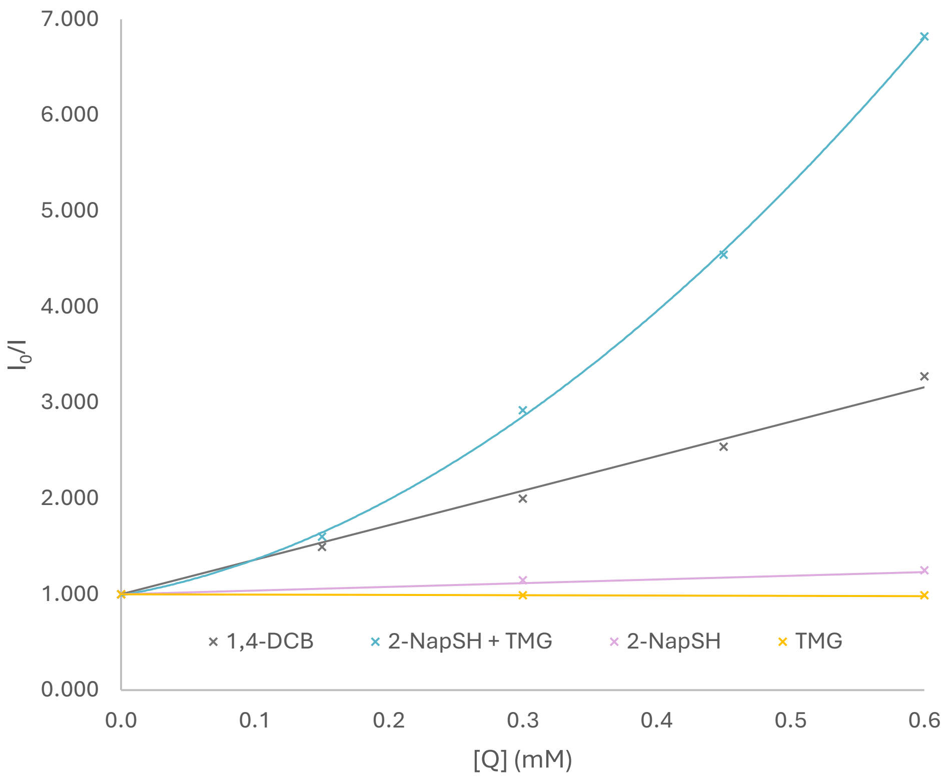

Figure S12. Stern-Volmer quenching experiments.

Stern-Volmer quenching studies were conducted on 2-naphthalene thiol (2-NapSH), TMG, 2-NapSH + TMG (1:2.5), and 1,4-DCB. Standard solutions of these species, their associated combinations, and Ir(ppy)$_3$ were prepared in dry, degassed DMA. Quenching concentrations of mixtures are given relative to the limiting reagent (2-NapSH). Solutions containing 0.025 mM photocatalyst and varying concentration of the quenching mixture were prepared in a quartz cuvette that had been evacuated and backfilled with N_2 (3×); final solutions were purged with N_2 for 3 minutes before irradiation. The samples were irradiated at 377 nm, and an emission spectrum was recorded over a range of 300 – 700 nm; luminescence peak intensity was measured at 517 nm. Each intensity measurement is an average of 6 readings made from the same sample to average out instrument variation.

2-NapSH

Quencher concentration (mM)	Intensity	I_0/I
0.0	600.892	1.000
0.30	524.715	1.145
0.6	481.519	1.248
0.90	470.443	1.277
1.2	400.642	1.500

TMG

Quencher concentration (mM)	Intensity	I_0/I
0.0	600.892	1.000
0.30	606.845	0.990
0.6	607.347	0.989
0.90	632.04	0.951
1.2	619.049	0.971

2-NapSH + TMG (1:2.5 – reaction relevant ratio)

Quencher concentration (mM)	Intensity	I_0/I
0.0	720.418	1.000
0.15	450.153	1.600
0.3	246.735	2.920
0.45	158.651	4.541
0.6	105.653	6.819

1,4-DCB

Quencher concentration (mM)	Intensity	I_0/I
0.0	600.892	1.000
0.15	402.522	1.493
0.3	300.674	1.998
0.45	236.661	2.539
0.6	183.572	3.273

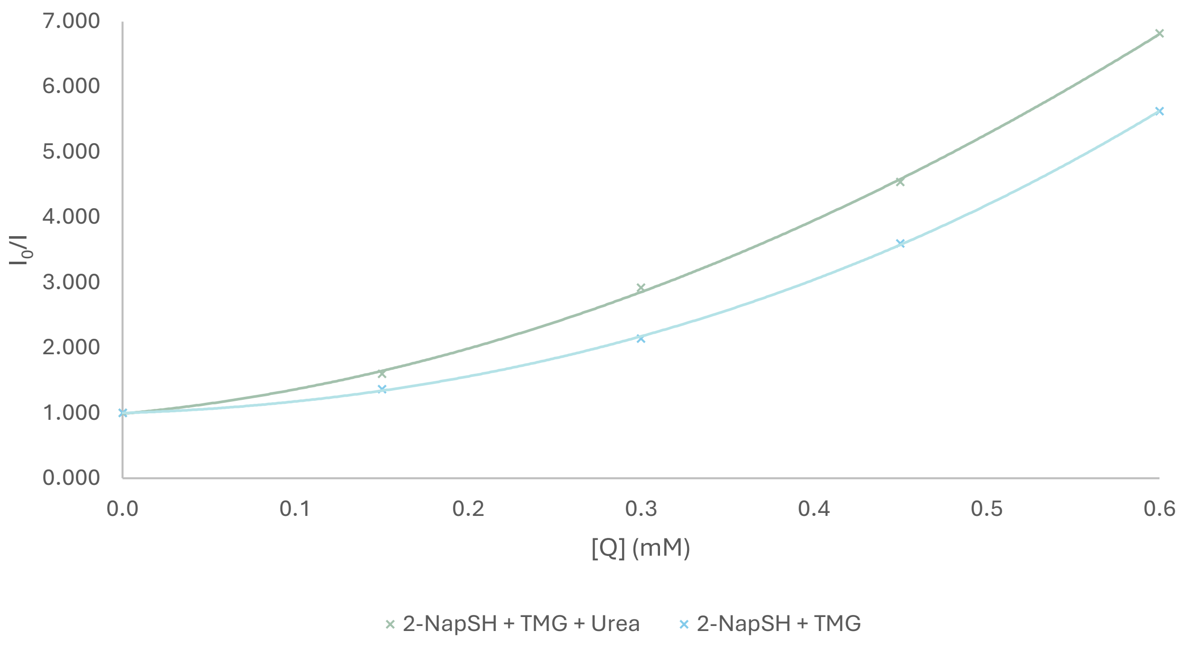

Figure S13. Stern-Volmer quenching experiment into role of the urea.

Procedure as for the previous plots. Quenching concentrations of mixtures are given relative to the limiting reagent and active quenching species (2-NapSH) in both cases.

2-NapSH + TMG + 1,3-diphenylurea (1:2.5:5 – reaction relevant ratio)

Quencher concentration (mM)	Intensity	I_0/I
0.0	900.185	1.000
0.15	660.492	1.364
0.3	420.338	2.143
0.45	250.101	3.600
0.6	160.299	5.625

7. Cyclic Voltammetry

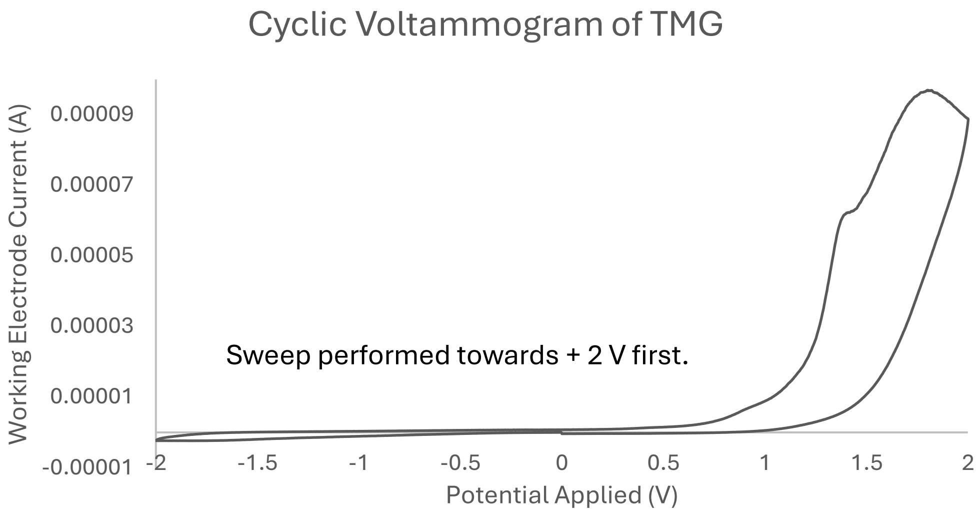

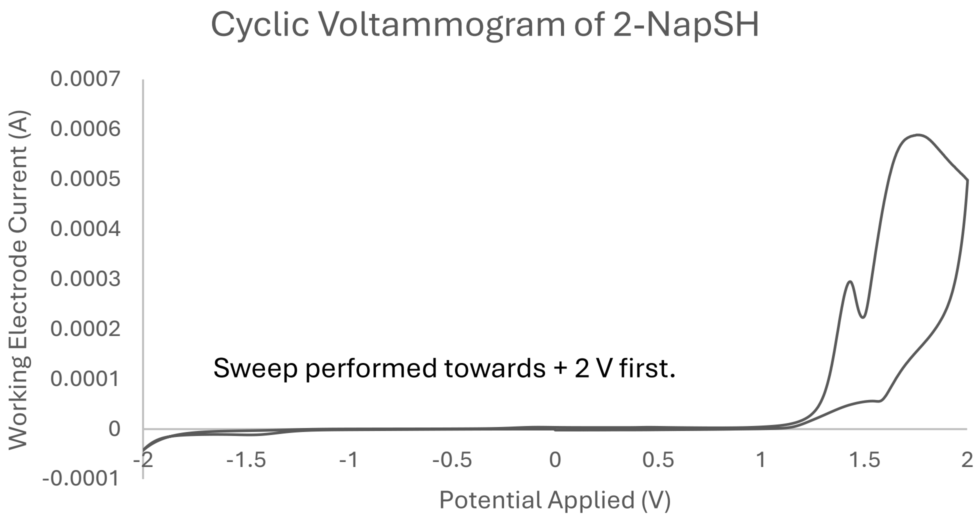

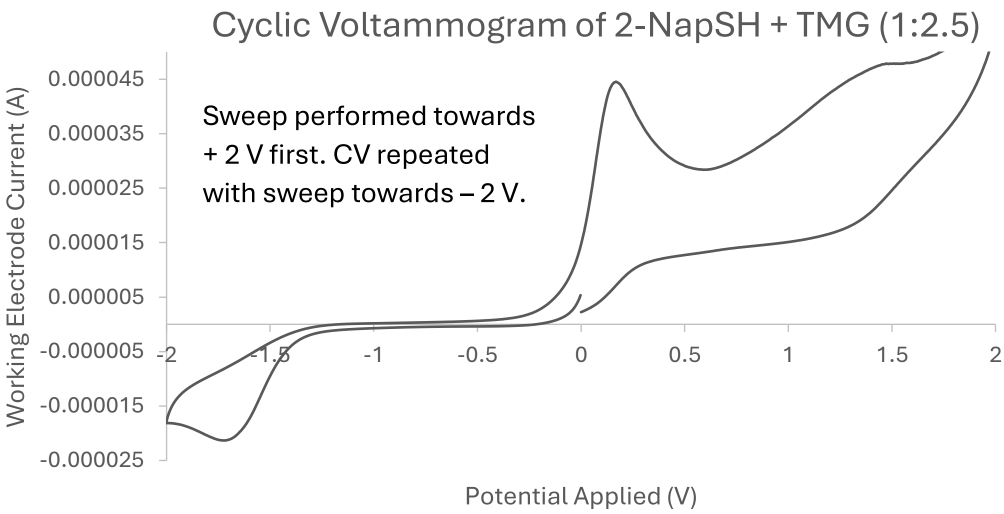

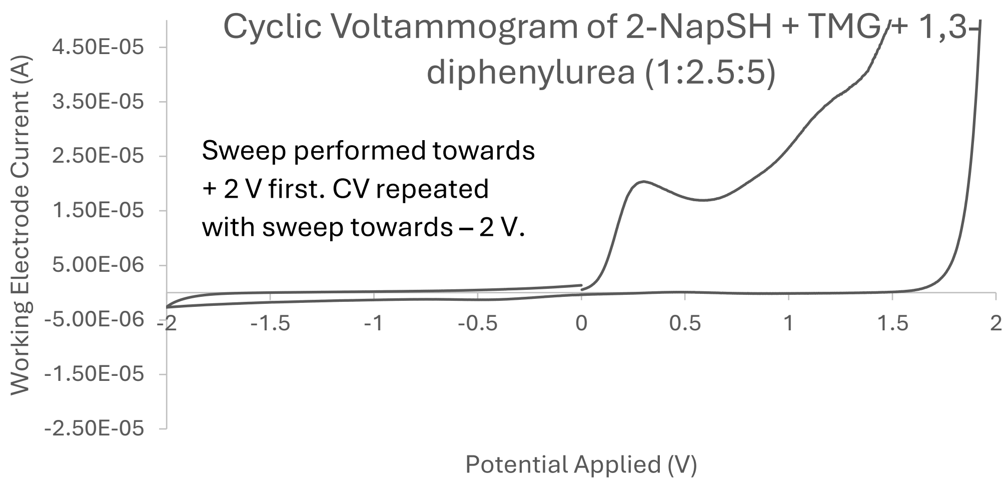
Cyclic Voltammogram of 2-NapSH + TMG + 1,3-diphenylurea (1:2.5:5)
Sweep performed towards + 2 V first. CV repeated with sweep towards – 2 V.
Working Electrode Current (A)
4.50E-05
3.50E-05
2.50E-05
1.50E-05
5.00E-06
-5.00E-06
-1.50E-05
-2.50E-05
-2
-1.5
-1
-0.5
0
0.5
1
1.5
2
Potential Applied (V)

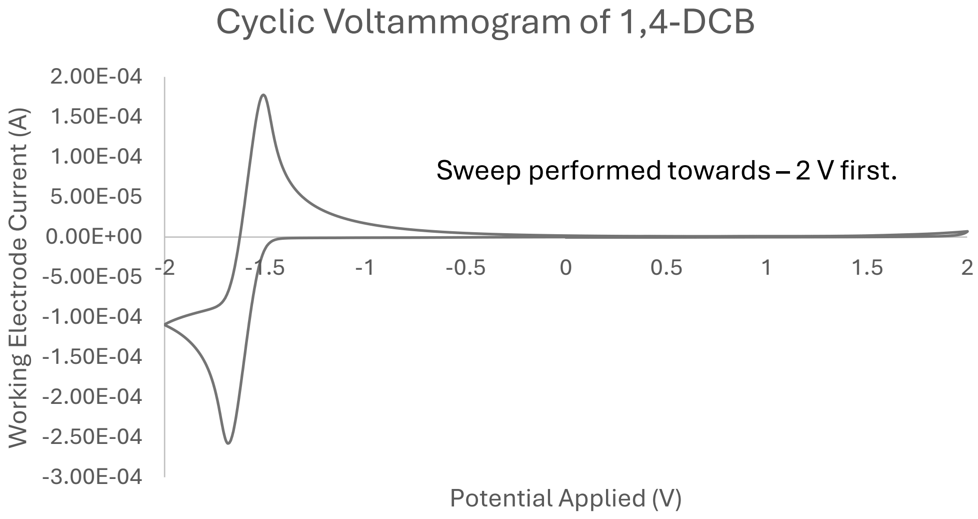
Cyclic Voltammogram of 1,4-DCB
Sweep performed towards – 2 V first.
Working Electrode Current (A)
2.00E-04
1.50E-04
1.00E-04
5.00E-05
0.00E+00
-5.00E-05
-1.00E-04
-1.50E-04
-2.00E-04
-2.50E-04
-3.00E-04
-2
-1.5
-1
-0.5
0
0.5
1
1.5
2
Potential Applied (V)

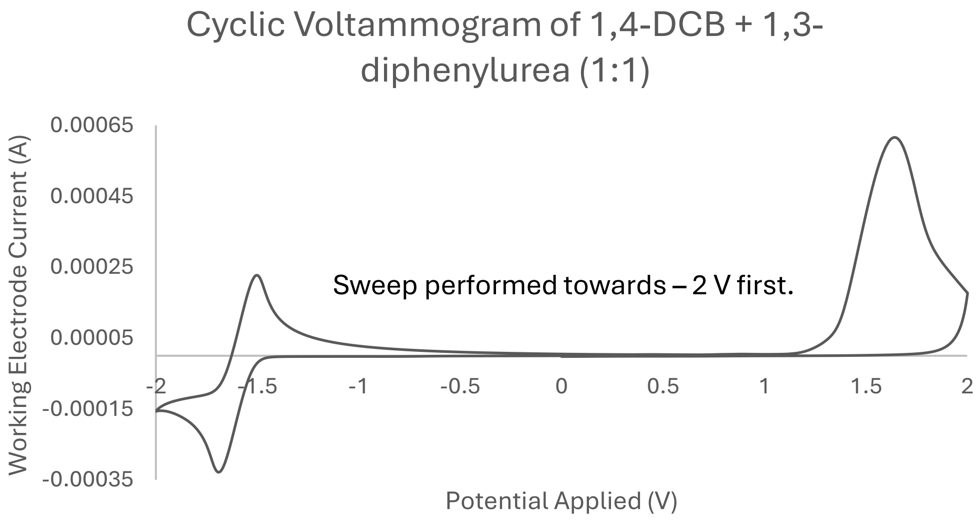
Cyclic Voltammogram of 1,4-DCB + 1,3-diphenylurea (1:1)
Sweep performed towards – 2 V first.
Working Electrode Current (A)
0.00065
0.00045
0.00025
0.00005
-0.00015
-0.00035
-2
-1.5
-1
-0.5
0
0.5
1
1.5
2
Potential Applied (V)

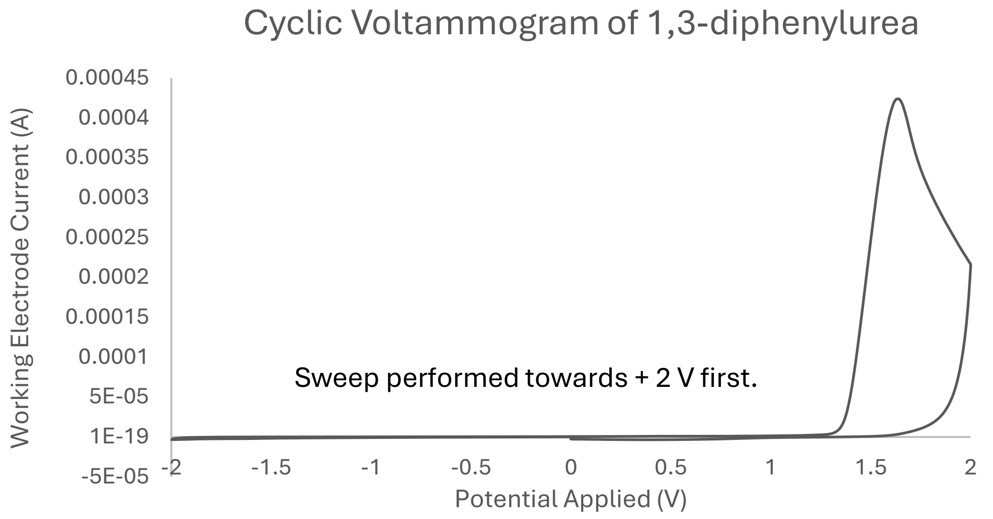

Figure S14. Cyclic Voltammograms of TMG, 2-NapSH, 2-NapSH + TMG, 2-NapSH + TMG + 1,3-diphenylurea, 1,4-DCB, 1,4-DCB + 1,3-diphenylurea, and 1,3-diphenylurea.

Cyclic voltammetry studies were conducted on 2-NapSH, TMG, 1,4-DCB, 1,3-diphenylurea and associated combinations of these components. Solutions containing combinations were produced with the same relative concentrations as are present in the reaction mixture: 2-NapSH + TMG (1:2.5); 2-NapSH + TMG + 1,3-diphenylurea (1:2.5:5); 1,4-DCB + 1,3-diphenylurea (1:1). Electrolyte solutions contained 0.05 mmol of the substrate to be measured (0.05 mmol of 2-NapSH in the solutions containing the mixture) and 0.5 mmol of *n*-Bu_4PF_6 as the electrolyte in 5 mL of dry MeCN. Cyclic voltammograms were recorded over a potential rage of −2.0 – +2.0 V, with a scan rate of 0.1 Vs^{-1} and a step interval of 0.00244 V against a Ag/AgCl electrode.

Key features:

1) No shift in half-wave potential of 1,4-DCB is observed in the presence of 1,3-diphenylurea in either the negative direction (hydrogen bonding to the nitrile is unlikely – no activation towards reduction) or the positive direction (stabilisation of the radical anion intermediate by the 1,3-diphenylurea is unlikely).
2) Facile oxidation of the thiolate anion is easily accessible to the excited state of $Ir(ppy)_3$.
3) Complete suppression of a reduction process occurring at the same half-wave potential as 1,4-DCB in the guanidium thiolate salt is observed in the presence of 1,3-diphenylurea.
4) This competitive reduction process is not present in any of the components in isolation, and so is most likely attributed to the reduction of the acceptor component (the guanidinium cation) of a charge transfer complex that is present within the guanidium thiolate salt. The irreversible nature of this reduction suggests a subsequent chemical reaction follows the redox process; it is possible that this could involve the evolution of H_2 at the electrode, leading to the generation of the neutral TMG species which cannot be easily oxidised in the reverse sweep.

8. General Procedures

General Procedure A: Silane-mediated α-amino radical generation

A 4 mL screw top vial was evacuated and back-refilled with N_2 (3×). DMA (0.1 mL), the relevant amine (0.6 mmol, 3.0 equiv), TMG (13 µL, 0.1 mmol, 0.5 equiv) and the relevant aldehyde (0.6 mmol, 3.0 equiv) were added and the mixture was stirred at rt for 5 min. The relevant arene (0.20 mmol, 1.0 equiv), $Ir(ppy)_3$ (1.3 mg, 1 mol%), 2-NapSH (6.4 mg, 20 mol%) and 1,3-diphenylurea (44 mg, 1.0 equiv) were added to the vial sequentially. The cap was returned, and the vial was evacuated and back-refilled with N_2 (×3). DMA (0.9 mL, 0.2 M) and TTMSS (126 µL, 0.40 mmol, 2.0 equiv) were added and the vial was evacuated and back-refilled with N_2 (×3). The vial was sealed with parafilm, and the solution was stirred vigorously under blue light irradiation for the stated reaction time. The solution was partitioned between EtOAc and sat. Na_2CO_3, extracted with EtOAc (3 × 15 mL), washed with brine, dried over anhydrous Na_2SO_4, and concentrated *in vacuo*. The crude residue was purified by flash column chromatography using the stated eluting system.

General Procedure B: Hantzsch Ester variation

A 4 mL screw top vial was evacuated and back-refilled with N_2 (3×). DMA (0.1 mL), the relevant amine (0.6 mmol, 3.0 equiv), TMG (13 µL, 0.1 mmol, 0.5 equiv) and the relevant aldehyde (0.6 mmol, 3.0 equiv) were added and the mixture was stirred at rt for 5 min. The relevant arene (0.20 mmol, 1.0 equiv), $Ir(ppy)_3$ (1.3 mg, 1 mol%), and Hantzsch Ester (101 mg, 0.4 mmol, 2.0 equiv) were added to the vial sequentially. The cap was returned, and the vial was evacuated and back-refilled with N_2 (×3). DMA (0.9 mL, 0.2 M) was added, and the vial was evacuated and back-refilled with N_2 (×3). The vial was sealed with parafilm, and the solution was stirred vigorously under blue light irradiation for the stated reaction time. The solution was partitioned between EtOAc and sat. Na_2CO_3, extracted with EtOAc (3 × 15 mL), washed with brine, dried over anhydrous Na_2SO_4, and concentrated *in vacuo*. The crude residue was purified by flash column chromatography using the stated eluting system.

9. Compound Characterisation

4-(1-Morpholino-3-phenylpropyl)benzonitrile (12a)

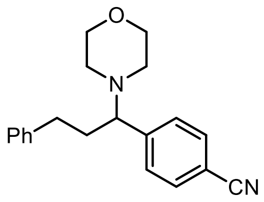

The title compound was synthesised according to General Procedure A, using hydrocinnamaldehyde (distilled prior to use - 79 µL, 0.6 mmol, 3.0 equiv), morpholine (52 µL, 0.6 mmol, 3.0 equiv) and 1,4-DCB (25.6 mg, 0.2 mmol, 1.0 equiv). Irradiation time 1 h. Purification by flash column chromatography on silica gel (0 – 40% EtOAc:PET) afforded the title compound as a yellow oil (46 mg, 0.150 mmol, 75%). **^{1}H NMR** (500 MHz, $CDCl_3$) δ 7.66 – 7.64 (m, 2H), 7.39 – 7.37 (m, 2H), 7.27 – 7.24 (m, 2H), 7.20 – 7.16 (m, 1H), 7.08 – 7.06 (m, 2H), 3.67 (t, *J* = 4.7 Hz, 4H), 3.34 (dd, *J* = 8.7, 5.2 Hz, 1H), 2.45 – 2.40 (m, 4H), 2.37 – 2.33 (m, 2H), 2.29 – 2.22 (m, 1H), 2.01 – 1.93 (m, 1H). **^{13}C NMR** (101 MHz, $CDCl_3$) δ 146.2, 141.5, 132.2, 129.5, 128.5, 128.4, 126.1, 118.9, 111.3, 69.5, 67.2, 51.0, 33.9, 32.1. **IR** (film) ν_{max}/cm^{-1} 3026, 2953, 2853, 2227 (C≡N), 1701, 1602, 1496, 1452, 1396, 1347, 1245, 1175, 1115, 1069, 958, 921, 838, 751, 699, 622, 601, 565, 496, 464. **HRMS** (ESI+) calculated for $C_{20}H_{22}N_2O$ $[M+H]^+$ 307.18049, found 307.18136.

4-(1-Morpholinobutyl)benzonitrile (12b)

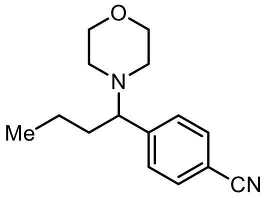

The title compound was synthesised according to General Procedure A, using butyraldehyde (distilled prior to use - 54 µL, 0.6 mmol, 3.0 equiv), morpholine (52 µL, 0.6 mmol, 3.0 equiv) and 1,4-DCB (25.6 mg, 0.2 mmol, 1.0 equiv). Irradiation time 1 h. Purification by flash column chromatography on silica gel (0 – 40% EtOAc:PET) afforded the title compound as a yellow oil (39 mg, 0.160 mmol, 80%). **^{1}H NMR** (400 MHz, $CDCl_3$) δ 7.59 (d, *J* = 7.9 Hz, 2H), 7.35 (d, *J* = 8.1 Hz, 2H), 3.64 (t, *J* = 4.7 Hz, 4H), 3.24 (dd, *J* = 9.2, 4.7 Hz, 1H), 2.46 – 2.39 (m, 2H), 2.34 – 2.27 (m, 2H), 1.88 – 1.80 (m, 1H), 1.64 – 1.55 (m, 1H), 1.13 – 0.93 (m, 2H), 0.82 (t, *J* = 7.3 Hz, 3H). **^{13}C NMR** (101 MHz, $CDCl_3$) δ 147.3, 132.1, 129.3, 119.0, 111.0, 70.2, 67.2, 51.3, 34.6, 19.1, 14.2. **IR** (film) ν_{max}/cm^{-1} 2957, 2855, 2808, 2227 (C≡N), 1607, 1502, 1452, 1395, 1348, 1269, 1208, 1115, 1070, 1032, 1018, 992, 978, 922, 876, 865, 846, 807, 758, 730, 648, 568. **HRMS** (ESI+) calculated for $C_{15}H_{20}N_2O$ $[M+H]^+$ 245.16484, found 245.16480.

4-(2-Methyl-1-morpholinobutyl)benzonitrile (12c)

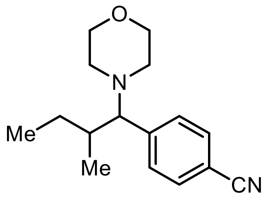

The title compound was synthesised according to General Procedure A, using 2-methylbutyraldehyde (distilled prior to use - 64 µL, 0.6 mmol, 3.0 equiv), morpholine (52 µL, 0.6 mmol, 3.0 equiv) and 1,4-DCB (25.6 mg, 0.2 mmol, 1.0 equiv). Irradiation time 2 h. Purification by flash column chromatography on silica gel (0 – 40% PET:(PET:Et_2O 90:10 + 3% Et_3N) afforded the title compound as a yellow oil in a 1.3:1 mixture of diastereoisomers (39 mg, 0.151 mmol, 76%). **^{1}H NMR** (400 MHz, $CDCl_3$) δ 7.60 (m, $2H_{major + minor}$), 7.33 – 7.26 (m, $2H_{major + minor}$), 3.67 (m, $4H_{major + minor}$), 3.16 (d, J = 7.1 Hz, $1H_{major}$), 3.12 (d, J = 7.8 Hz, $1H_{minor}$), 2.40 – 2.25 (m, $4H_{major + minor}$), 2.06 – 1.96 (m, $1H_{major + minor}$), 1.54 – 1.40 (m, $1H_{major + minor}$), 0.93 – 0.70 (m, $7H_{major + minor}$). **^{13}C NMR** (101 MHz, $CDCl_3$) δ 144.2 (d_{major}), 144.0 (d_{minor}), 131.7 (d_{minor}), 131.7 (d_{major}), 130.1 (d_{major}), 130.1 (d_{minor}), 119.1 ($d_{major + minor}$), 111.0 ($d_{major + minor}$), 75.4 (d_{minor}), 74.5 (d_{major}), 67.4 ($d_{major + minor}$), 51.0 (d_{major}), 50.8 (d_{minor}), 34.3 (d_{minor}), 34.1 (d_{major}), 26.9 (d_{major}), 24.3 (d_{minor}), 16.7 (d_{minor}), 14.2 (d_{major}), 11.6 (d_{major}), 11.5 (d_{minor}). **IR** (film) ν_{max}/cm^{-1} 2961, 2929, 2875, 2853, 2811, 2227, 1606, 1502, 1451, 1379, 1304, 1288, 1273, 1252, 1117, 1070, 1003, 916, 877, 866, 569. **HRMS** (ESI+) calculated for $C_{16}H_{23}N_2O$ $[M+H]^+$ 259.18049, found 259.18078.

Methyl 4-(4-cyanophenyl)-4-morpholinobutanoate (12d)

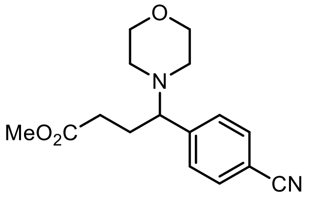

The title compound was synthesised according to General Procedure A, using methyl 4-oxobutanoate (63 uL, 0.6 mmol, 3.0 equiv), morpholine (52 µL, 0.6 mmol, 3.0 equiv) and 1,4-DCB (25.6 mg, 0.2 mmol, 1.0 equiv). Irradiation time 3 h. Purification by flash column chromatography on silica gel (0 – 20% PET:EtOAc) afforded the title compound as a pale yellow oil (29 mg, 0.101 mmol, 51%). **^{1}H NMR** (500 MHz, $CDCl_3$) δ 7.64 – 7.60 (m, 2H), 7.37 – 7.33 (m, 2H), 3.65 (m, 4H), 3.62 (s, 3H), 3.35 (dd, J = 8.0, 5.8 Hz, 1H), 2.44 – 2.33 (m, 4H), 2.32 – 2.23 (m, 1H), 2.20 – 2.14 (m, 2H), 1.97 – 1.89 (m, 1H). **^{13}C NMR** (126 MHz, $CDCl_3$) δ 173.6, 145.6, 132.3, 129.3, 118.8, 111.5, 69.2, 67.2, 51.8, 51.0, 30.5, 27.3. **IR** (film) ν_{max}/cm^{-1} 2952, 2922, 2852, 2813, 2227 (C≡N), 1732, 1607, 1503, 1452, 1436, 1417, 1349, 1330, 1267, 1205, 1170, 1115, 1070, 1014, 962, 922, 893, 853, 765, 568. **HRMS** (ESI+) calculated for $C_{16}H_{21}N_2O_3$ $[M+H]^+$ 289.15467, found 289.15519.

4-(7-Hydroxy-3,7-dimethyl-1-morpholinooctyl)benzonitrile (12e)

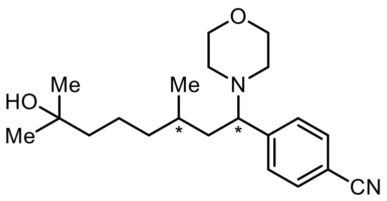

The title compound was synthesised according to General Procedure A, using hydroxycitronellal (112 uL, 0.6 mmol, 3.0 equiv), morpholine (52 µL, 0.6 mmol, 3.0 equiv) and 1,4-DCB (25.6 mg, 0.2 mmol, 1.0 equiv). Irradiation time 1 h. Purification by flash column chromatography on silica gel (0 – 60% PET:EtOAc) afforded the title compound as pale yellow 1:1 mixture of diastereoisomers (49 mg, 0.142 mmol, 71%). **^{1}H NMR** (400 MHz, $CDCl_3$) δ 7.65 – 7.55 (m, 2H$_{a\ and\ b}$), 7.34 (m, 2H$_{a\ and\ b}$), 3.64 (m, 4H$_{a\ and\ b}$), 3.41 (dd, *J* = 8.5, 6.2 Hz, 1H$_a$), 3.35 (dd, *J* = 9.1, 6.1 Hz, 1H$_b$), 2.48 – 2.30 (m, 4H$_{a\ and\ b}$), 1.97 – 1.86 (m, 1H$_a$), 1.69 (m, 1H$_{a\ and\ b}$), 1.48 – 1.42 (m, 1H$_b$), 1.39 – 1.19 (m, 9H$_{a\ and\ b}$), 1.16 (s, 3H$_{a\ or\ b}$), 1.14 – 0.92 (m, 2H$_{a\ and\ b}$), 0.85 (d, *J* = 6.4 Hz, 3H$_{a\ or\ b}$), 0.82 (d, *J* = 6.4 Hz, 3H$_{a\ or\ b}$). **^{13}C NMR** (126 MHz, $CDCl_3$) δ 146.6 (d_a), 146.4 (d_b), 132.2 (d_a), 132.1 (d_b), 129.5 (d_a), 129.4 (d_b), 119.0 ($d_{a\ and\ b}$), 111.2 (d_b), 111.1 (d_a), 71.0 (d_b), 71.0 (d_a), 68.3 ($d_{a\ or\ b}$), 67.9 ($d_{a\ or\ b}$), 67.3 ($d_{a\ and\ b}$), 51.3 (d_b), 50.9 (d_a), 44.2 (d_a), 44.0 (d_b), 39.6 (d_b), 39.5 (d_a), 38.5 (d_a), 37.0 (d_b), 29.8 ($d_{a\ or\ b}$), 29.7 ($d_{a\ or\ b}$), 29.5 ($d_{a\ or\ b}$), 29.5 ($d_{a\ or\ b}$), 29.4 ($d_{a\ or\ b}$), 29.4 ($d_{a\ or\ b}$), 21.5 (d_b), 21.5 (d_a), 20.4 (d_b), 19.5 (d_a). The region from δ = 29.85 – 29.41 ppm has 6 signals owed to the presence of diastereotopic methyl groups within two sets of diastereoisomers (4 signals) overlayed with the tertiary carbon (attached to the stereogenic methyl group) from the two diastereoisomers.

IR (film) ν_{max}/cm^{-1} 3430, 2960, 2933, 2855, 2814, 2228 (C≡N), 1607, 1503, 1453, 1377, 1273, 1116, 1070, 998, 924, 908, 880, 868, 845, 764, 568. **HRMS** (ESI+) calculated for $C_{21}H_{33}N_2O_2$ [M+H]$^+$ 345.25365, found 345.25394.

4-(Cyclohexyl(morpholino)methyl)benzonitrile (12f)

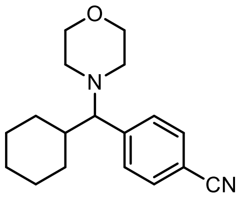

The title compound was synthesised according to General Procedure A, using cyclohexanecarboxaldehyde (distilled prior to use - 73 uL, 0.6 mmol, 3.0 equiv), morpholine (52 μL, 0.6 mmol, 3.0 equiv) and 1,4-DCB (25.6 mg, 0.2 mmol, 1.0 equiv). Irradiation time 2 h. Purification by flash column chromatography on silica gel (0 – 100% PET:CH_2Cl_2) afforded the title compound as a colourless oil (43 mg, 0.151 mmol, 76%). **^{1}H NMR** (700 MHz, $CDCl_3$) δ 7.62 – 7.58 (m, 2H), 7.29 – 7.26 (m, 2H), 3.69 – 3.63 (m, 4H), 3.12 (d, J = 7.5 Hz, 1H), 2.40 – 2.22 (m, 4H), 1.94 – 1.85 (m, 2H), 1.76 – 1.71 (m, 1H), 1.66 – 1.61 (m, 2H), 1.53 – 1.48 (m, 1H), 1.28 – 1.22 (m, 1H), 1.18 – 1.11 (m, 1H), 1.06 – 0.99 (m, 1H), 0.79 – 0.68 (m, 2H). **^{13}C NMR** (176 MHz, $CDCl_3$) δ 144.1, 131.7, 130.1, 119.1, 110.9, 75.4, 67.4, 50.8, 37.6, 31.2, 28.6, 26.8, 26.5, 26.3. **IR** (film) ν_{max}/cm^{-1} 2923, 2851, 2227 (C≡N), 1606, 1502, 1449, 1394, 1346, 1273, 1254, 1117, 1056, 1033, 1012, 922, 894, 876, 866, 830, 758, 567. **HRMS** (ESI+) calculated for $C_{18}H_{25}N_2O$ $[M+H]^+$ 285.19614, found 285.19647.

4-(Morpholino(tetrahydro-2H-pyran-4-yl)methyl)benzonitrile (12g)

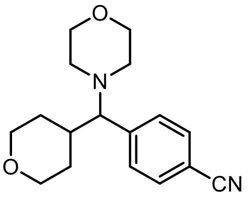

The title compound was synthesised according to General Procedure A, using tetrahydropyran-4-carbaldehyde (62 uL, 0.6 mmol, 3.0 equiv), morpholine (52 μL, 0.6 mmol, 3.0 equiv) and 1,4-DCB (25.6 mg, 0.2 mmol, 1.0 equiv). Irradiation time 2 h. Purification by flash column chromatography on silica gel (0 – 100% PET:CH_2Cl_2) afforded the title compound as a colourless oil (37 mg, 0.129 mmol, 65%). **^{1}H NMR** (500 MHz, $CDCl_3$) δ 7.64 (d, J = 8.1 Hz, 2H), 7.26 – 7.23 (m, 2H), 4.00 (dd, J = 11.5, 3.3 Hz, 1H), 3.88 (dd, J = 11.4, 2.9 Hz, 1H), 3.71 – 3.60 (m, 4H), 3.41 (td, J = 11.9, 2.1 Hz, 1H), 3.31 (td, J = 11.8, 2.3 Hz, 1H), 3.20 (d, J = 8.9 Hz, 1H), 2.40 – 2.26 (m, 4H), 2.23 – 2.10 (m, 1H), 1.91 – 1.84 (m, 1H), 1.32 – 1.21 (m, 2H), 1.14 (qd, J = 12.0, 4.3 Hz, 1H). **^{13}C NMR** (126 MHz, $CDCl_3$) δ 142.2, 131.9, 130.0, 118.8, 111.4, 74.8, 68.2, 67.8, 67.3, 50.3, 35.0, 31.1, 29.6. **IR** (film) ν_{max}/cm^{-1} 2951, 2848, 2228 (C≡N), 1606, 1453, 1275, 1260, 1236, 1115, 1091, 1019, 989, 922, 878, 863, 824, 764, 750, 569. **HRMS** (ESI+) calculated for $C_{17}H_{23}N_2O_2$ $[M+H]^+$ 287.17540, found 287.17559.

4-(Cyclohex-3-en-1-yl(morpholino)methyl)benzonitrile (12h)

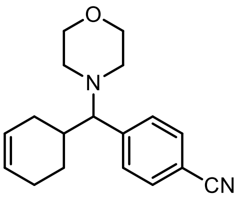

The title compound was synthesised according to General Procedure A, using 1,2,3,6-tetrahydrobenzaldehyde (distilled prior to use - 68 uL, 0.6 mmol, 3.0 equiv), morpholine (52 µL, 0.6 mmol, 3.0 equiv) and 1,4-DCB (25.6 mg, 0.2 mmol, 1.0 equiv). Irradiation time 2 h. Purification by flash column chromatography on silica gel (0 – 100% PET:CH_2Cl_2) afforded the title compound as a colourless oil (38 mg, 0.135 mmol, 68%). **^{1}H NMR** (400 MHz, $CDCl_3$) δ 7.66 – 7.56 (m, 2H$_{major\ and\ minor}$), 7.30 – 7.26 (m, 2H$_{major\ and\ minor}$), 5.71 – 5.50 (m, 2H$_{major\ and\ minor}$), 3.71 – 3.62 (m, 4H$_{major\ and\ minor}$), 3.27 (d, J = 8.7 Hz, 1H$_{major}$), 3.24 (d, J = 8.4 Hz, 1H$_{minor}$), 2.41 – 2.18 (m, 6H$_{major\ and\ minor}$), 2.12 – 2.01 (m, 2H$_{major\ and\ minor}$), 1.99 – 1.92 (m, 1H$_{major\ and\ minor}$), 1.84 – 1.74 (m, 1H$_{major\ and\ minor}$), 1.58 – 1.45 (m, 1H$_{major\ and\ minor}$), 1.22 – 1.10 (m, 1H$_{major}$), 1.10 – 0.95 (m, 1H$_{minor}$). **^{13}C NMR** (101 MHz, $CDCl_3$) δ 143.3 (d_{minor}), 142.9 (d_{major}), 131.8 (d_{minor}), 131.7 (d_{major}), 130.1 (d_{major}), 130.0 (d_{minor}), 127.3 (d_{major}), 127.0 (d_{minor}), 126.5 (d_{minor}), 125.9 (d_{major}), 119.0 ($d_{major\ +\ minor}$), 111.1 (d_{minor}), 111.1 (d_{major}), 74.3 (d_{major}), 74.3 (d_{minor}), 67.4 (d_{major}), 67.3 (d_{minor}), 50.4 ($d_{major\ +\ minor}$), 33.5 (d_{minor}), 33.2 (d_{major}), 29.7 (d_{minor}), 28.0 (d_{major}), 26.4 (d_{major}), 25.2 (d_{major}), 25.1 (d_{minor}), 24.9 (d_{minor}). **IR** (film) ν_{max}/cm^{-1} 3019, 2956, 2911, 2852, 2228 (C≡N), 1606, 1502, 1451, 1410, 1275, 1261, 1117, 1070, 1004, 922, 881, 862, 833, 764, 750, 704, 656, 568. **HRMS** (ESI+) calculated for $C_{18}H_{23}N_2O$ [M+H]$^+$ 283.18049, found 283.18074.

***Tert*-butyl 4-((4-cyanophenyl)(morpholino)methyl)piperidine-1-carboxylate (12i)**

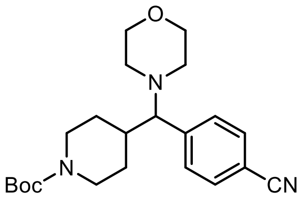

The title compound was synthesised according to General Procedure A, using *N*-Boc-4-piperidinecarboxaldehyde (128 mg, 0.6 mmol, 3.0 equiv – the aldehyde was dissolved in 0.1 mL DMA and added onto a mixture containing 0.1 mL DMA, morpholine and TMG), morpholine (52 μL, 0.6 mmol, 3.0 equiv) and 1,4-DCB (25.6 mg, 0.2 mmol, 1.0 equiv). 0.8 mL DMA was added at the end to bring the total volume to 1 mL. Irradiation time 2 h. Purification by flash column chromatography on silica gel (0 – 10% PET (+3% Et_3N):EtOAc) followed by SCX afforded the title compound as a colourless oil (53 mg, 0.137 mmol, 69%). **^{1}H NMR** (500 MHz, $CDCl_3$) δ 7.62 (d, J = 7.9 Hz, 2H), 7.24 (d, J = 8.0 Hz, 2H), 4.21 – 3.91 (m, 2H), 3.67 – 3.61 (m, 4H), 3.18 (d, J = 8.5 Hz, 1H), 2.72 – 2.54 (m, 2H), 2.37 – 2.24 (m, 4H), 2.09 – 1.99 (m, 1H), 1.91 (m, 1H), 1.42 – 1.36 (m, 10H), 1.07 – 0.85 (m, 2H). **^{13}C NMR** (176 MHz, $CDCl_3$) δ 154.9, 142.5, 132.0, 130.0, 118.9, 111.4, 79.6, 74.6, 67.3, 50.4, 36.1, 30.2, 28.6. Note: the signal at δ 30.2 ppm represents one of the CH_2 environments of the piperidine ring. This signal is very weak (although cross-peaks can be observed in the HSQC and HMBC), and the other signal representing the remaining α-amino-CH_2 of this ring cannot be observed. **IR** (film) ν_{max}/cm^{-1} 2954, 2924, 2853, 2228 (C≡N), 1686, 1450, 1424, 1365, 1277, 1250, 1224, 1162, 1117, 1015, 865, 764, 750, 567. **HRMS** (ESI+) calculated for $C_{22}H_{32}N_3O_3$ [M+H]$^+$ 386.24382, found 386.24380.

***Tert*-butyl 3-((4-cyanophenyl)(morpholino)methyl)azetidine-1-carboxylate (12j)**

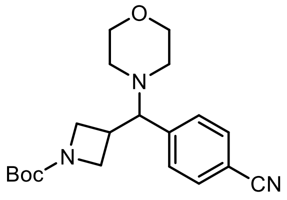

The title compound was synthesised according to General Procedure A, using 1-boc-3-formylazetidine (111 mg, 0.6 mmol, 3.0 equiv – the aldehyde was dissolved in 0.1 mL DMA and added onto a mixture containing 0.1 mL DMA, morpholine and TMG), morpholine (52 μL, 0.6 mmol, 3.0 equiv) and 1,4-DCB (25.6 mg, 0.2 mmol, 1.0 equiv). 0.8 mL DMA was added at the end to bring the total volume to 1 mL. Irradiation time 3 h. Purification by flash column chromatography on silica gel (0 – 10% PET (+3% Et_3N):EtOAc) followed by SCX afforded the title compound as a colourless oil (53 mg, 0.148 mmol, 74%). **^{1}H NMR** (400 MHz, $CDCl_3$) δ 7.65 – 7.60 (m, 2H), 7.36 – 7.32 (m, 2H), 4.08 (t, *J* = 8.4 Hz, 1H), 3.85 (dd, *J* = 8.8, 5.9 Hz, 1H), 3.62 (t, *J* = 4.6 Hz, 4H), 3.57 (t, *J* = 8.7 Hz, 1H), 3.49 (d, *J* = 10.5 Hz, 1H), 3.25 (dd, *J* = 8.9, 6.3 Hz, 1H), 3.05 – 2.91 (m, 1H), 2.36 – 2.26 (m, 4H), 1.40 (s, 9H). **^{13}C NMR** (101 MHz, $CDCl_3$) δ 156.2, 144.0, 132.4, 129.4, 118.6, 112.0, 79.7, 73.8, 67.1, 51.1, 31.7, 28.5. Note that the signals corresponding to the *C*H_2 signals of the azetidine ring are not visible in the ^{13}C NMR spectrum owed to very fast relaxation. However, cross peaks from these signals are visible in the HSQC, enabling them to be located at 54.8 and 51.9 ppm, respectively. **IR** (film) ν_{max}/cm^{-1} 2959, 2926, 2882, 2855, 2815, 2228 (C≡N), 1692, 1607, 1503, 1478, 1452, 1405, 1392, 1365, 1289, 1264, 1254, 1209, 1141, 1116, 1070, 1031, 994, 940, 876, 862, 842, 771, 756, 734, 702, 567. **HRMS** (ESI+) calculated for $C_{20}H_{28}N_3O_3$ $[M+H]^+$ 358.21252, found 358.21256.

Methyl 4-(4-cyanophenyl)-4-thiomorpholinobutanoate (12k)

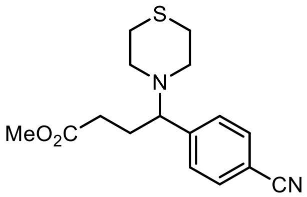

The title compound was synthesised according to General Procedure A, using methyl 4-oxobutanoate (63 uL), thiomorpholine (60 μL, 0.6 mmol, 3.0 equiv) and 1,4-DCB (25.6 mg, 0.2 mmol, 1.0 equiv). Irradiation time 2 h. Purification by flash column chromatography on silica gel (0 – 10% PET:EtOAc) afforded the title compound as a colourless oil (46 mg, 0.151 mmol, 76%). **^{1}H NMR** (500 MHz, $CDCl_3$) δ 7.64 – 7.61 (m, 2H), 7.31 – 7.27 (m, 2H), 3.65 (s, 3H), 3.52 (t, *J* = 6.9 Hz, 1H), 2.76 – 2.68 (m, 2H), 2.65 – 2.55 (m, 6H), 2.33 – 2.24 (m, 3H), 2.00 – 1.90 (m, 1H). **^{13}C NMR** (126 MHz, $CDCl_3$) δ 173.7, 144.4, 132.2, 129.2, 118.8, 111.5, 69.3, 52.2, 51.8, 31.1, 28.3, 26.4. **IR** (film) ν_{max}/cm^{-1} 2949, 2915, 2815, 2227 (C≡N), 1731, 1607, 1503, 1436, 1417, 1362, 1324, 1282, 1202, 1170, 1121, 1017, 979, 954, 934, 890, 851, 834, 614, 560. **HRMS** (ESI+) calculated for $C_{16}H_{21}N_2O_2S$ $[M+H]^+$ 305.13183, found 305.13222.

1-((4-Cyanophenyl)(cyclohexyl)methyl)piperidine-4-carbonitrile (12l)

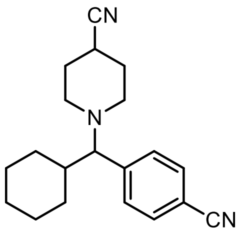

The title compound was synthesised according to General Procedure A, using cyclohexanecarboxaldehyde (distilled prior to use - 73 uL, 0.6 mmol, 3.0 equiv), 4-cyanopiperidine (67 µL, 0.6 mmol, 3.0 equiv) and 1,4-DCB (25.6 mg, 0.2 mmol, 1.0 equiv). Irradiation time 2 h. Purification by flash column chromatography on silica gel (0–20% PET:(PET:Et_2O 90:10 + 3% Et_3N) afforded the title compound as a colourless oil (32 mg, 0.104 mmol, 52%). **^{1}H NMR** (500 MHz, $CDCl_3$) δ 7.65 – 7.60 (m, 2H), 7.26 – 7.23 (m, 2H), 3.19 (d, *J* = 8.9 Hz, 1H), 2.67 – 2.53 (m, 2H), 2.53 – 2.45 (m, 1H), 2.22 – 2.02 (m, 2H), 1.97 – 1.75 (m, 7H), 1.69 – 1.62 (m, 2H), 1.40 – 1.34 (m, 1H), 1.31 – 1.24 (m, 1H), 1.22 – 1.05 (m, 2H), 0.91 – 0.81 (m, 1H), 0.78 – 0.68 (m, 1H). **^{13}C NMR** (126 MHz, $CDCl_3$) δ 143.4, 131.8, 129.7, 121.8, 119.0, 111.0, 75.2, 47.4 (2 × α-amino CH$_2$), 37.7, 31.0, 29.6, 29.4, 29.3, 26.7, 26.6, 26.4, 26.2. **IR** (film) ν_{max}/cm^{-1} 2927, 2851, 2807, 2228 (C≡N), 1686, 1606, 1501, 1447, 1414, 1262, 1125, 1091, 1044, 1006, 966, 893, 861, 841, 768, 757, 746, 562. **HRMS** (ESI+) calculated for $C_{20}H_{26}N_3$ [M+H]$^+$ 308.21212, found 308.21245.

4-(Cyclohexyl(1,4-dioxa-8-azaspiro[4.5]decan-8-yl)methyl)benzonitrile (12m)

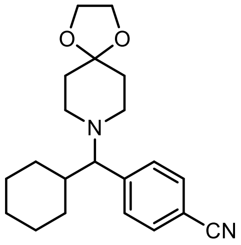

The title compound was synthesised according to General Procedure A, using cyclohexanecarboxaldehyde (distilled prior to use - 73 uL, 0.6 mmol, 3.0 equiv), 1,4-dioxa-8-azaspiro[4.5]decane (77 uL, 0.6 mmol, 3.0 equiv) and 1,4-DCB (25.6 mg, 0.2 mmol, 1.0 equiv). Irradiation time 2 h. Purification by flash column chromatography on silica gel (0–40% PET:(PET:Et_2O 90:10 + 3% Et_3N) afforded the title compound as a colourless oil (46 mg, 0.135 mmol, 68%). **^{1}H NMR** (400 MHz, $CDCl_3$) δ 7.61 – 7.57 (m, 2H), 7.24 – 7.21 (m, 2H), 3.87 (s, 4H), 3.20 (d, *J* = 9.0 Hz, 1H), 2.42 – 2.28 (m, 4H), 2.03 – 1.96 (m, 1H), 1.96 – 1.84 (m, 1H), 1.78 – 1.61 (m, 7H), 1.39 – 1.31 (m, 1H), 1.25 – 1.18 (m, 1H), 1.17 – 1.04 (m, 2H), 0.89 – 0.81 (m, 1H), 0.76 – 0.64 (m, 1H). **^{13}C NMR** (101 MHz, $CDCl_3$) δ 144.1, 131.7, 129.8, 119.2, 110.7, 107.4, 74.8, 64.3, 47.6, 38.1, 35.5, 31.1, 29.8, 26.8, 26.4, 26.3. **IR** (film) ν_{max}/cm^{-1} 2923, 2849, 2227 (C≡N), 1606, 1501, 1468, 1449, 1418, 1363, 1340, 1309, 1269, 1228, 1211, 1142, 1087, 1073, 1039, 964, 947, 912, 861, 831. **HRMS** (ESI+) calculated for $C_{21}H_{29}N_2O_2$ [M+H]$^+$ 341.22235, found 341.22261.

4-(Cyclohexyl(4-(pyrimidin-2-yl)piperazin-1-yl)methyl)benzonitrile (12n)

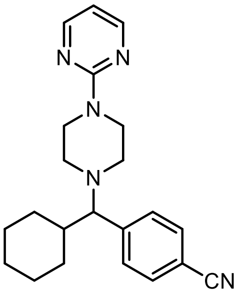

The title compound was synthesised according to General Procedure A, using cyclohexanecarboxaldehyde (distilled prior to use - 73 uL, 0.6 mmol, 3.0 equiv), 1-(2-pyrimidyl)-piperazine (99 mg, 0.6 mmol, 3.0 equiv) and 1,4-DCB (25.6 mg, 0.2 mmol, 1.0 equiv). Irradiation time 2 h. Purification by flash column chromatography on silica gel (0–20% PET:(PET:Et_2O 90:10 + 3% Et_3N) afforded the title compound as a brown oil (54 mg, 0.149 mmol, 75%). **^{1}H NMR** (500 MHz, $CDCl_3$) δ 8.24 (d, *J* = 4.7 Hz, 2H), 7.61 – 7.56 (m, 2H), 7.26 – 7.24 (m, 2H), 6.42 (t, *J* = 4.7 Hz, 1H), 3.82 – 3.73 (m, 4H), 3.21 (d, *J* = 8.6 Hz, 1H), 2.41 – 2.30 (m, 4H), 2.04 – 1.92 (m, 2H), 1.80 – 1.75 (m, 1H), 1.67 – 1.62 (m, 2H), 1.47 – 1.42 (m, 1H), 1.30 – 1.08 (m, 3H), 0.90 – 0.82 (m, 1H), 0.78 – 0.69 (m, 1H). **^{13}C NMR** (101 MHz, $CDCl_3$) δ 161.6, 157.8, 143.7, 131.7, 130.0, 119.1, 110.9, 109.9, 75.2, 49.8, 44.1, 37.7, 31.1, 29.4, 26.8, 26.4, 26.2. **IR** (film) ν_{max}/cm^{-1} 2925, 2850, 2227 (C≡N), 1585, 1546, 1501, 1446, 1393, 1358, 1307, 1260, 1221, 1176, 1139, 1004, 983, 958, 861, 832, 796, 639, 598, 554. **HRMS** (ESI+) calculated for $C_{22}H_{28}N_5$ $[M+H]^+$ 362.23392, found 362.23414.

4-(Cyclohexyl(4-(methylsulfonyl)piperazin-1-yl)methyl)benzonitrile (12o)

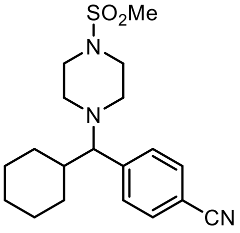

The title compound was synthesised according to General Procedure A, using cyclohexanecarboxaldehyde (distilled prior to use - 73 uL, 0.6 mmol, 3.0 equiv), 4-methanesulfonylpiperazine (99 mg, 0.6 mmol, 3.0 equiv) and 1,4-DCB (25.6 mg, 0.2 mmol, 1.0 equiv). Irradiation time 2 h. Purification by flash column chromatography on silica gel (0–60% PET:(PET:Et_2O 90:10 + 3% Et_3N) afforded the title compound as a pale yellow oil (47 mg, 0.130 mmol, 65%). **^{1}H NMR** (500 MHz, $CDCl_3$) δ 7.63 – 7.60 (m, 2H), 7.25 – 7.23 (m, 2H), 3.23 – 3.15 (m, 5H), 2.74 (s, 3H), 2.48 – 2.38 (m, 4H), 1.96 – 1.88 (m, 2H), 1.78 – 1.71 (m, 1H), 1.68 – 1.61 (m, 2H), 1.45 – 1.38 (m, 1H), 1.29 – 1.24 (m, 1H), 1.22 – 1.12 (m, 1H), 1.12 – 1.01 (m, 1H), 0.86 – 0.77 (m, 1H), 0.77 – 0.68 (m, 1H). **^{13}C NMR** (126 MHz, $CDCl_3$) δ 143.2, 131.9, 129.8, 118.9, 111.3, 74.8, 49.2, 46.3, 37.7, 34.3, 31.1, 29.2, 26.7, 26.4, 26.2. **IR** (film) ν_{max}/cm^{-1} 2926, 2850, 2227 (C≡N), 1606, 1502, 1452, 1341, 1325, 1259, 1160, 1111, 1002, 960, 863, 833, 778, 735, 702, 643, 601, 562, 533, 518. **HRMS** (ESI+) calculated for $C_{19}H_{28}N_3O_2S$ $[M+H]^+$ 362.18967, found 362.19000.

Tert-butyl (1-(4-cyanophenyl)-4-methoxy-4-oxobutyl)prolinate (12p)

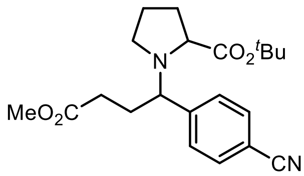

The title compound was synthesised according to General Procedure A, using cyclohexanecarboxaldehyde (73 uL, 0.6 mmol, 3.0 equiv), L-proline *tert*-butyl ester (103 mg, 0.6 mmol, 3.0 equiv) and 1,4-DCB (25.6 mg, 0.2 mmol, 1.0 equiv). Irradiation time 2 h. Purification by flash column chromatography on silica gel (0–50% PET:(PET:Et_2O 90:10 + 3% Et_3N) afforded the title compound as a colourless oil in a 1.1:1 mixture of diastereoisomers (44 mg, 0.118 mmol, 59%). **^{1}H NMR** (500 MHz, $CDCl_3$) δ 7.63 – 7.58 (m, $2H_{major + minor}$), 7.44 – 7.40 (m, $1H_{minor}$), 7.39 – 7.35 (m, $1H_{major}$), 3.80 – 3.74 (m, $1H_{major + minor}$), 3.61 (s, $3H_{major}$), 3.59 (s, $3H_{minor}$), 3.49 (dd, *J* = 9.2, 2.3 Hz, $1H_{minor}$), 3.12 (dd, *J* = 8.8, 3.8 Hz, $1H_{major}$), 2.98 – 2.90 (m, $1H_{major + minor}$), 2.60 – 2.48 (m, $1H_{major + minor}$), 2.29 – 1.72 (m, $8H_{major + minor}$), 1.44 (s, $9H_{major}$), 1.37 (s, $9H_{minor}$). **^{13}C NMR** (101 MHz, $CDCl_3$) δ 174.1 (d_{major}), 173.9 (d_{minor}), 173.7 (d_{major}), 173.4 (d_{minor}), 147.9 (d_{minor}), 146.3 (d_{major}), 132.3 (d_{minor}), 132.2 (d_{major}), 129.4 (d_{minor}), 129.3 (d_{major}), 119.0 (d_{minor}), 118.9 (d_{major}), 111.4 (d_{major}), 111.3 (d_{minor}), 80.7 (d_{major}), 80.7 (d_{minor}), 66.1 (d_{minor}), 64.8 (d_{major}), 63.1 ($d_{major + minor}$), 51.7 (d_{minor}), 51.7 (d_{major}), 51.4 (d_{minor}), 49.4 (d_{major}), 30.8 (d_{major}), 30.6 (d_{minor}), 30.2 (d_{minor}), 30.0 (d_{major}), 29.8 (d_{minor}), 29.7 (d_{major}), 28.2 (d_{major}), 28.1 (d_{minor}), 23.4 (d_{minor}), 23.2 (d_{major}). **IR** (film) ν_{max}/cm^{-1} 2951, 2849, 2228 (C≡N), 1736, 1607, 1503, 1437, 1392, 1367, 1294, 1249, 1212, 1149, 1019, 845. **HRMS** (ESI+) calculated for $C_{21}H_{29}N_2O_4$ $[M+H]^+$ 373.21218, found 373.21254.

Methyl 4-(3-((tert-butoxycarbonyl)amino)azetidine-1-yl)-4-(4-cyanophenyl)butanoate (12q)

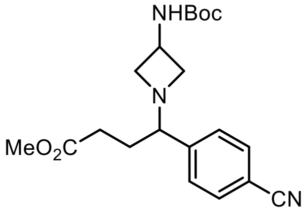

The title compound was synthesised according to General Procedure A, using cyclohexanecarboxaldehyde (73 uL, 0.6 mmol, 3.0 equiv), 3-*N*-Boc-Amino-azetidine (103 mg, 0.6 mmol, 3.0 equiv) and 1,4-DCB (25.6 mg, 0.2 mmol, 1.0 equiv). Irradiation time 2 h. Purification by flash column chromatography on silica gel (0–20% PET:(EtOAc + 3% Et_3N) afforded the title compound as a colourless oil (46 mg, 0.123 mmol, 62%). **^{1}H NMR** (400 MHz, $CDCl_3$) δ 7.63 – 7.58 (m, 2H), 7.39 – 7.34 (m, 2H), 4.82 (s, 1H), 4.24 (s, 1H), 3.67 – 3.62 (m, 1H), 3.61 (s, 3H), 3.41 – 3.34 (m, 1H), 3.26 – 3.19 (m, 1H), 2.90 – 2.81 (m, 1H), 2.78 – 2.67 (m, 1H), 2.18 – 2.08 (m, 1H), 2.06 – 1.95 (m, 2H), 1.84 – 1.72 (m, 1H), 1.42 (s, 9H). **^{13}C NMR** (126 MHz, $CDCl_3$) δ 173.5, 155.1, 146.0, 132.4, 128.9, 118.8, 111.7, 79.9, 72.2, 61.0, 61.0, 51.8, 41.2, 29.5, 29.3, 28.5. **IR** (film) ν_{max}/cm^{-1} 3362, 2952, 2848, 2228 (C≡N), 1735, 1711, 1608, 1522, 1438, 1391, 1366, 1252, 1166, 1044, 1018, 851, 782. **HRMS** (ESI+) calculated for $C_{20}H_{28}N_3O_4$ $[M+H]^+$ 374.20743, found 374.20763.

4-((Bis(2-methoxyethyl)amino)(cyclohexyl)methyl)benzonitrile (12r)

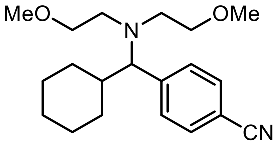

The title compound was synthesised according to General Procedure A, using cyclohexanecarboxaldehyde (distilled prior to use - 73 uL, 0.6 mmol, 3.0 equiv), bis(2-methoxyethyl)amine (89 uL, 0.6 mmol, 3.0 equiv) and 1,4-DCB (25.6 mg, 0.2 mmol, 1.0 equiv). Irradiation time 2 h. Purification by flash column chromatography on silica gel (0–20% PET:(EtOAc + 3% Et_3N) afforded the title compound as a colourless oil (38 mg, 0.115 mmol, 58%). **^{1}H NMR** (500 MHz, $CDCl_3$) δ 7.61 – 7.58 (m, 2H), 7.29 – 7.26 (m, 2H), 3.47 – 3.37 (m, 5H), 3.33 (s, 6H), 2.72 (m, 2H), 2.35 (m, 2H), 2.22 – 2.17 (m, 1H), 1.85 (m, 1H), 1.81 – 1.75 (m, 1H), 1.67 – 1.56 (m, 2H), 1.28 – 1.22 (m, 2H), 1.14 (m, 2H), 0.98 – 0.87 (m, 1H), 0.72 – 0.63 (m, 1H). **^{13}C NMR** (126 MHz, $CDCl_3$) δ 144.2, 131.8, 129.9, 119.1, 110.7, 72.5, 71.6, 59.0, 50.2, 38.3, 30.9, 26.8, 26.2. **IR** (film) ν_{max}/cm^{-1} 2923, 2850, 2227 (C≡N), 1686, 1605, 1502, 1449, 1371, 1291, 1248, 1197, 1116, 1018, 975, 894, 862, 843. **HRMS** (ESI+) calculated for $C_{20}H_{31}N_2O_2$ $[M+H]^+$ 331.23800, found 331.23824.

***Tert*-butyl (2-(((4-cyanophenyl)(cyclohexyl)methyl)(methyl)amino)ethyl)carbamate (12s)**

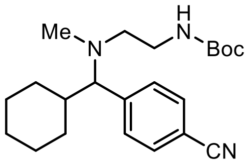

The title compound was synthesised according to General Procedure A, using cyclohexanecarboxaldehyde (distilled prior to use - 73 uL, 0.6 mmol, 3.0 equiv), *N*-boc-2-methylamino-ethylamine (104 mg, 0.6 mmol, 3.0 equiv) and 1,4-DCB (25.6 mg, 0.2 mmol, 1.0 equiv). Irradiation time 2 h. Purification by flash column chromatography on silica gel (0–50% PET:(PET:Et_2O 90:10 + 3% Et_3N) afforded the title compound as a colourless oil (45 mg, 0.121 mmol, 61%). **^{1}H NMR** (500 MHz, $CDCl_3$) δ 7.62 – 7.59 (m, 2H), 7.23 – 7.20 (m, 2H), 4.90 (s, 1H), 3.24 – 3.13 (m, 3H), 2.36 – 2.21 (m, 2H), 2.12 – 2.03 (m, 4H), 1.95 – 1.87 (m, 1H), 1.83 – 1.75 (m, 1H), 1.69 – 1.60 (m, 2H), 1.45 (s, 9H), 1.32 – 1.26 (m, 2H), 1.19 – 1.10 (m, 2H), 0.99 – 0.88 (m, 1H), 0.75 – 0.66 (m, 1H). **^{13}C NMR** (126 MHz, $CDCl_3$) δ 156.2, 143.0, 131.8, 129.9, 119.0, 110.9, 79.3, 73.8, 53.4, 38.0, 37.8, 36.7, 30.8, 30.4, 28.6, 26.8, 26.3, 26.2. **IR** (film) ν_{max}/cm^{-1} 3373, 2925, 2850, 2795, 2227 (C≡N), 1701, 1605, 1501, 1450, 1391, 1365, 1248, 1166, 1030, 967, 894, 862, 780. **HRMS** (ESI+) calculated for $C_{22}H_{34}N_3O_2$ $[M+H]^+$ 372.26455, found 372.26455.

4-(Cyclohexyl((4-methoxyphenyl)amino)methyl)benzonitrile (12t)

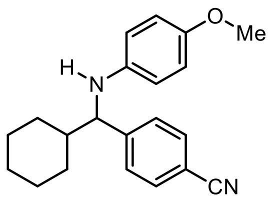

The title compound was synthesised according to General Procedure A, using cyclohexanecarboxaldehyde (distilled prior to use - 73 uL, 0.6 mmol, 3.0 equiv), *p*-anisidine (74 mg, 0.6 mmol, 3.0 equiv) and 1,4-DCB (25.6 mg, 0.2 mmol, 1.0 equiv). Irradiation time 2 h. Purification by flash column chromatography on silica gel (0–50% PET:(PET:Et_2O 90:10 + 3% Et_3N) afforded the title compound as a pale brown oil (38 mg, 0.119 mmol, 60%). **^{1}H NMR** (500 MHz, $CDCl_3$) δ 7.61 – 7.56 (m, 2H), 7.43 – 7.39 (m, 2H), 6.69 – 6.64 (m, 2H), 6.40 – 6.35 (m, 2H), 4.10 (d, *J* = 5.9 Hz, 1H), 3.68 (s, 3H), 1.84 – 1.70 (m, 3H), 1.70 – 1.61 (m, 2H), 1.56 – 1.50 (m, 1H), 1.25 – 0.99 (m, 5H). **^{13}C NMR** (126 MHz, $CDCl_3$) δ 152.2, 149.1, 141.4, 132.2, 128.2, 119.1, 114.9, 114.4, 110.7, 64.3, 55.9, 44.9, 30.3, 29.3, 26.4. **IR** (film) ν_{max}/cm^{-1} 3402, 2926, 2851, 2226 (C≡N), 1606, 1510, 1449, 1413, 1298, 1235, 1178, 1112, 1037, 896, 818, 798, 736. **HRMS** (ESI+) calculated for $C_{21}H_{25}N_2O$ $[M+H]^+$ 321.19614, found 321.19628.

4-(4-Morpholinotetrahydro-2H-pyran-4-yl)benzonitrile (12u)

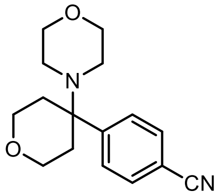

The title compound was synthesised according to General Procedure A, using 4-oxotetrahydropyran (55 uL, 0.6 mmol, 3.0 equiv), morpholine (52 µL, 0.6 mmol, 3.0 equiv) and 1,4-DCB (25.6 mg, 0.2 mmol, 1.0 equiv). The mixture of ketone, amine and base was heated to 80 °C for 30 min before the remaining reagents were added. Irradiation time 2 h. Purification by flash column chromatography on silica gel (0 – 50% PET:EtOAc) afforded the title compound as a colourless oil (29 mg, 0.106 mmol, 53%). **^{1}H NMR** (400 MHz, $CDCl_3$) δ 7.70 – 7.66 (m, 2H), 7.37 – 7.32 (m, 2H), 3.95 (ddd, *J* = 10.9, 7.3, 3.2 Hz, 2H), 3.67 – 3.62 (m, 4H), 3.52 (ddd, *J* = 12.4, 8.2, 4.1 Hz, 2H), 2.31 – 2.19 (m, 6H), 2.12 – 2.05 (m, 2H). **^{13}C NMR** (101 MHz, $CDCl_3$) δ 144.2, 132.0, 127.7, 118.8, 111.1, 67.7, 63.8, 59.2, 45.9, 33.2. **IR** (film) ν_{max}/cm^{-1} 2953, 2922, 2853, 2227 (C≡N), 1605, 1505, 1458, 1390, 1357, 1304, 1269, 1180, 1139, 1116, 1068, 1043, 1021, 982, 921, 909, 868, 838, 810, 741, 686, 584, 567. **HRMS** (ESI+) calculated for $C_{16}H_{21}N_2O_2$ $[M+H]^+$ 273.15975, found 273.16002.

***Tert*-butyl 3-(4-cyanophenyl)-3-morpholinopyrrolidine-1-carboxylate (12v)**

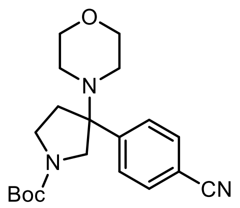

The title compound was synthesised according to General Procedure A, using 1-*N*-boc-3-pyrrolidinone (111 mg, 0.6 mmol, 3.0 equiv), morpholine (52 μL, 0.6 mmol, 3.0 equiv) and 1,4-DCB (25.6 mg, 0.2 mmol, 1.0 equiv). The mixture of ketone, amine and base was heated to 80 °C for 30 min before the remaining reagents were added. Irradiation time 3 h. Purification by flash column chromatography on silica gel (0 – 50% PET:EtOAc) afforded the title compound as a colourless oil (23 mg, 0.064 mmol, 32%). **^{1}H NMR** (700 MHz, $CDCl_3$) δ 7.67 – 7.65 (m, 2H), 7.45 – 7.41 (m, 2H), 3.80 (m, 1H), 3.76 – 3.69 (m, 1H), 3.66 – 3.62 (m, 4H), 3.56 – 3.45 (m, 1H), 3.20 – 3.08 (m, 1H), 2.47 – 2.35 (m, 5H), 2.13 – 2.07 (m, 1H), 1.46 (s, 9H). Peaks duplicated because of rotamers. **^{13}C NMR** (176 MHz, $CDCl_3$) δ 154.8 (R_{major}), 154.5 (R_{minor}), 145.3 (R_{minor}), 144.9 (R_{major}), 132.2 (R_{minor}), 132.2 (R_{major}), 128.1 (R_{major}), 128.0 (R_{minor}), 118.7 (R_{major}), 118.7 (R_{minor}), 111.6 (R_{minor}), 111.5 (R_{major}), 80.0 (R_{minor}), 79.8 (R_{major}), 70.3 (R_{minor}), 69.5 (R_{major}), 67.6 (R_{major}), 67.5 (R_{minor}), 51.7 (R_{minor}), 51.3 (R_{major}), 47.9 ($R_{major + minor}$), 44.3 (R_{major}), 43.9 (R_{minor}), 33.8 (R_{major}), 32.9 (R_{minor}), 28.7 (R_{minor}), 28.6 (R_{major}). **IR** (film) ν_{max}/cm^{-1} 2968, 2890, 2227 (C≡N), 1690, 1607, 1477, 1453, 1404, 1366, 1268, 1167, 1137, 1117, 1024, 959, 928, 869, 772, 736, 569. **HRMS** (ESI+) calculated for $C_{20}H_{28}N_3O_3$ $[M+H]^+$ 358.21252, found 358.21276.

2-(1-Morpholinobutyl)benzonitrile (12w)

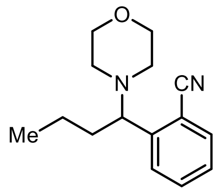

The title compound was synthesised according to General Procedure A, using butyraldehyde (distilled prior to use - 54 uL, 0.6 mmol, 3.0 equiv), morpholine (52 μL, 0.6 mmol, 3.0 equiv) and 1,2-DCB (25.6 mg, 0.2 mmol, 1.0 equiv). Irradiation time 2 h. Purification by flash column chromatography on silica gel (0–20% PET:(PET:Et_2O 90:10 + 3% Et_3N) afforded the title compound as a pale yellow oil (25 mg, 0.102 mmol, 51%). **^{1}H NMR** (400 MHz, $CDCl_3$) δ 7.65 – 7.61 (m, 1H), 7.59 – 7.53 (m, 1H), 7.53 – 7.48 (m, 1H), 7.36 – 7.30 (m, 1H), 3.76 (dd, *J* = 9.3, 4.7 Hz, 1H), 3.69 – 3.63 (m, 4H), 2.61 – 2.52 (m, 2H), 2.39 – 2.32 (m, 2H), 1.97 – 1.87 (m, 1H), 1.74 – 1.65 (m, 1H), 1.25 – 1.13 (m, 1H), 1.08 – 0.96 (m, 1H), 0.86 (t, *J* = 7.2 Hz, 3H). **^{13}C NMR** (101 MHz, CDCl3) δ 145.9, 133.1, 132.7, 128.5, 127.6, 118.3, 113.7, 67.5, 67.3, 51.1, 34.3, 19.1, 14.3. **IR** (film) ν_{max}/cm^{-1} 2957, 2854, 2810, 2222 (C≡N), 1598, 1482, 1448, 1352, 1269, 1208, 1117, 1070, 1017, 993, 979, 924, 873, 801, 764, 547. **HRMS** (ESI+) calculated for $C_{15}H_{21}N_2O$ $[M+H]^+$ 245.16484, found 245.16540.

Methyl 4-(cyclohexyl(morpholino)methyl)benzoate (12x)

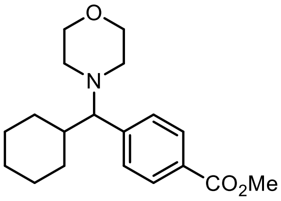

The title compound was synthesised according to General Procedure B, using cyclohexanecarboxaldehyde (distilled prior to use - 73 uL, 0.6 mmol, 3.0 equiv), morpholine (52 μL, 0.6 mmol, 3.0 equiv) and methyl 4-cyanobenzoate (32 mg, 0.2 mmol, 1.0 equiv). Irradiation time 2 h. Purification by flash column chromatography on silica gel (0 – 20% PET:EtOAc) afforded the title compound as a colourless oil (24 mg, 0.076 mmol, 38%). **^{1}H NMR** (500 MHz, $CDCl_3$) δ 7.99 – 7.95 (m, 2H), 7.26 – 7.17 (m, 2H), 3.91 (s, 3H), 3.69 – 3.62 (m, 4H), 3.13 (d, J = 7.7 Hz, 1H), 2.38 – 2.26 (m, 4H), 1.96 – 1.87 (m, 2H), 1.76 – 1.69 (m, 1H), 1.65 – 1.57 (m, 2H), 1.54 – 1.47 (m, 1H), 1.29 – 1.25 (m, 1H), 1.21 – 1.11 (m, 1H), 1.10 – 0.98 (m, 1H), 0.83 – 0.70 (m, 2H). **^{13}C NMR** (101 MHz, $CDCl_3$) δ 167.2, 143.7, 129.5, 129.1, 129.0, 75.4, 67.5, 52.2, 50.7, 37.7, 31.2, 28.8, 26.9, 26.6, 26.3. **IR** (film) ν_{max}/cm^{-1} 2924, 2850, 1722, 1610, 1435, 1416, 1281, 1183, 1116, 1006, 967, 922, 871, 812, 783, 763, 720, 549. **HRMS** (ESI+) calculated for $C_{19}H_{28}NO_3$ $[M+H]^+$ 318.20637, found 318.20672.

4-(Cyclohexyl(4-(phenylsulfonyl)phenyl)methyl)morpholine (12y)

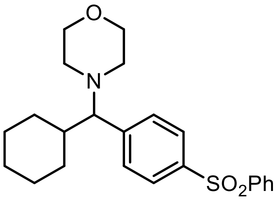

The title compound was synthesised according to General Procedure B, using cyclohexanecarboxaldehyde (distilled prior to use - 73 uL, 0.6 mmol, 3.0 equiv), morpholine (52 μL, 0.6 mmol, 3.0 equiv) and 4-(phenylsulfonyl)benzonitrile (49 mg, 0.2 mmol, 1.0 equiv). Irradiation time 15 min. Purification by flash column chromatography on silica gel (0 – 60% PET:EtOAc) followed by SCX afforded the title compound as a white solid (44 mg, 0.110 mmol, 55%). **^{1}H NMR** (400 MHz, $CDCl_3$) δ 7.99 – 7.96 (m, 2H), 7.89 – 7.85 (m, 2H), 7.61 – 7.56 (m, 1H), 7.55 – 7.50 (m, 2H), 7.31 – 7.27 (m, 2H), 3.67 – 3.60 (m, 4H), 3.12 (d, J = 7.6 Hz, 1H), 2.33 – 2.23 (m, 4H), 1.95 – 1.83 (m, 2H), 1.76 – 1.69 (m, 1H), 1.63 – 1.57 (m, 2H), 1.49 – 1.43 (m, 1H), 1.25 – 0.99 (m, 3H), 0.80 – 0.62 (m, 2H). **^{13}C NMR** (101 MHz, $CDCl_3$) δ 144.3, 141.7, 140.1, 133.2, 130.1, 129.3, 127.7, 127.0, 75.0, 67.2, 50.5, 37.5, 31.0, 28.5, 26.6, 26.4, 26.1. **IR** (film) ν_{max}/cm^{-1} 2925, 2850, 1594, 1447, 1409, 1318, 1306, 1155, 1116, 1107, 1071, 1005, 923, 894, 866, 751, 729, 720, 694, 653, 640, 593, 569. **HRMS** (ESI+) calculated for $C_{23}H_{30}NO_3S$ $[M+H]^+$ 400.19409, found 400.19443.

Diethyl (4-(cyclohexyl(morpholino)methyl)phenyl)phosphonate (12z)

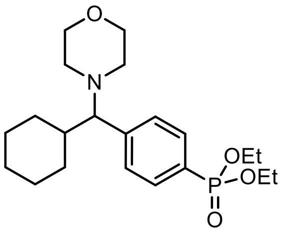

The title compound was synthesised according to General Procedure A, using cyclohexanecarboxaldehyde (distilled prior to use - 73 uL, 0.6 mmol, 3.0 equiv), morpholine (52 μL, 0.6 mmol, 3.0 equiv) and diethyl (4-cyanophenyl)phosphonate (48 mg, 0.2 mmol, 1.0 equiv). Irradiation time 2.5 h. Purification by flash column chromatography on silica gel (0 – 20% PET:EtOAc) afforded the title compound as a colourless oil (23 mg, 0.058 mmol, 29%). **^{1}H NMR** (400 MHz, $CDCl_3$) δ 7.77 – 7.69 (m, 2H), 7.25 – 7.21 (m, 2H), 4.21 – 4.03 (m, 4H), 3.69 – 3.57 (m, 4H), 3.11 (d, *J* = 7.9 Hz, 1H), 2.37 – 2.26 (m, 4H), 1.95 – 1.86 (m, 2H), 1.78 – 1.68 (m, 1H), 1.68 – 1.58 (m, 2H), 1.58 – 1.43 (m, 1H), 1.33 (t, *J* = 7.0 Hz, 6H), 1.28 – 1.00 (m, 3H), 0.86 – 0.65 (m, 2H). **^{13}C NMR** (101 MHz, $CDCl_3$) δ 142.9 (d, *J* = 3.4 Hz), 131.2 (d, *J* = 10.3 Hz), 129.5 (d, *J* = 15.2 Hz), 126.9 (d, *J* = 189.7 Hz), 75.4, 67.4, 62.2 (d, *J* = 5.6 Hz), 50.6, 37.6, 31.2, 28.9, 26.8, 26.6, 26.3, 16.5 (d, *J* = 6.4 Hz). **IR** (film) ν_{max}/cm^{-1} 2924, 2849, 1603, 1448, 1392, 1247, 1164, 1117, 1052, 1020, 960, 894, 876, 865, 782, 762, 700, 654, 571, 553. **HRMS** (ESI+) calculated for $C_{21}H_{35}NO_4P$ [M+H]$^+$ 396.22982, found 396.23027.

4-(Cyclohexyl(pyridin-4-yl)methyl)morpholine (12aa)

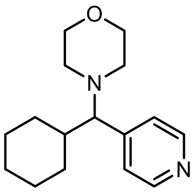

The title compound was synthesised according to General Procedure A, using cyclohexanecarboxaldehyde (distilled prior to use - 73 uL, 0.6 mmol, 3.0 equiv), morpholine (52 μL, 0.6 mmol, 3.0 equiv) and 4-pyridinecarbonitrile (23 mg, 0.2 mmol, 1.0 equiv). Irradiation time 2 h. Purification by flash column chromatography on silica gel (0 – 30% PET:EtOAc) afforded the title compound as a pale yellow oil (32 mg, 0.123 mmol, 62%). **^{1}H NMR** (500 MHz, $CDCl_3$) δ 8.61 – 8.43 (m, 2H), 7.10 – 7.05 (m, 2H), 3.70 – 3.62 (m, 4H), 3.06 (d, *J* = 7.7 Hz, 1H), 2.38 – 2.27 (m, 4H), 1.93 – 1.86 (m, 2H), 1.78 – 1.70 (m, 1H), 1.66 – 1.59 (m, 2H), 1.54 – 1.48 (m, 1H), 1.28 – 1.23 (m, 1H), 1.21 – 1.10 (m, 1H), 1.09 – 0.99 (m, 1H), 0.84 – 0.70 (m, 2H). **^{13}C NMR** (126 MHz, $CDCl_3$) δ 149.5, 147.0, 124.7, 74.8, 67.4, 50.7, 37.3, 31.0, 28.7, 26.8, 26.5, 26.2. **IR** (film) ν_{max}/cm^{-1} 2925, 2851, 2813, 1690, 1597, 1557, 1448, 1412, 1377, 1348, 1312, 1289, 1265, 1218, 1117, 1069, 1005, 993, 923, 895, 875, 865, 838, 827, 680, 597, 553. **HRMS** (ESI+) calculated for $C_{16}H_{25}N_2O$ [M+H]$^+$ 261.19614, found 261.19649.

4-(Cyclohexyl(2,6-dimethylpyridin-4-yl)methyl)morpholine (12ab)

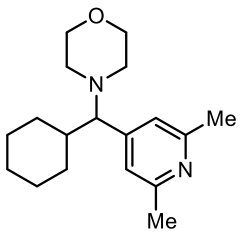

The title compound was synthesised according to General Procedure A, using cyclohexanecarboxaldehyde (distilled prior to use - 73 uL, 0.6 mmol, 3.0 equiv), morpholine (52 μL, 0.6 mmol, 3.0 equiv) and 2,6-dimethylisonicotinonitrile (26 mg, 0.2 mmol, 1.0 equiv). Irradiation time 2 h. Purification by flash column chromatography on silica gel (0 – 20% PET:EtOAc) afforded the title compound as a pale yellow oil (31 mg, 0.107 mmol, 54%). **^{1}H NMR** (400 MHz, $CDCl_3$) δ 6.73 (s, 2H), 3.70 – 3.62 (m, 4H), 2.97 (d, *J* = 7.9 Hz, 1H), 2.52 (s, 6H), 2.39 – 2.24 (m, 4H), 1.93 – 1.81 (m, 2H), 1.78 – 1.70 (m, 1H), 1.68 – 1.58 (m, 2H), 1.53 – 1.44 (m, 1H), 1.27 – 1.02 (m, 3H), 0.88 – 0.66 (m, 2H). **^{13}C NMR** (101 MHz, $CDCl_3$) δ 157.1, 147.8, 121.3, 74.9, 67.4, 50.7, 37.2, 31.0, 28.9, 26.8, 26.5, 26.3, 24.6. **IR** (film) ν_{max}/cm^{-1} 2923, 2850, 2814, 1687, 1603, 1564, 1448, 1418, 1381, 1347, 1327, 1288, 1266, 1210, 1117, 1069, 1032, 1006, 924, 893, 873, 754, 712. **HRMS** (ESI+) calculated for $C_{18}H_{29}N_2O$ $[M+H]^+$ 289.22744, found 289.22778.

4-(Cyclohexyl(3-methylpyridin-4-yl)methyl)morpholine (12ac)

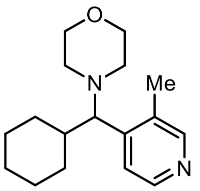

The title compound was synthesised according to General Procedure A, using cyclohexanecarboxaldehyde (distilled prior to use - 73 uL, 0.6 mmol, 3.0 equiv), morpholine (52 μL, 0.6 mmol, 3.0 equiv) and 3-methylisonicotinonitrile (24 mg, 0.2 mmol, 1.0 equiv). Irradiation time 2 h. Purification by flash column chromatography on silica gel (0–100% PET:(PET:Et_2O 90:10 + 3% Et_3N) afforded the title compound as a pale yellow oil (28 mg, 0.102 mmol, 51%). **^{1}H NMR** (400 MHz, $CDCl_3$) δ 8.41 – 8.37 (m, 2H), 7.16 (d, *J* = 5.1 Hz, 1H), 3.69 – 3.59 (m, 4H), 3.49 (d, *J* = 7.8 Hz, 1H), 2.43 – 2.29 (m, 7H), 1.98 – 1.86 (m, 2H), 1.78 – 1.69 (m, 1H), 1.66 – 1.59 (m, 2H), 1.52 – 1.43 (m, 1H), 1.26 – 1.01 (m, 3H), 0.88 – 0.76 (m, 2H). **^{13}C NMR** (101 MHz, $CDCl_3$) δ 151.5, 146.9, 146.1, 133.1, 123.0, 68.7, 67.4, 50.7, 38.6, 30.7, 28.6, 26.8, 26.5, 26.3, 17.5. **IR** (film) ν_{max}/cm^{-1} 2926, 2850, 2814, 1590, 1449, 1402, 1289, 1266, 1212, 1139, 1118, 1070, 1033, 1006, 923, 895, 876, 866, 795, 681. **HRMS** (ESI+) calculated for $C_{17}H_{27}N_2O$ $[M+H]^+$ 275.21179, found 275.21215.

(4-(Cyclohexyl(morpholino)methyl)pyridin-2-yl)methanol (12ad)

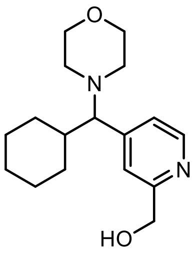

The title compound was synthesised according to General Procedure A, using cyclohexanecarboxaldehyde (distilled prior to use - 73 uL, 0.6 mmol, 3.0 equiv), morpholine (52 μL, 0.6 mmol, 3.0 equiv) and 2-(hydroxymethyl)isonicotinonitrile (27 mg, 0.2 mmol, 1.0 equiv). Irradiation time 2 h. Purification by flash column chromatography on silica gel (0–100% PET:(PET:Et_2O 90:10 + 3% Et_3N) afforded the title compound as a pale yellow oil (19 mg, 0.065 mmol, 33%). **^{1}H NMR** (700 MHz, $CDCl_3$) δ 8.49 – 8.45 (m, 1H), 7.03 – 6.99 (m, 2H), 4.76 (s, 2H), 3.79 (s, 1H), 3.69 – 3.62 (m, 4H), 3.07 (d, J = 7.7 Hz, 1H), 2.36 – 2.29 (m, 4H), 1.93 – 1.87 (m, 2H), 1.77 – 1.71 (m, 1H), 1.66 – 1.61 (m, 2H), 1.51 – 1.47 (m, 1H), 1.27 – 1.23 (m, 1H), 1.18 – 1.12 (m, 1H), 1.08 – 0.99 (m, 1H), 0.82 – 0.70 (m, 2H). **^{13}C NMR** (176 MHz, $CDCl_3$) δ 158.8, 148.1, 148.0, 123.5, 121.2, 74.9, 67.4, 64.4, 50.7, 37.3, 31.0, 28.7, 26.8, 26.5, 26.2. **IR** (film) ν_{max}/cm^{-1} 3240, 2923, 2850, 2815, 1603, 1557, 1448, 1413, 1362, 1266, 1139, 1117, 1067, 1033, 1005, 924, 896, 865, 688. **HRMS** (ESI+) calculated for $C_{17}H_{27}N_2O_2$ [M+H]$^+$ 291.20670, found 291.20716.

4-Benzylbenzonitrile (19a)

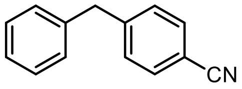

The title compound was synthesised according to General Procedure A, using benzaldehyde (distilled prior to use - 61 uL, 0.6 mmol, 3.0 equiv), morpholine (52 μL, 0.6 mmol, 3.0 equiv) and 1,4-DCB (25.6 mg, 0.2 mmol, 1.0 equiv). Irradiation time 2 h. Purification by flash column chromatography on silica gel (0–10% PET:EtOAc) afforded the title compound as a colourless oil (24 mg, 0.124 mmol, 62%). **^{1}H NMR** (400 MHz, $CDCl_3$) δ 7.60 – 7.54 (m, 2H), 7.32 – 7.24 (m, 5H), 7.19 – 7.14 (m, 2H), 4.03 (s, 2H). **^{13}C NMR** (101 MHz, $CDCl_3$) δ 146.9, 139.5, 132.4, 129.8, 129.1, 128.9, 126.8, 119.1, 110.2, 42.1. **IR** (film) ν_{max}/cm^{-1} 3063, 3028, 2923, 2853, 2227 (C≡N), 1603, 1507, 1495, 1453, 1413, 1251, 1178, 1112, 1074, 1029, 1021, 854, 797, 761, 726, 699, 593, 543. **HRMS** (ESI+) calculated for $C_{14}H_{12}N$ [M+H]$^+$ 194.09643, found 194.09635.

4-(Cyclohex-3-en-1-ylmethyl)benzonitrile (19b)

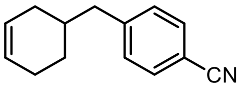

The title compound was synthesised according to General Procedure A, using 1,2,3,6-tetrahydrobenzaldehyde (distilled prior to use - 68 uL, 0.6 mmol, 3.0 equiv), morpholine (52 μL, 0.6 mmol, 3.0 equiv) and 1,4-DCB (25.6 mg, 0.2 mmol, 1.0 equiv). Irradiation time 4 h. Purification by flash column chromatography on silica gel (0–20% PET:EtOAc) afforded the title compound as a colourless oil (16 mg, 0.081 mmol, 41%). **^{1}H NMR** (500 MHz, $CDCl_3$) δ 7.58 – 7.55 (m, 2H), 7.28 – 7.26 (m, 2H), 5.71 – 5.57 (m, 2H), 2.63 (d, *J* = 7.3 Hz, 2H), 2.10 – 1.97 (m, 3H), 1.92 – 1.82 (m, 1H), 1.74 – 1.66 (m, 2H), 1.31 – 1.24 (m, 1H). **^{13}C NMR** (126 MHz, $CDCl_3$) δ 147.0, 132.2, 130.0, 127.2, 126.0, 119.3, 109.8, 43.2, 35.5, 31.6, 28.5, 25.1. **IR** (film) ν_{max}/cm^{-1} 3022, 2918, 2852, 2226 (C≡N), 1679, 1607, 1504, 1451, 1436, 1415, 1252, 1177, 1142, 1113, 1043, 1021, 847, 822, 797, 655, 564. **HRMS** (ESI+) calculated for $C_{14}H_{16}N$ $[M+H]^+$ 198.12773, found 198.12767.

Methyl 4-(4-cyanophenyl)butanoate (19c)

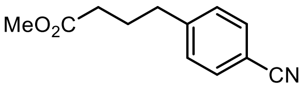

The title compound was synthesised according to General Procedure A, using methyl 4-oxobutanoate (63 uL, 0.6 mmol, 3.0 equiv), morpholine (52 μL, 0.6 mmol, 3.0 equiv) and 1,4-DCB (25.6 mg, 0.2 mmol, 1.0 equiv). Irradiation time 16 h. Purification by flash column chromatography on silica gel (0–20% PET:EtOAc) afforded the title compound as a colourless oil (22 mg, 0.108 mmol, 54%). **^{1}H NMR** (400 MHz, $CDCl_3$) δ 7.59 – 7.55 (m, 2H), 7.30 – 7.27 (m, 2H), 3.67 (s, 3H), 2.74 – 2.68 (m, 2H), 2.33 (t, *J* = 7.3 Hz, 2H), 2.01 – 1.92 (m, 2H). **^{13}C NMR** (101 MHz, $CDCl_3$) δ 173.6, 147.2, 132.4, 129.4, 119.1, 110.1, 51.8, 35.3, 33.3, 26.1. **IR** (film) ν_{max}/cm^{-1} 2922, 2853, 2227 (C≡N), 1733, 1608, 1505, 1437, 1416, 1374, 1252, 1198, 1175, 1148, 1020, 844, 817, 735, 702, 560. **HRMS** (ESI+) calculated for $C_{12}H_{14}NO_2$ $[M+H]^+$ 204.10191, found 204.10186.

Tert-butyl 3-(4-cyanobenzyl)azetidine-1-carboxylate (19d)

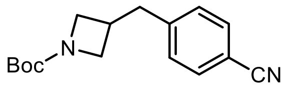

The title compound was synthesised according to General Procedure A, using methyl 1-boc-3-formylazetidine (111 mg, 0.6 mmol, 3.0 equiv), morpholine (52 µL, 0.6 mmol, 3.0 equiv) and 1,4-DCB (25.6 mg, 0.2 mmol, 1.0 equiv). Irradiation time 6 h. Purification by flash column chromatography on silica gel (0–20% PET:EtOAc) afforded the title compound as a colourless oil (16 mg, 0.059 mmol, 30%). **^{1}H NMR** (400 MHz, $CDCl_3$) δ 7.61 – 7.57 (m, 2H), 7.26 – 7.23 (m, 2H), 4.00 (t, J = 8.4 Hz, 2H), 3.63 (dd, J = 8.7, 5.3 Hz, 2H), 2.97 (d, J = 7.9 Hz, 2H), 2.86 – 2.77 (m, 1H), 1.43 (s, 9H). **^{13}C NMR** (101 MHz, $CDCl_3$) δ 156.5, 145.0, 132.6, 129.3, 118.9, 110.6, 79.7, 40.4, 29.6, 28.5. Note, the CH$_2$ of the azetidine are not observed. This is owed to rapid quadrupolar relaxation of these signals. The cross peak corresponding to this carbon environment can be observed in the HSQC and HMBC and is present at δ 54.0. **IR** (film) ν_{max}/cm^{-1} 2964, 2927, 2880, 2227 (C≡N), 1698, 1608, 1478, 1401, 1365, 1255, 1138, 859, 772, 565. **HRMS** (ESI+) calculated for $C_{12}H_{13}N_2O_2$ [M+2H–C_4H_8]$^+$ 217.09715, found 217.09789.

4-(7-Hydroxy-3,7-dimethyloctyl)benzonitrile (19e)

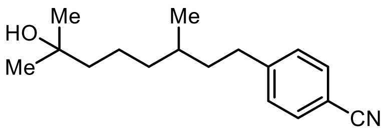

The title compound was synthesised according to General Procedure A, using hydroxycitronellal (112 uL, 0.6 mmol, 3.0 equiv), morpholine (52 µL, 0.6 mmol, 3.0 equiv) and 1,4-DCB (26 mg, 0.2 mmol, 1.0 equiv). Irradiation time 16 h. Purification by flash column chromatography on silica gel (0–20% PET:EtOAc) afforded the title compound as a colourless oil (34 mg, 0.131 mmol, 66%). **^{1}H NMR** (400 MHz, $CDCl_3$) δ 7.58 – 7.54 (m, 2H), 7.30 – 7.27 (m, 2H), 2.76 – 2.58 (m, 2H), 1.67 – 1.58 (m, 1H), 1.48 – 1.30 (m, 7H), 1.23 – 1.17 (m, 8H), 0.97 – 0.92 (m, 3H). **^{13}C NMR** (101 MHz, $CDCl_3$) δ 148.9, 132.3, 129.3, 119.3, 109.6, 71.1, 44.3, 38.5, 37.5, 33.9, 32.6, 29.5, 29.4, 21.8, 19.6. **IR** (film) ν_{max}/cm^{-1} 3402, 2964, 2934, 2867, 2227 (C≡N), 1607, 1505, 1463, 1414, 1377, 1177, 1160, 935, 908, 822, 560. **HRMS** (ESI+) calculated for $C_{17}H_{24}N$ [M+H–H_2O]$^+$ 242.19033, found 242.19039.

4-Benzylpyridine (19f)

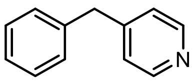

The title compound was synthesised according to General Procedure A, using benzaldehyde (distilled prior to use - 61 uL, 0.6 mmol, 3.0 equiv), morpholine (52 μL, 0.6 mmol, 3.0 equiv) and 4-pyridinecarbonitrile (21 mg, 0.2 mmol, 1.0 equiv). Irradiation time 4 h. Purification by flash column chromatography on silica gel (0–20% PET:EtOAc) followed by SCX afforded the title compound as a colourless oil (17 mg, 0.100 mmol, 50%). **^{1}H NMR** (400 MHz, $CDCl_3$) δ 8.54 – 8.44 (m, 2H), 7.38 – 7.31 (m, 2H), 7.25 – 7.22 (m, 1H), 7.19 – 7.15 (m, 2H), 7.12 – 7.09 (m, 2H), 3.97 (s, 2H). **^{13}C NMR** (101 MHz, $CDCl_3$) δ 150.2, 150.0, 139.0, 129.2, 128.9, 126.8, 124.3, 41.4. NMR data agree with that reported in the literature:

Li, M.; Tian, Y.; Sun, K.; Xu, Z.; Tian, L.; Wang, Y. Electrochemical deoxygenative arylation of aldehydes and ketones. *Chem. Commun*., **2023**, *59*, 5587–5590.

4-(Thiophen-3-ylmethyl)pyridine (19g)

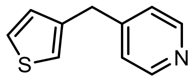

The title compound was synthesised according to General Procedure A, using 3-thiophenecarboxaldehyde (53 uL, 0.6 mmol, 3.0 equiv), morpholine (52 μL, 0.6 mmol, 3.0 equiv) and 4-pyridinecarbonitrile (21 mg, 0.2 mmol, 1.0 equiv). Irradiation time 16 h. Purification by flash column chromatography on silica gel (0–20% PET:EtOAc) afforded the title compound as a colourless oil (11 mg, 0.063 mmol, 32%). **^{1}H NMR** (400 MHz, $CDCl_3$) δ 8.53 – 8.49 (m, 2H), 7.29 (dd, J = 4.9, 2.9 Hz, 1H), 7.14 – 7.09 (m, 2H), 6.99 – 6.96 (m, 1H), 6.89 (dd, J = 4.9, 1.3 Hz, 1H), 3.98 (s, 2H). **^{13}C NMR** (101 MHz, $CDCl_3$) δ 150.1, 149.6, 139.1, 128.4, 126.3, 124.2, 122.2, 35.9. **IR** (film) ν_{max}/cm^{-1} 3070, 2923, 2852, 1659, 1600, 1560, 1496, 1416, 1264, 1218, 1069, 994, 942, 835, 774, 737, 684, 640, 574, 476. **HRMS** (ESI+) calculated for $C_{10}H_{10}NS$ $[M+H]^+$ 176.05285, found 176.05325.

4-(4-(4-(4-Chlorophenyl)-4-hydroxypiperidin-1-yl)-1-(4-fluorophenyl)butyl)benzonitrile (19h)

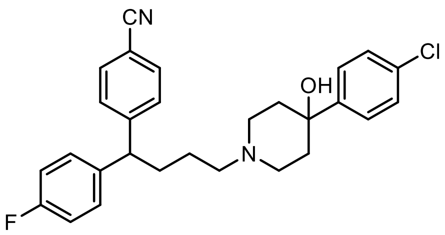

The title compound was synthesised according to General Procedure A, using haloperidol (226 mg, 0.6 mmol, 3.0 equiv), morpholine (52 µL, 0.6 mmol, 3.0 equiv) and 1,4-DCB (26 mg, 0.2 mmol, 1.0 equiv). Irradiation time 16 h. Purification by flash column chromatography on silica gel (0–20% PET:EtOAc) afforded the title compound as a colourless oil (61 mg, 0.132 mmol, 66%). **^{1}H NMR** (400 MHz, $CDCl_3$) δ 7.59 – 7.54 (m, 2H), 7.44 – 7.39 (m, 2H), 7.34 – 7.28 (m, 4H), 7.18 – 7.12 (m, 2H), 7.02 – 6.95 (m, 2H), 3.95 (t, J = 7.8 Hz, 1H), 2.77 – 2.67 (m, 2H), 2.45 – 2.31 (m, 4H), 2.12 – 2.01 (m, 4H), 1.73 – 1.66 (m, 2H), 1.58 – 1.35 (m, 2H). **^{13}C NMR** (101 MHz, $CDCl_3$) δ 161.7 (d, J = 245.6 Hz), 150.5, 147.0, 139.2 (d, J = 3.1 Hz), 132.9, 132.5, 129.3 (d, J = 7.8 Hz), 128.7, 128.5, 126.2, 119.0, 115.7 (d, J = 21.3 Hz), 110.3, 71.2, 58.6, 50.7, 49.6 (α-amino CH$_2$), 49.6 (α-amino CH$_2$), 38.5 (2×CH$_2$ piperidine), 33.5, 25.5. **IR** (film) ν_{max}/cm^{-1} 3321, 2944, 2818, 2227 (C≡N), 1639, 1606, 1508, 1470, 1399, 1378, 1300, 1223, 1176, 1159, 1132, 1095, 1045, 1013, 987, 915, 827, 738, 573, 543, 491, 472. **HRMS** (ESI+) calculated for $C_{28}H_{29}N_2OFCl$ [M+H]$^+$ 463.19470, found 463.19490.

10. NMR Spectra

Diethyl (4-cyanophenyl)phosphonate (11z)

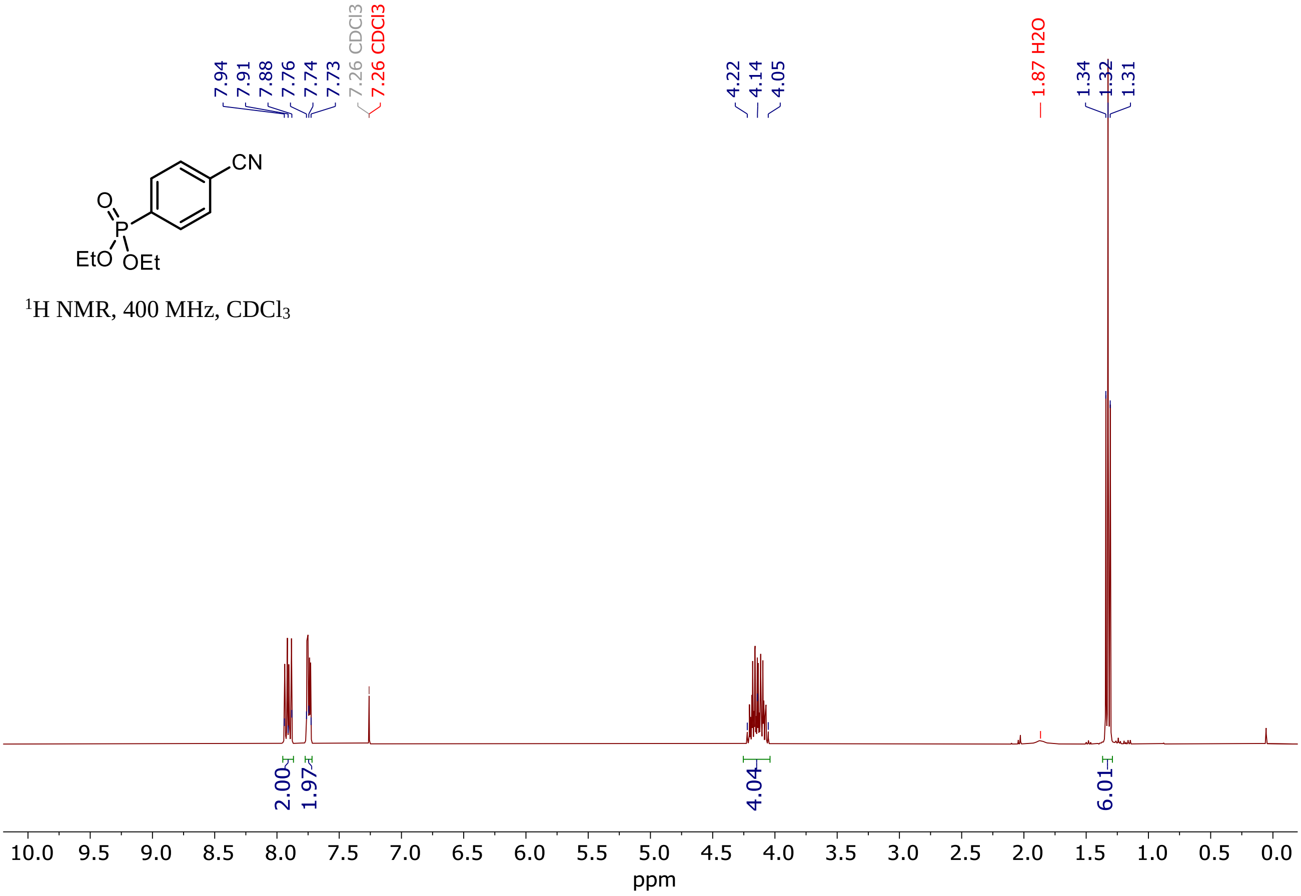

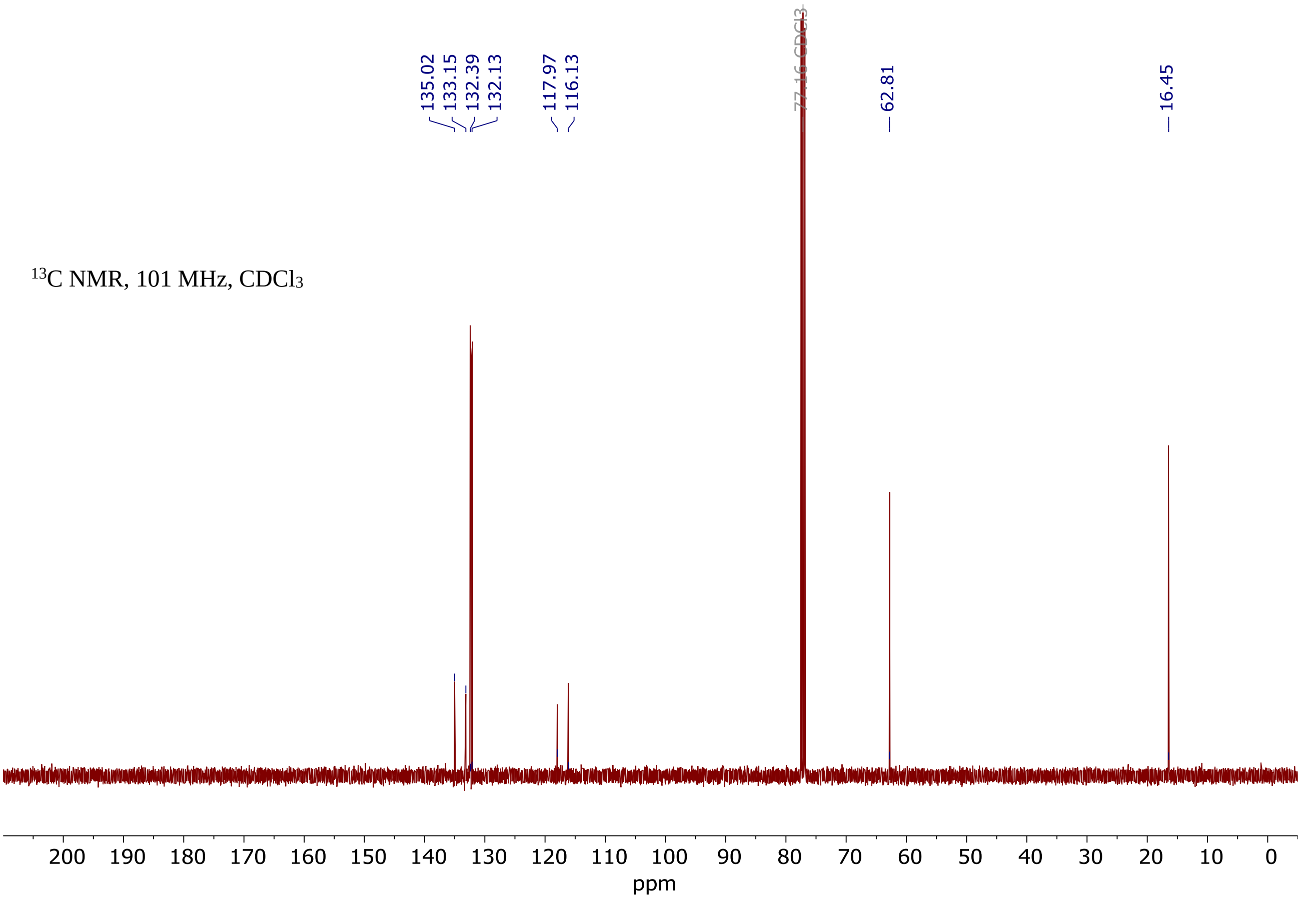

4-(1-(1,1,1,3,3,3-Hexamethyl-2-(trimethylsilyl)trisilan-2-yl)-2-phenylpropan-2-yl)benzonitrile (14)

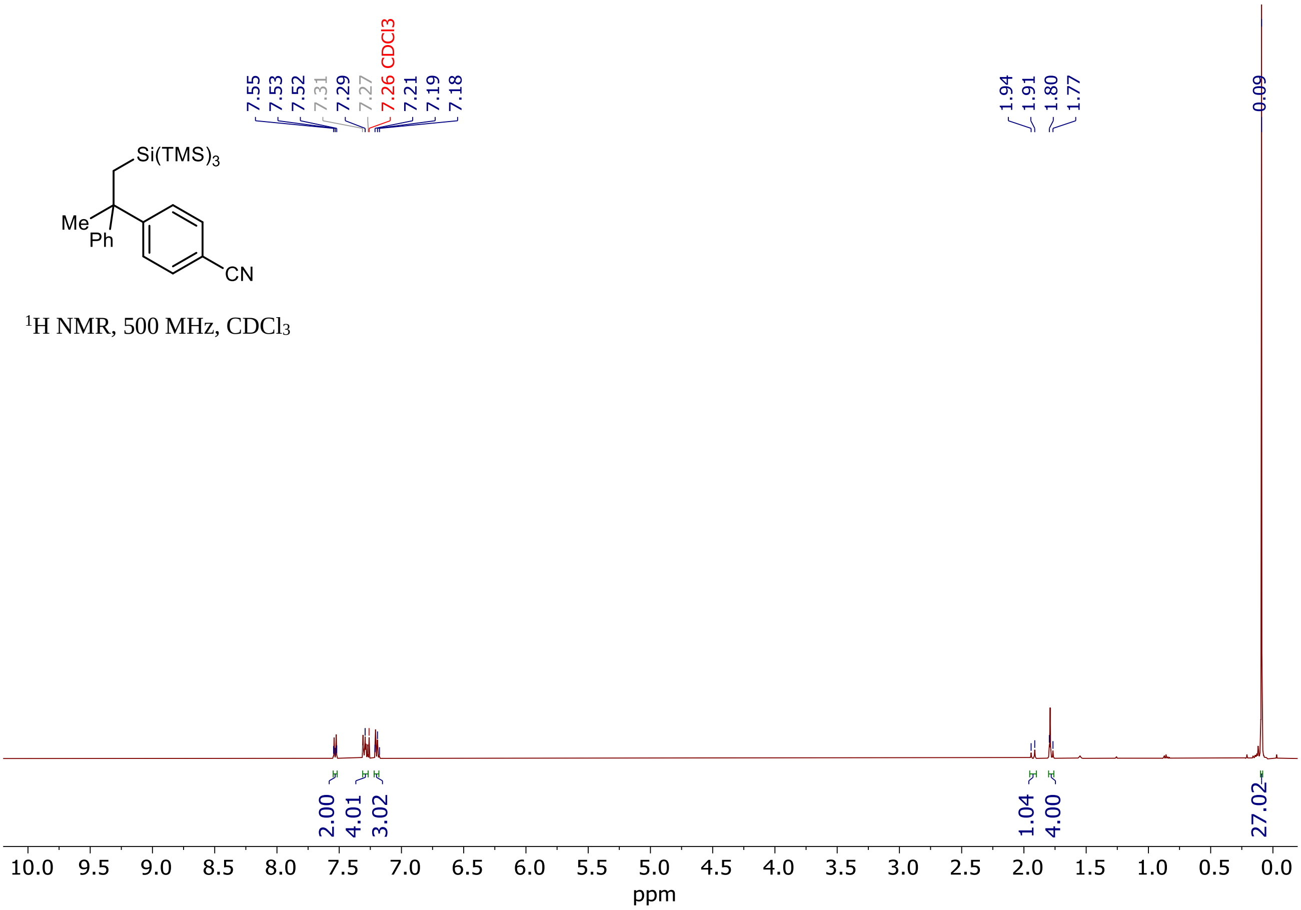

^{1}H NMR, 500 MHz, $CDCl_3$

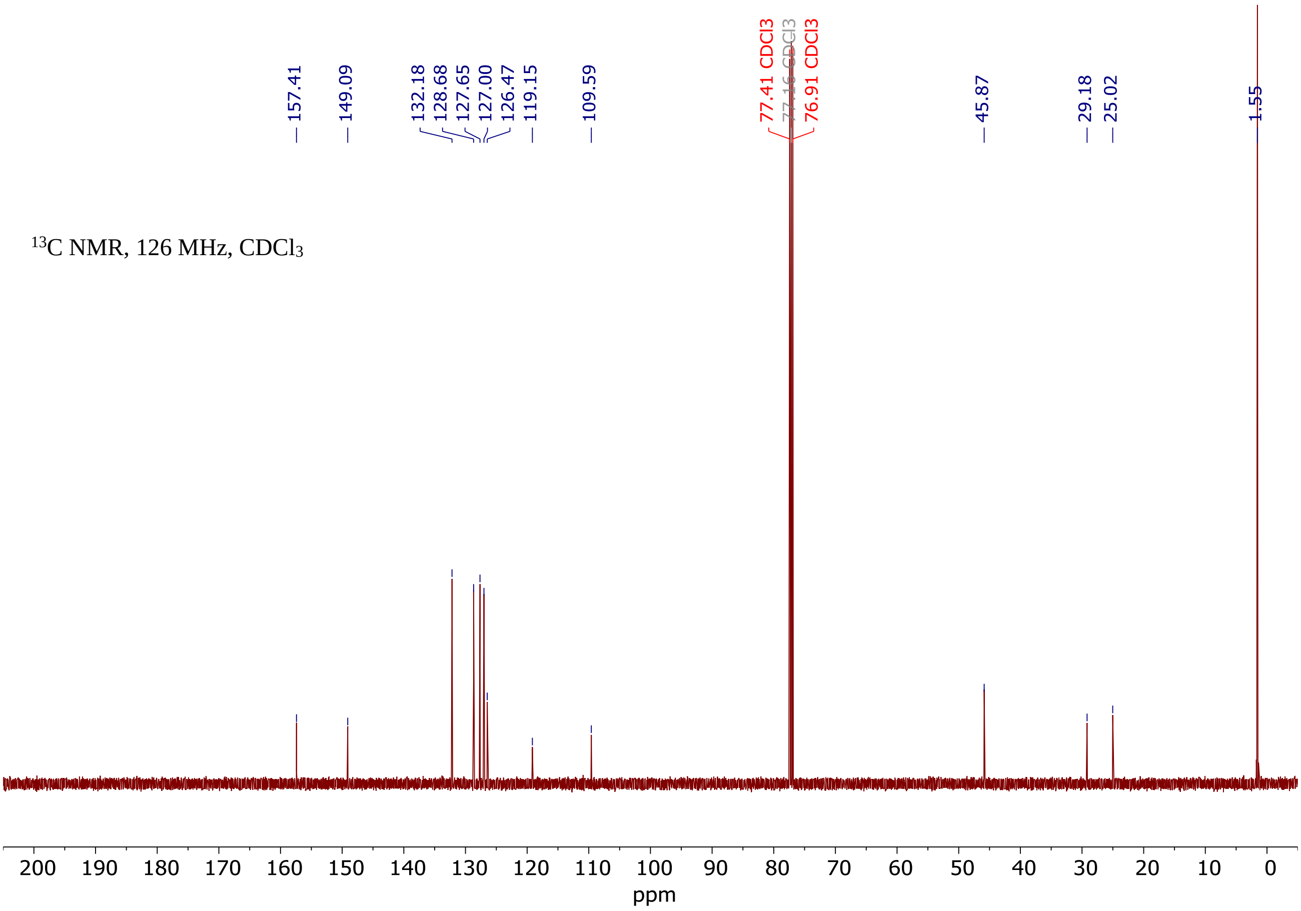

^{13}C NMR, 126 MHz, $CDCl_3$

4-(1-Morpholino-3-phenylpropyl)benzonitrile (12a)

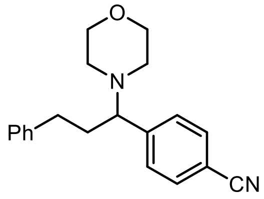

^{1}H NMR, 500 MHz, $CDCl_3$

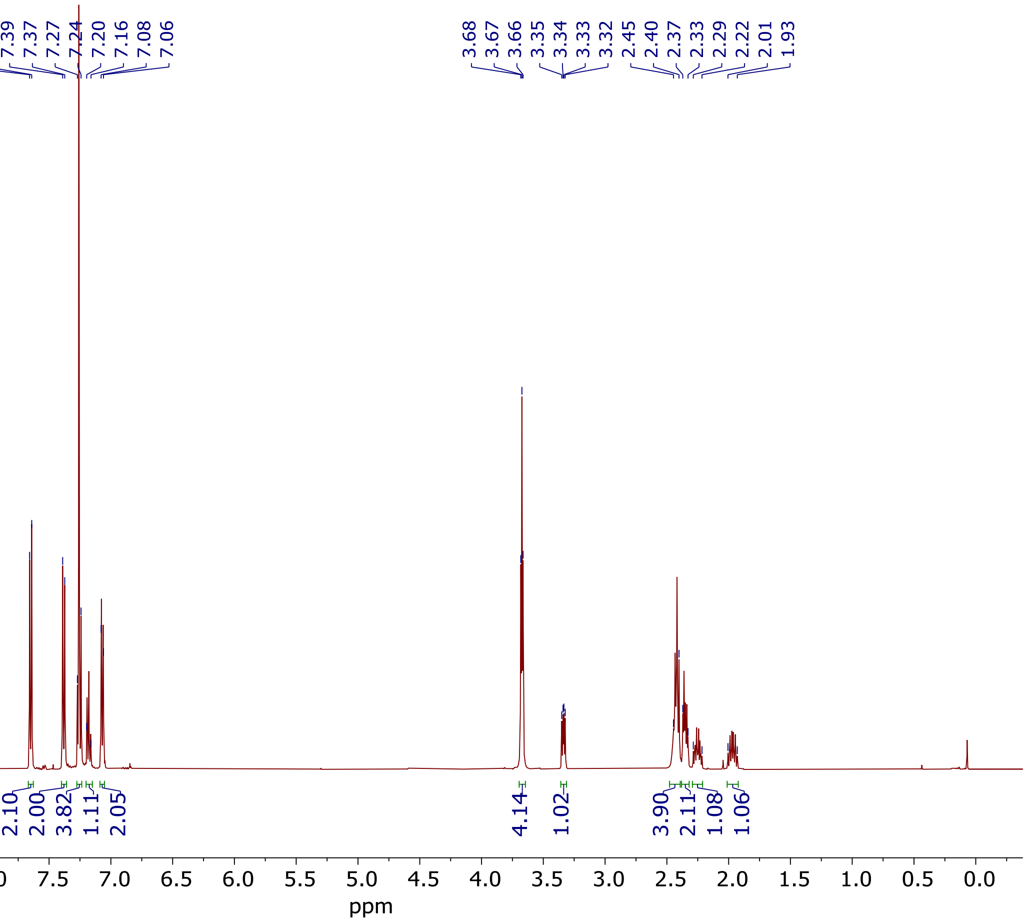

^{13}C NMR, 101 MHz, $CDCl_3$

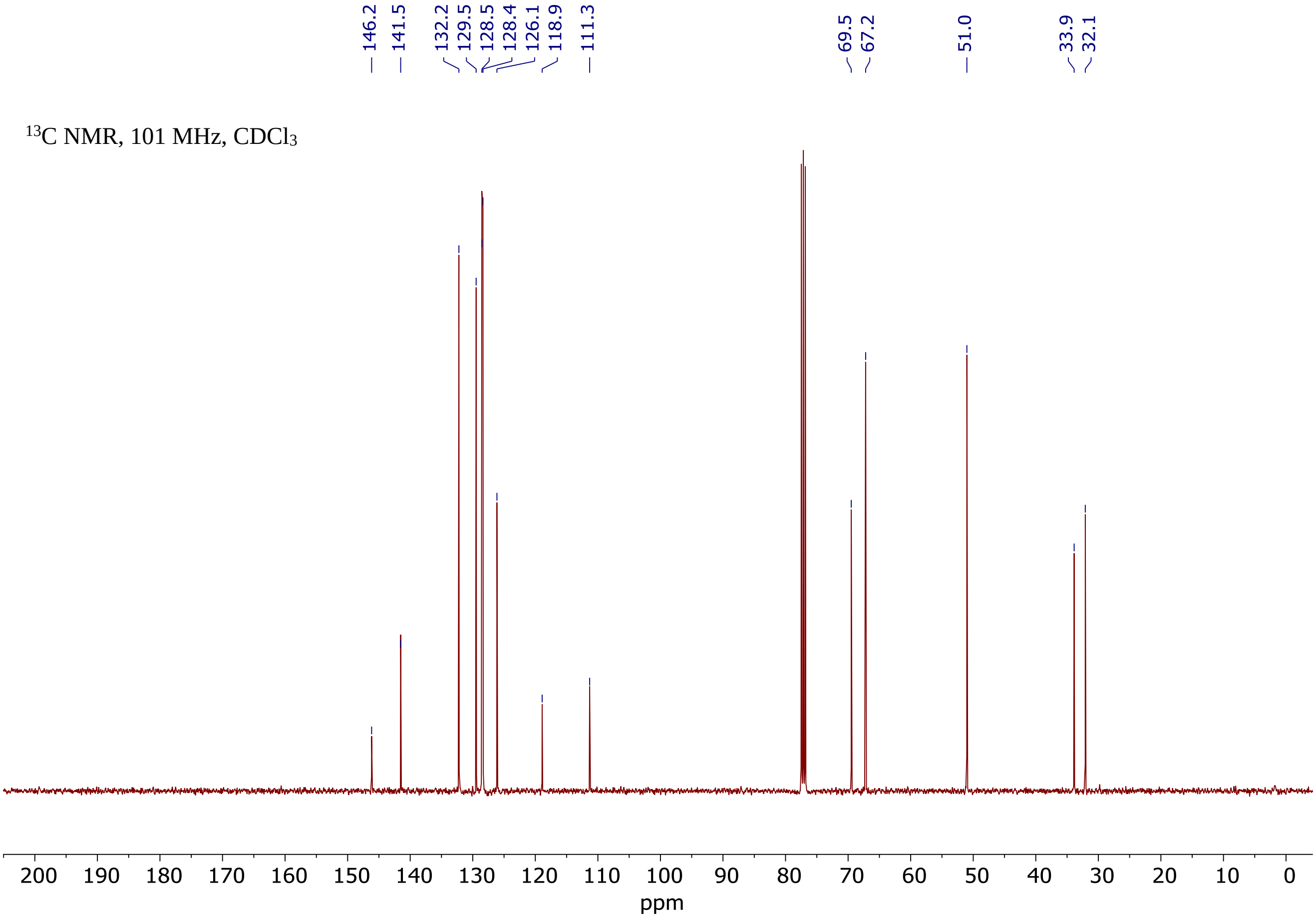

4-(1-Morpholinobutyl)benzonitrile (12b)

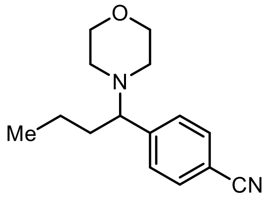

^{1}H NMR, 400 MHz, $CDCl_3$

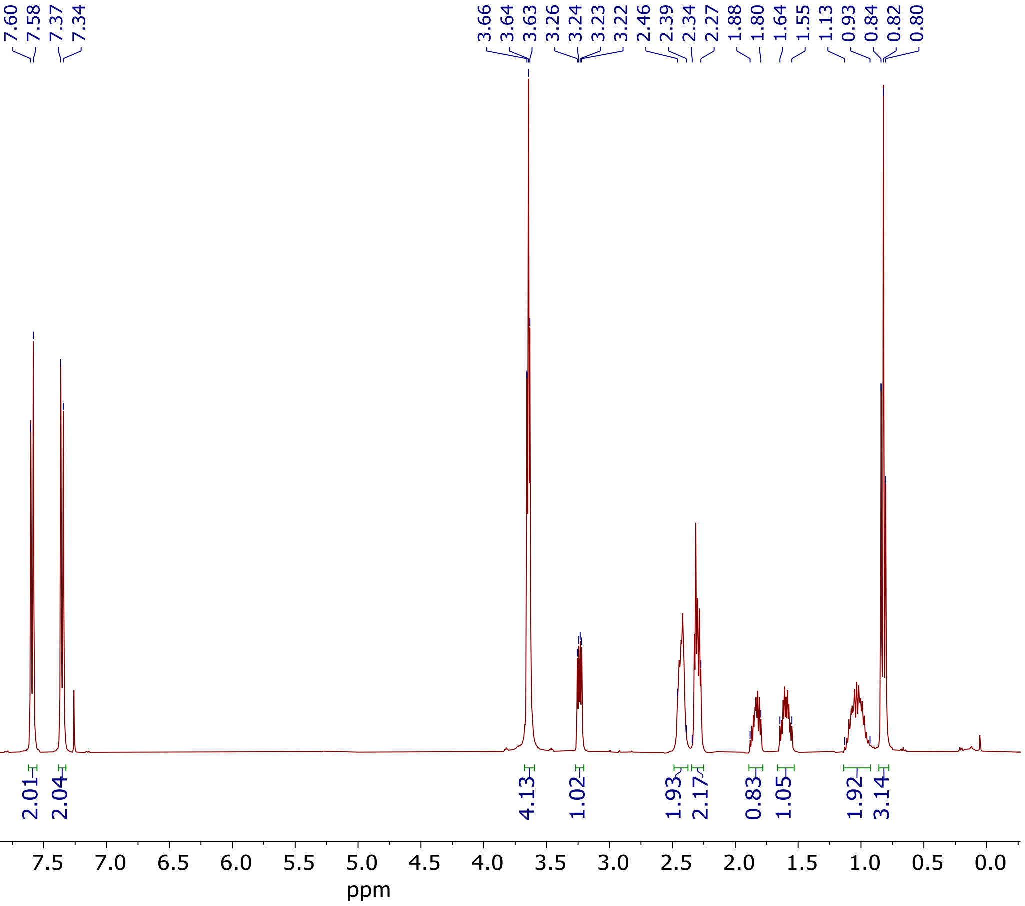

^{13}C NMR, 101 MHz, $CDCl_3$

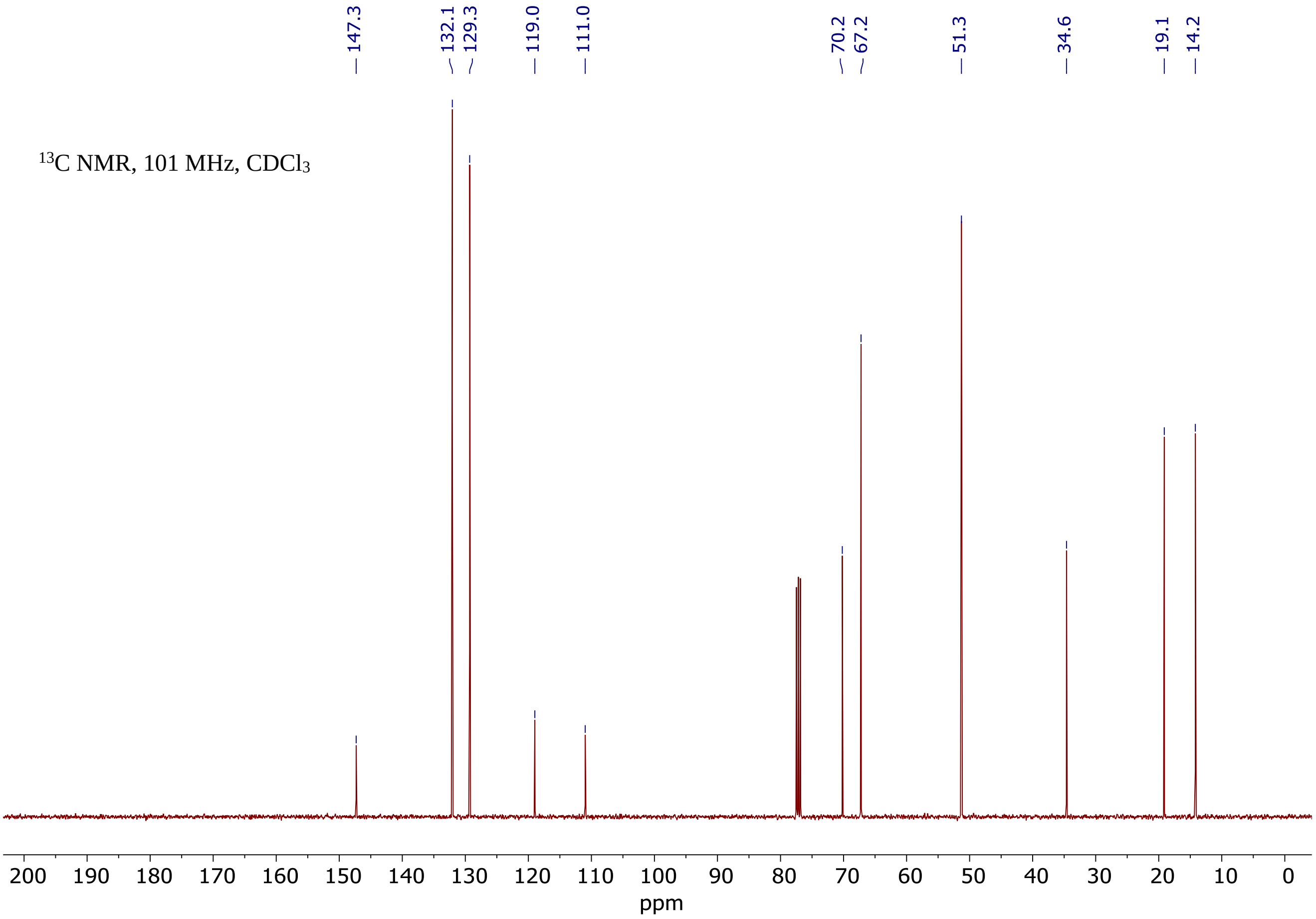

4-(2-Methyl-1-morpholinobutyl)benzonitrile (12c)

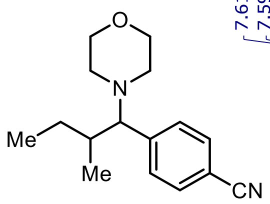

^{1}H NMR, 400 MHz, $CDCl_3$

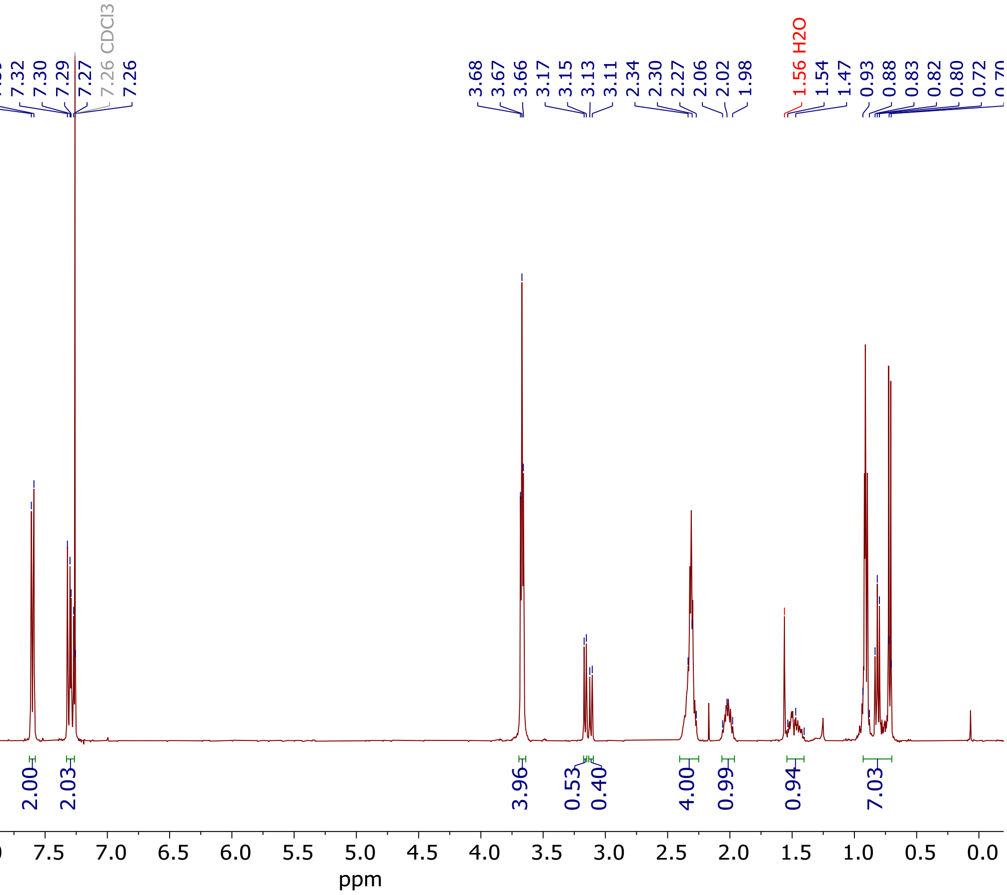

^{13}C NMR, 101 MHz, $CDCl_3$

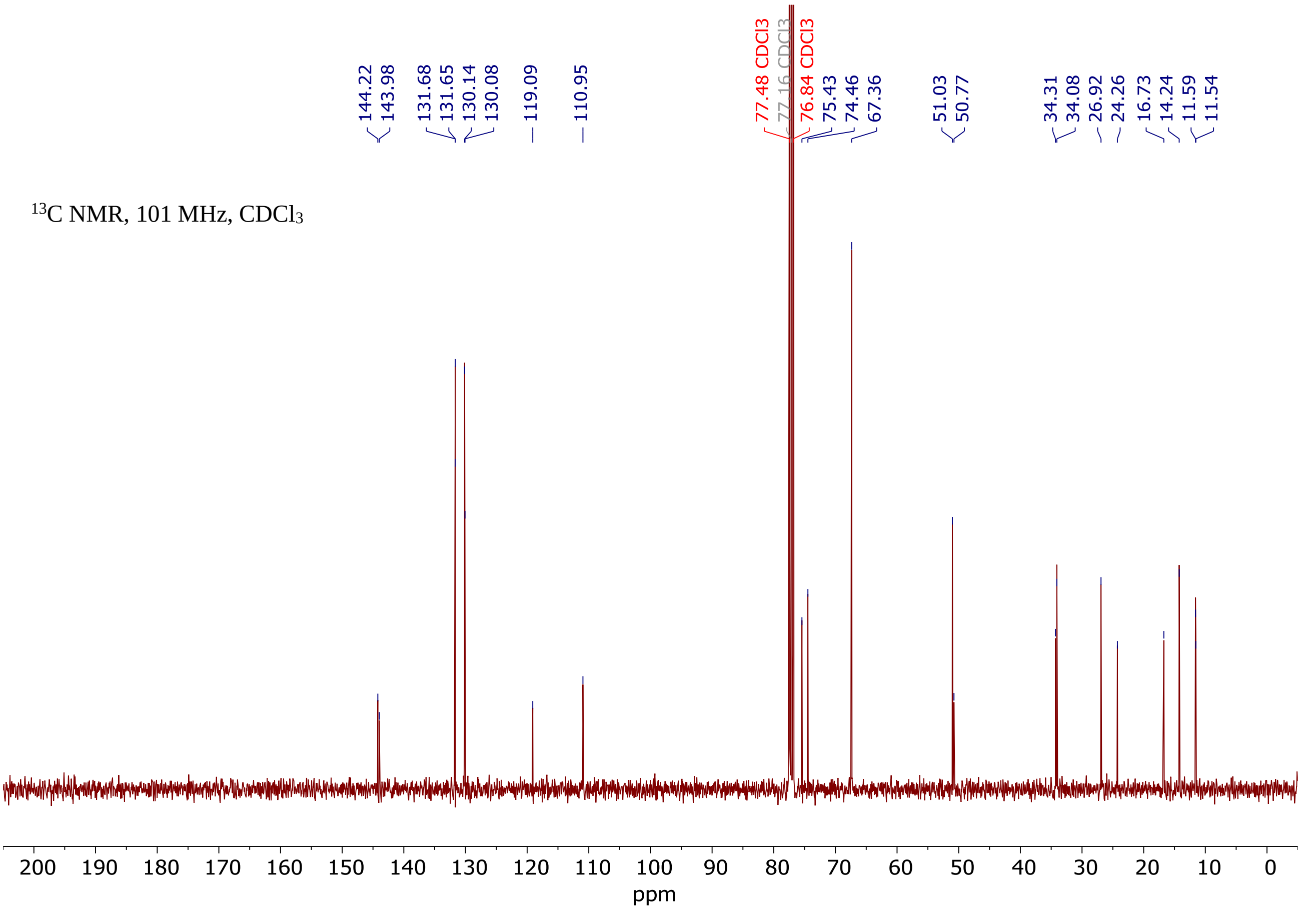

Methyl 4-(4-cyanophenyl)-4-morpholinobutanoate (12d)

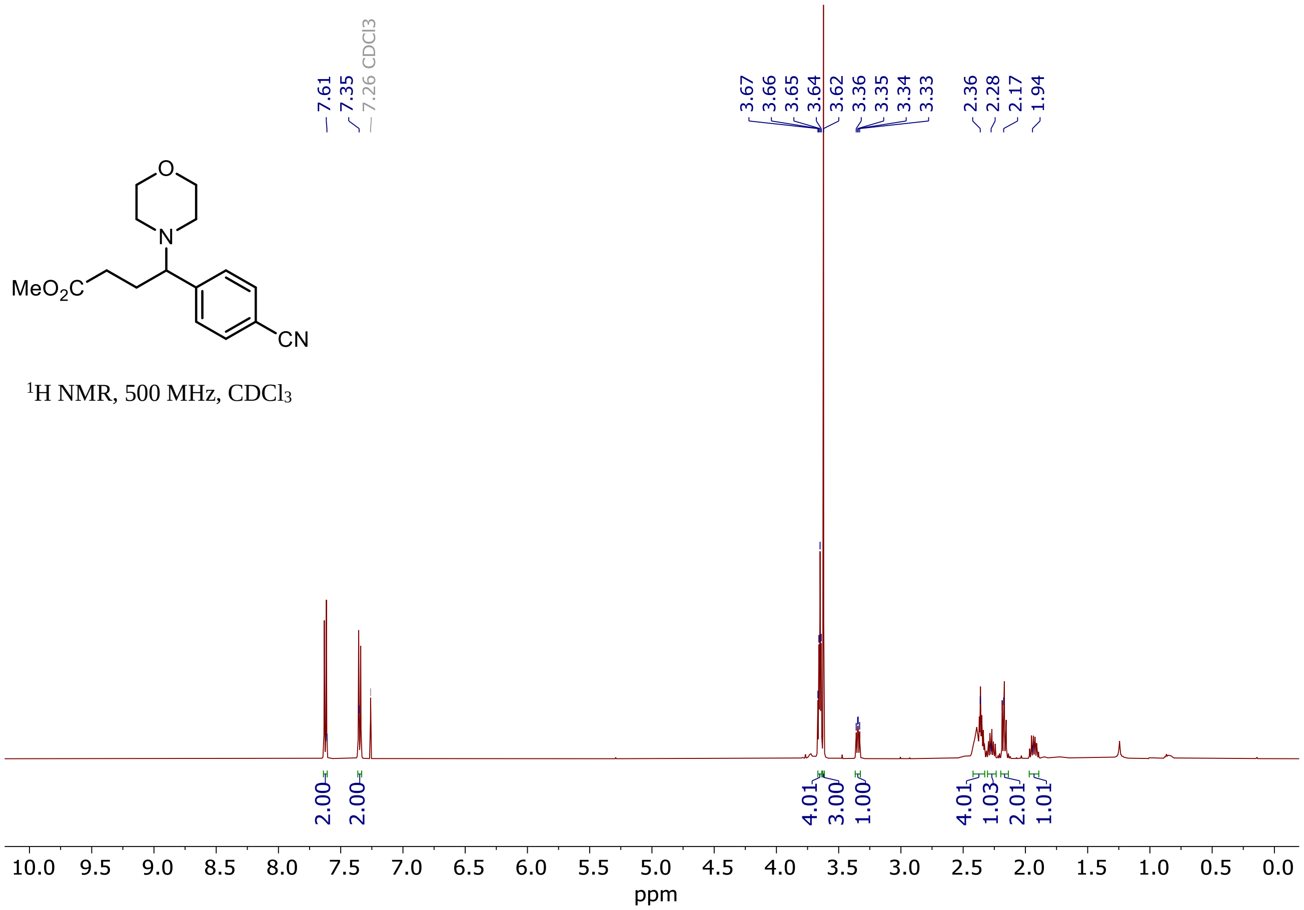

^{1}H NMR, 500 MHz, $CDCl_3$

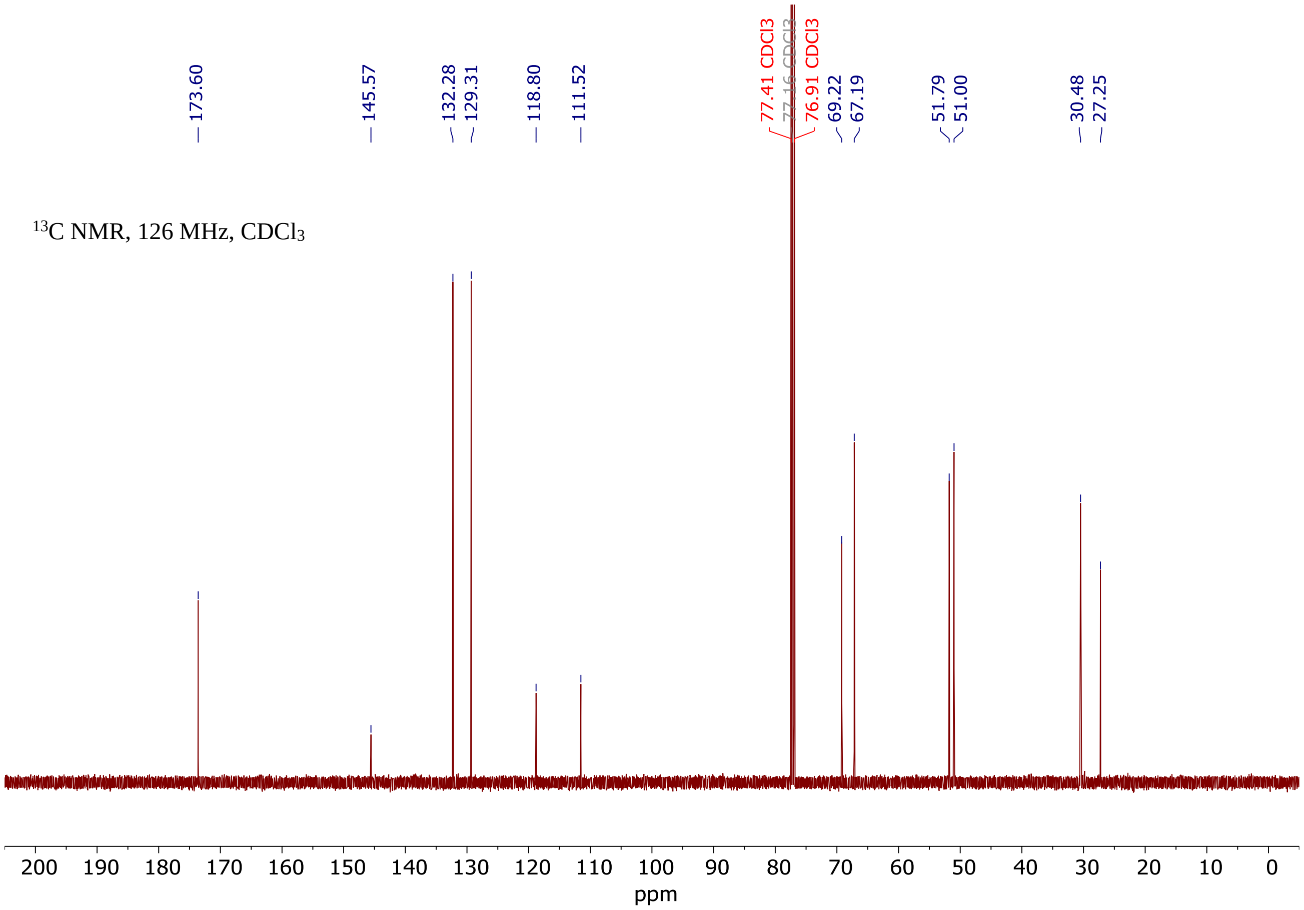

^{13}C NMR, 126 MHz, $CDCl_3$

4-(7-Hydroxy-3,7-dimethyl-1-morpholinooctyl)benzonitrile (12e)

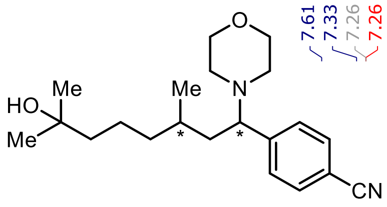

^{1}H NMR, 400 MHz, $CDCl_3$

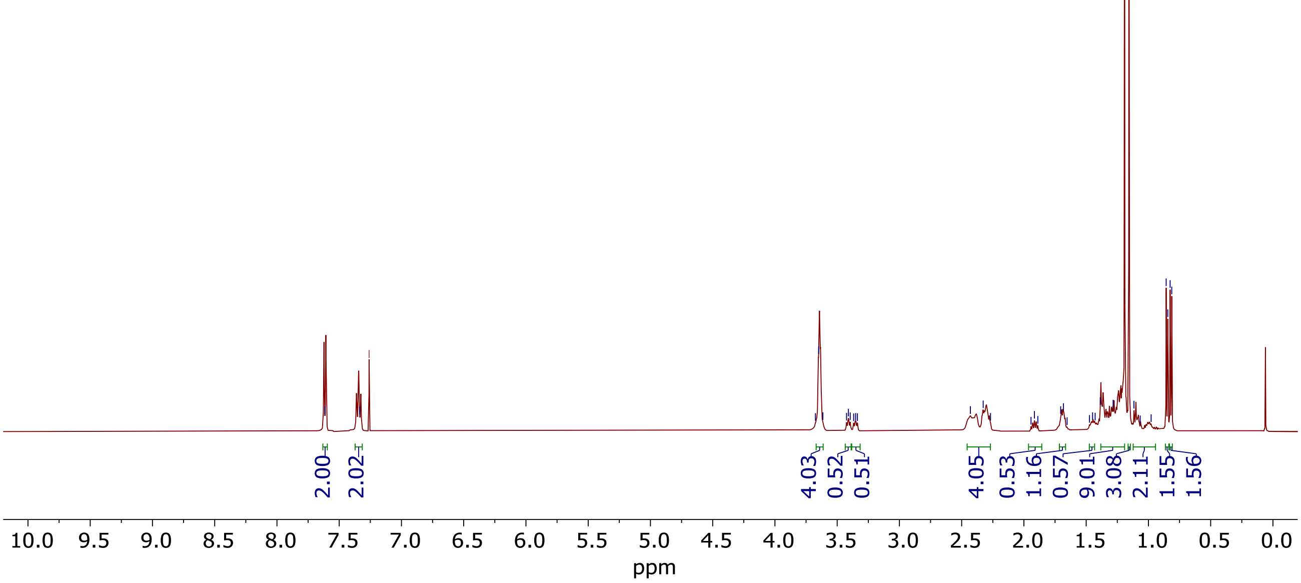

^{13}C NMR, 126 MHz, $CDCl_3$

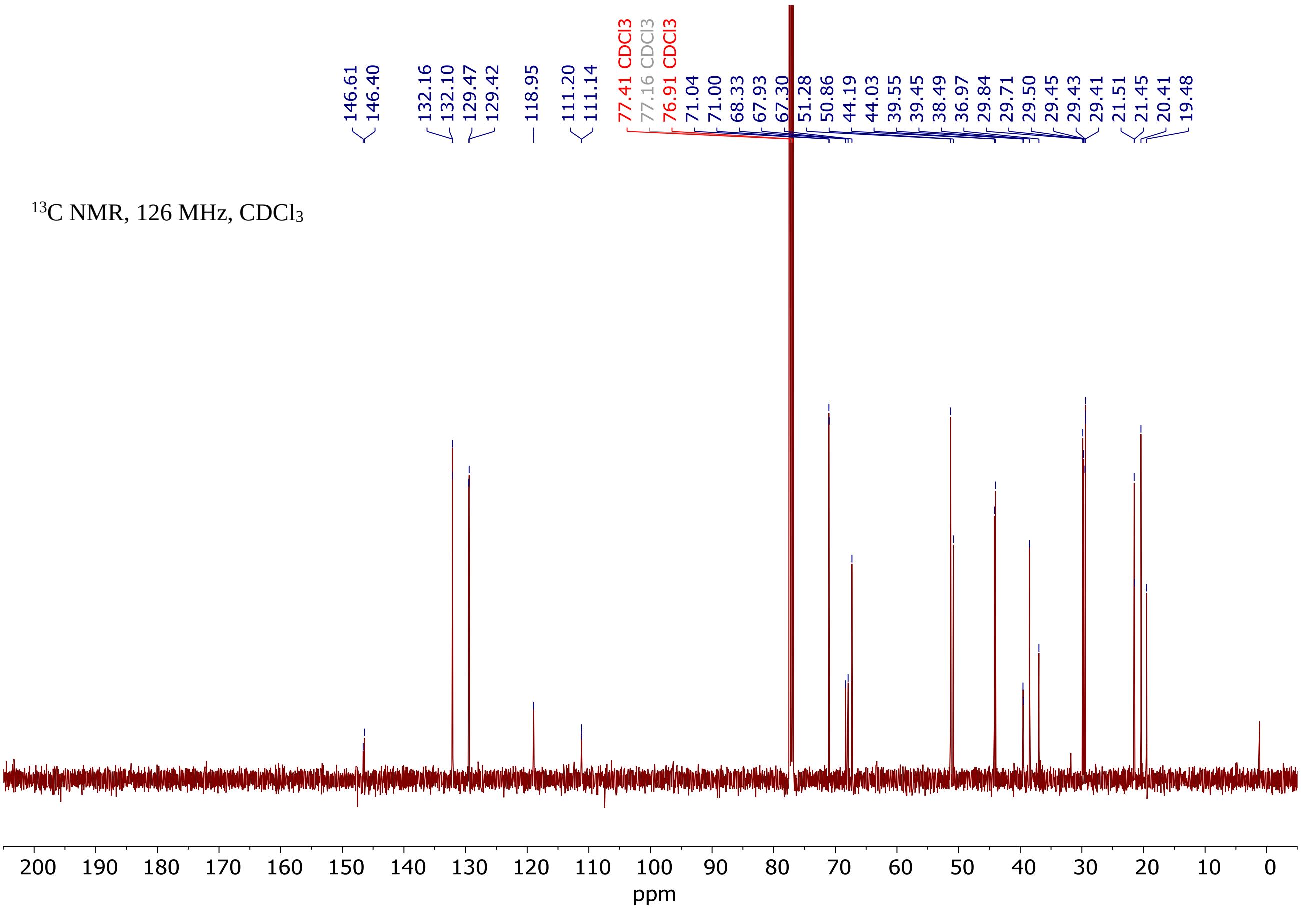

4-(Cyclohexyl(morpholino)methyl)benzonitrile (12f)

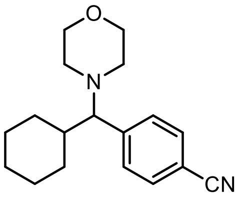

^{1}H NMR, 700 MHz, $CDCl_3$

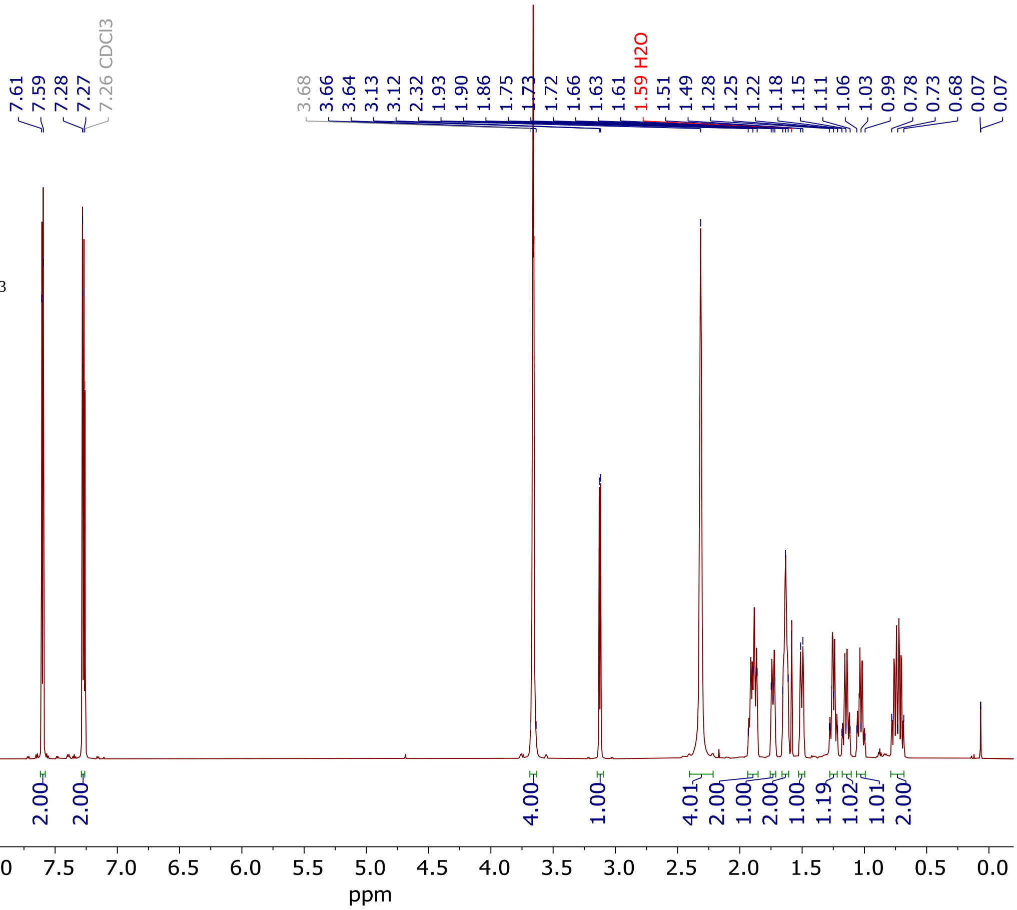

^{13}C NMR, 176 MHz, $CDCl_3$

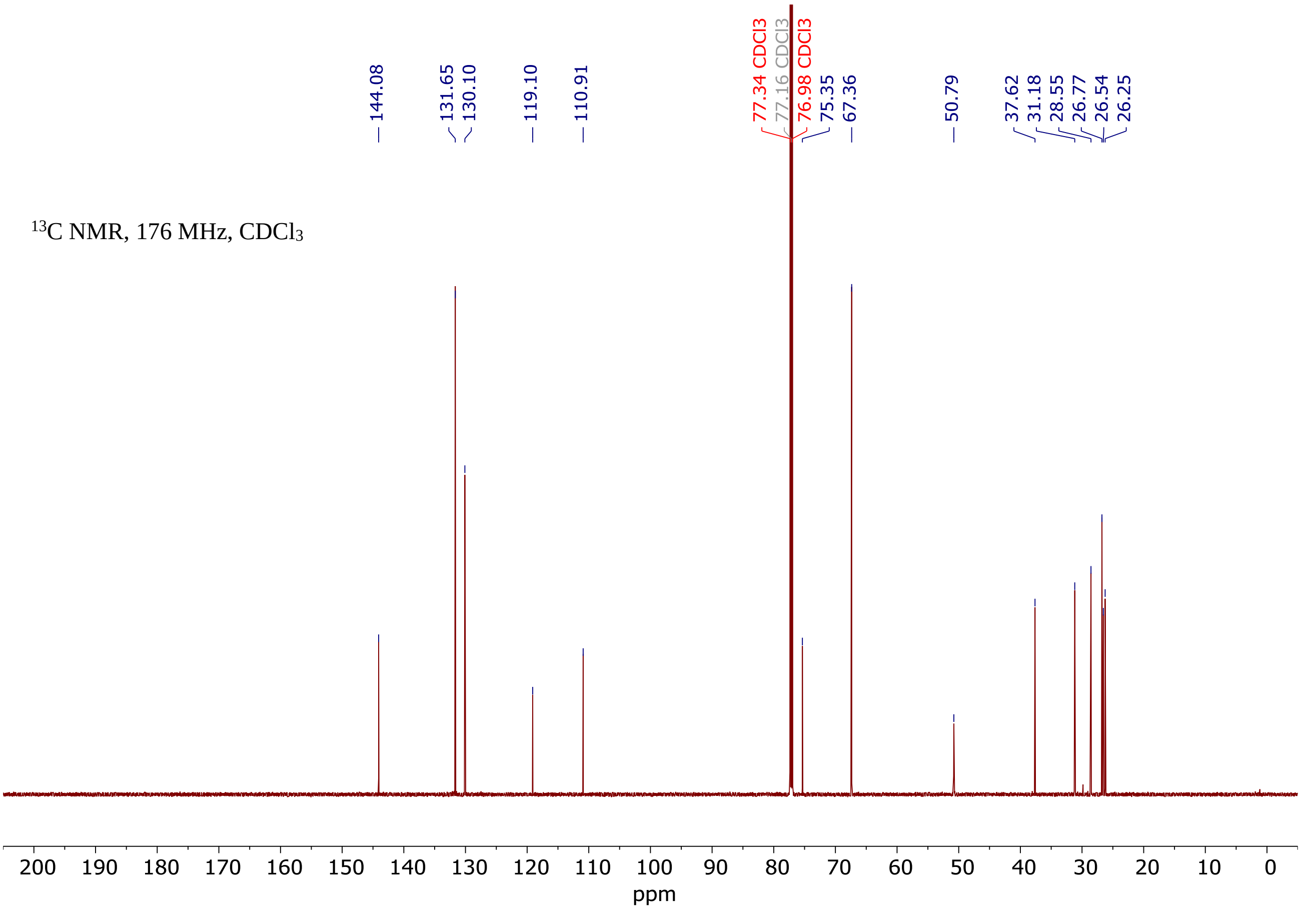

4-(Morpholino(tetrahydro-2H-pyran-4-yl)methyl)benzonitrile (12g)

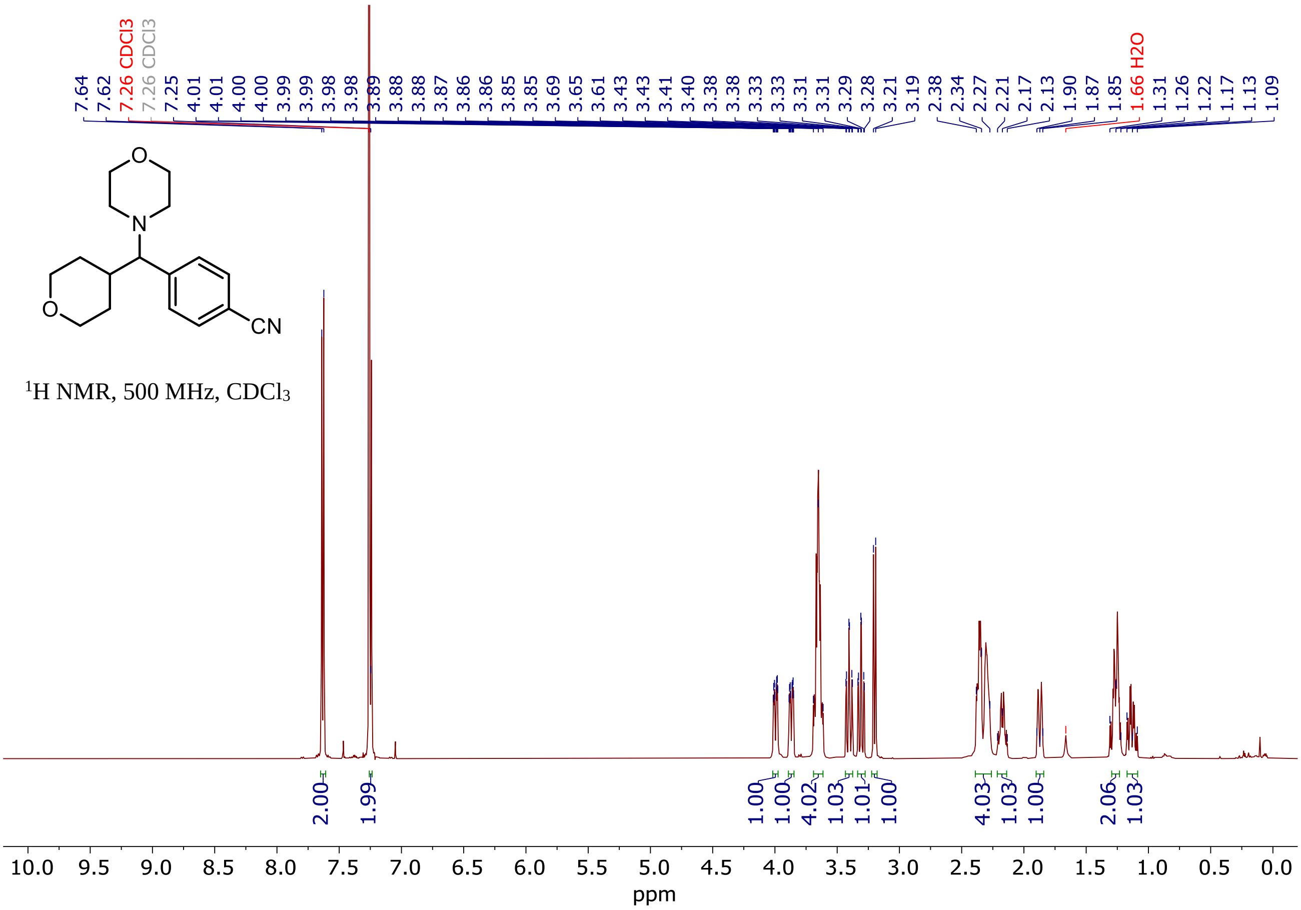

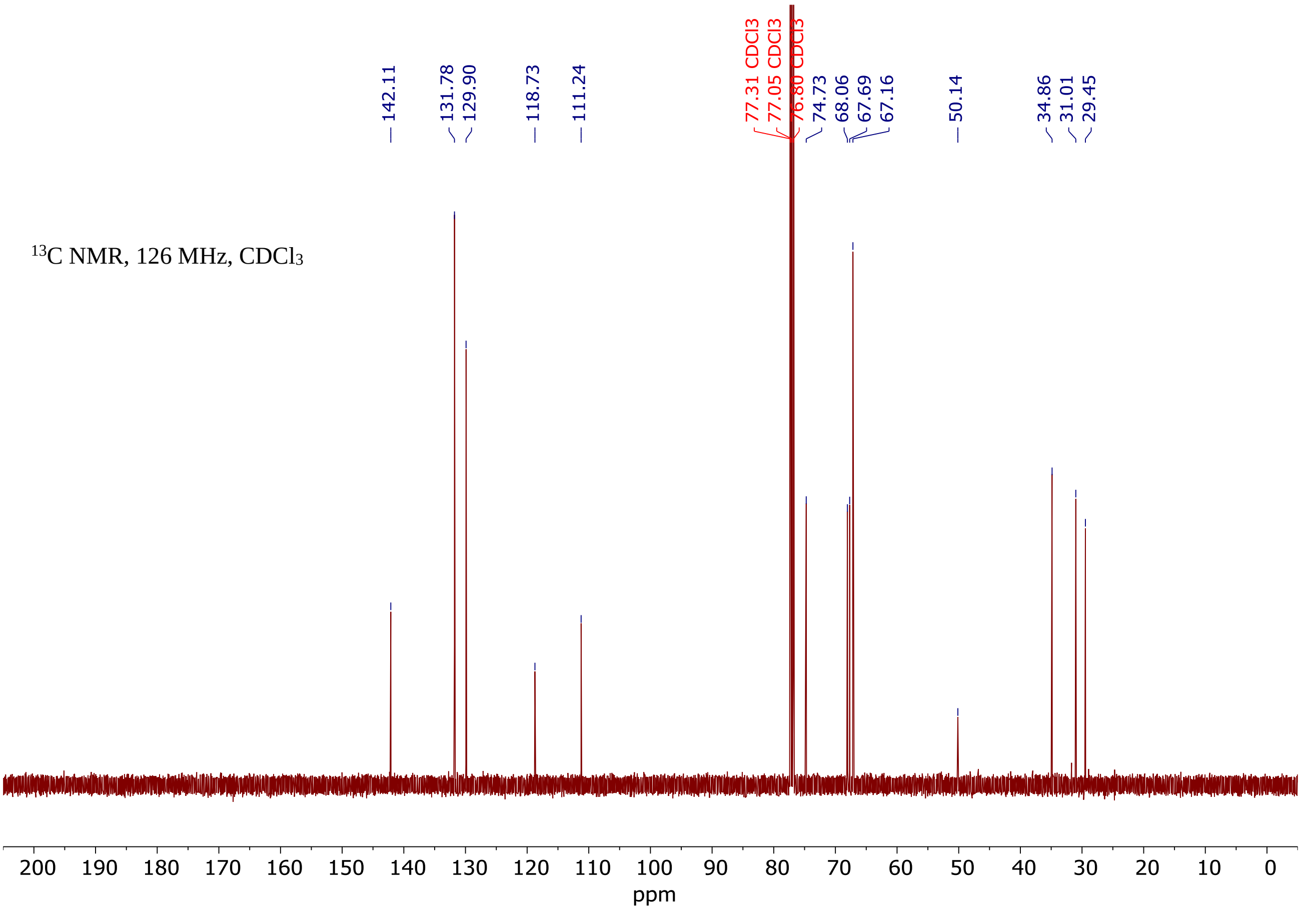

4-(Cyclohex-3-en-1-yl(morpholino)methyl)benzonitrile (12h)

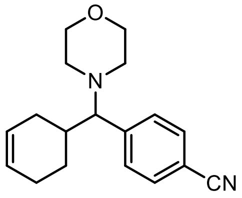

^{1}H NMR, 400 MHz, $CDCl_3$

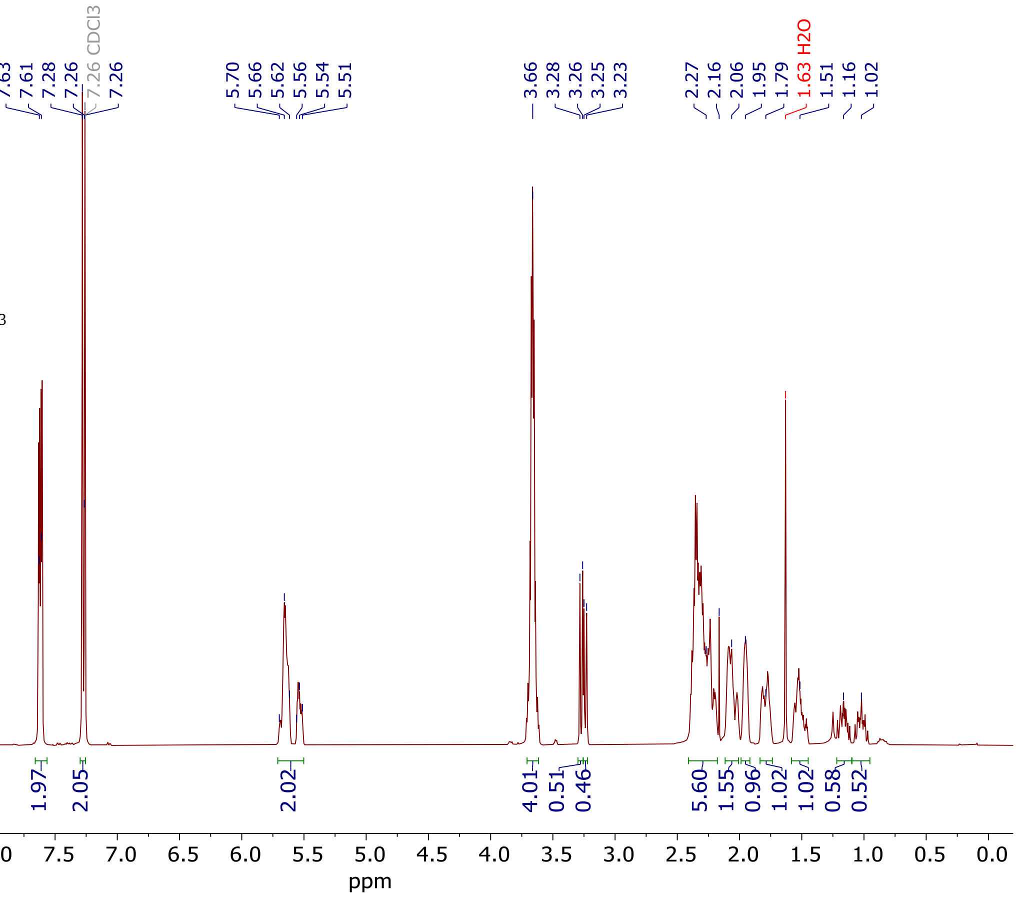

^{13}C NMR, 101 MHz, $CDCl_3$

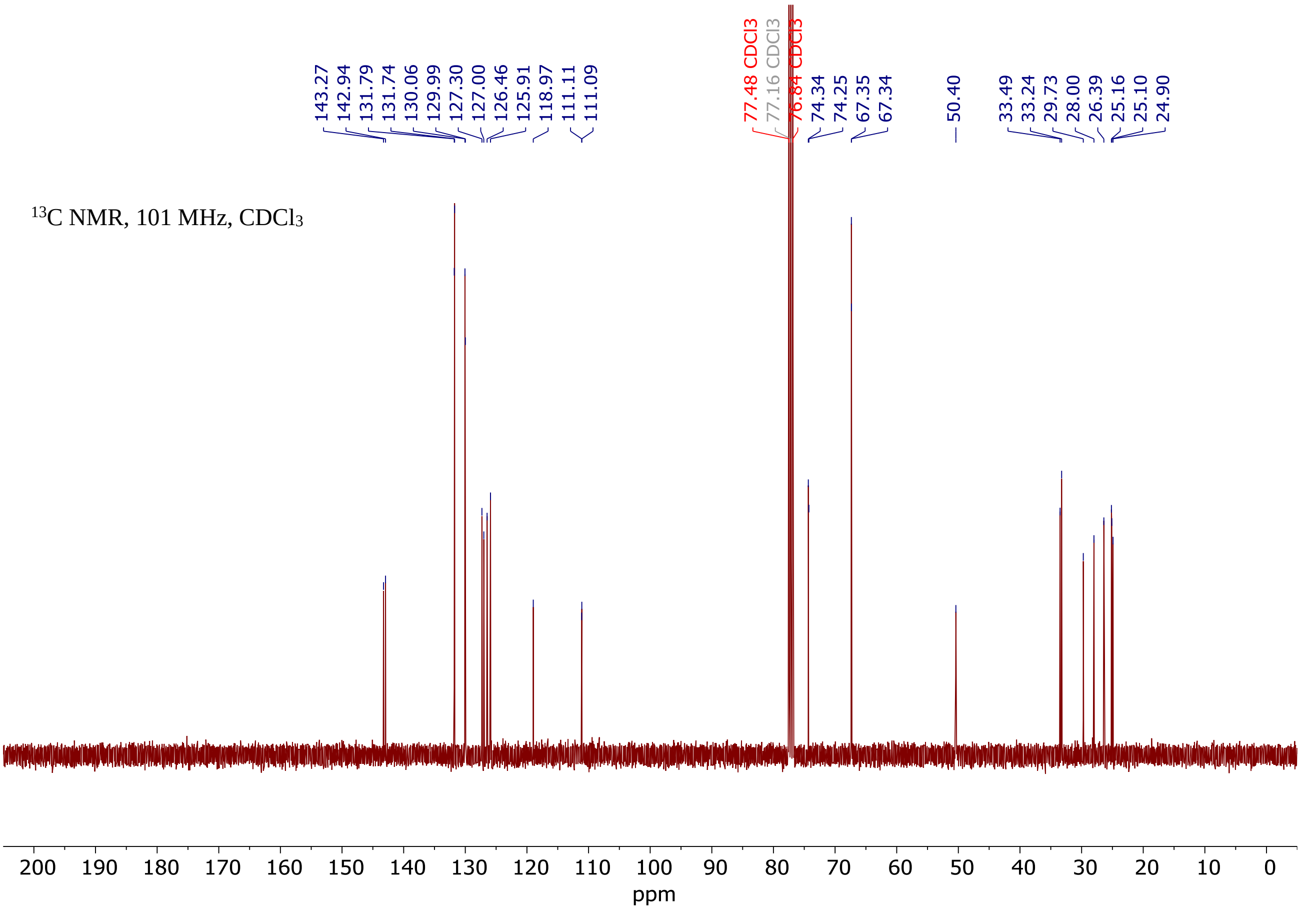

Tert-butyl 4-((4-cyanophenyl)(morpholino)methyl)piperidine-1-carboxylate (12i)

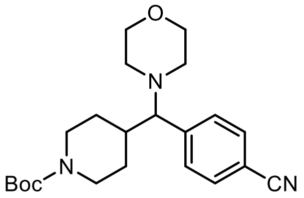

^{1}H NMR, 500 MHz, $CDCl_3$

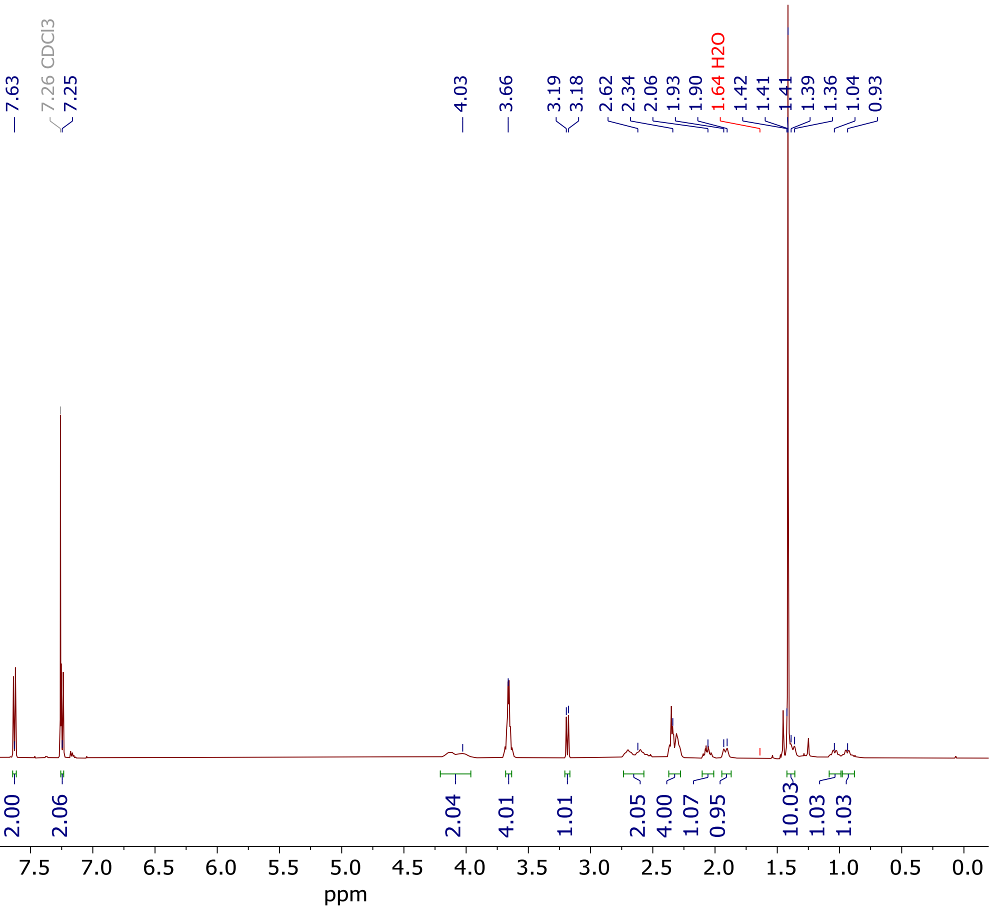

^{13}C NMR, 176 MHz, $CDCl_3$

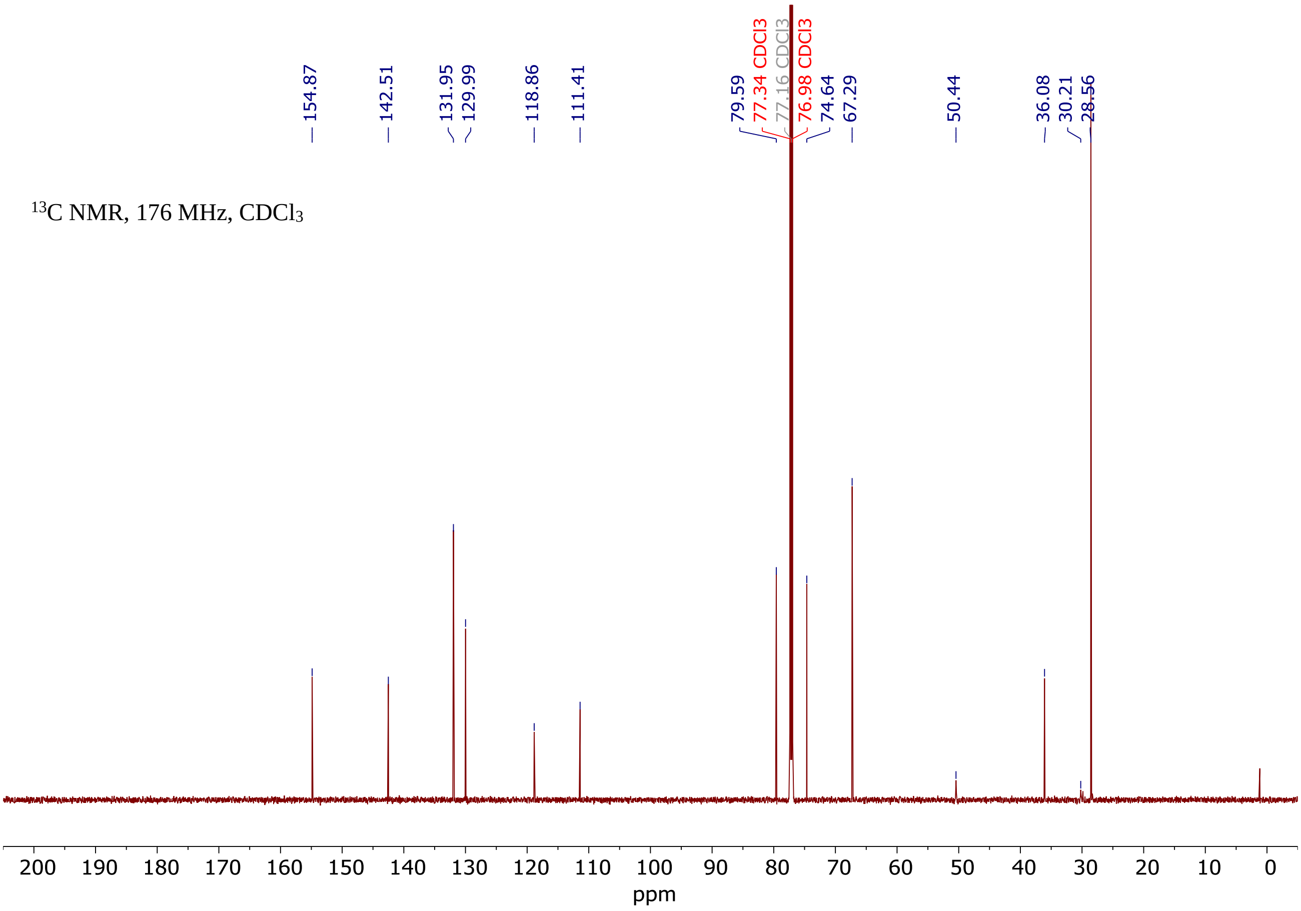

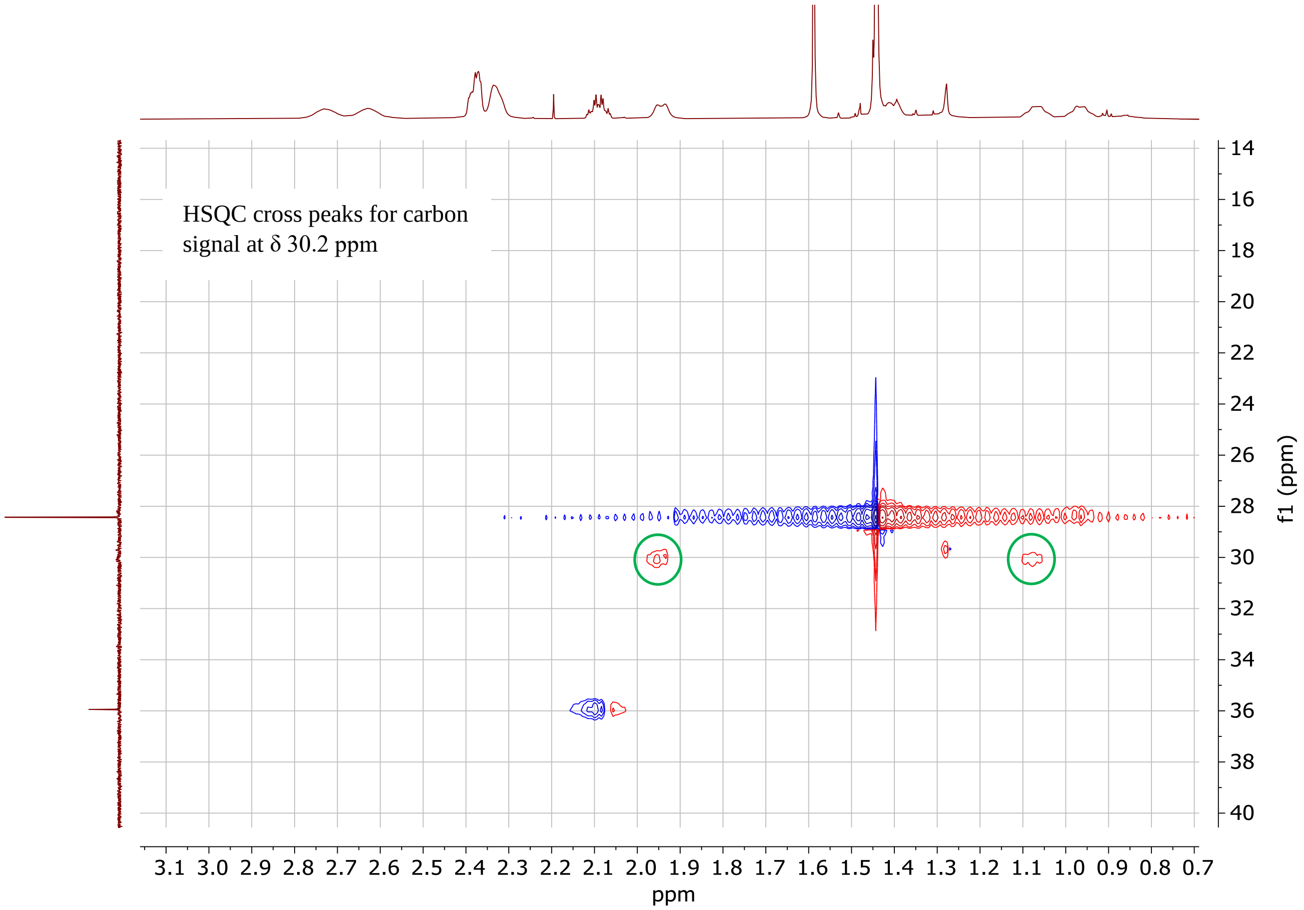
HSQC cross peaks for carbon signal at δ 30.2 ppm
ppm
f1 (ppm)

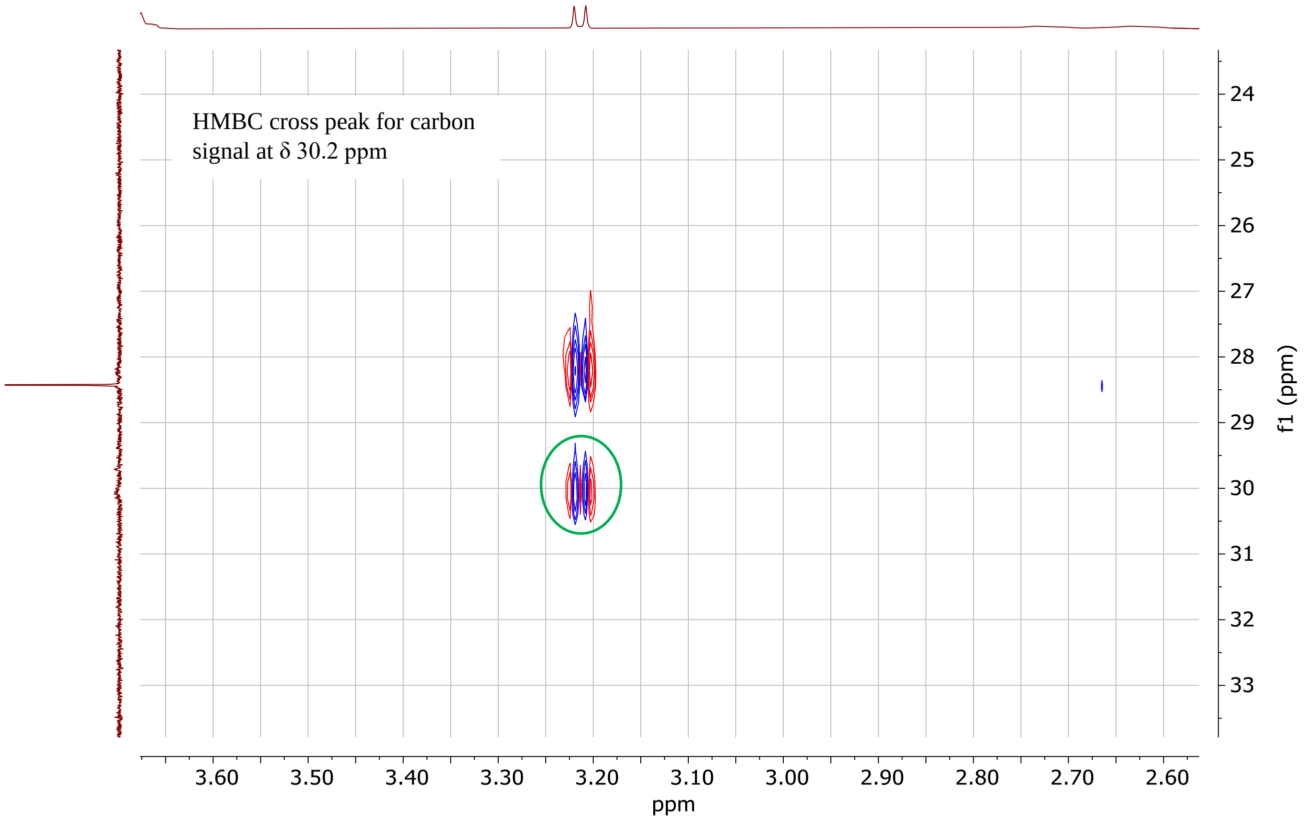
HMBC cross peak for carbon signal at δ 30.2 ppm
ppm
f1 (ppm)

Tert-butyl 3-((4-cyanophenyl)(morpholino)methyl)azetidine-1-carboxylate (12j)

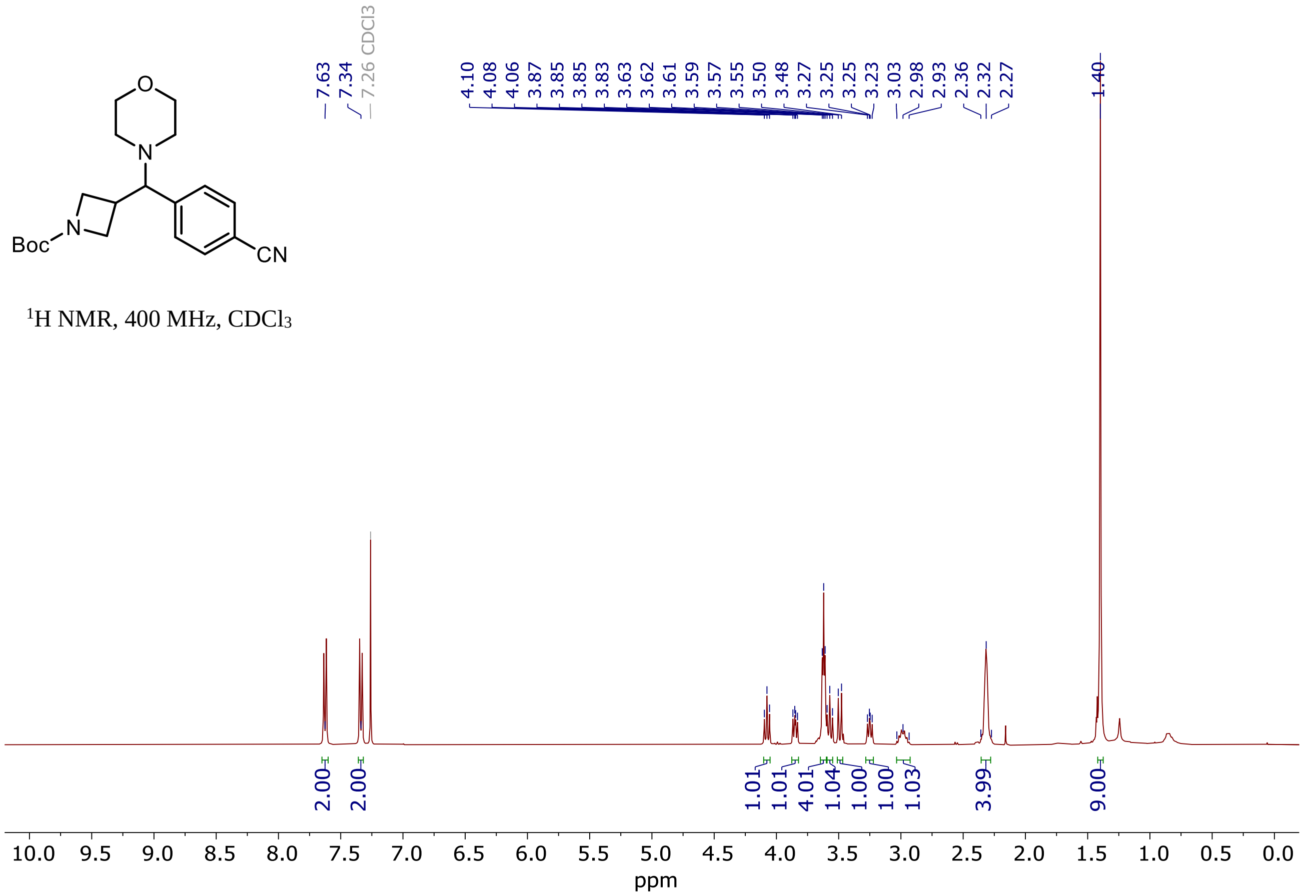

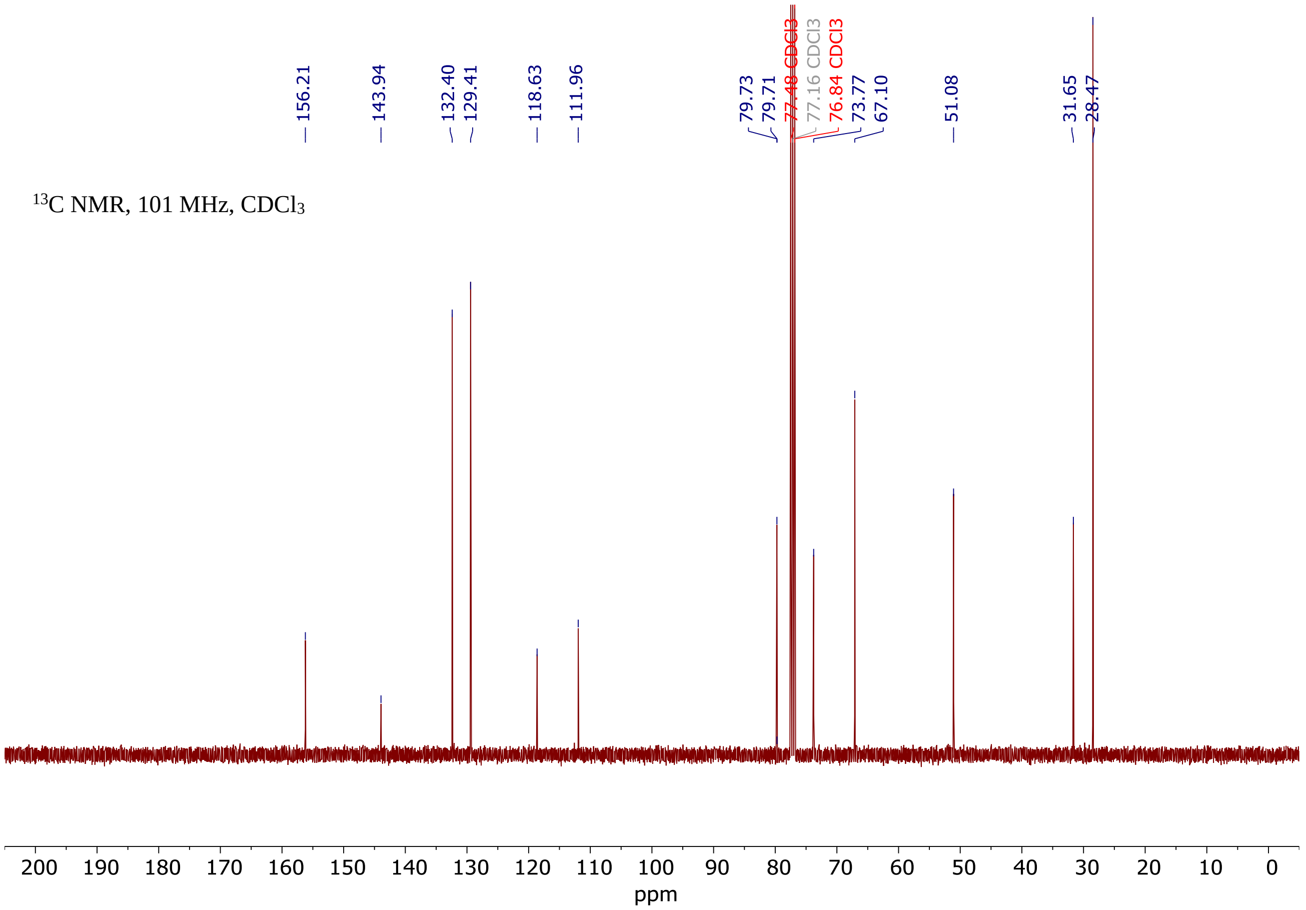

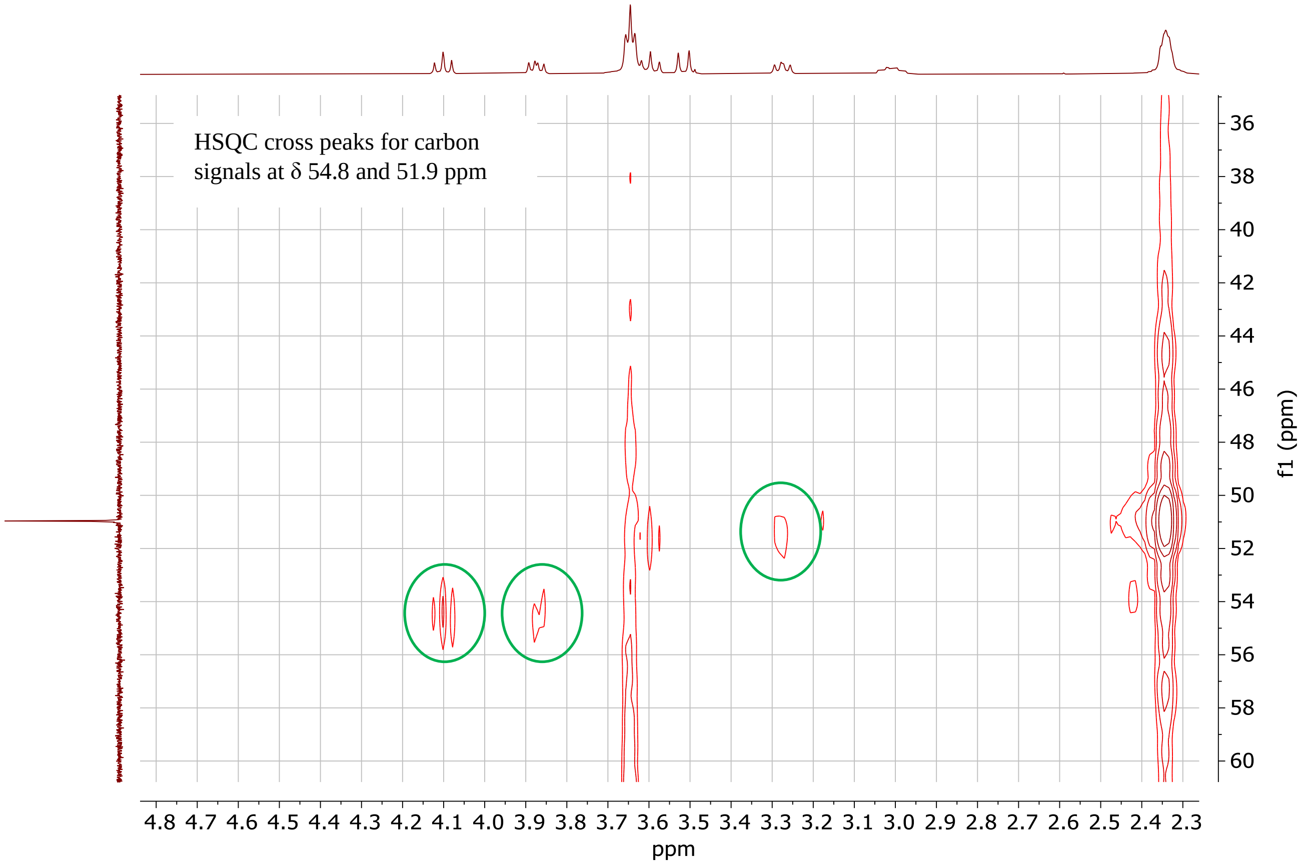
HSQC cross peaks for carbon signals at δ 54.8 and 51.9 ppm
f1 (ppm)
ppm

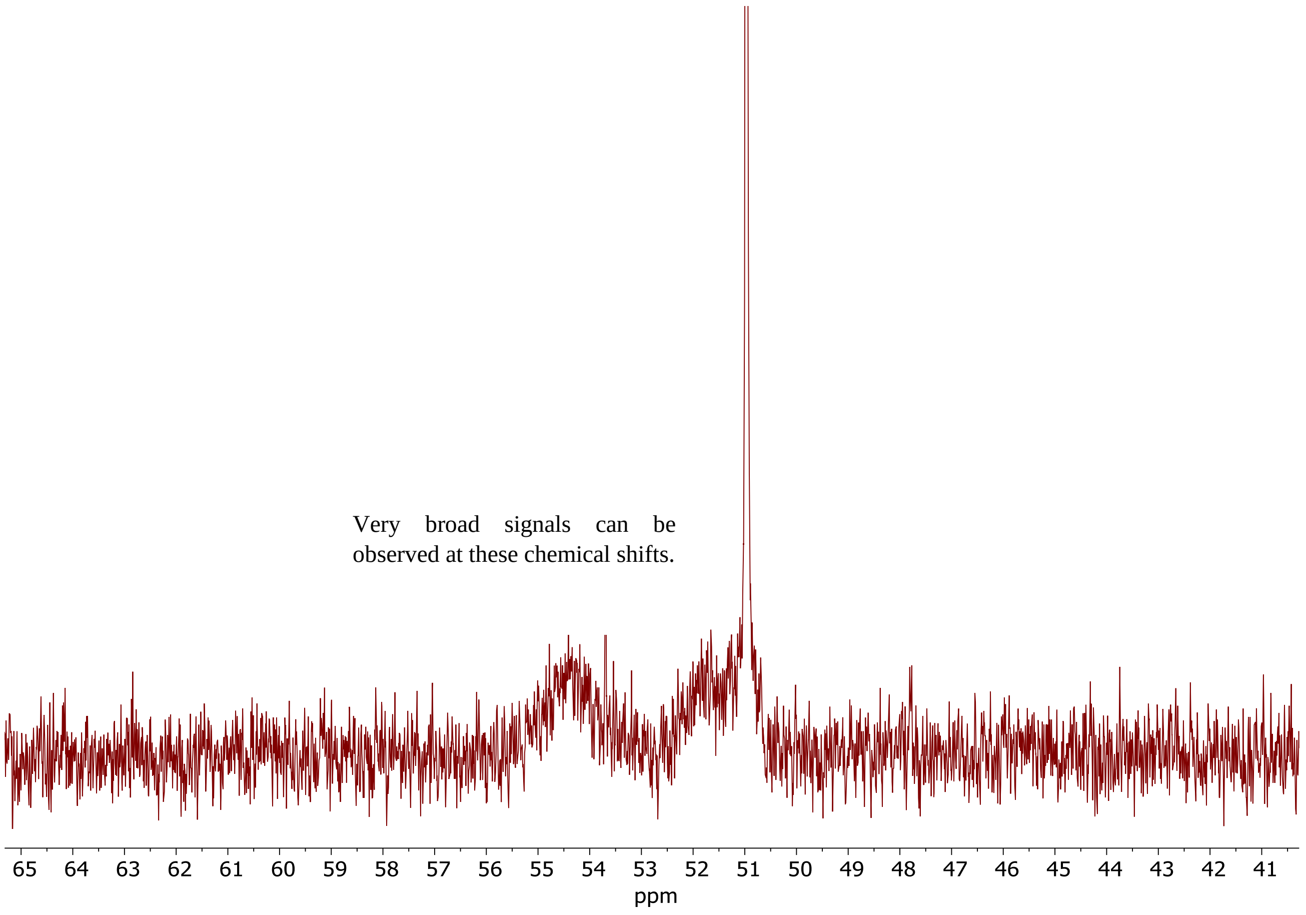
Very broad signals can be observed at these chemical shifts.
ppm

Methyl 4-(4-cyanophenyl)-4-thiomorpholinobutanoate (12k)

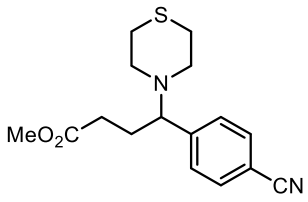

^{1}H NMR, 500 MHz, $CDCl_3$

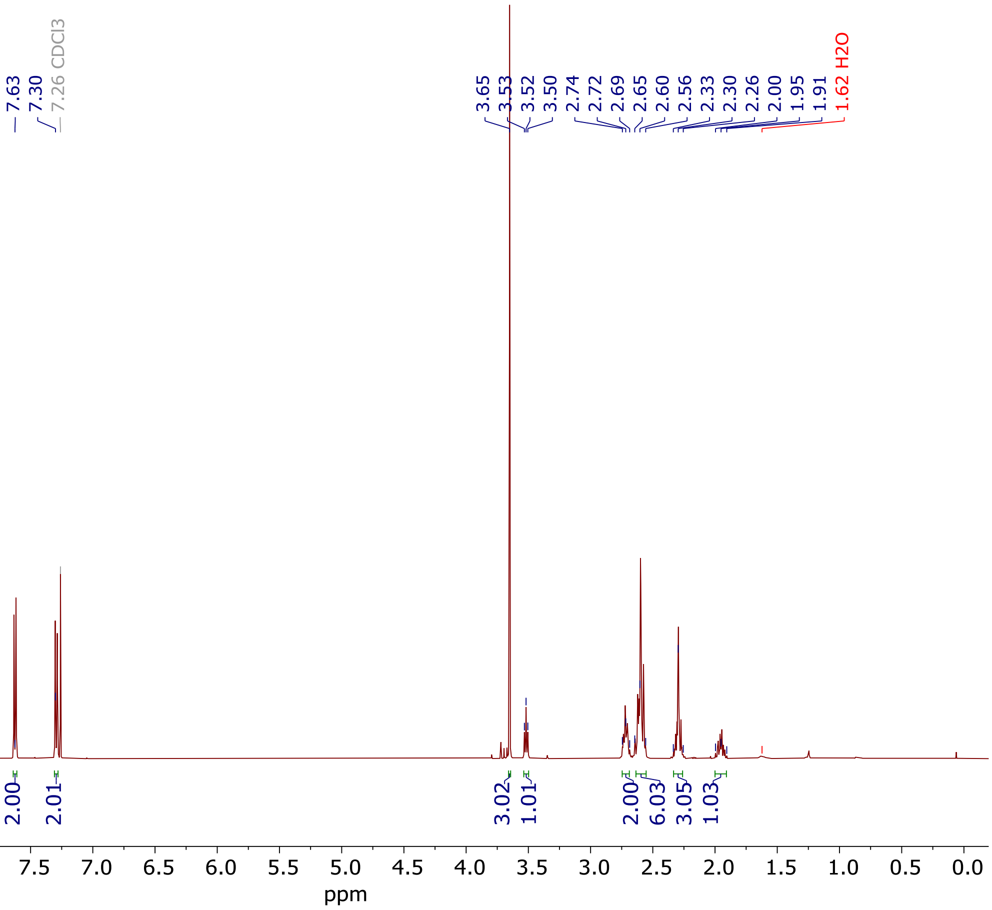

^{13}C NMR, 126 MHz, $CDCl_3$

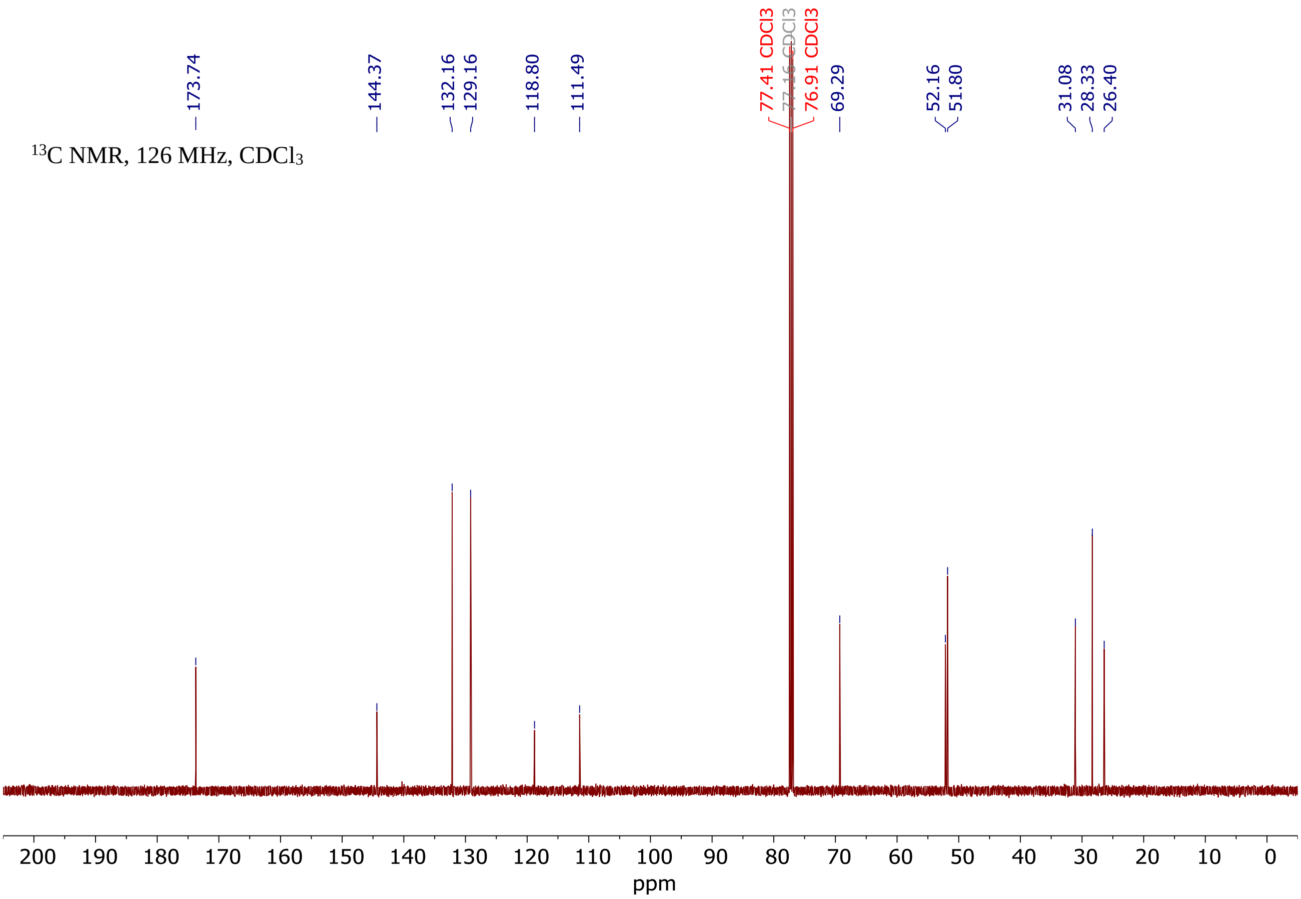

1-((4-Cyanophenyl)(cyclohexyl)methyl)piperidine-4-carbonitrile (12l)

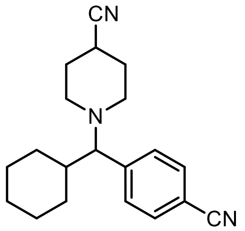

^{1}H NMR, 500 MHz, $CDCl_3$

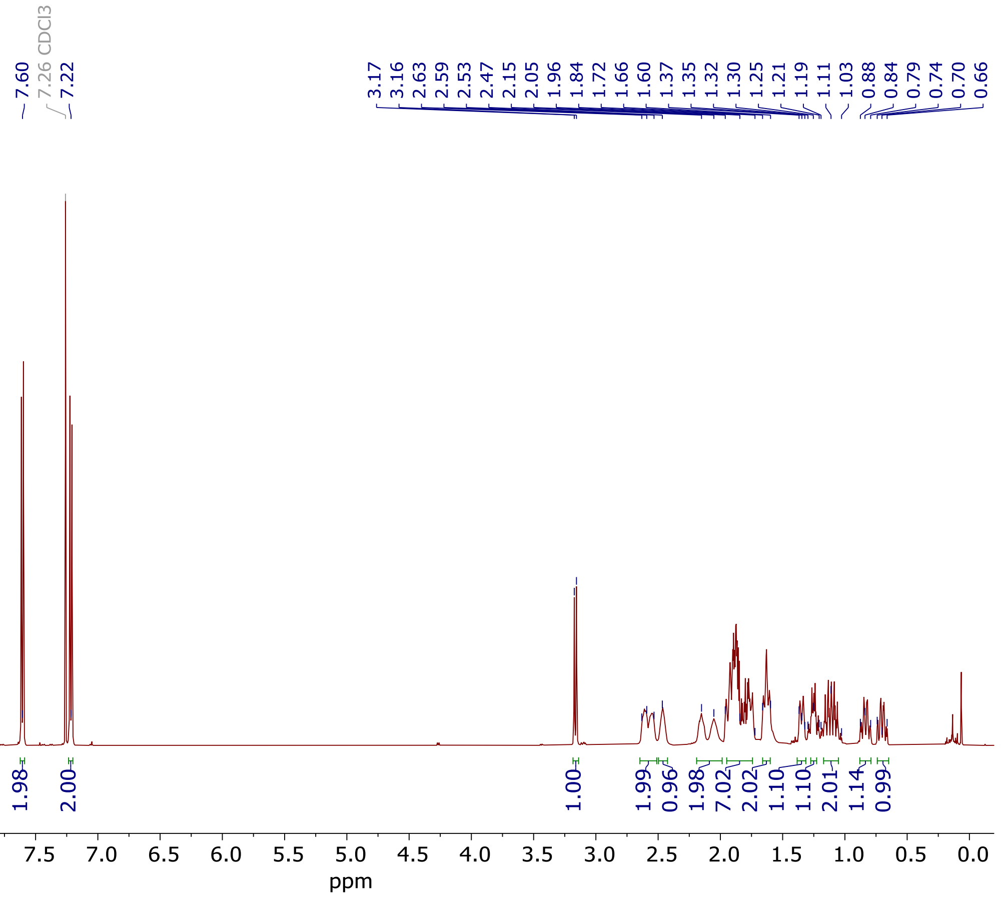

^{13}C NMR, 126 MHz, $CDCl_3$

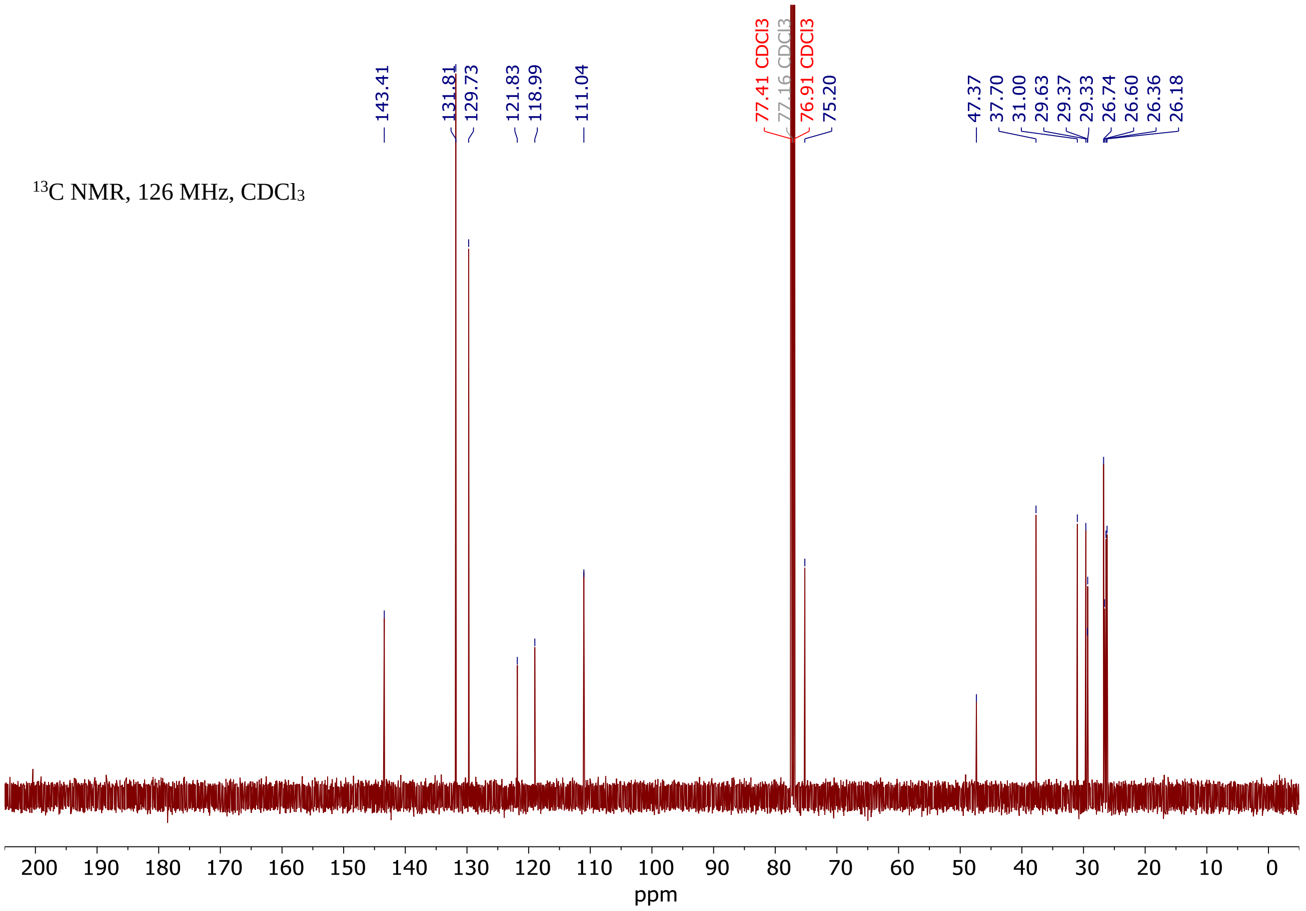

4-(Cyclohexyl(1,4-dioxa-8-azaspiro[4.5]decan-8-yl)methyl)benzonitrile (12m)

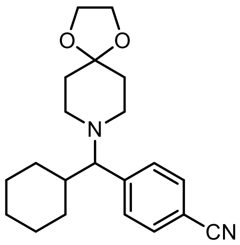

^{1}H NMR, 400 MHz, $CDCl_3$

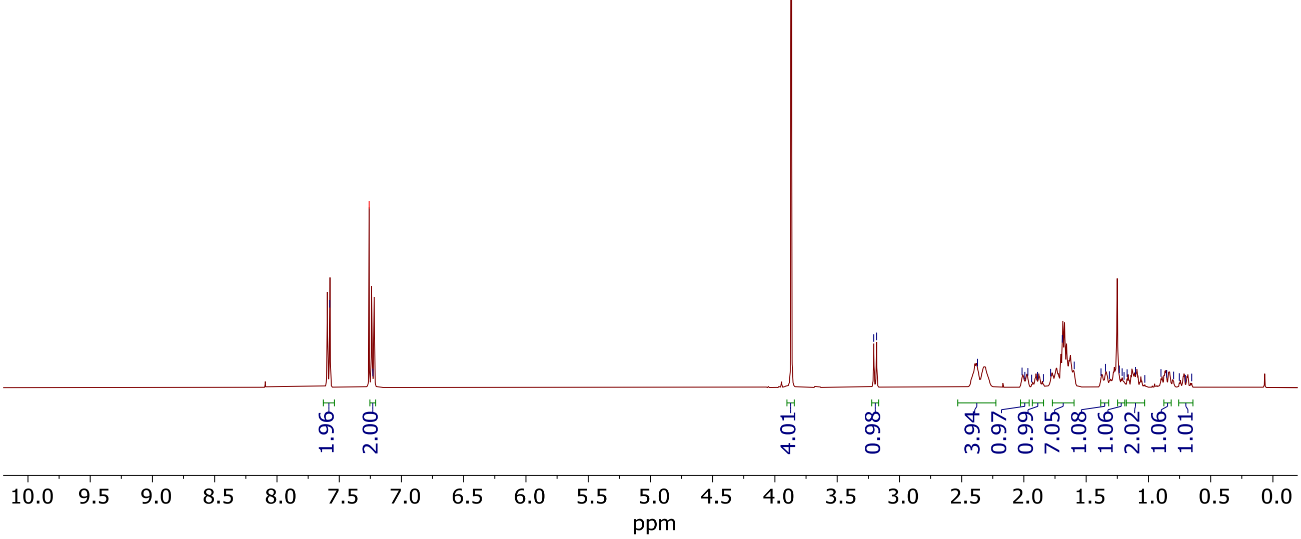

^{13}C NMR, 101 MHz, $CDCl_3$

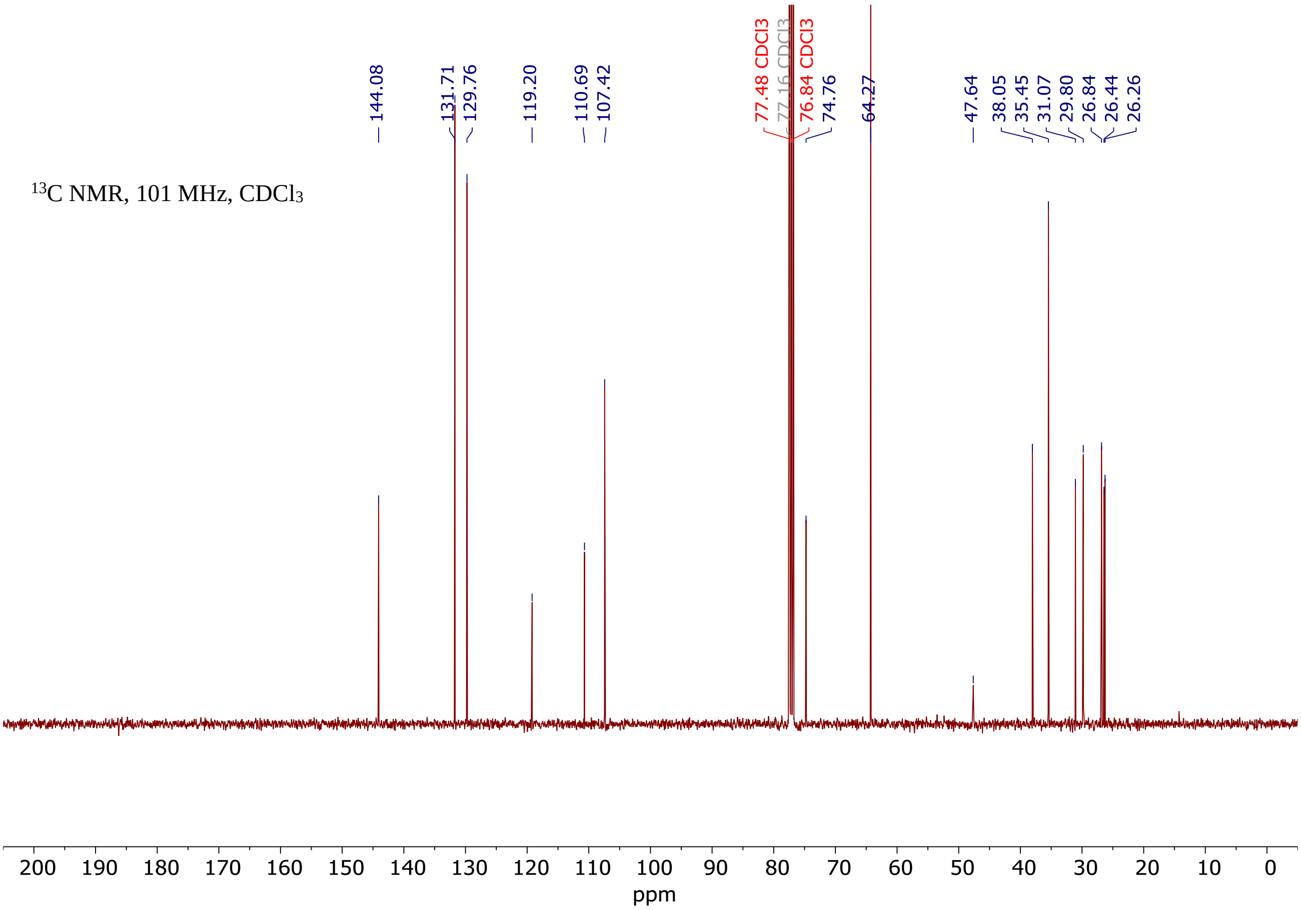

4-(Cyclohexyl(4-(pyrimidin-2-yl)piperazin-1-yl)methyl)benzonitrile (12n)

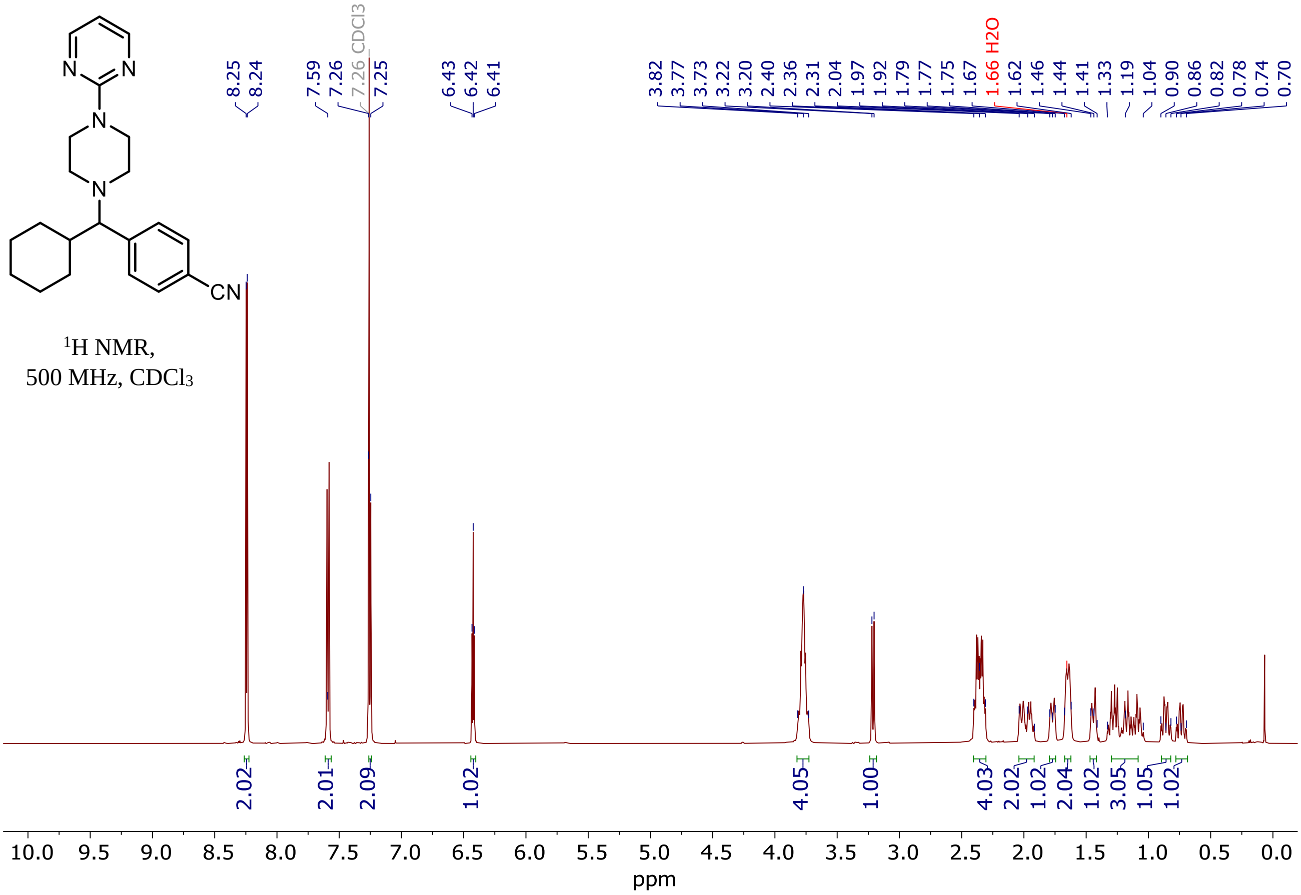

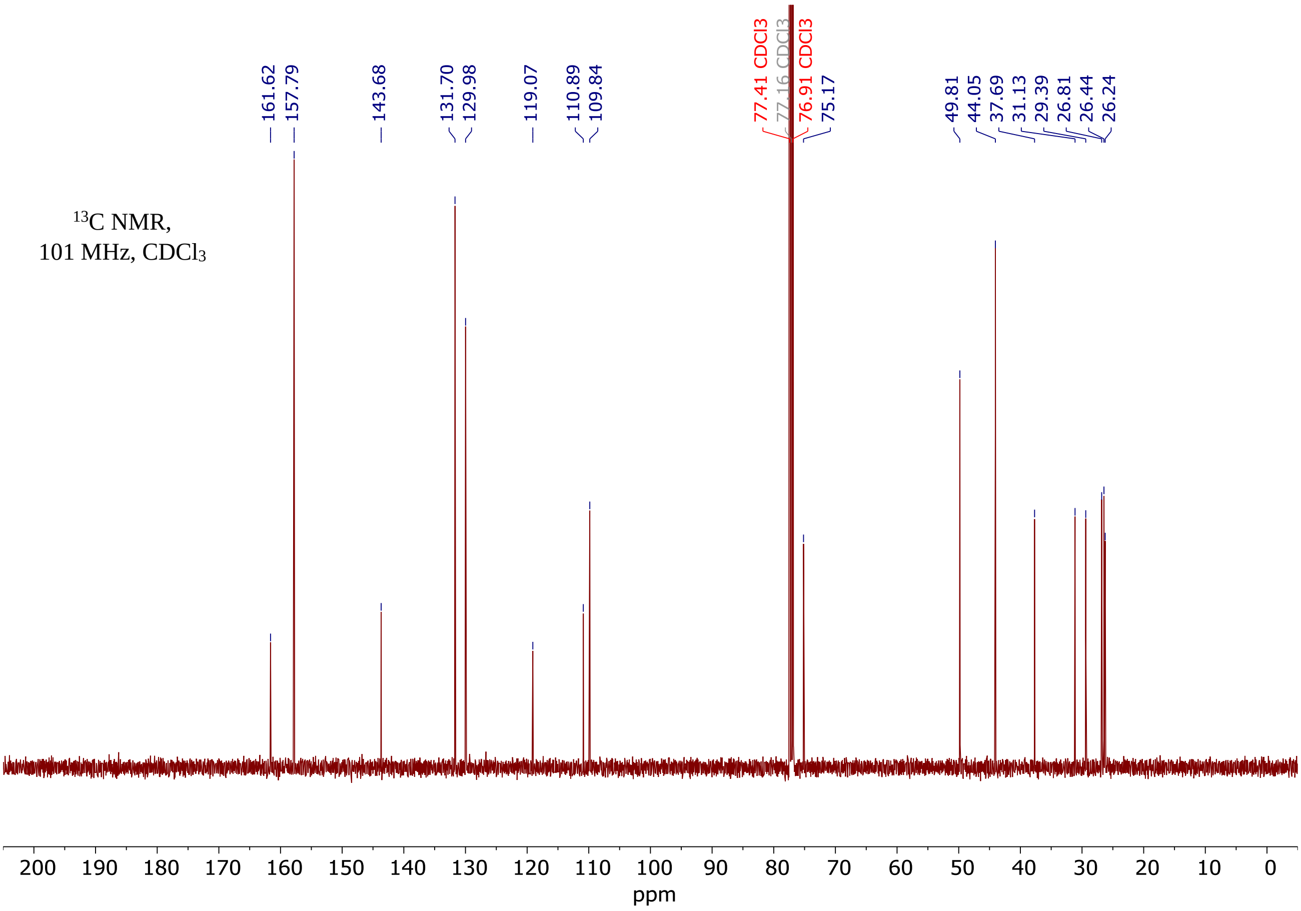

4-(Cyclohexyl(4-(methylsulfonyl)piperazin-1-yl)methyl)benzonitrile (12o)

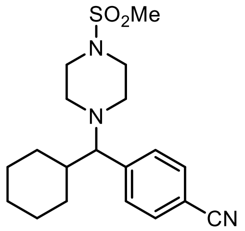

^{1}H NMR, 500 MHz, $CDCl_3$

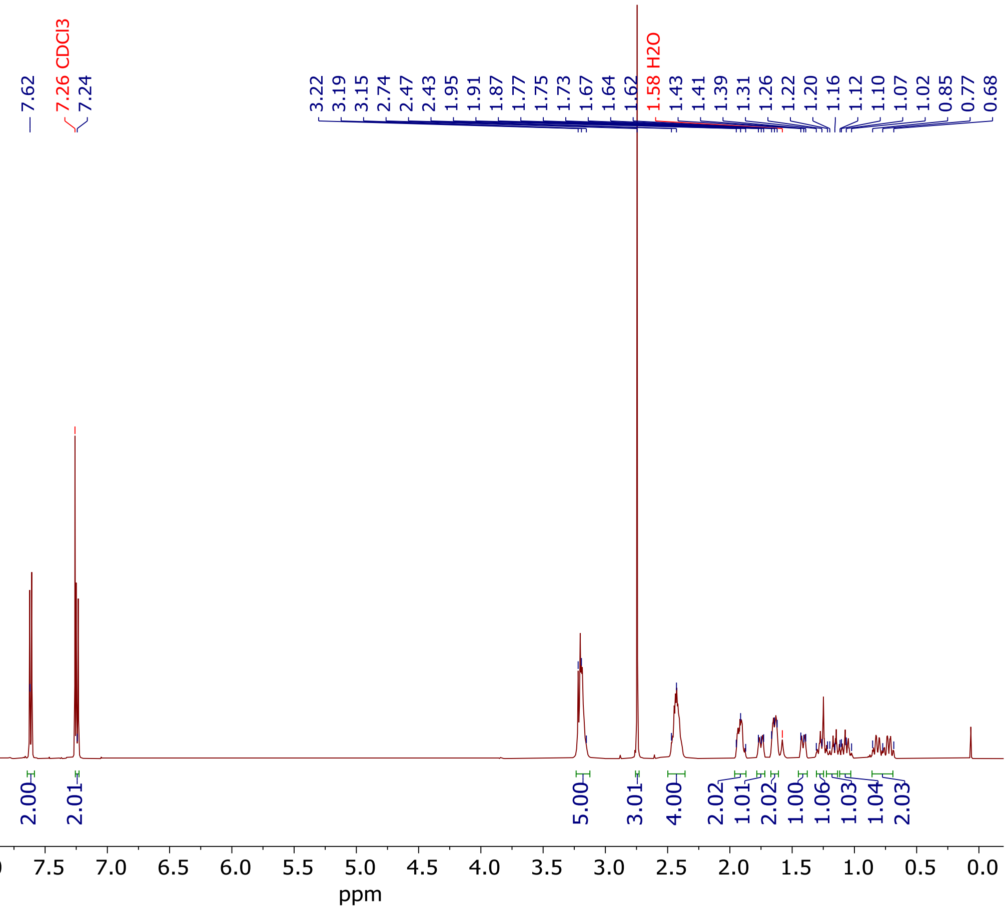

^{13}C NMR, 126 MHz, $CDCl_3$

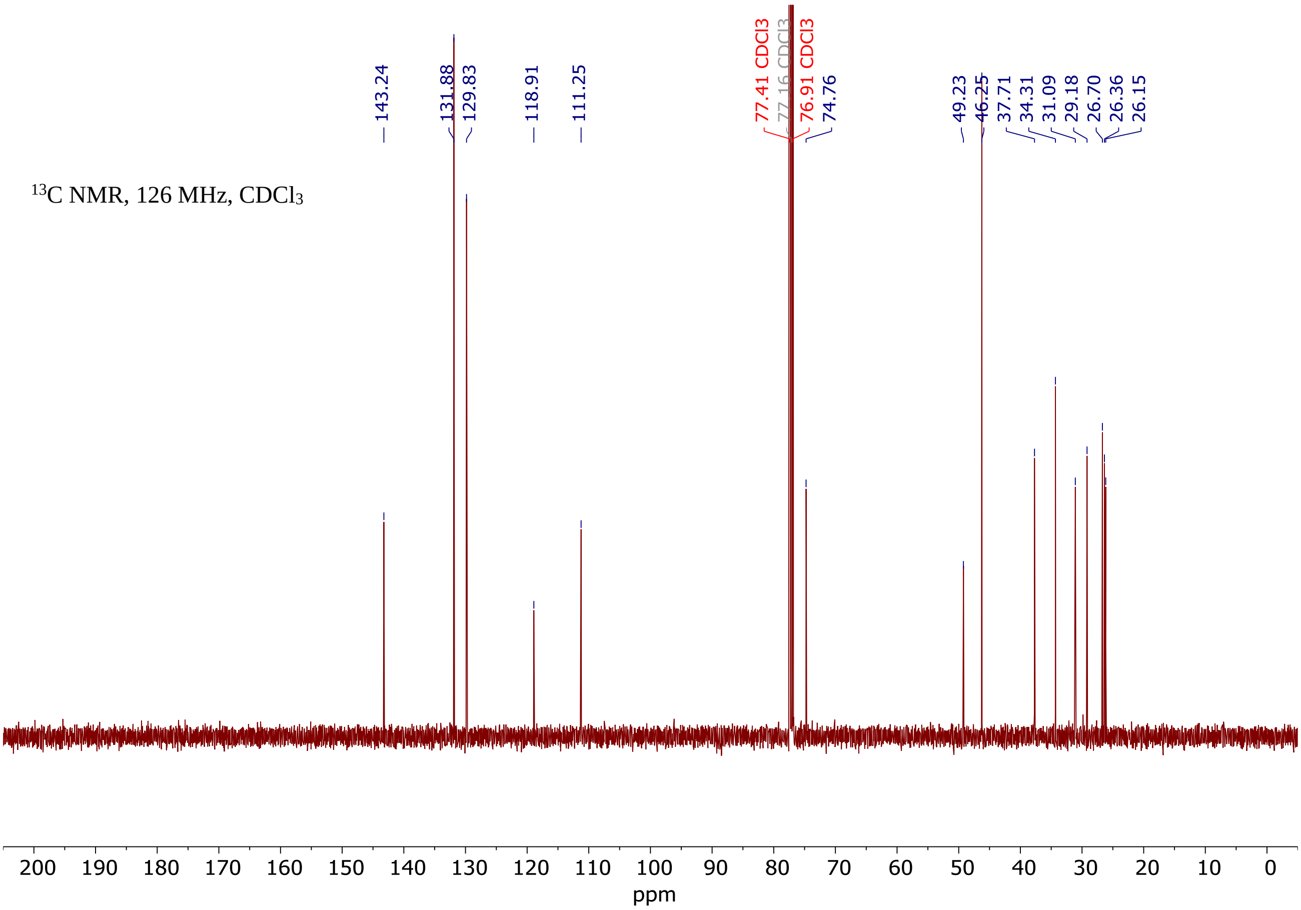

Tert-butyl (1-(4-cyanophenyl)-4-methoxy-4-oxobutyl)prolinate (12p)

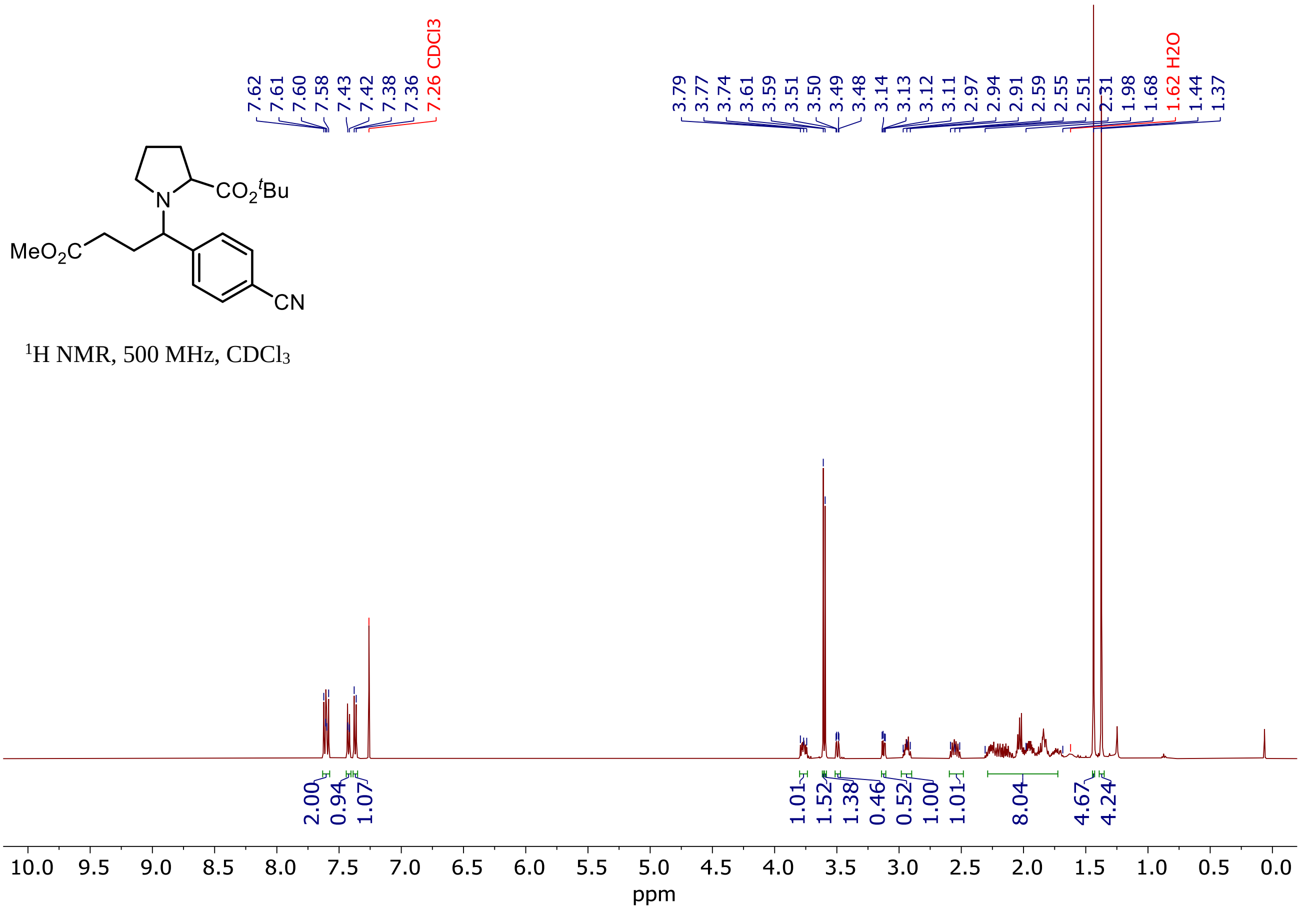

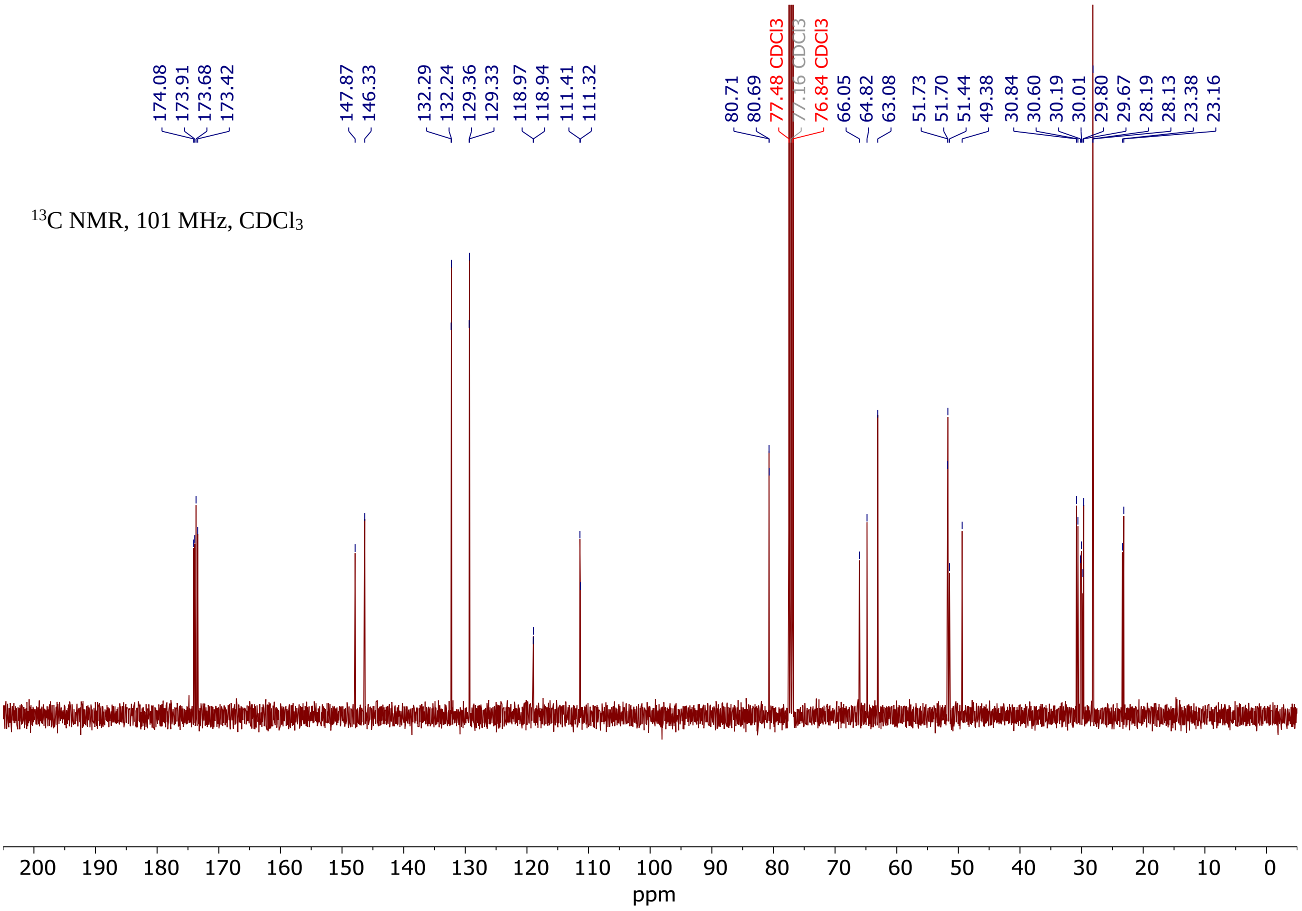

Methyl 4-(3-((tert-butoxycarbonyl)amino)azetidine-1-yl)-4-(4-cyanophenyl)butanoate (12q)

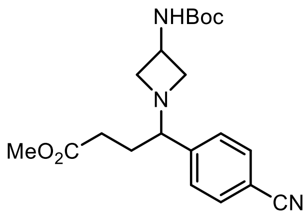

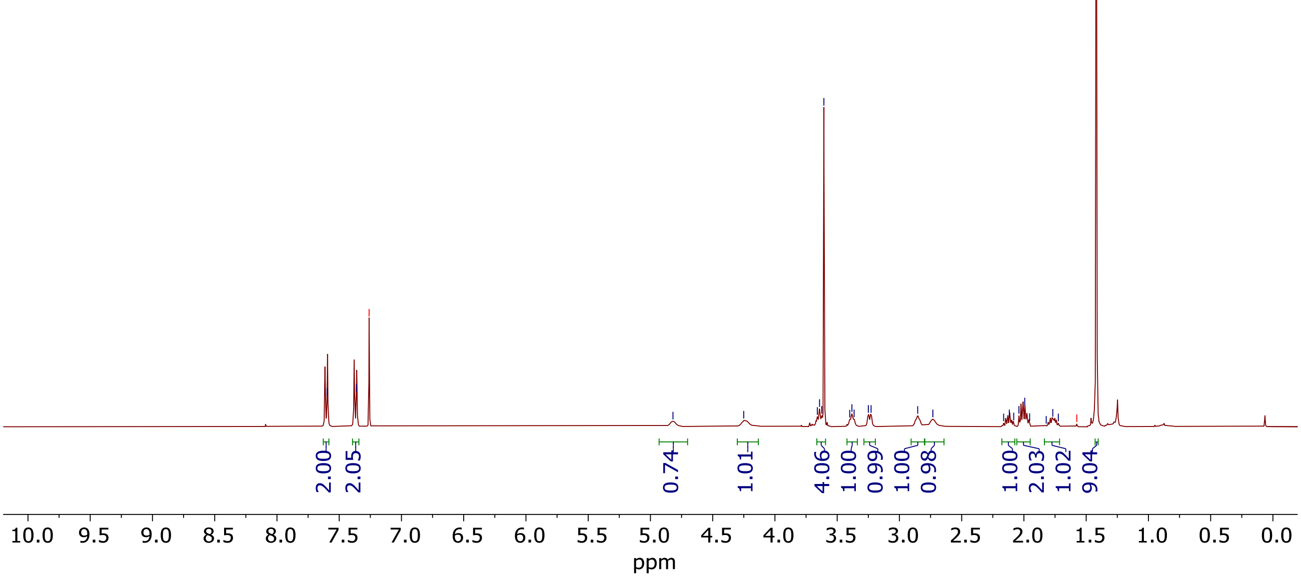

^{1}H NMR, 400 MHz, $CDCl_3$

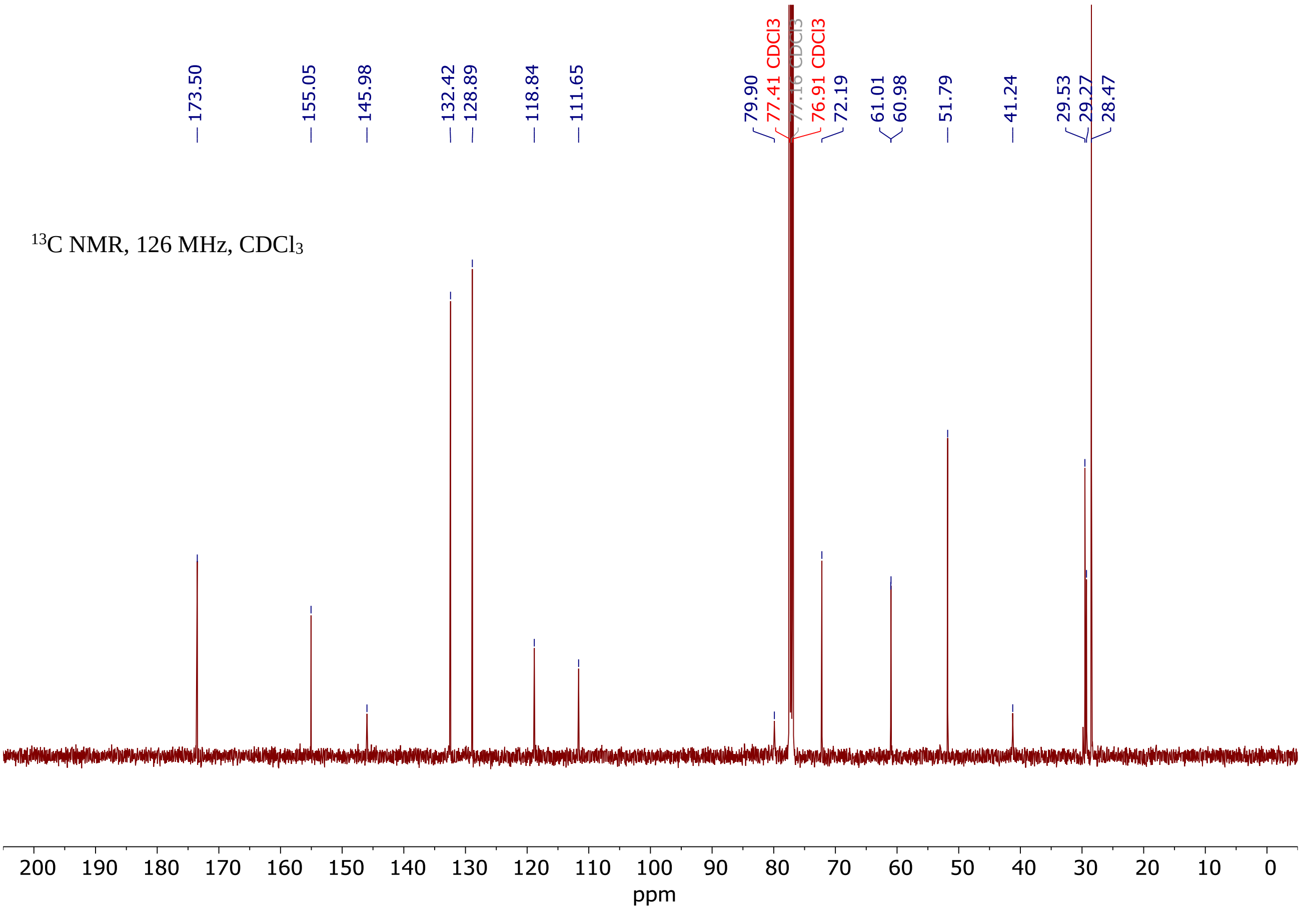

^{13}C NMR, 126 MHz, $CDCl_3$

4-((Bis(2-methoxyethyl)amino)(cyclohexyl)methyl)benzonitrile (12r)

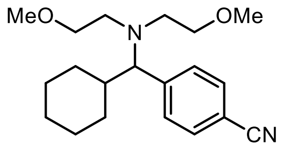

^{1}H NMR, 500 MHz, $CDCl_3$

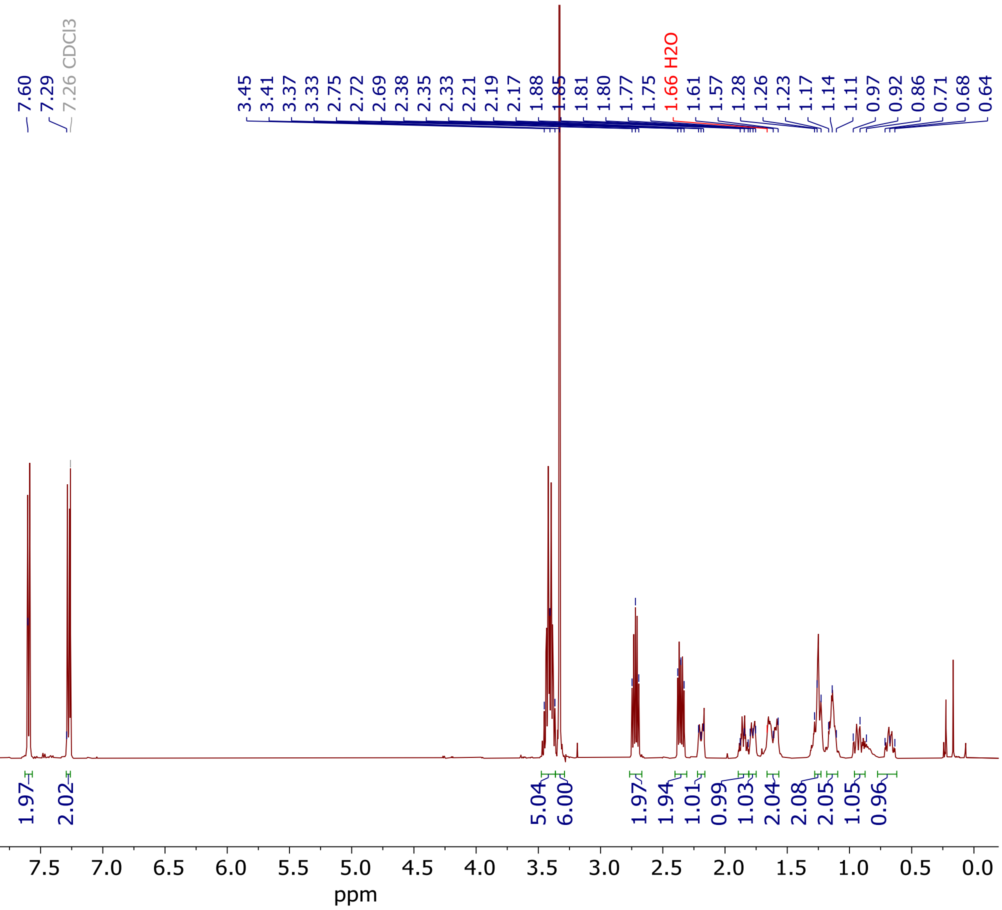

^{13}C NMR, 126 MHz, $CDCl_3$

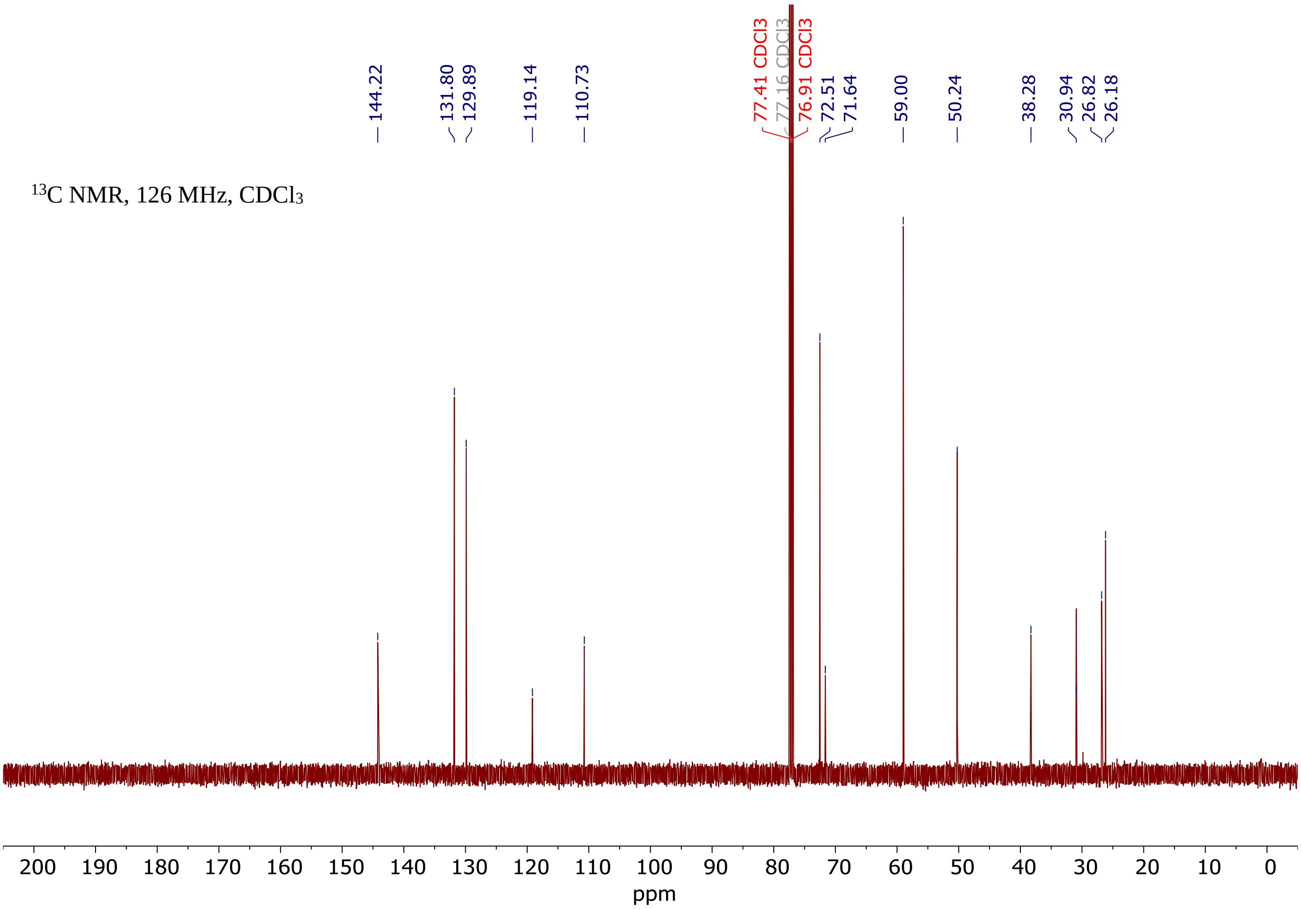

***Tert*-butyl (2-(((4-cyanophenyl)(cyclohexyl)methyl)(methyl)amino)ethyl)carbamate (12s)**

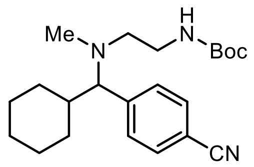

^{1}H NMR, 500 MHz, $CDCl_3$

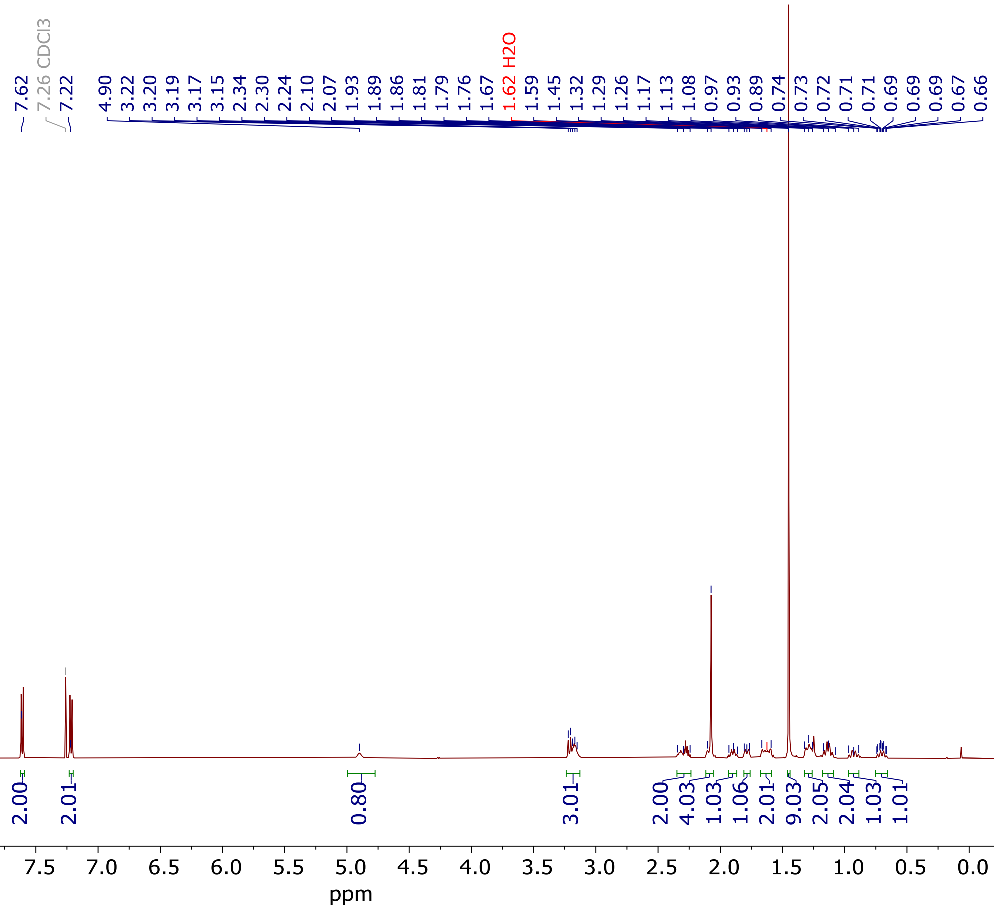

^{13}C NMR, 126 MHz, $CDCl_3$

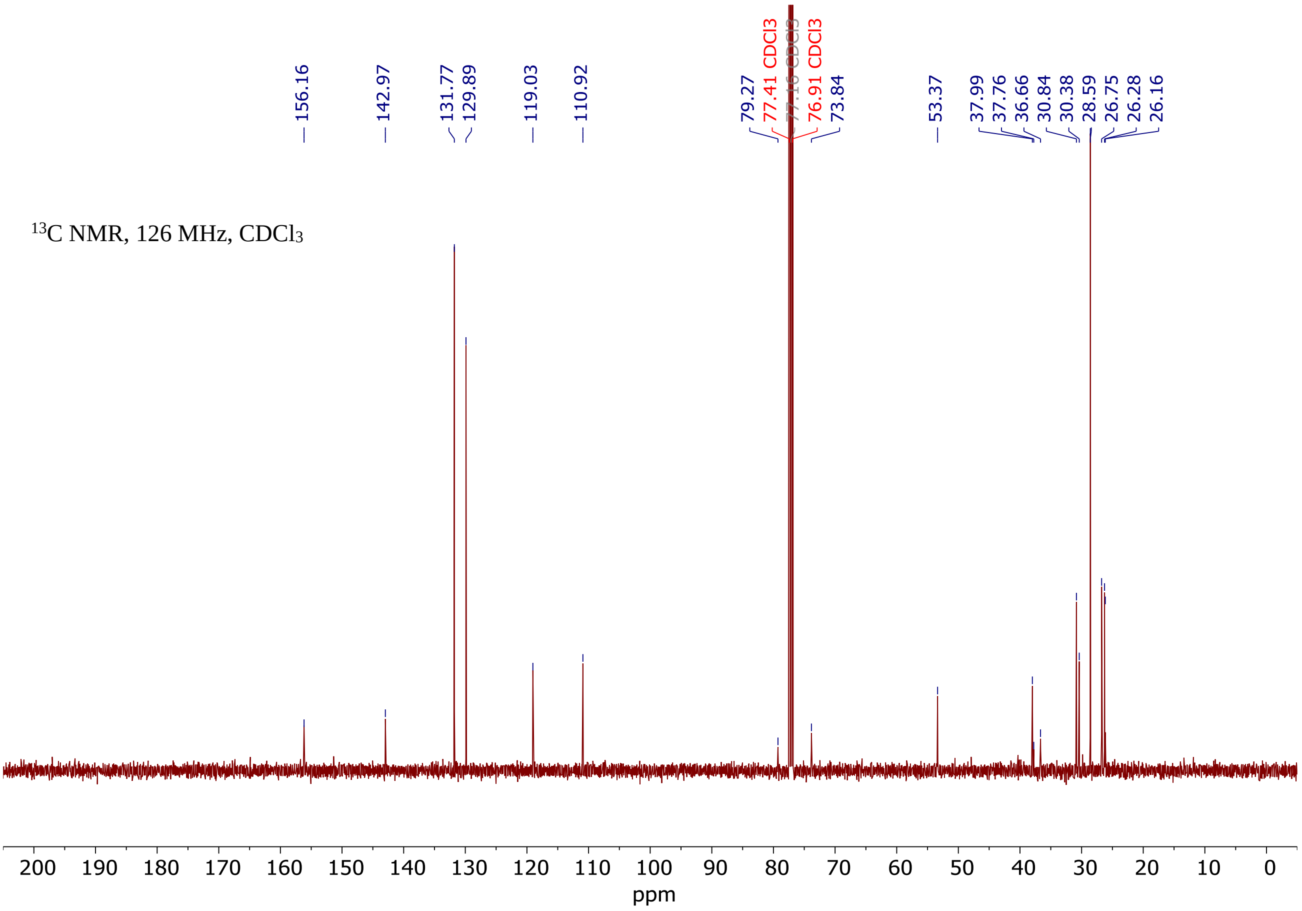

4-(Cyclohexyl((4-methoxyphenyl)amino)methyl)benzonitrile (12t)

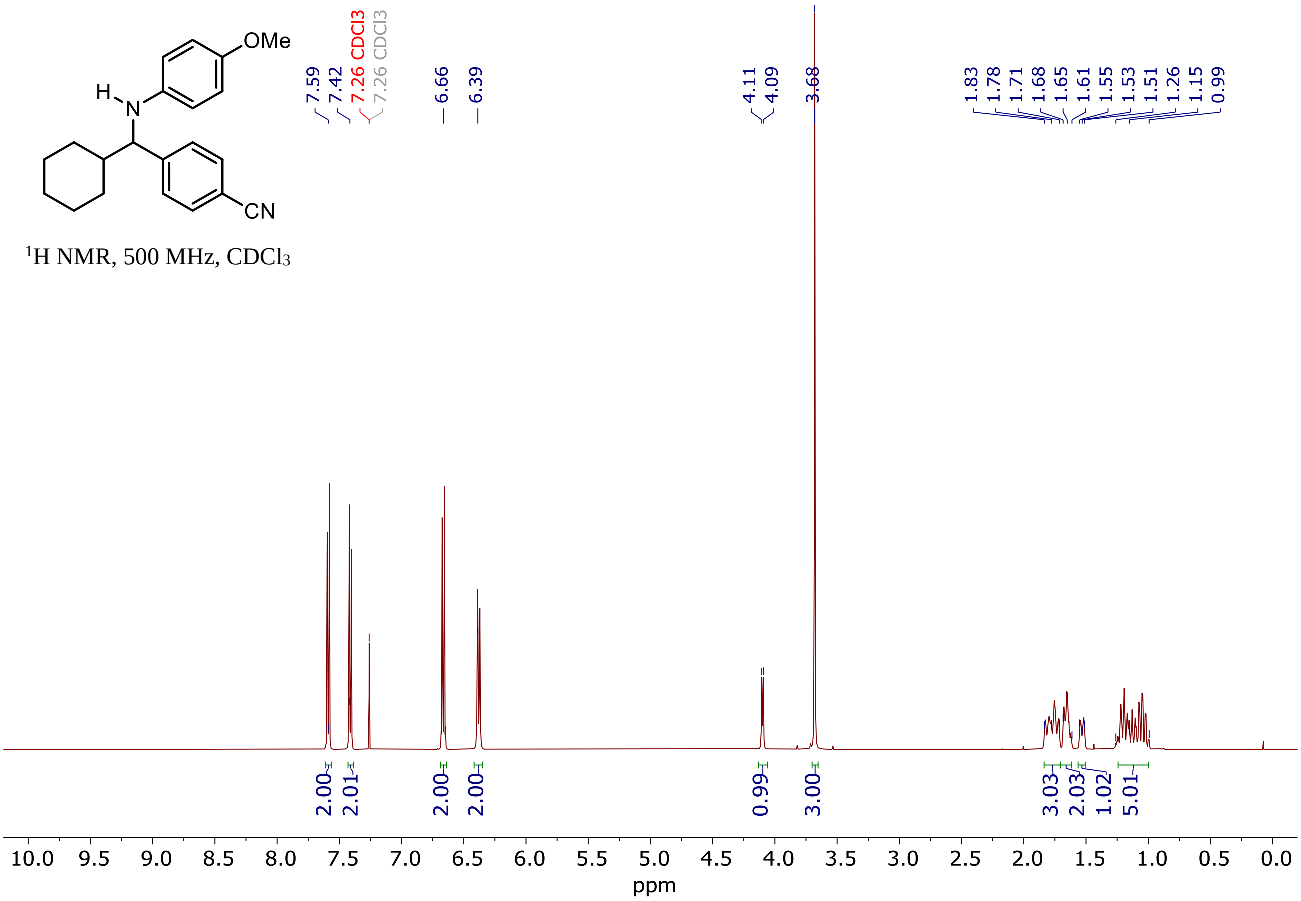

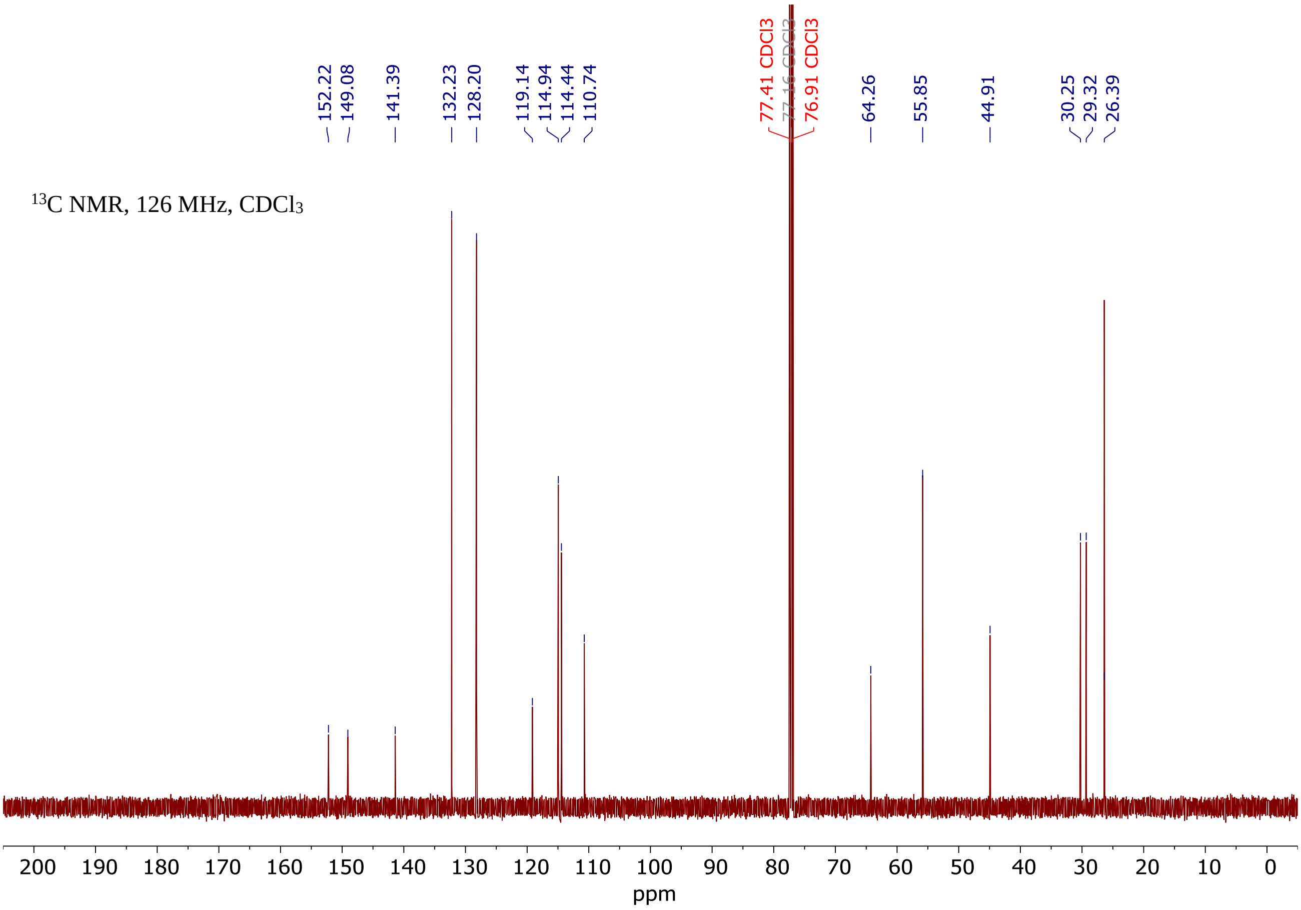

4-(4-Morpholinotetrahydro-2H-pyran-4-yl)benzonitrile (12u)

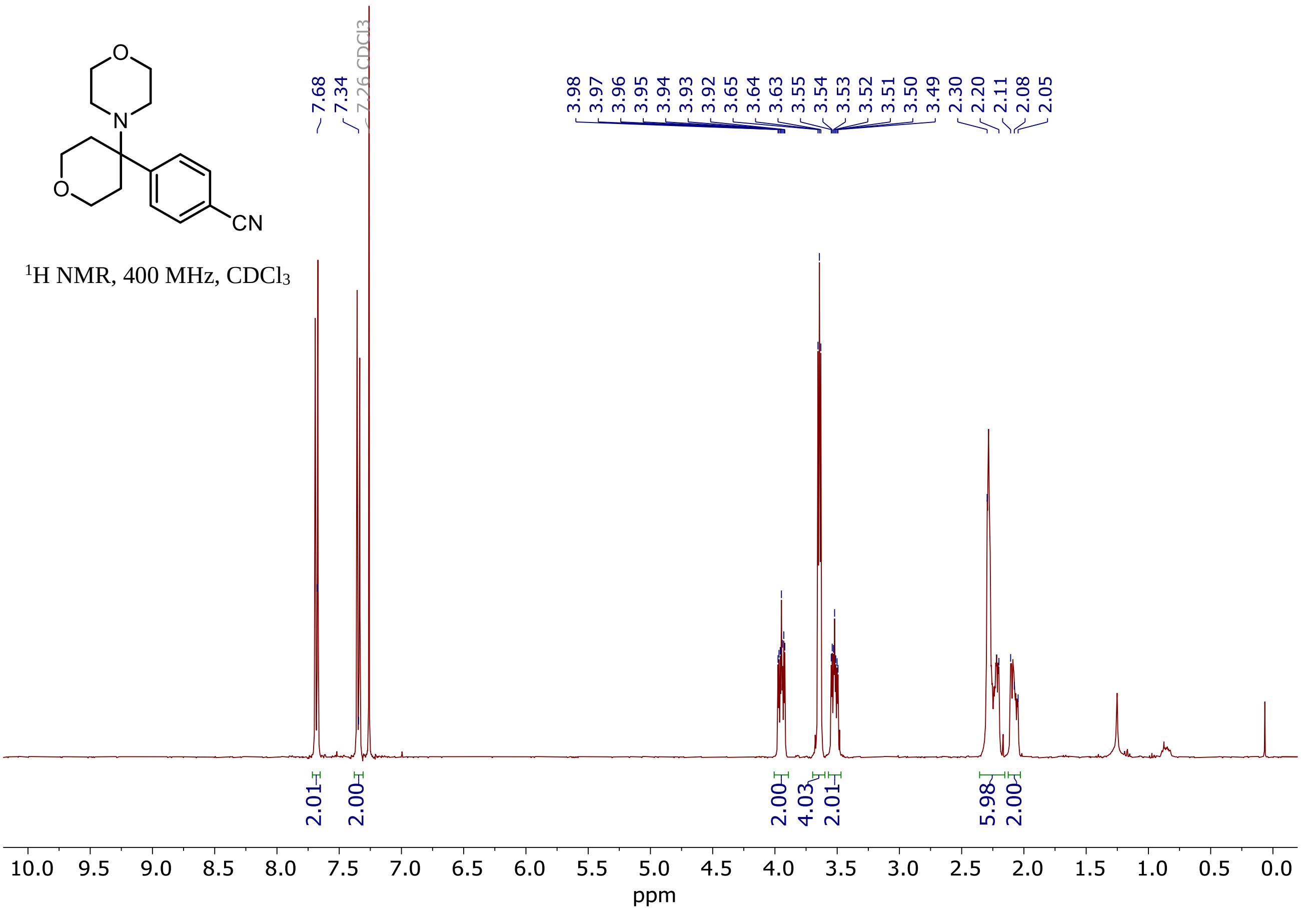

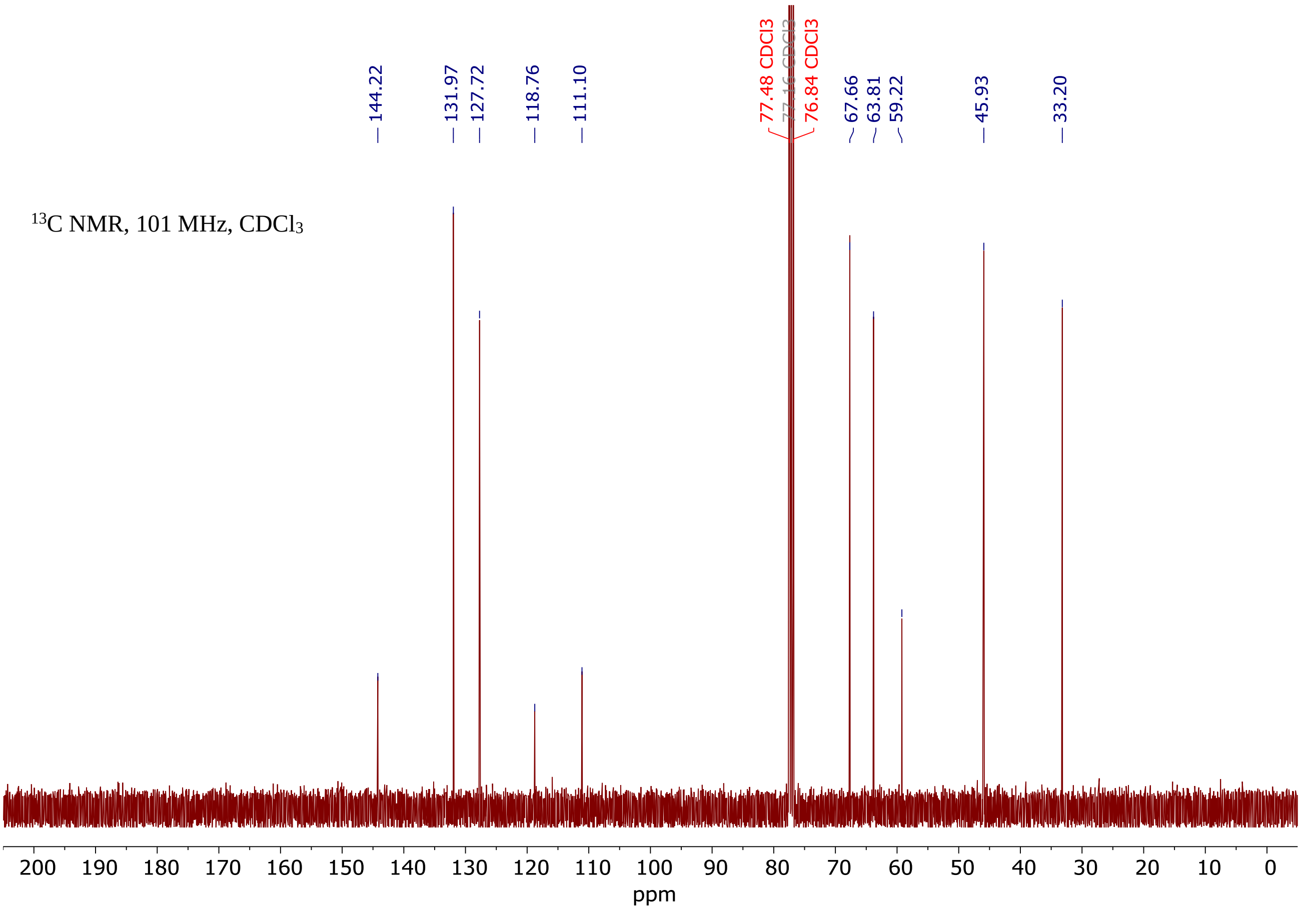

Tert-butyl 3-(4-cyanophenyl)-3-morpholinopyrrolidine-1-carboxylate (12v)

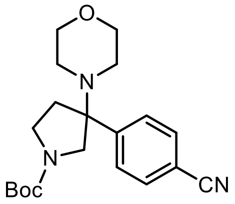

^{1}H NMR, 700 MHz, $CDCl_3$

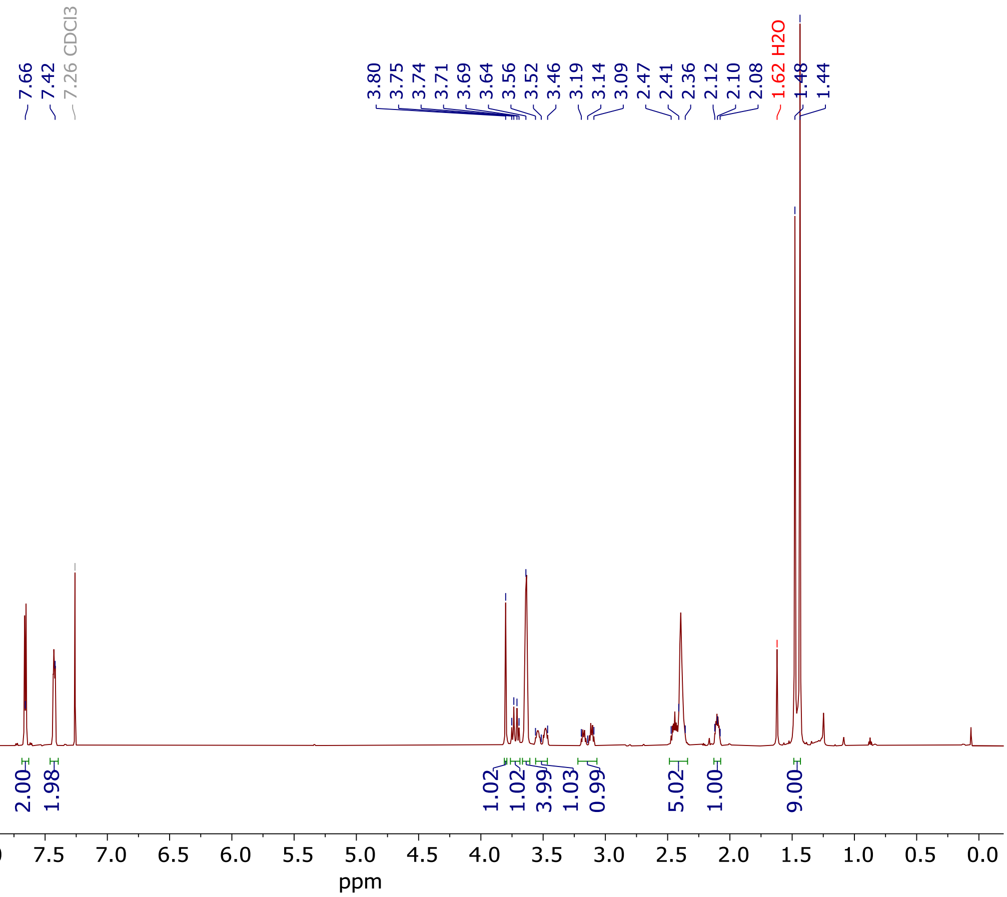

^{13}C NMR, 176 MHz, $CDCl_3$

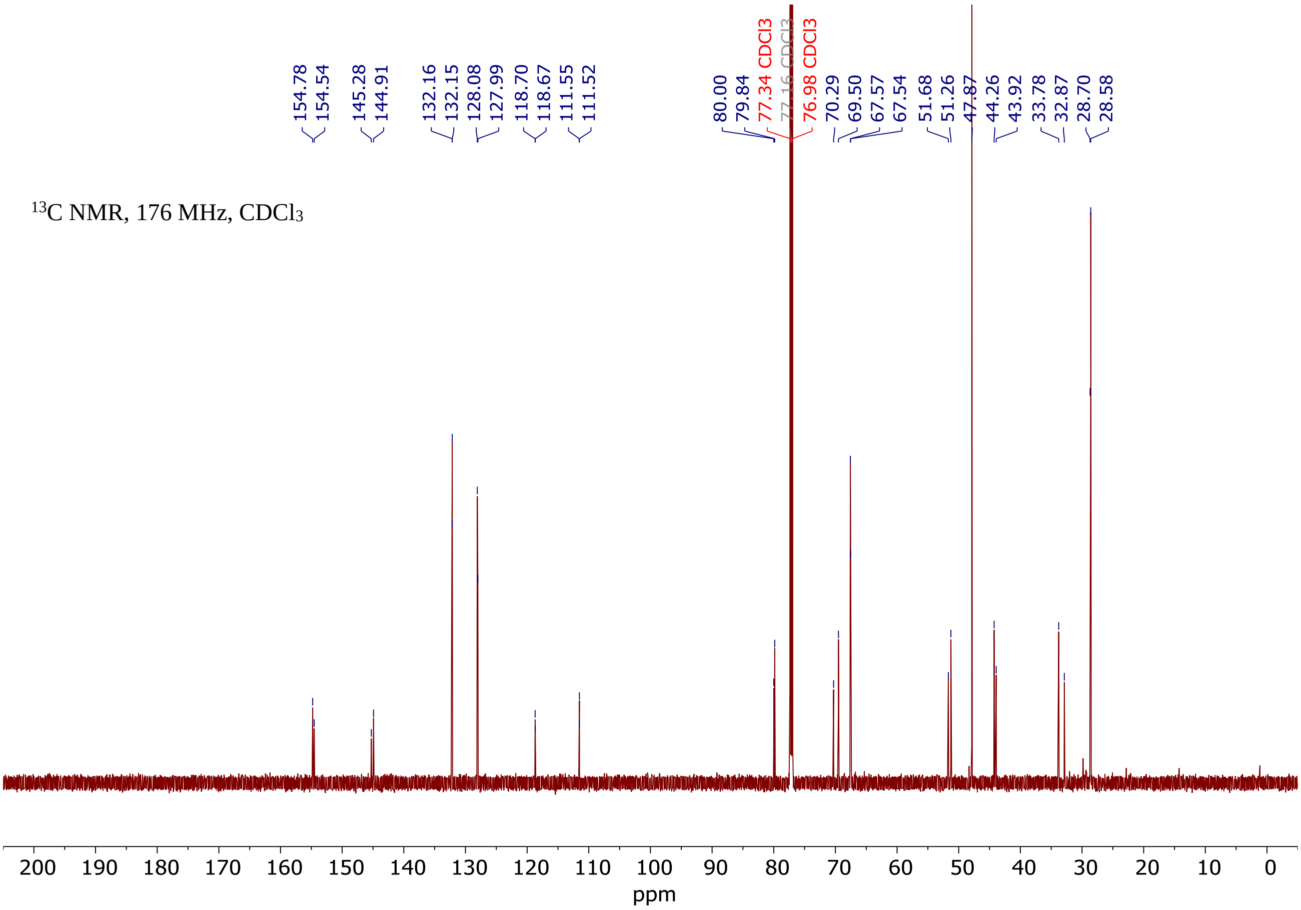

2-(1-Morpholinobutyl)benzonitrile (12w)

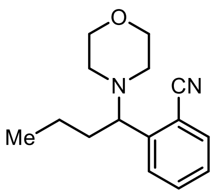

^{1}H NMR, 400 MHz, $CDCl_3$

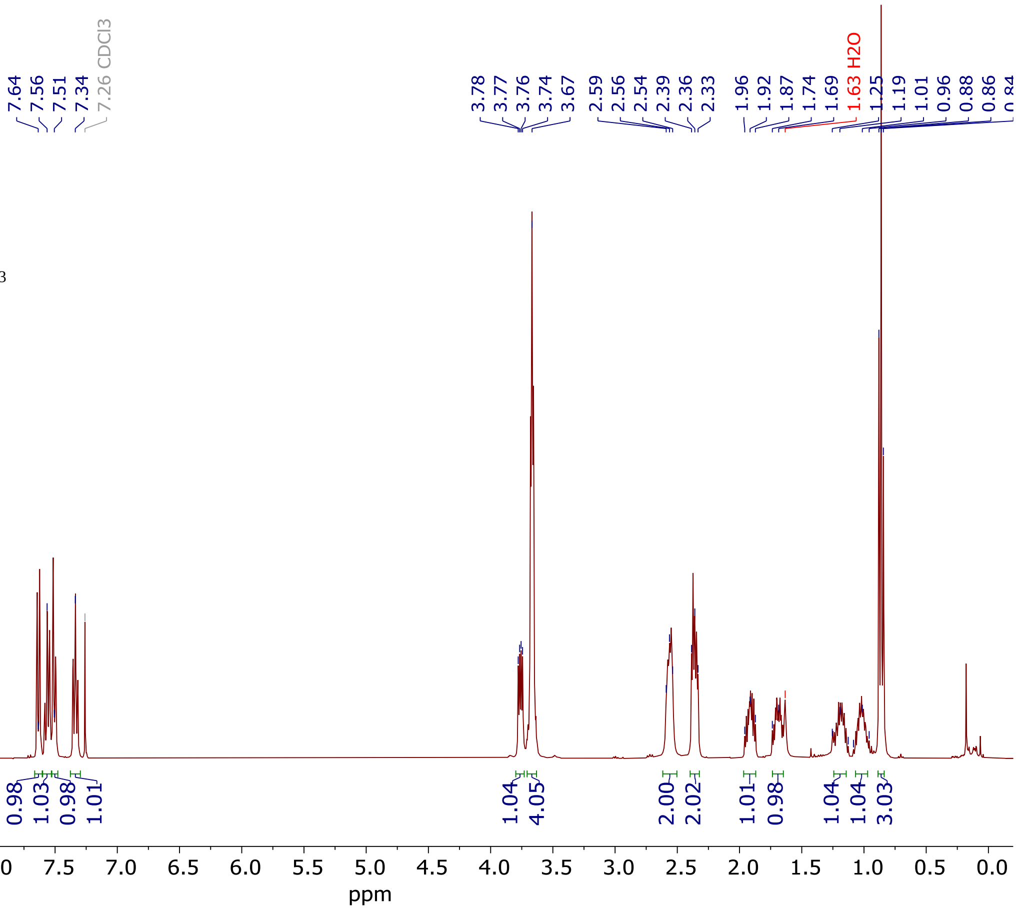

^{13}C NMR, 101 MHz, $CDCl_3$

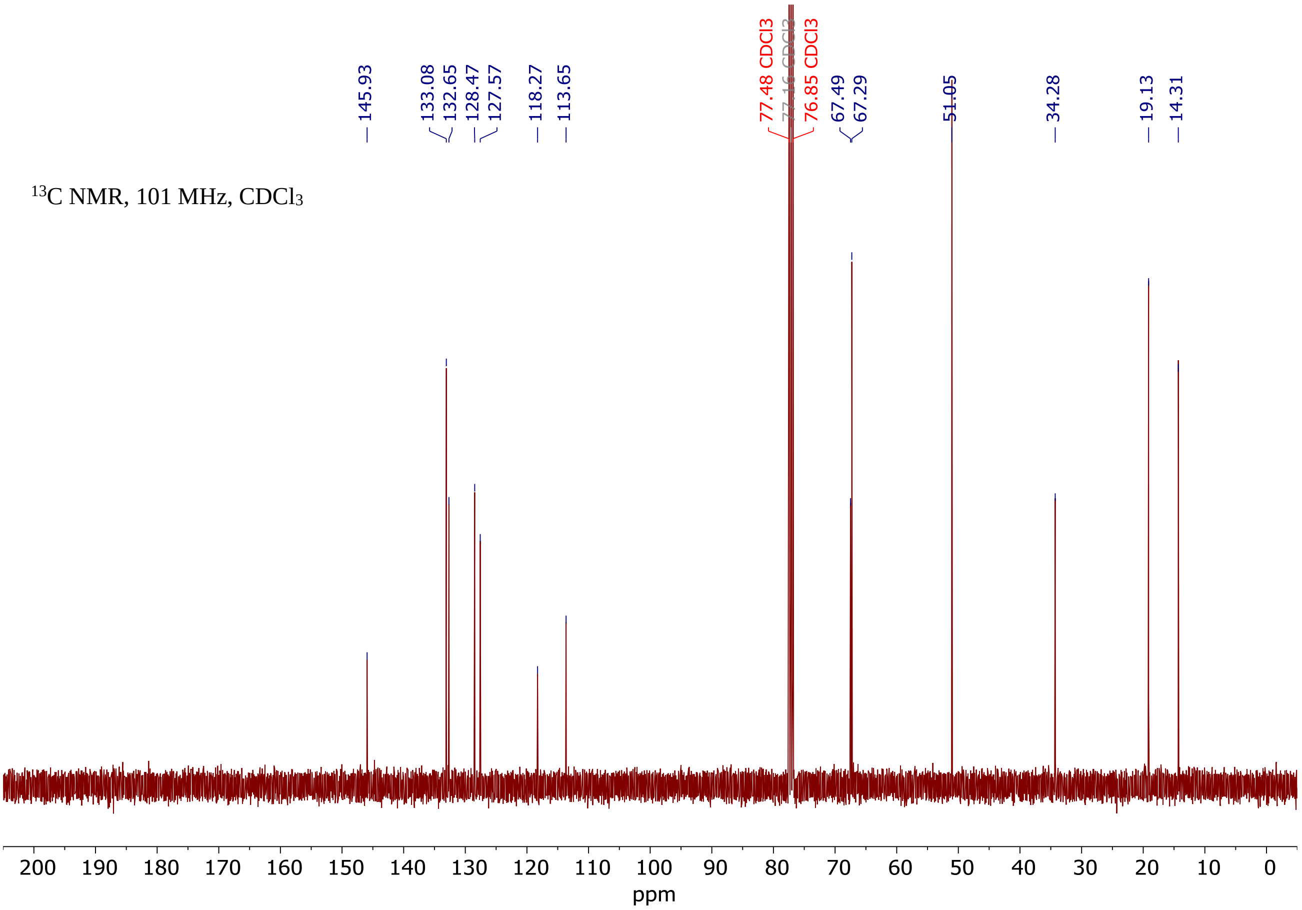

Methyl 4-(cyclohexyl(morpholino)methyl)benzoate (12x)

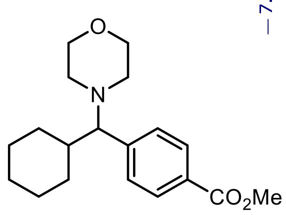

^{1}H NMR, 500 MHz, $CDCl_3$

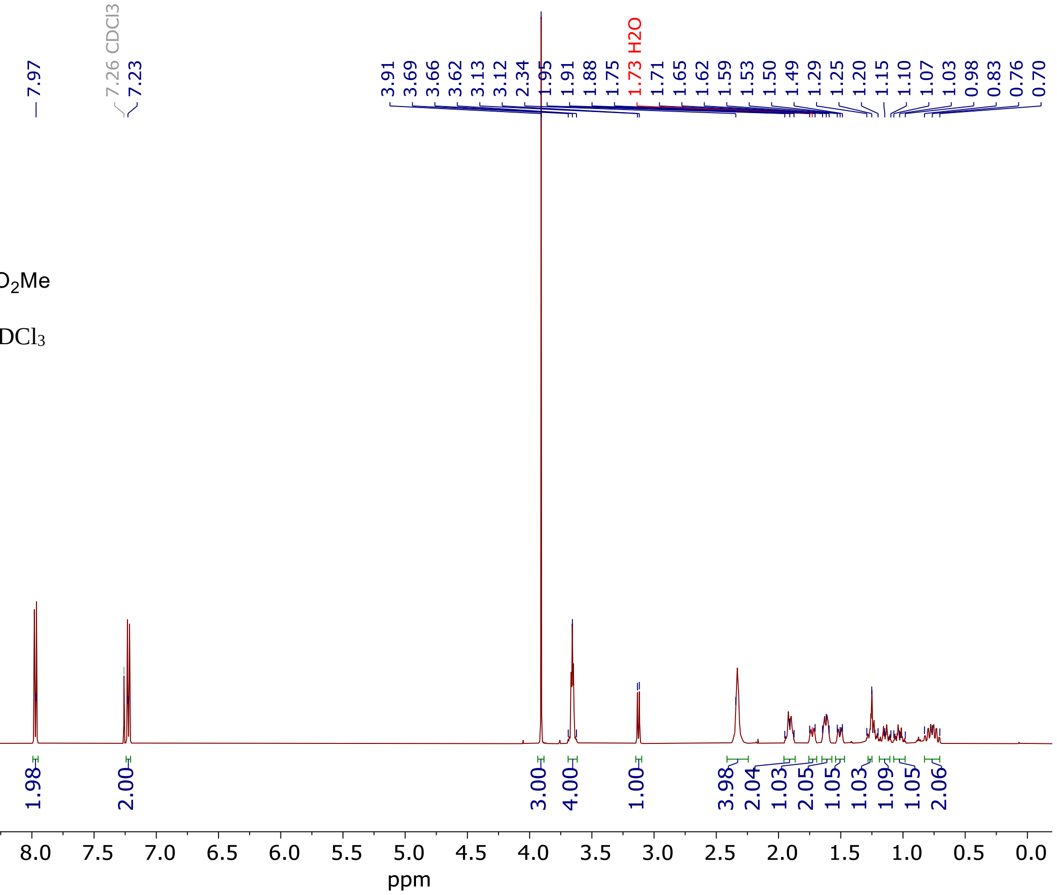

^{13}C NMR, 101 MHz, $CDCl_3$

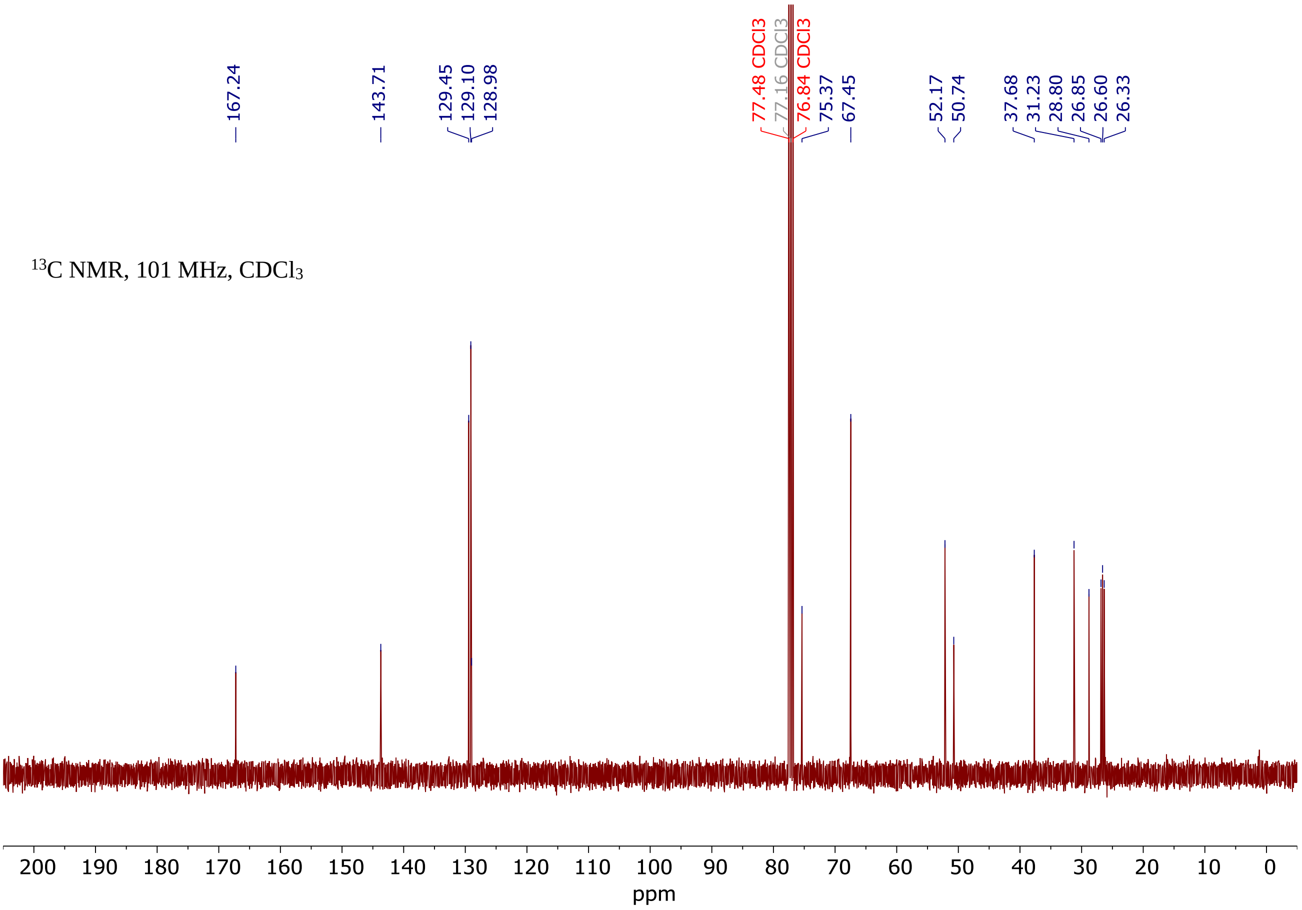

4-(Cyclohexyl(4-(phenylsulfonyl)phenyl)methyl)morpholine (12y)

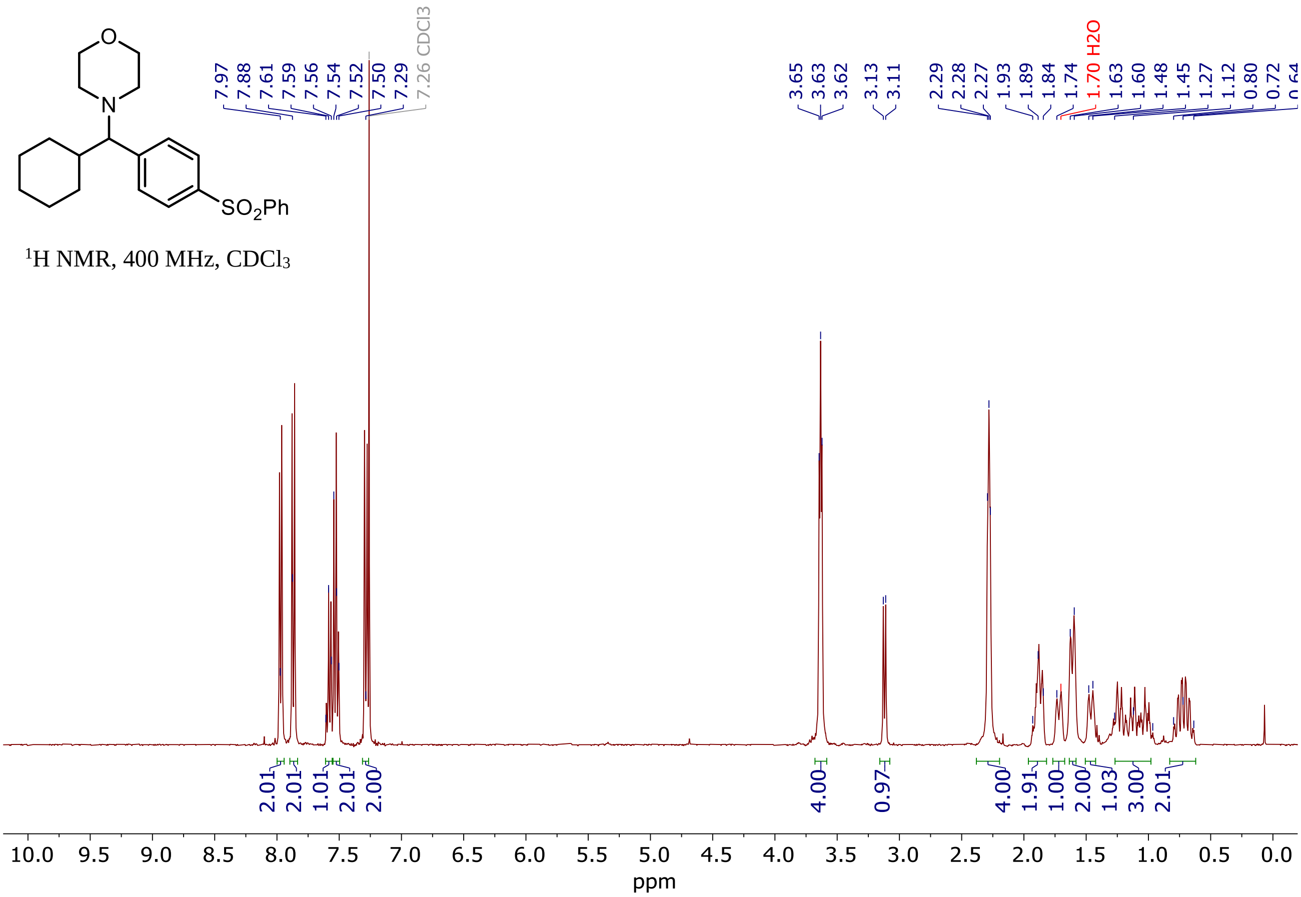

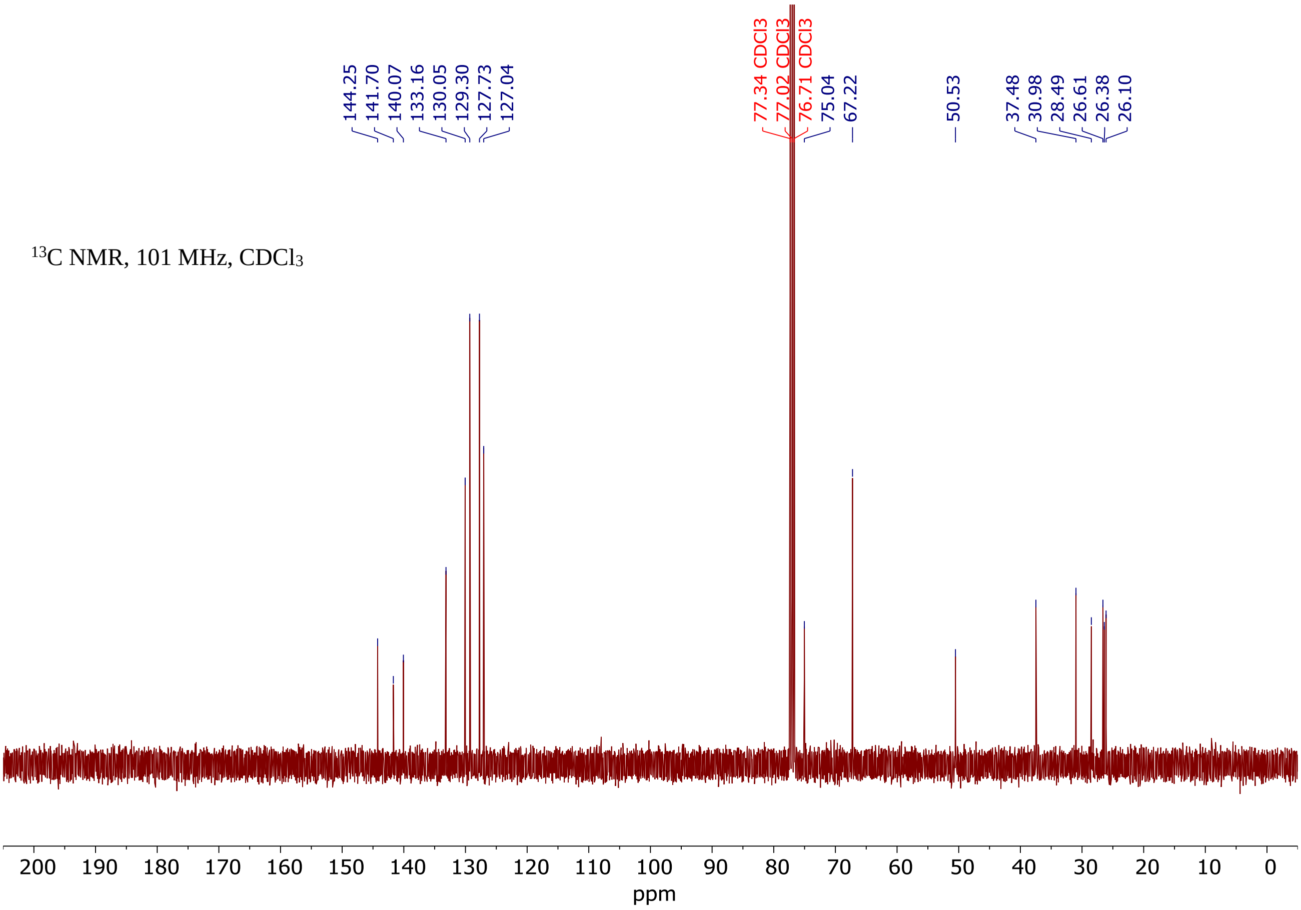

Diethyl (4-(cyclohexyl(morpholino)methyl)phenyl)phosphonate (12z)

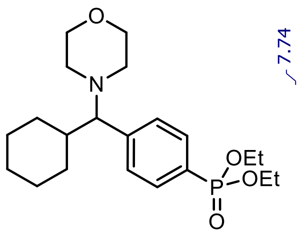

^{1}H NMR, 400 MHz, $CDCl_3$

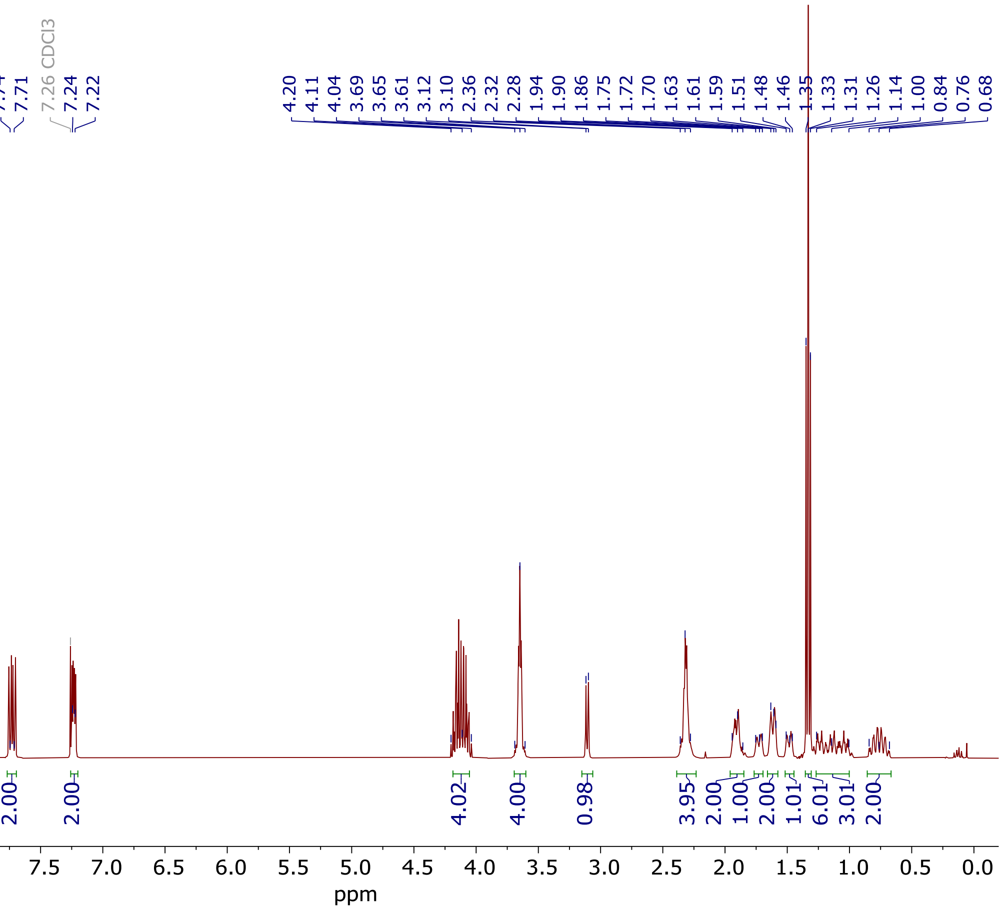

^{13}C NMR, 101 MHz, $CDCl_3$

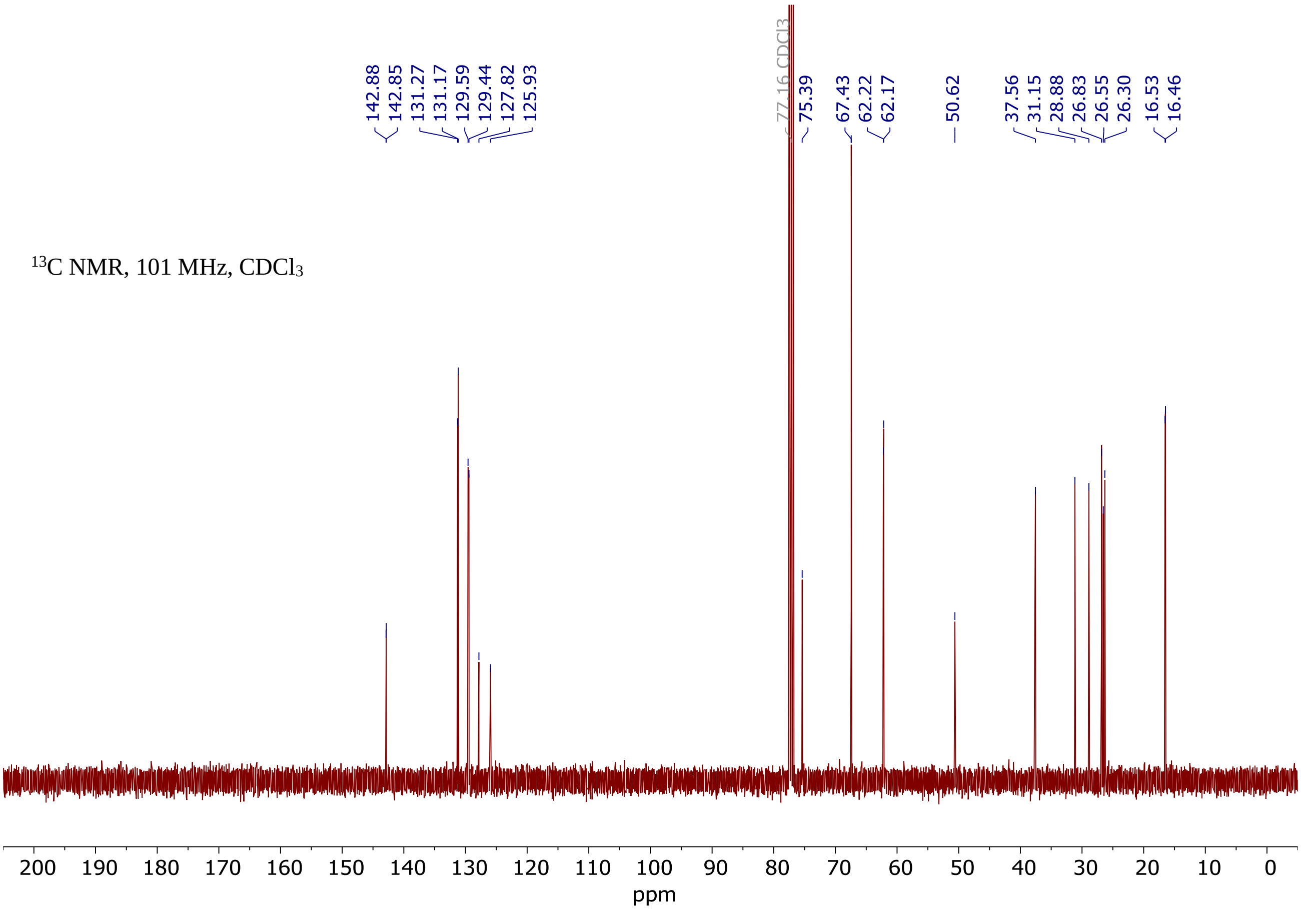

4-(Cyclohexyl(pyridin-4-yl)methyl)morpholine (12aa)

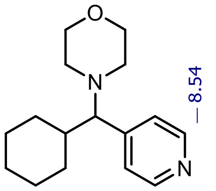

^{1}H NMR, 500 MHz, $CDCl_3$

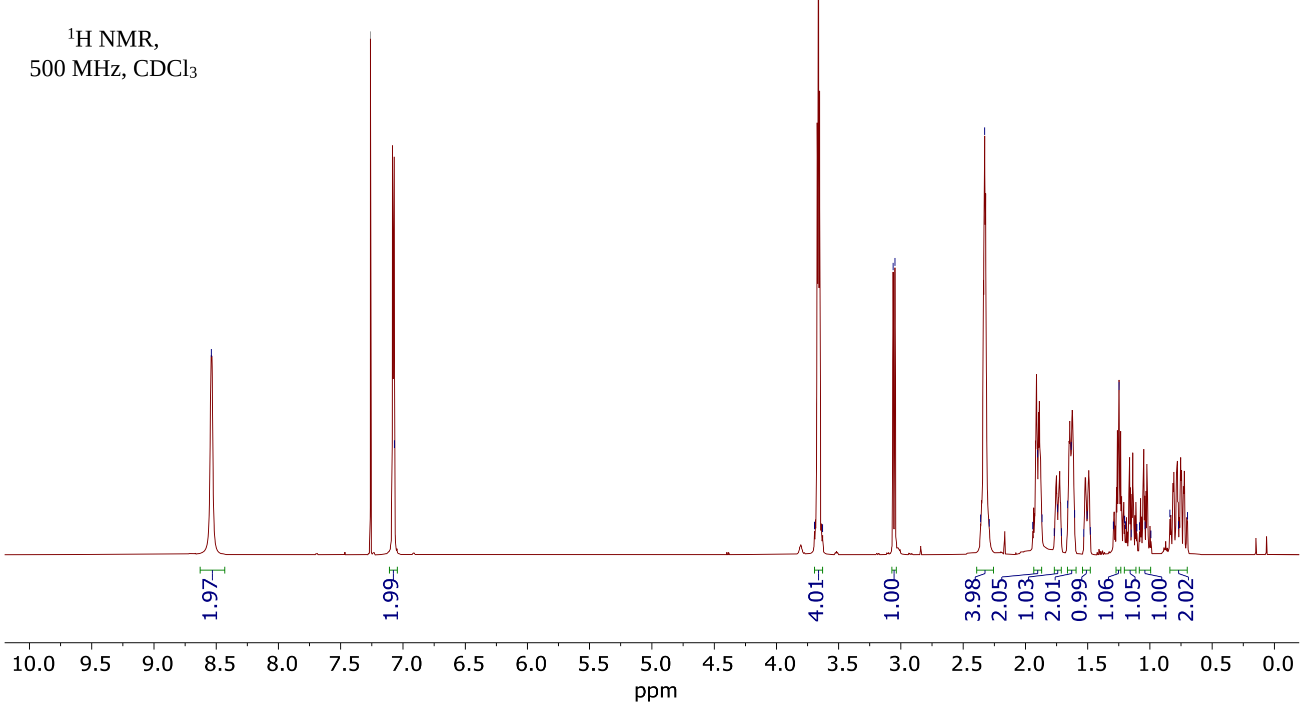

^{13}C NMR, 126 MHz, $CDCl_3$

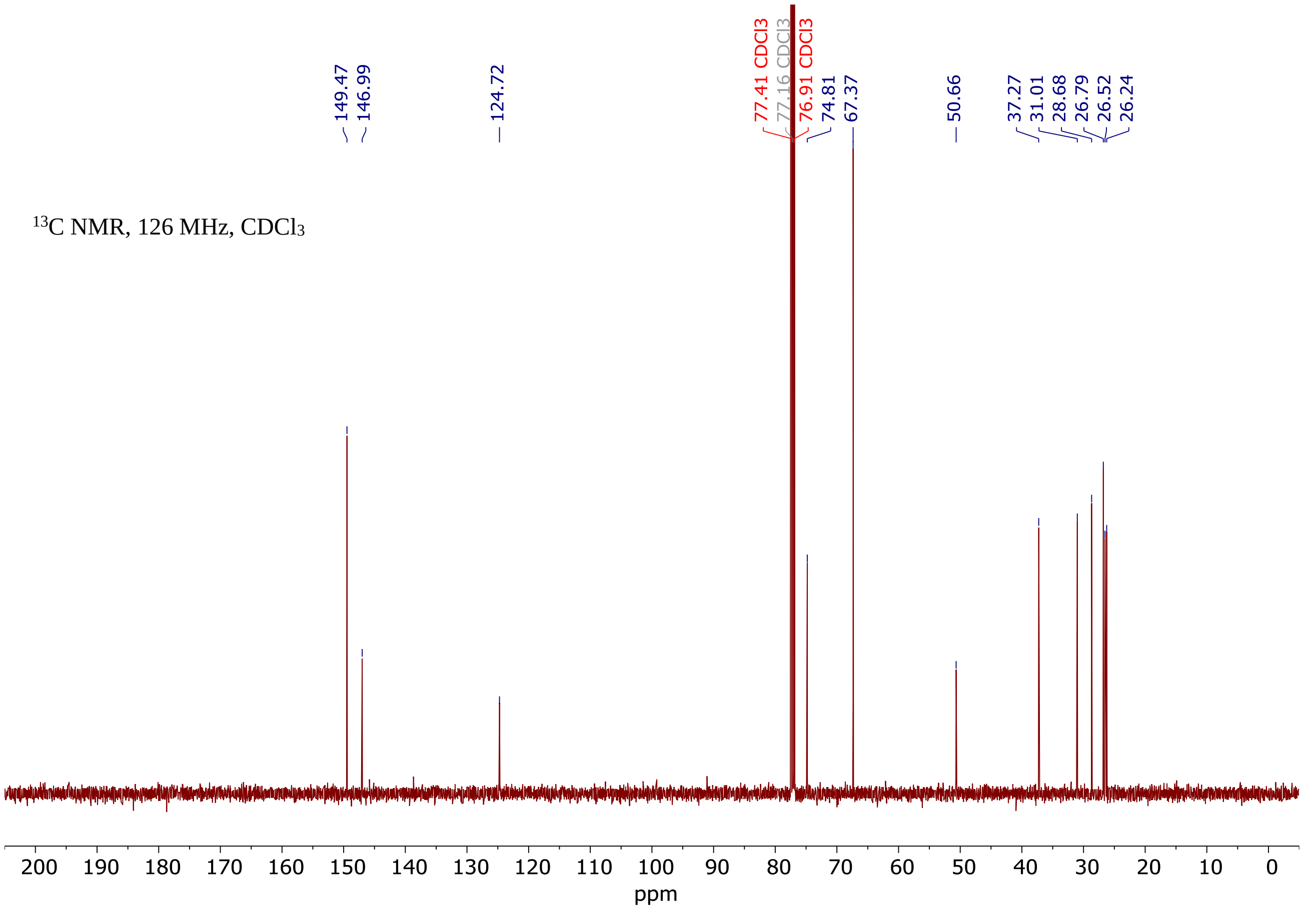

4-(Cyclohexyl(2,6-dimethylpyridin-4-yl)methyl)morpholine (12ab)

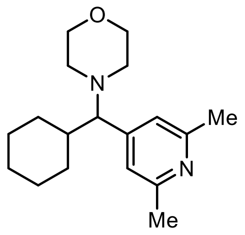

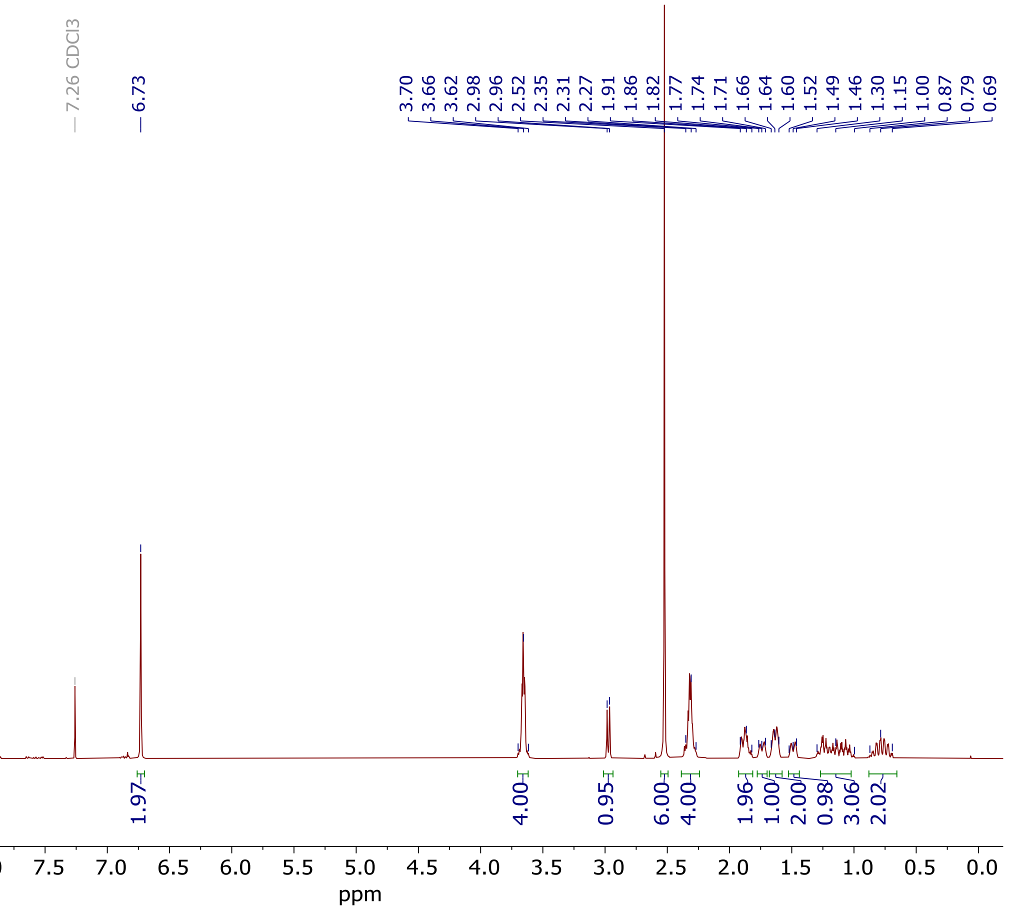

^{1}H NMR, 400 MHz, $CDCl_3$

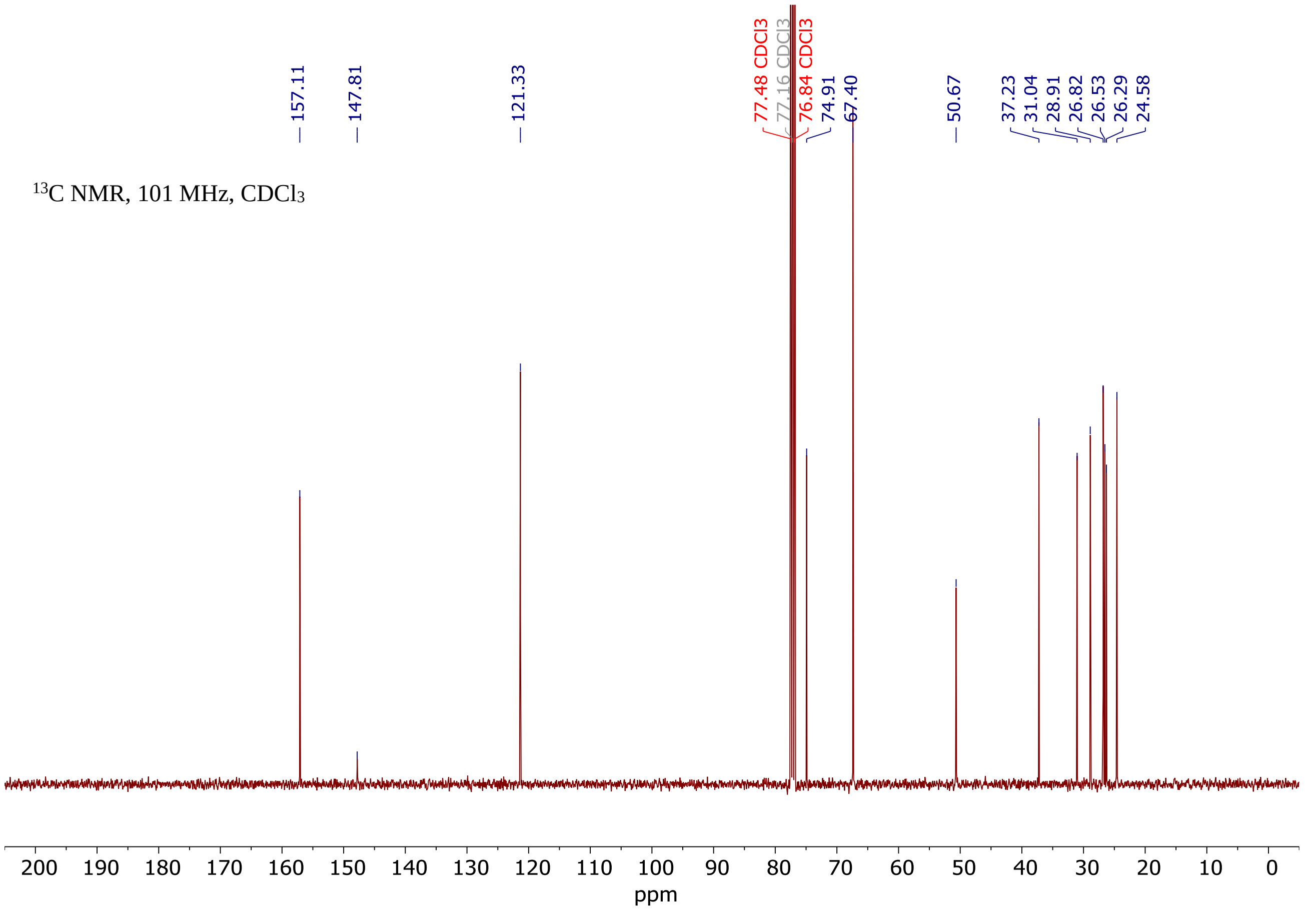

^{13}C NMR, 101 MHz, $CDCl_3$

4-(Cyclohexyl(3-methylpyridin-4-yl)methyl)morpholine (12ac)

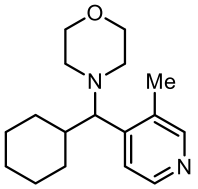

^{1}H NMR,
400 MHz, $CDCl_3$

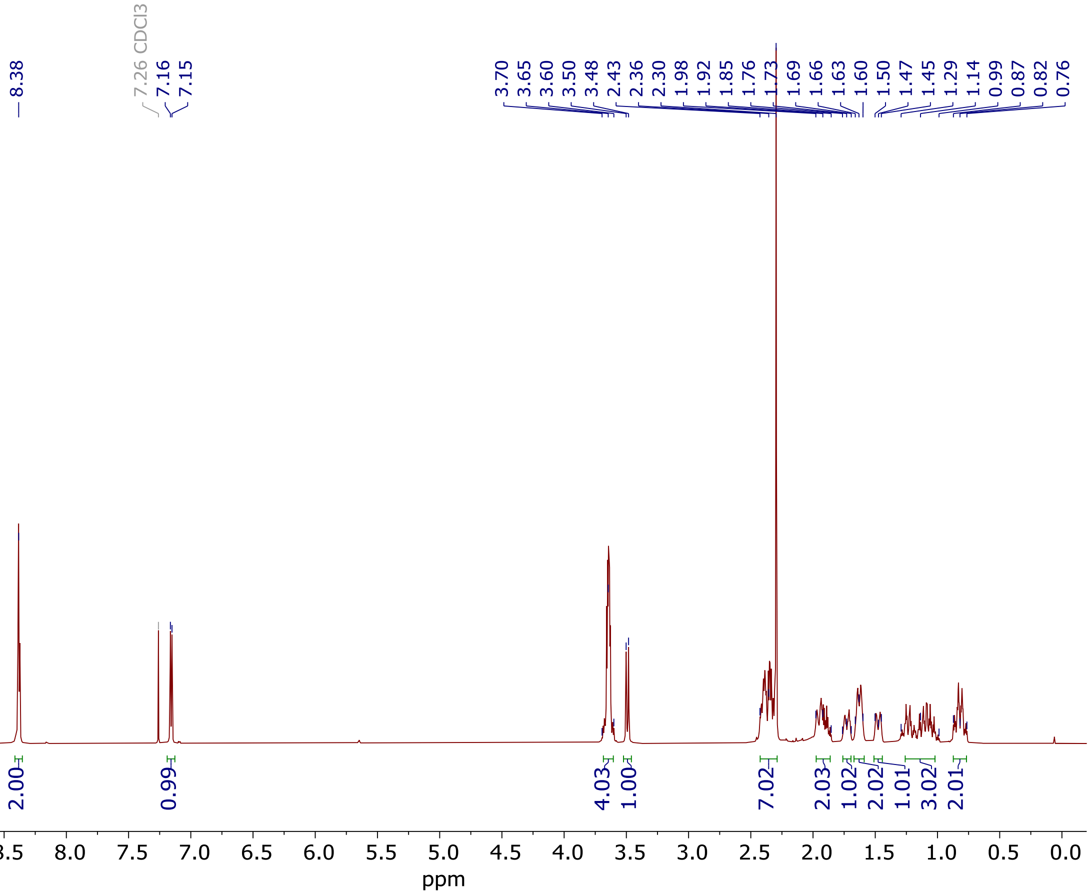

^{13}C NMR, 101 MHz, $CDCl_3$

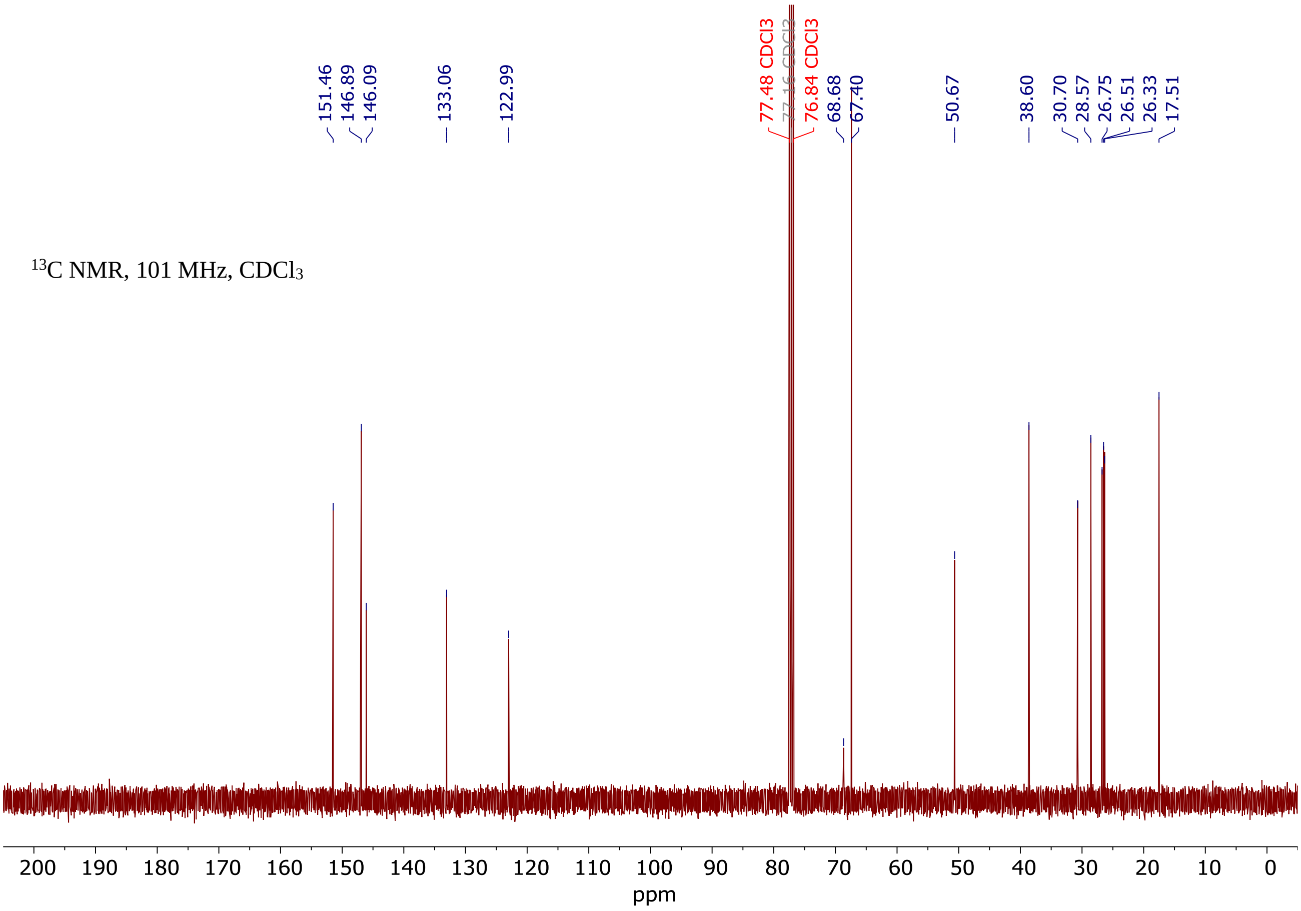

(4-(Cyclohexyl(morpholino)methyl)pyridin-2-yl)methanol (12ad)

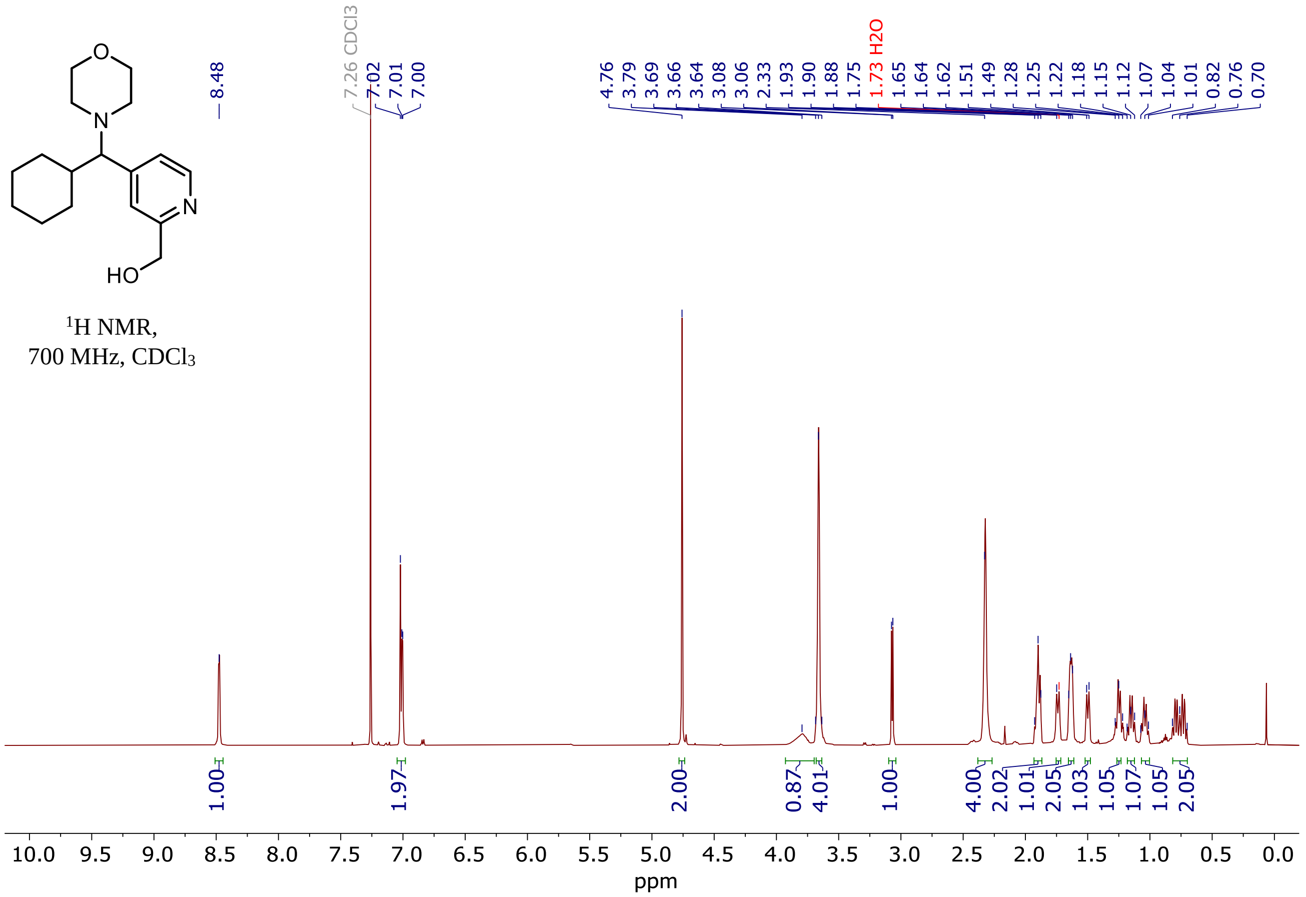

^{1}H NMR,
700 MHz, $CDCl_3$

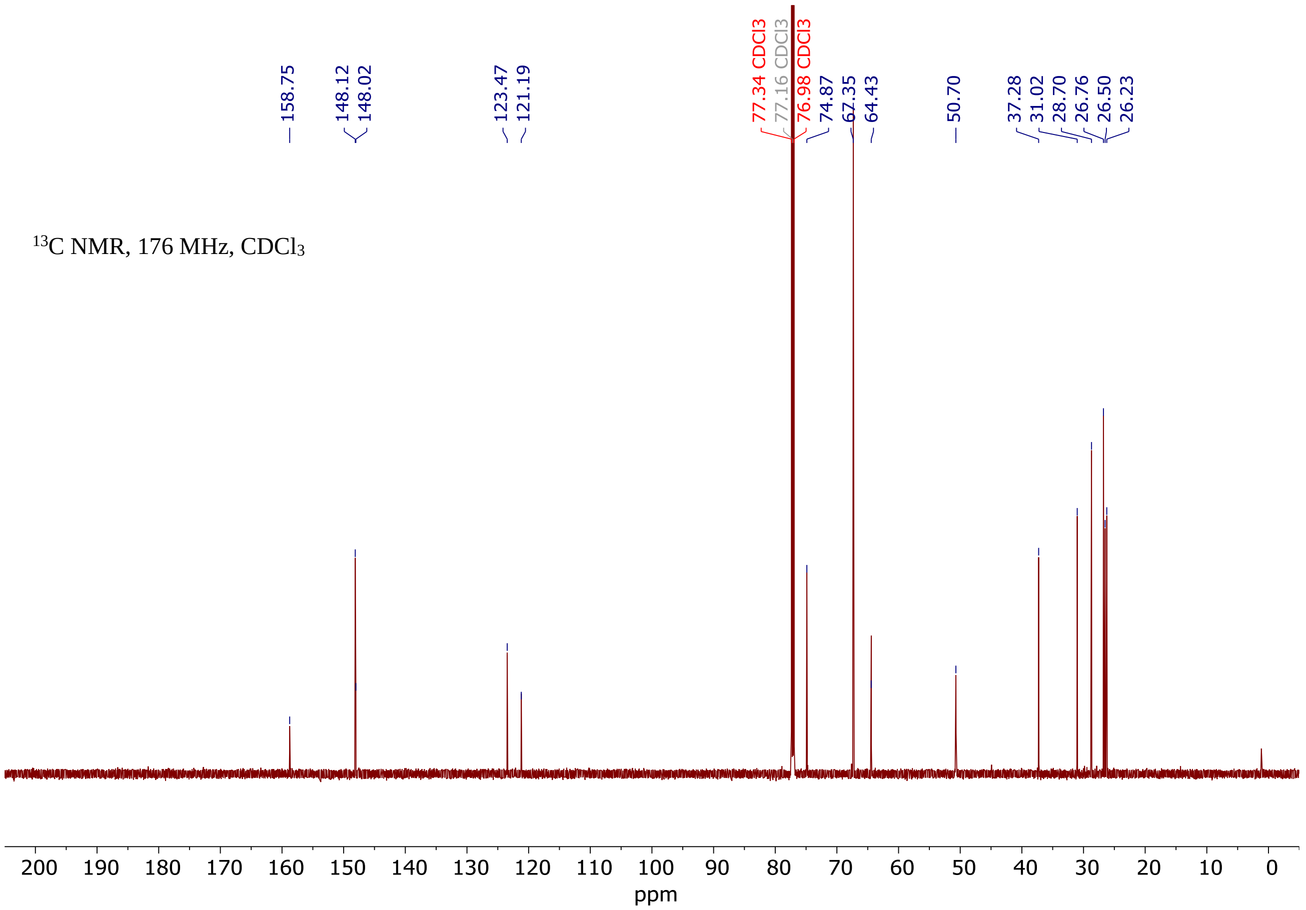

^{13}C NMR, 176 MHz, $CDCl_3$

4-Benzylbenzonitrile (19a)

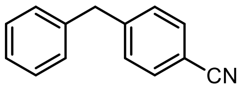

^{1}H NMR, 400 MHz, $CDCl_3$

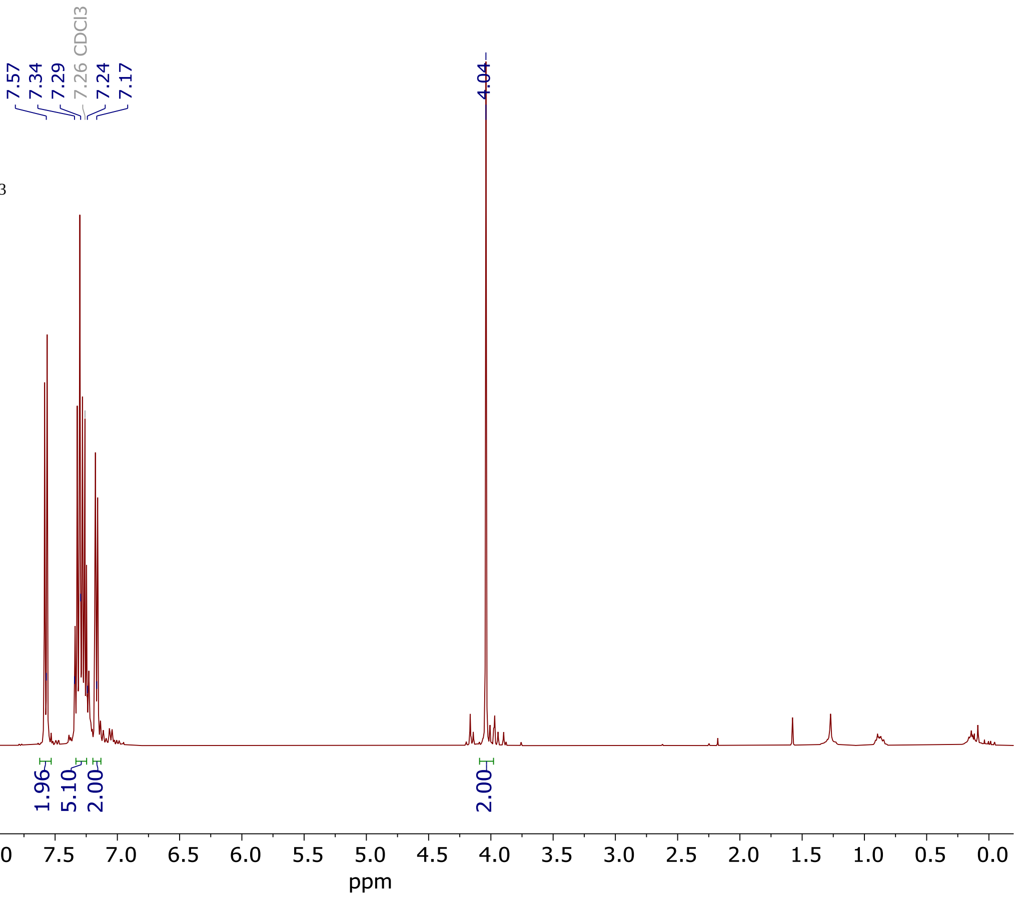

^{13}C NMR, 101 MHz, $CDCl_3$

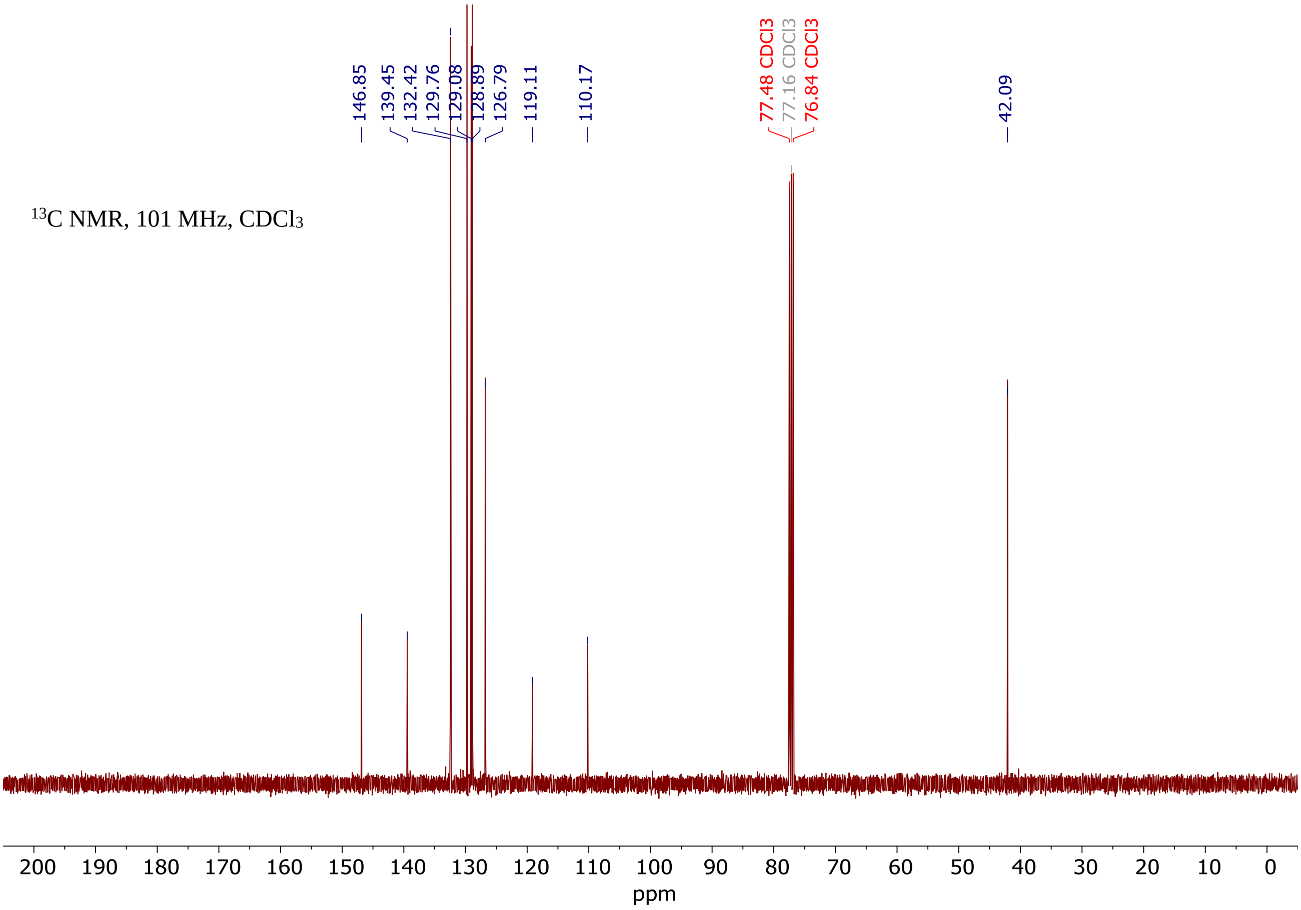

4-(Cyclohex-3-en-1-ylmethyl)benzonitrile (19b)

^{1}H NMR, 500 MHz, $CDCl_3$

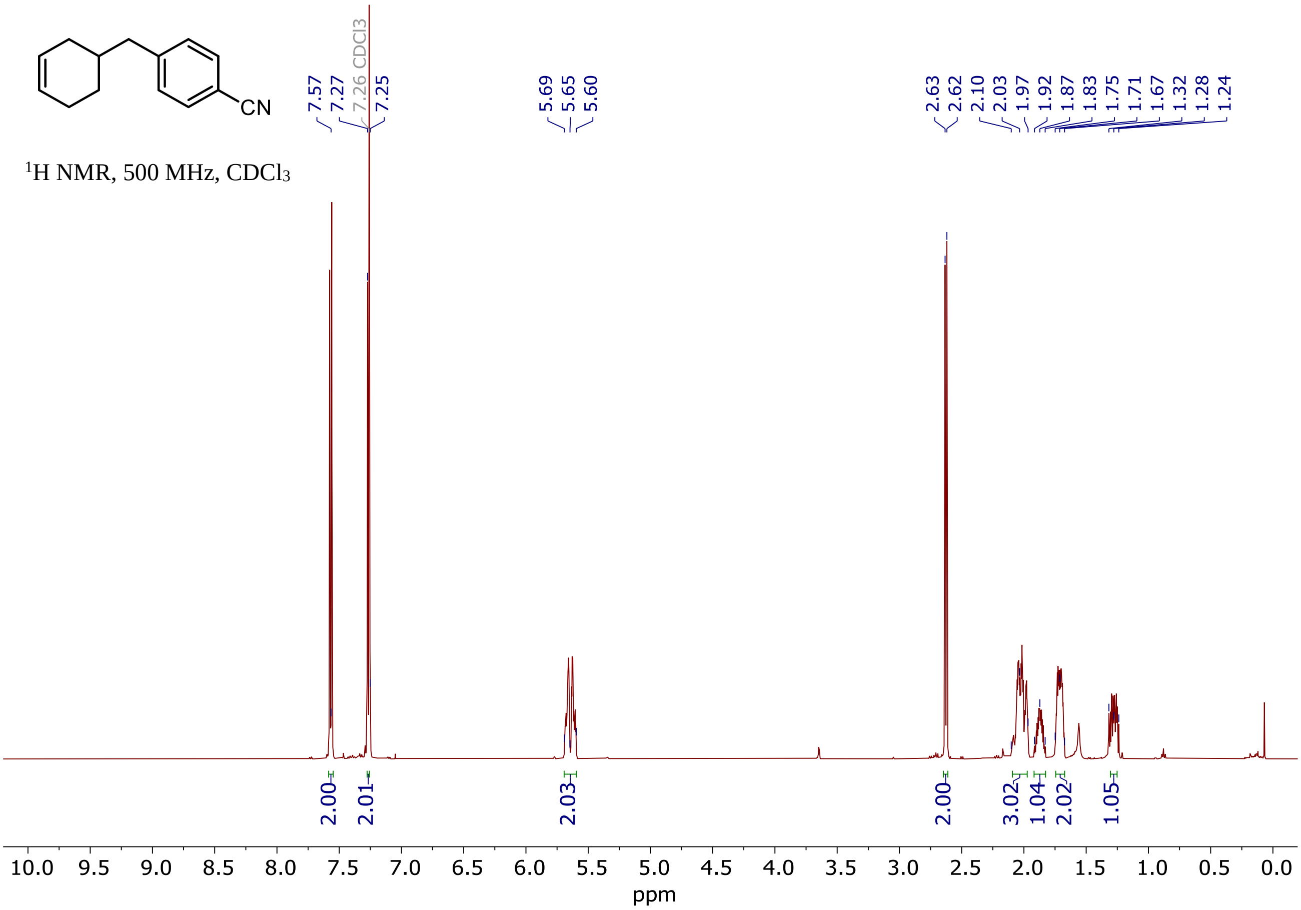

^{13}C NMR, 126 MHz, $CDCl_3$

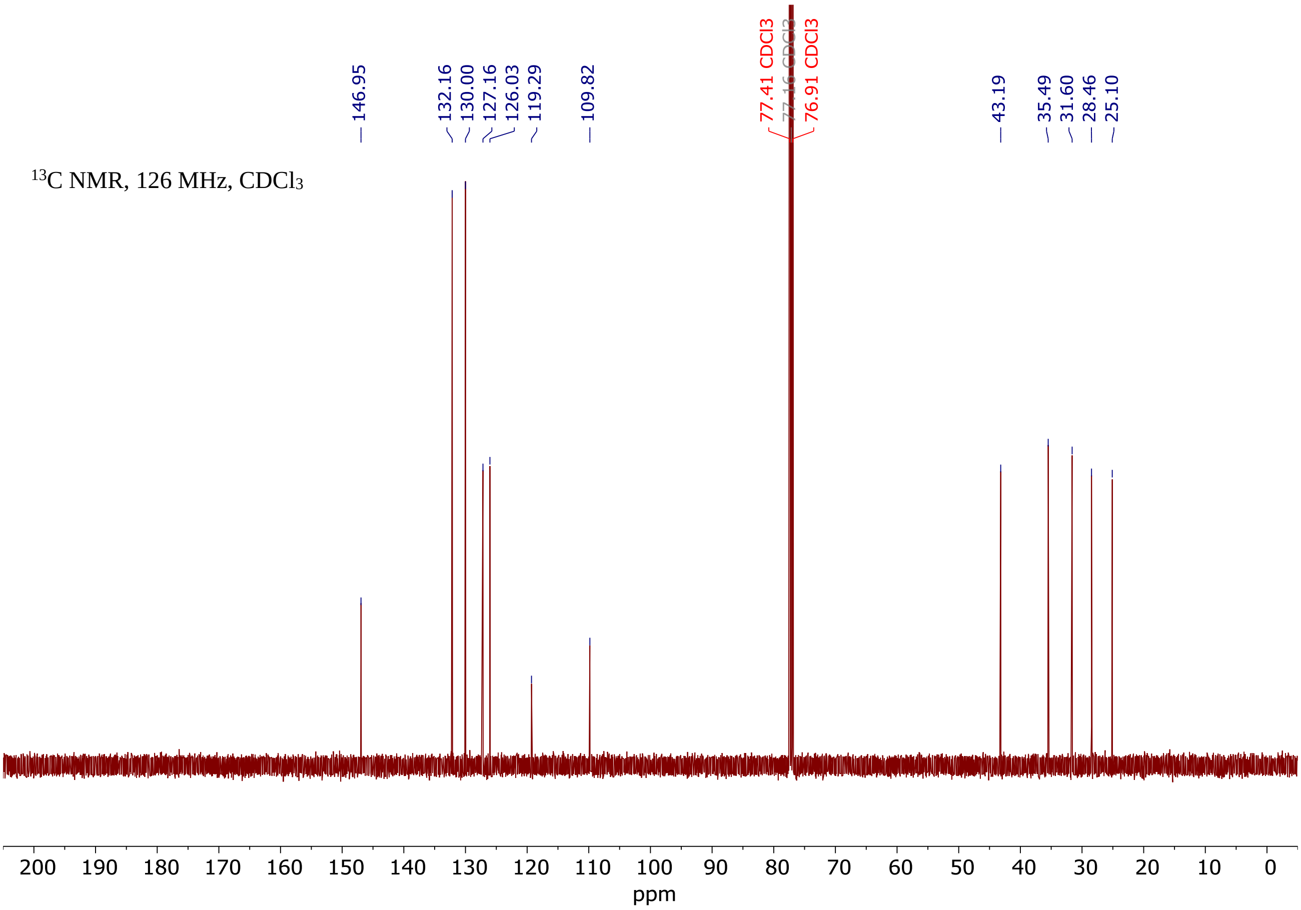

Methyl 4-(4-cyanophenyl)butanoate (19c)

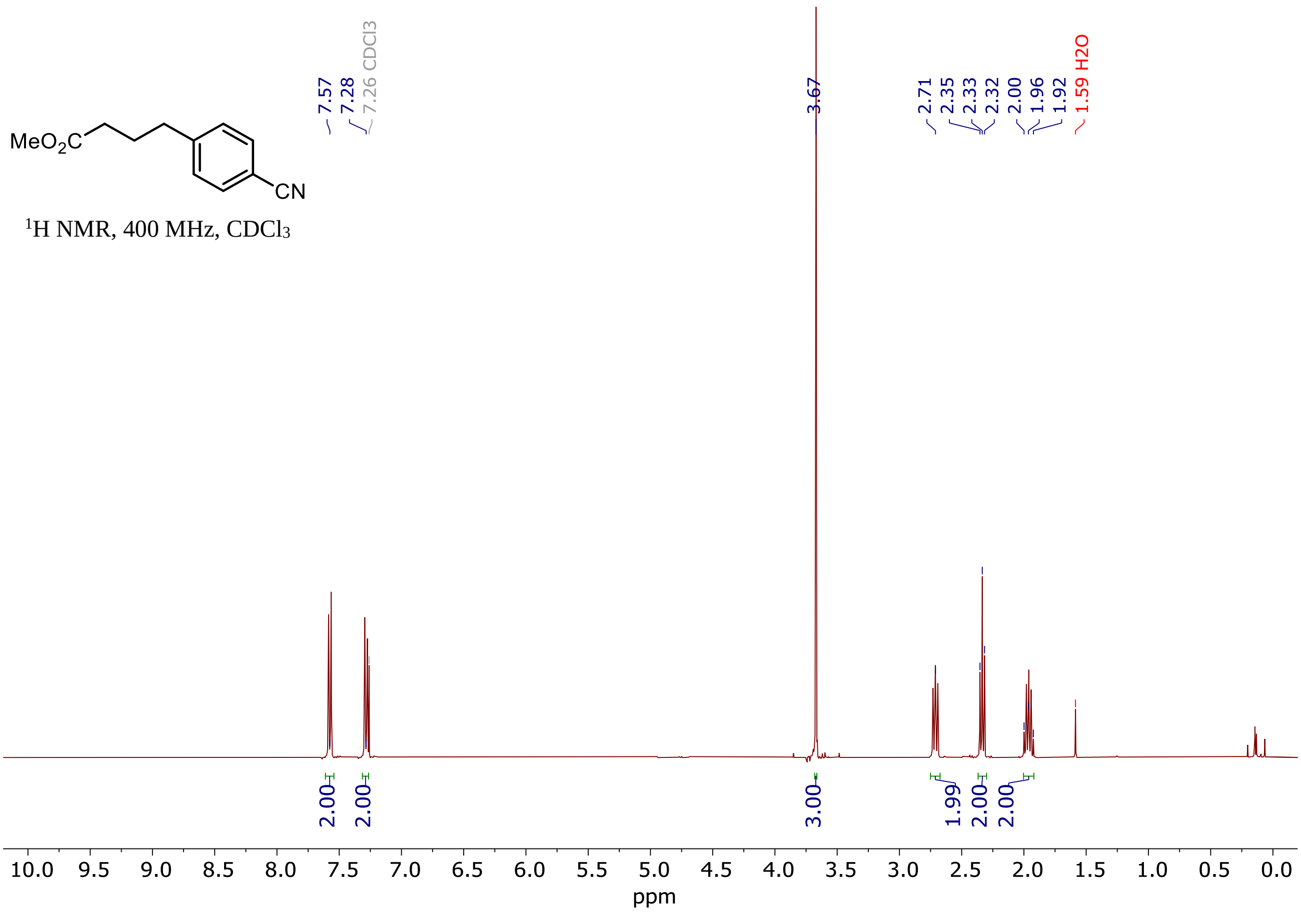

^{1}H NMR, 400 MHz, $CDCl_3$

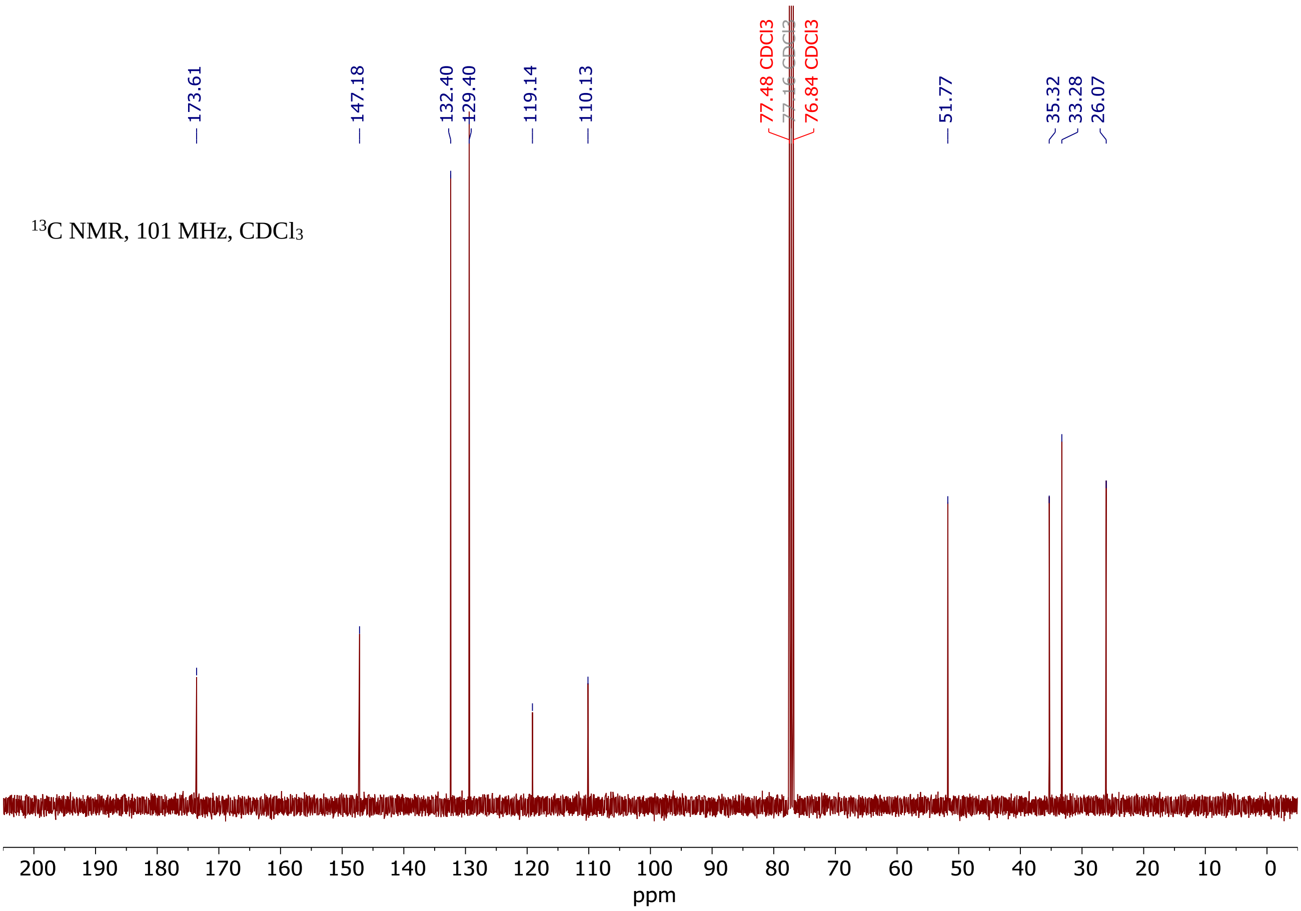

^{13}C NMR, 101 MHz, $CDCl_3$

Tert-butyl 3-(4-cyanobenzyl)azetidine-1-carboxylate (19d)

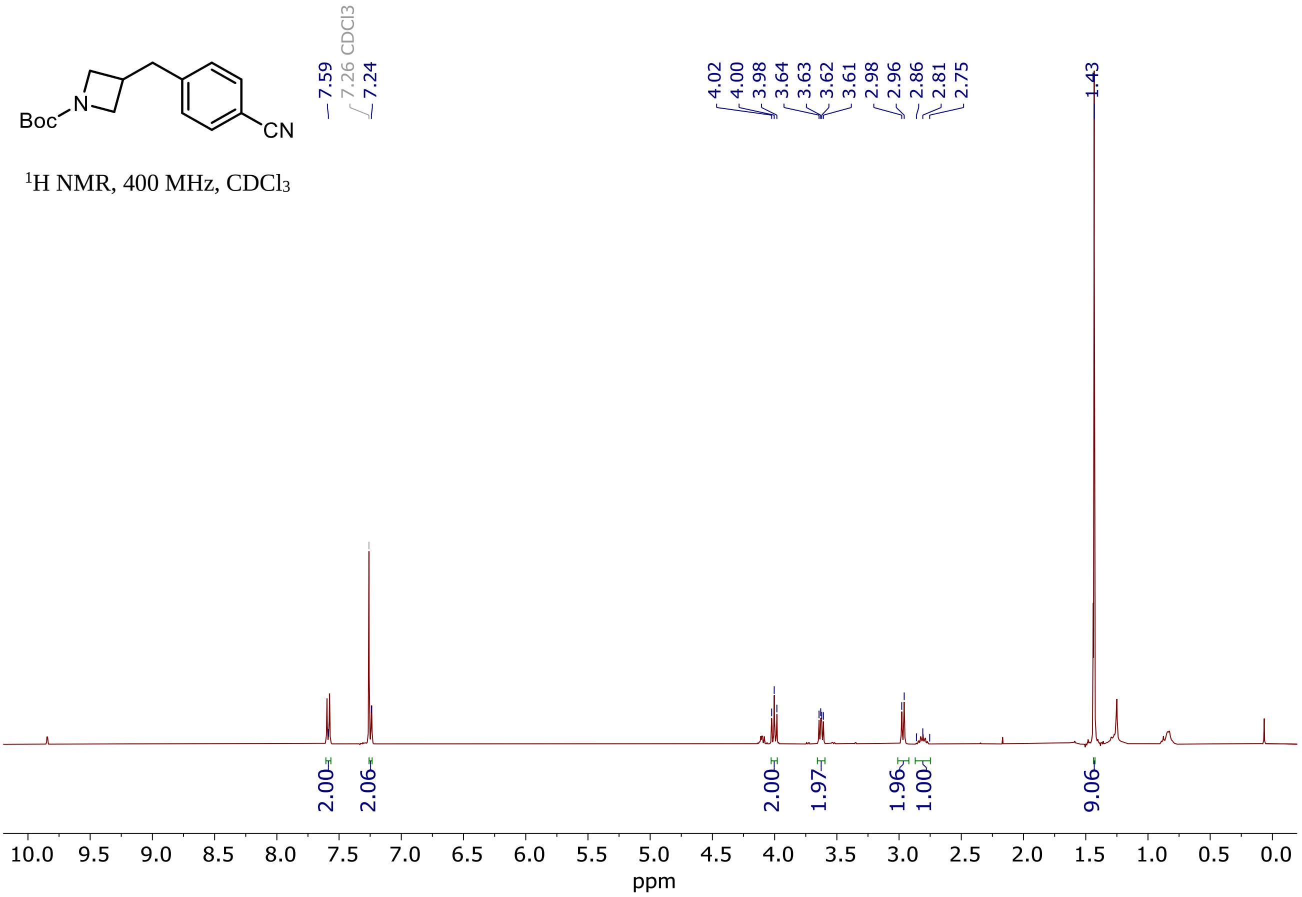

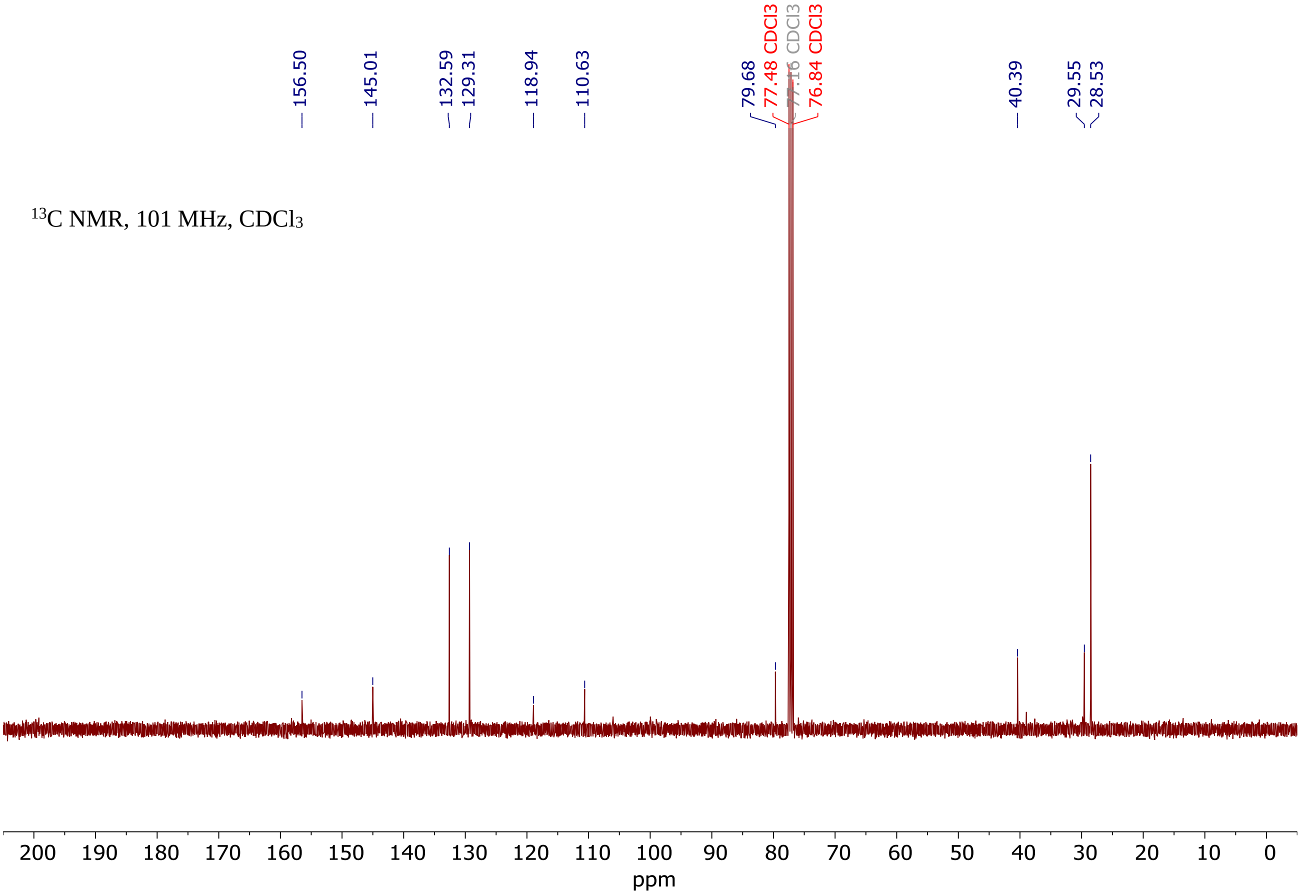

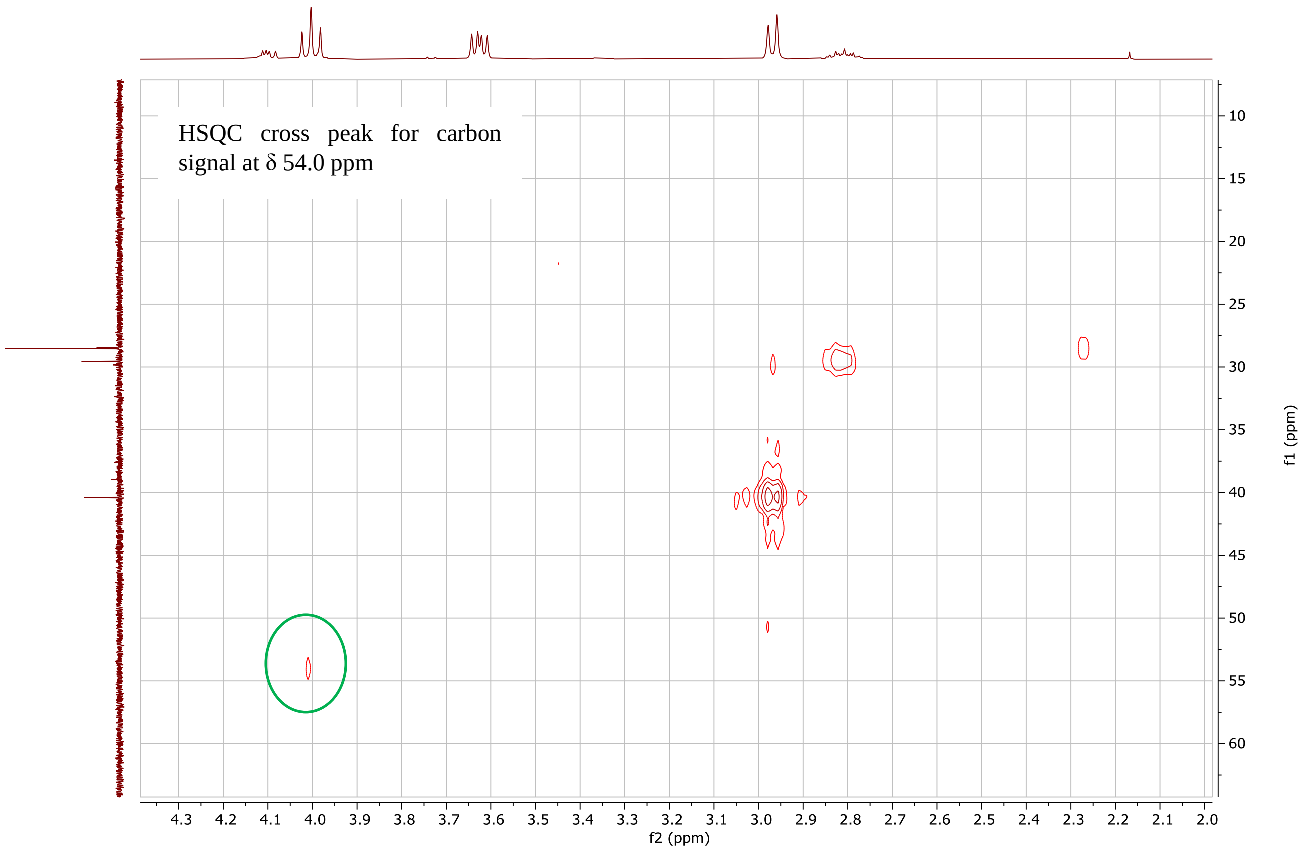
HSQC cross peak for carbon signal at δ 54.0 ppm
f2 (ppm)
f1 (ppm)

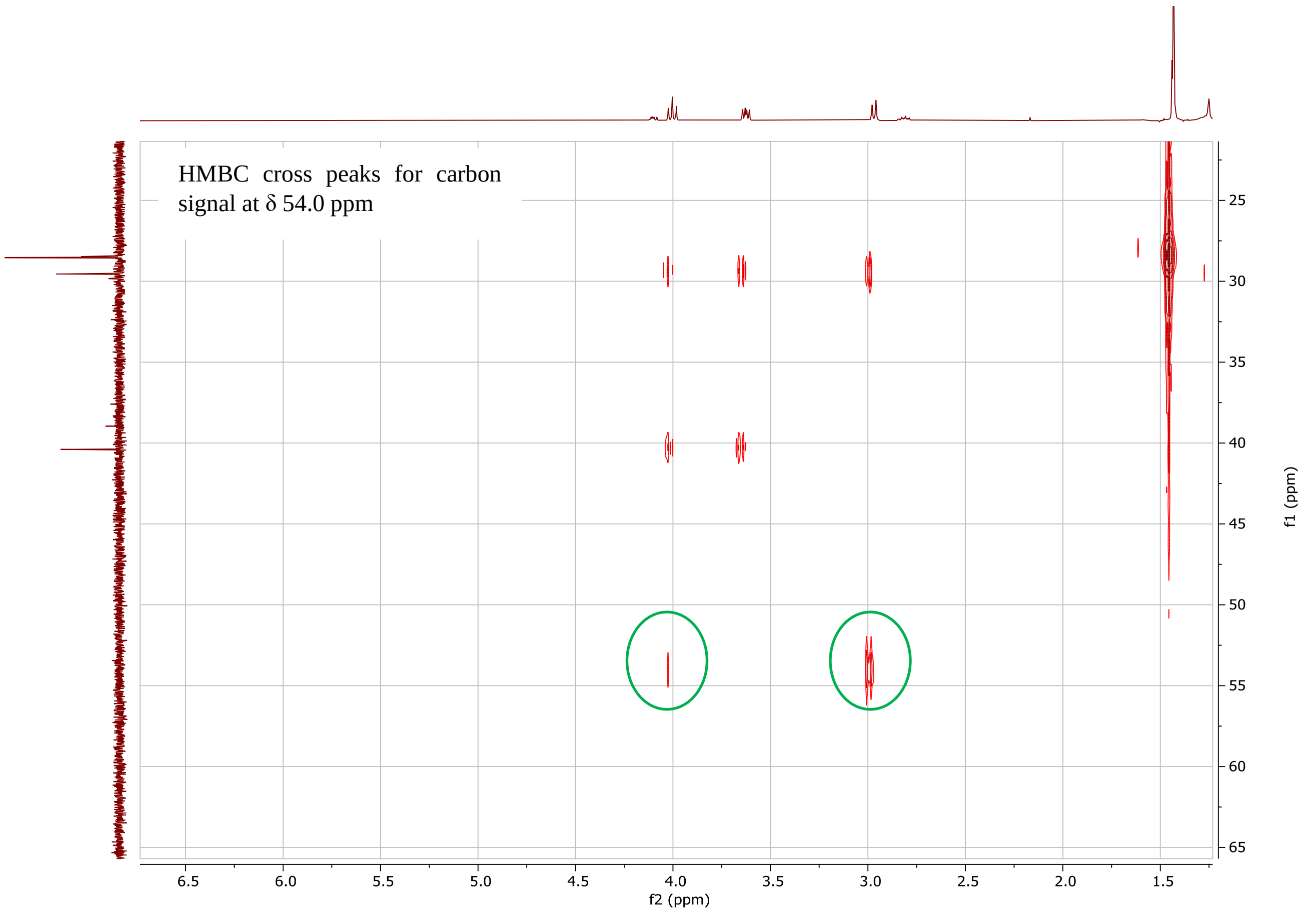
HMBC cross peaks for carbon signal at δ 54.0 ppm
f2 (ppm)
f1 (ppm)

4-(7-Hydroxy-3,7-dimethyloctyl)benzonitrile (19e)

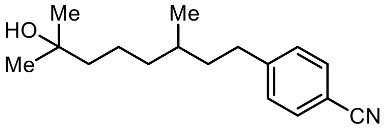

^{1}H NMR, 400 MHz, $CDCl_3$

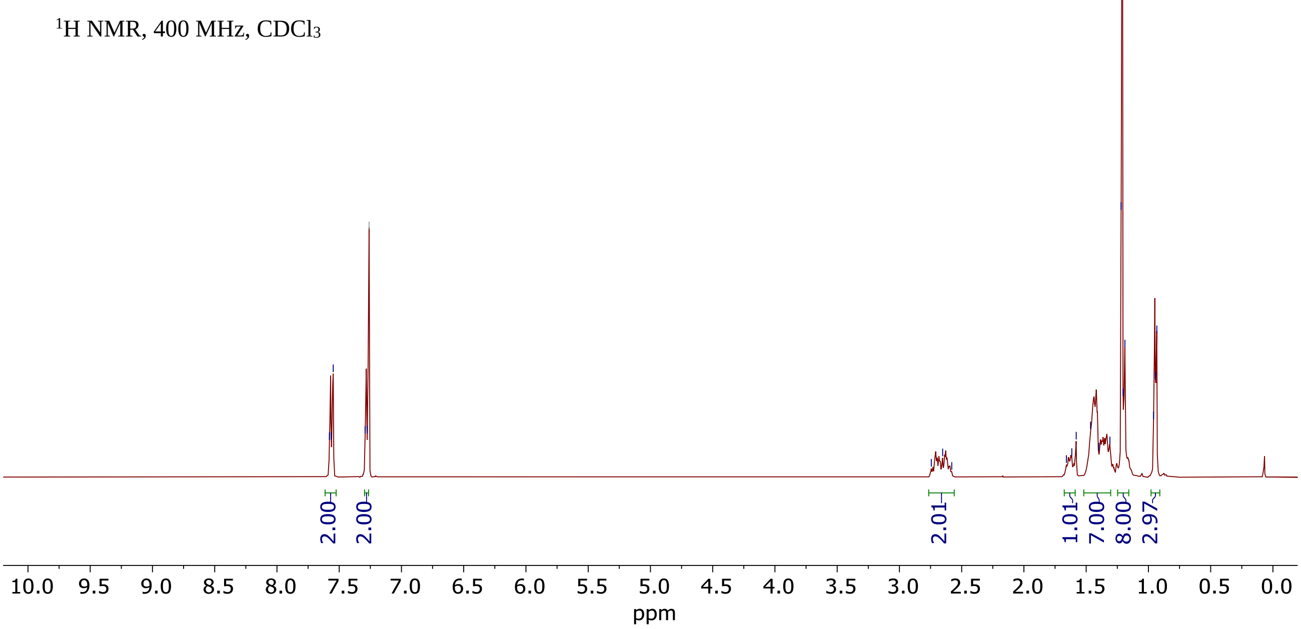

^{13}C NMR, 101 MHz, $CDCl_3$

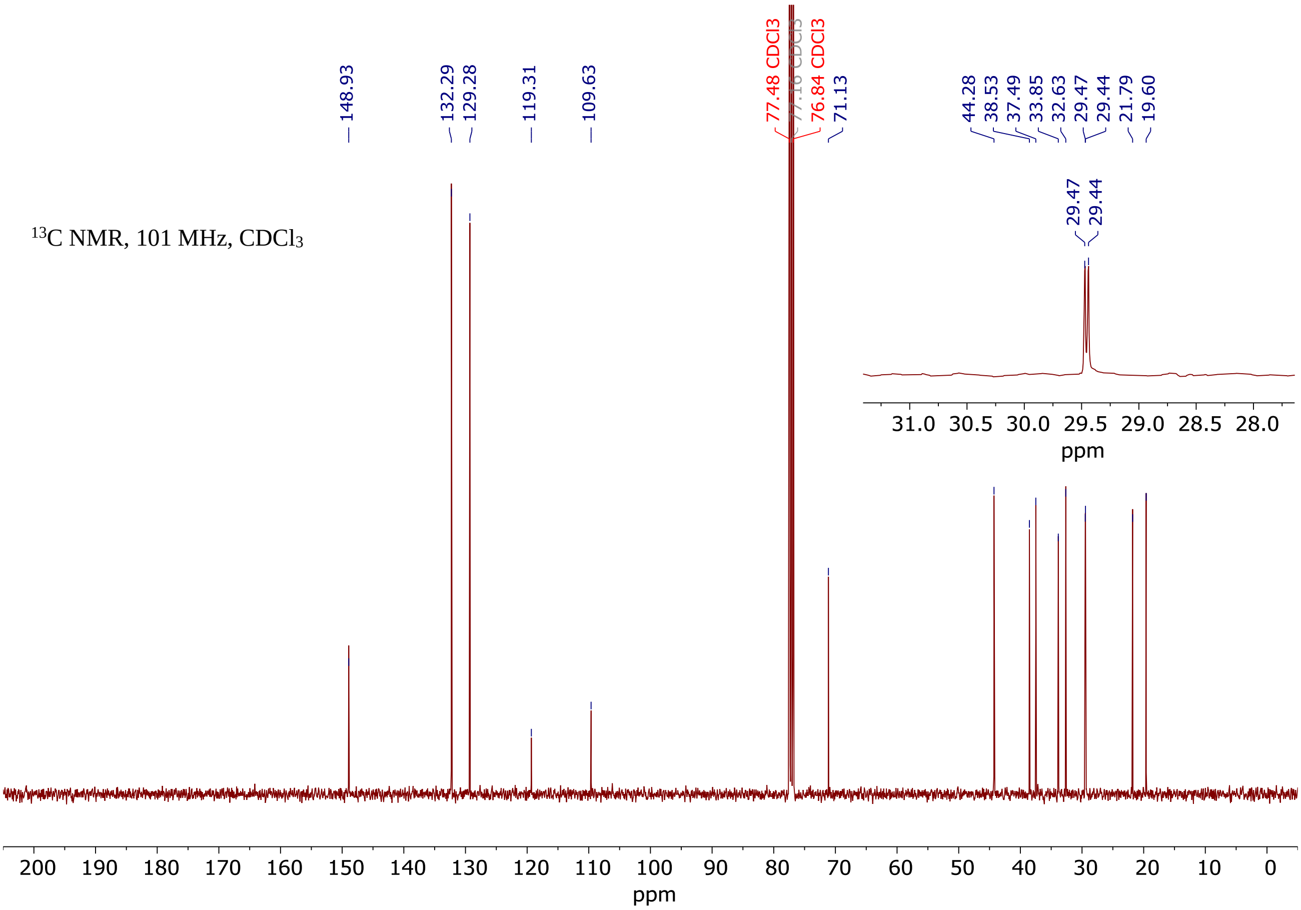

4-Benzylpyridine (19f)

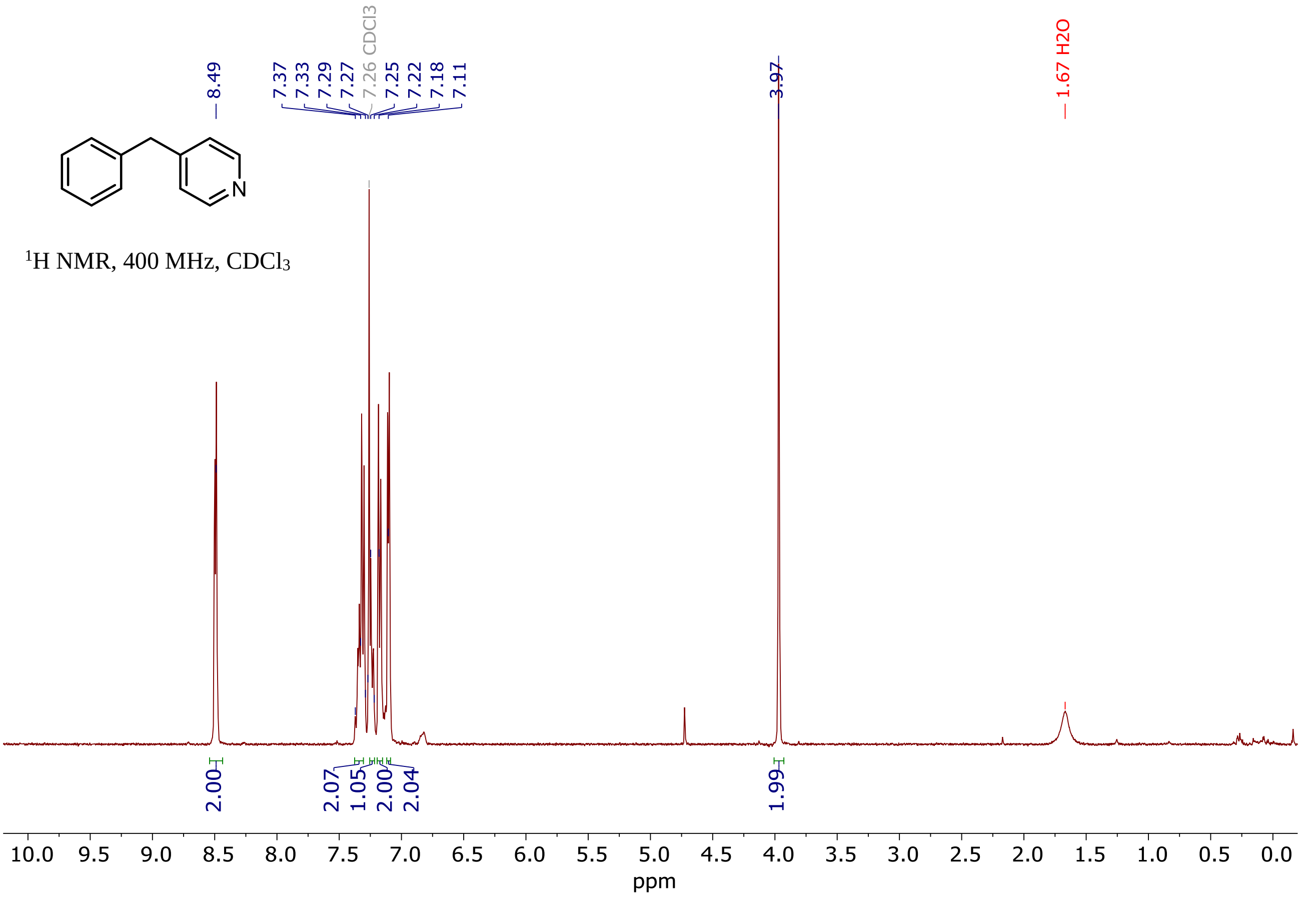

^{1}H NMR, 400 MHz, $CDCl_3$

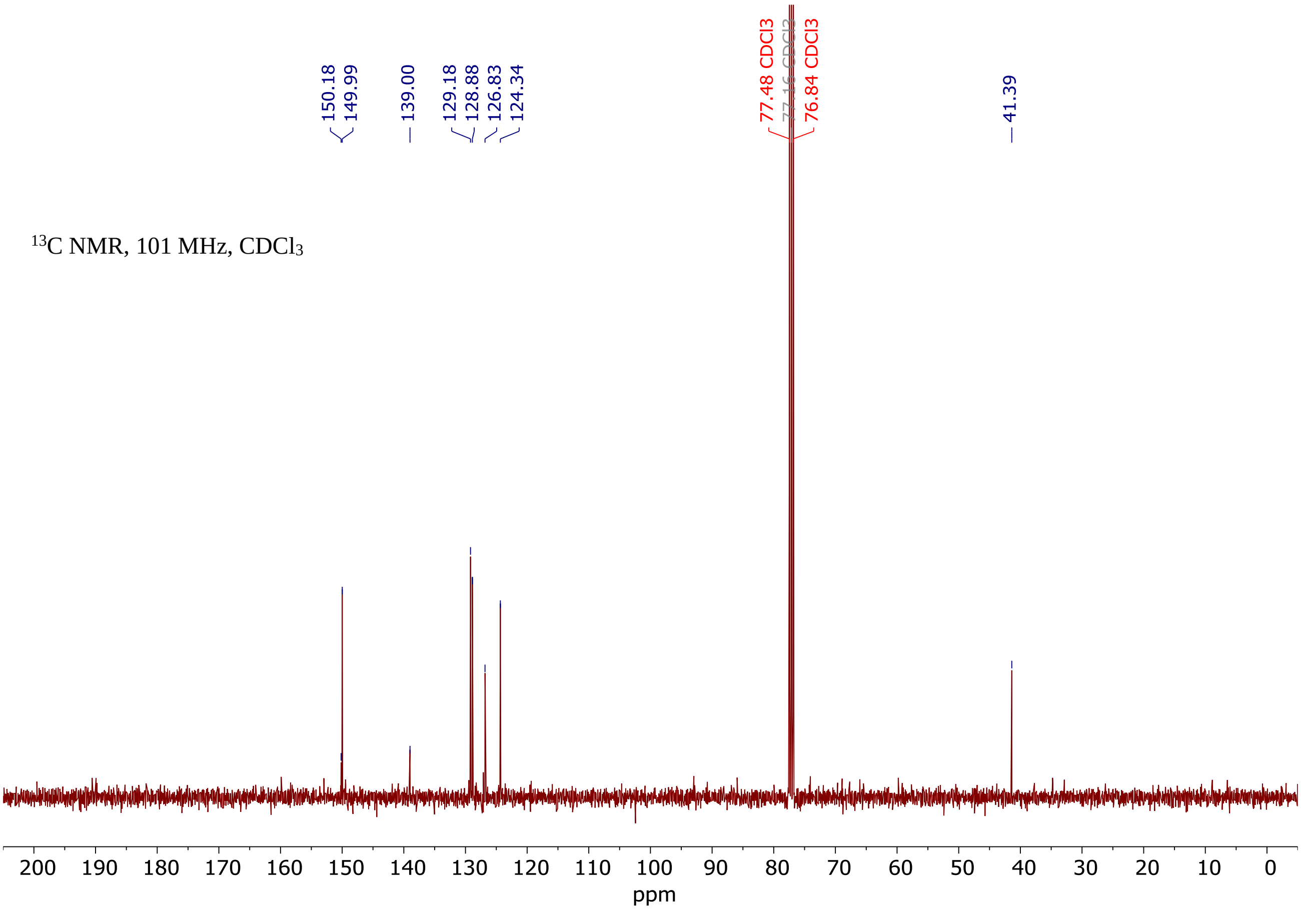

^{13}C NMR, 101 MHz, $CDCl_3$

4-(Thiophen-3-ylmethyl)pyridine (19g)

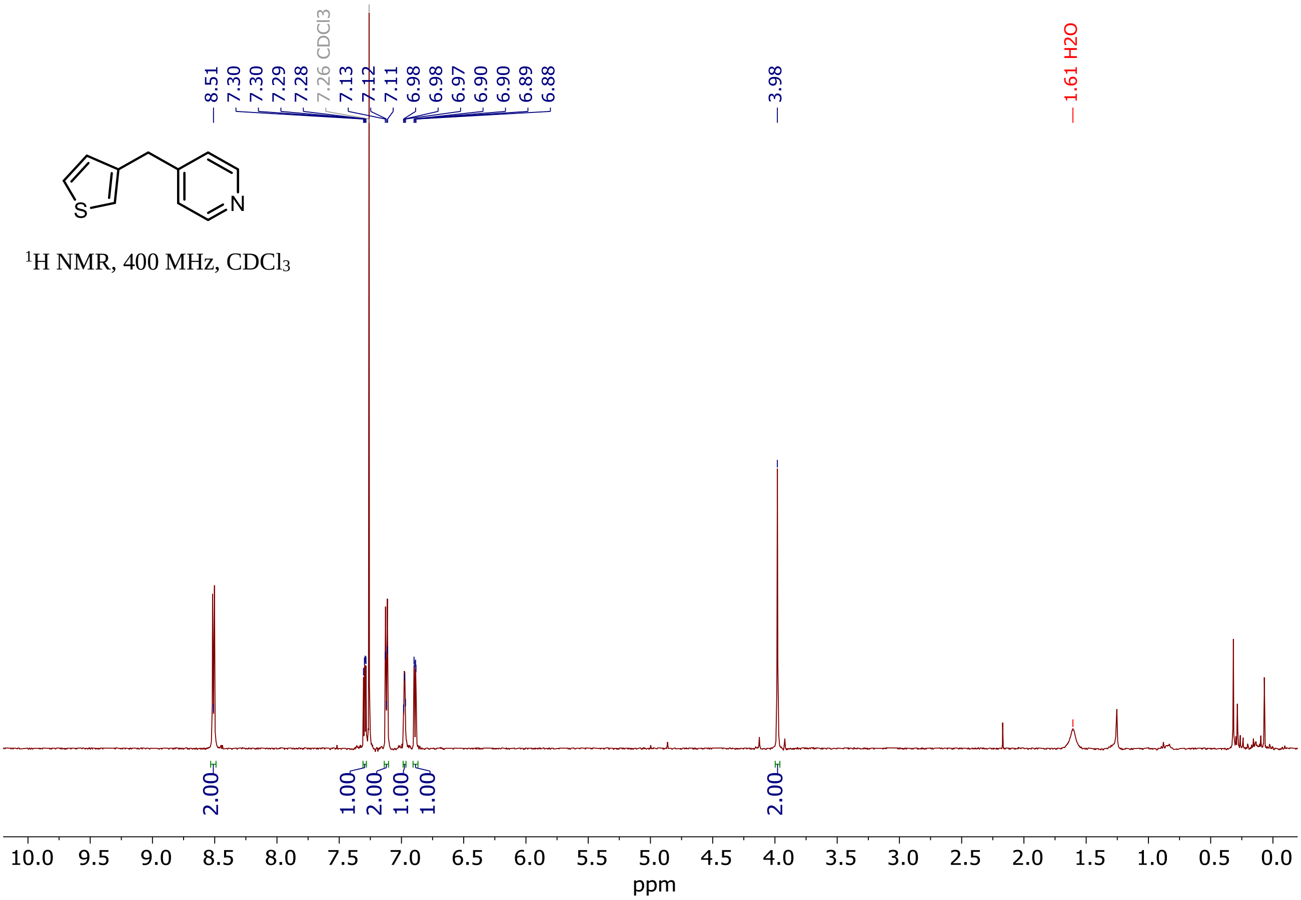

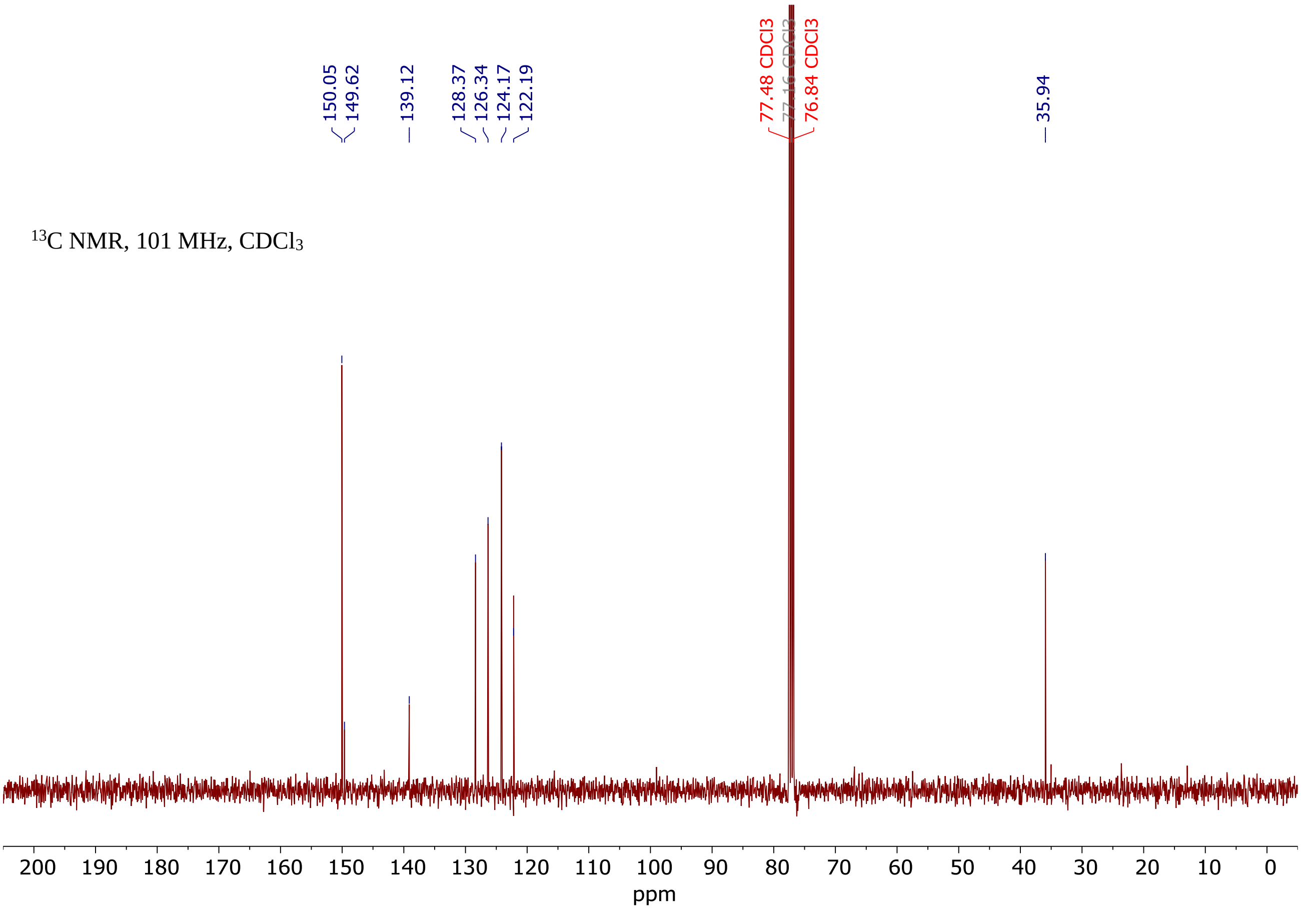

4-(4-(4-(4-Chlorophenyl)-4-hydroxypiperidin-1-yl)-1-(4-fluorophenyl)butyl)benzonitrile (19h)

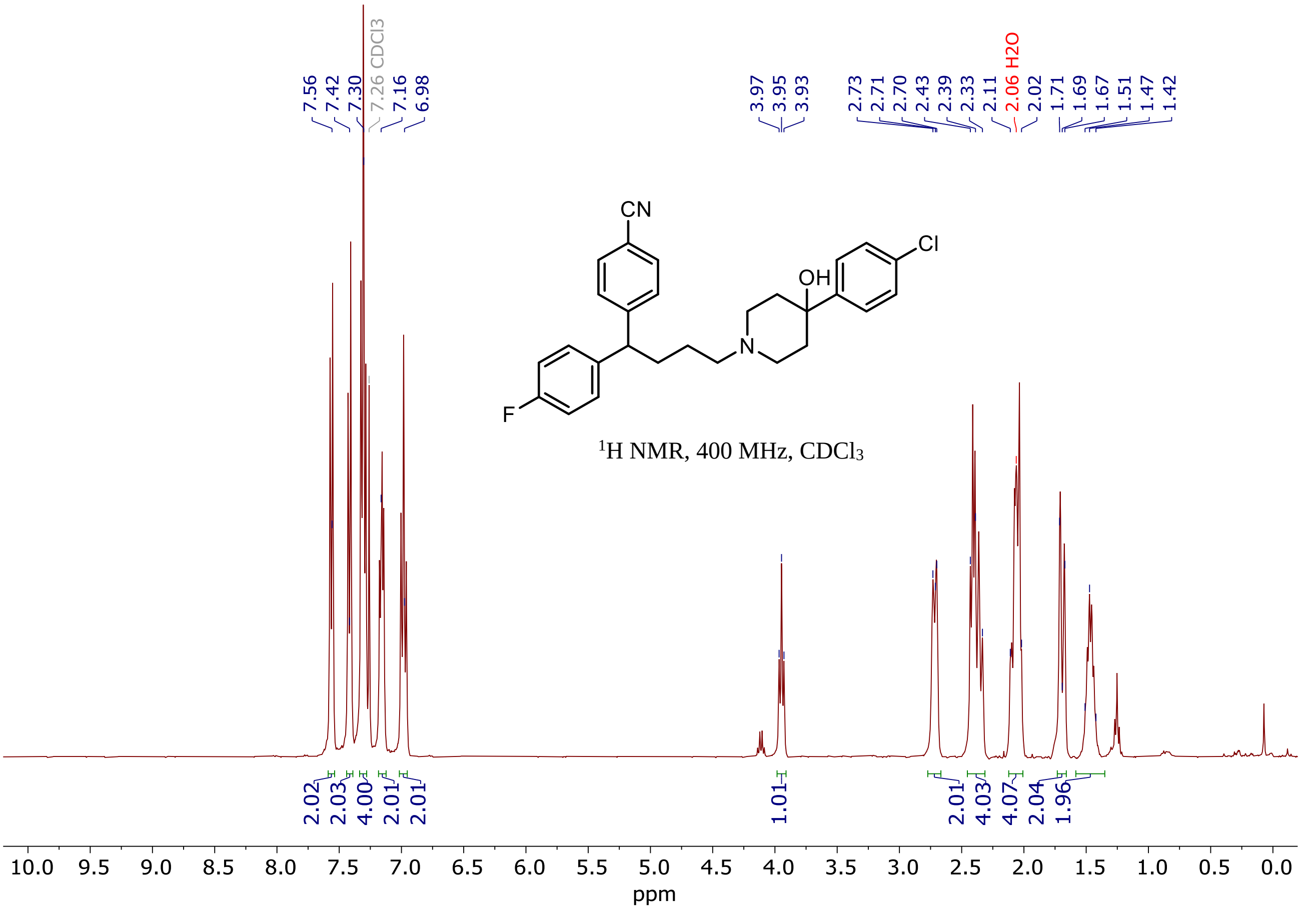

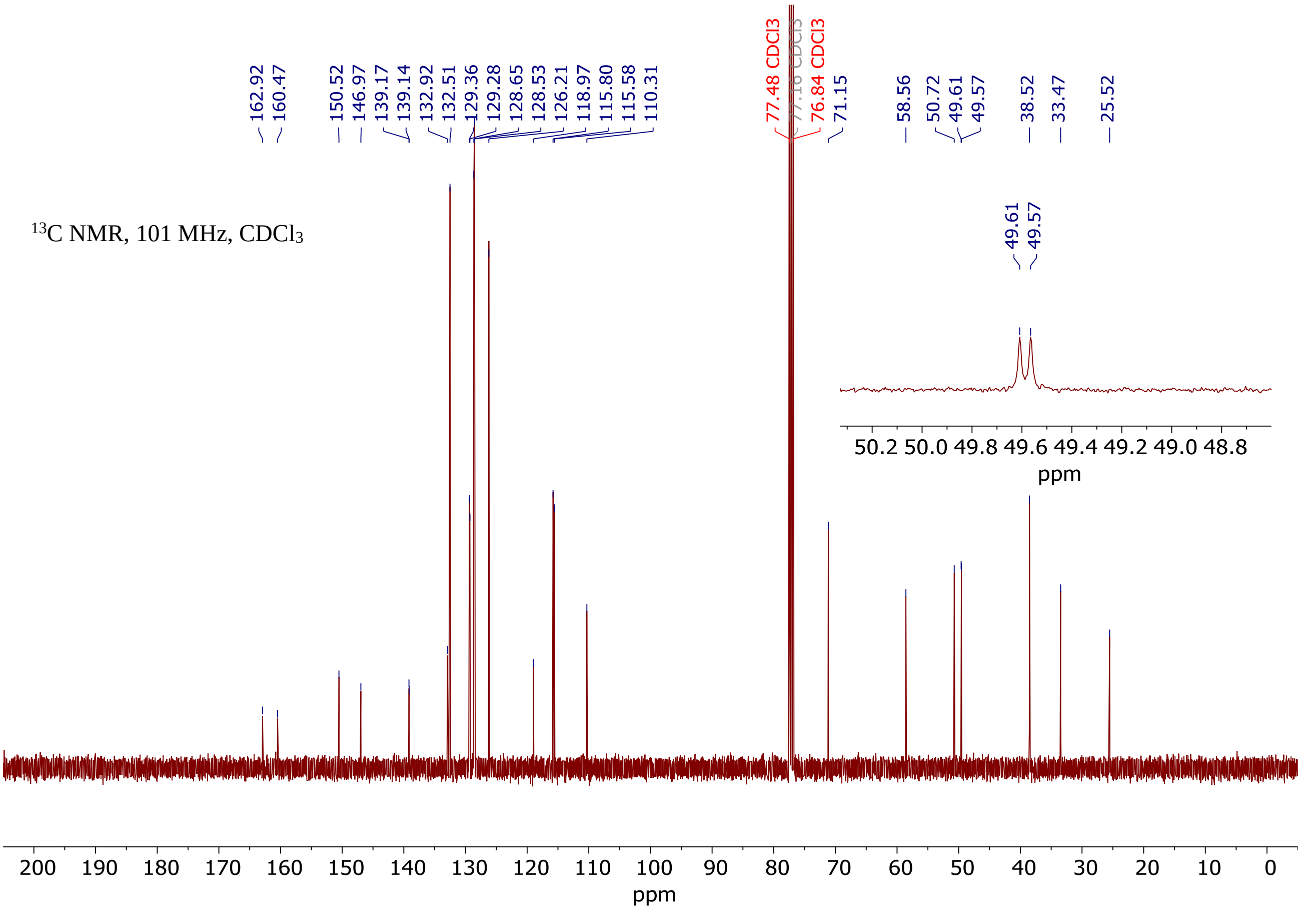

Supplement: Supplementary file 1 — ja4c08230_si_001.pdf [file ja4c08230_si_001.pdf]
